# Mass spectrometry identification of age-associated proteins from the malaria mosquitoes *Anopheles gambiae* s.s. and *Anopheles stephensi*

**DOI:** 10.1016/j.dib.2015.07.007

**Published:** 2015-07-16

**Authors:** Maggy T. Sikulu, James Monkman, Keyur A. Dave, Marcus L. Hastie, Patricia E. Dale, Roger L. Kitching, Gerry F. Killeen, Brian H. Kay, Jeffry J. Gorman, Leon E. Hugo

**Affiliations:** a Mosquito Control Laboratory, QIMR Berghofer Medical Research Institute, Brisbane, Queensland, Australia; b Protein Discovery Centre, QIMR Berghofer Medical Research Institute, Brisbane, Queensland, Australia; c Environmental Futures Research Institute and Griffith School of Environment, Griffith University, Brisbane, Queensland, Australia; d Environmental Health and Ecological Sciences Thematic Group, Ifakara Health Institute, Ifakara, United Republic of Tanzania; e Vector Biology Department, Liverpool School of Tropical Medicine, Liverpool, United Kingdom

## Abstract

This study investigated proteomic changes occurring in *Anopheles gambiae* and *Anopheles stephensi* during adult mosquito aging. These changes were evaluated using two-dimensional difference gel electrophoresis (2D-DIGE) and the identities of aging related proteins were determined using capillary high-pressure liquid chromatography (capHPLC) coupled with a linear ion-trap (LTQ)-Orbitrap XL hybrid mass spectrometry (MS). Here, we have described the techniques used to determine age associated proteomic changes occurring in heads and thoraces across three age groups; 1, 9 and 17 d old *A. gambiae* and 4 age groups; 1, 9, 17 and 34 d old *A. stephensi*. We have provided normalised spot volume raw data for all protein spots that were visible on 2D-DIGE images for both species and processed Orbitrap mass spectrometry data. For public access, mass spectrometry raw data are available via ProteomeXchange with identifier PXD002153. A detailed description of this study has been described elsewhere [1].

**Specifications Table**

**Table:** 

Subject area	Proteomics
More specific subject area	Protein analysis, MS
Type of data	Images, tables
How data was acquired	2D-DIGE
Data format	Typhoon 9000 and 94,000 images, raw and analysed
Experimental factors	Changes in protein abundance was evaluated at 1,9 and 17 d of age for *A. gambiae* and at 1, 9, 17 and 34 d for *A. stephensi*
Experimental features	Exploratory data for establishing age related biomarkers of malaria vectors
Data source location	QIMR Berghofer Medical Research Institute, Brisbane, Australia, Ifakara Health Institute, Tanzania
Data accessibility	LTQ-Orbitrap XL hybrid MS processed data is within this article but raw MALDI-TOF/TOF and LTQ-Orbitrap XL hybrid MS data is available in a public repository via ProteomeXchange with identifier PXD002153

**Value of the data**

• This study investigated the changes that occur in the head and thorax proteome of two major malaria vectors; the African and Asian malaria vectors, *A. gambiae* and *A. stephensi* respectively, during adult aging.
• These changes may highlight underlying mechanisms of the relationship between mosquito age and factors affecting *Plasmodium* transmission and mosquito control.
• A future study could use these data to identify robust age biomarkers to be incorporated into rapid age detection assays such as ELISA or dipstick assays applicable as surveillance tools in vector control settings.

## 1 Experimental design, materials and methods

### 1.1 Mosquito preparation and protein extraction

Two cohorts each of *A. gambiae* and *A. stephensi* were reared at different times. For each cohort, adults were collected 24 h post emergence and again at 9 and 17 d post emergence for *A. gambiae* and at 9, 17 and 34 d post emergence for *A. stephensi*. Heads and thoraces of five females at 1, 9 and 17 d of age for *A. gambiae* and 1, 9, 17 and 34 d for *A. stephensi* were dissected on a bed of dry ice and pooled into 2 ml plastic screw-cap vials containing 90 µl of 2D buffer (7 M Urea, 2 M Thiourea and 4% [w/v] CHAPS), 1×dissolved PhosSTOP (Roche, Penzerb, upper Bavaria, Germany) and two 3 mm silica glass beads. Proteins were extracted by homogenising samples using a Minibead-beater 96 (BioSpec Products, Inc. Bartlesville, OK, USA) for 1.45 min. The contents were collected by brief centrifugation at 12,000×g. The sample was transferred to new 1.5 ml micro-centrifuge tubes and clarified by centrifuging twice at 21,000×g for 10 min. Protein was purified using the 2D clean-up kit (GE Healthcare, Waukesha, WI, USA.) according to the manufacturer׳s protocol. Samples were then resuspended in 2D buffer and total protein content was quantified using the 2D quantification kit (GE Healthcare) following the manufacturer׳s protocol. Fifty micrograms of protein was prepared in a 2D buffer for each age sample. An internal standard sample was prepared by combining 25 µg of protein from each age sample into a single pool. The pH of all the samples was adjusted to between 8 and 9.1. A total of four and three biological replicates of *A. gambiae* and *A. stephensi*, respectively, were included at each age and the entire experiment was replicated a second time using the second cohort of mosquitoes.

### 1.2 2D-DIGE

Experiments with 2D-DIGE followed the procedures described by Hugo and colleagues [2]. The Stock solutions of each of Cy2, Cy3 and Cy5 fluorescent cyanine dyes (GE Healthcare) was prepared by reconstituting the appropriate dye in 5 µl Dimethylformamide (Sigma Chemical Co. Ltd., St Louis, MO, USA.) to a concentration of 1 nM. Four hundred picomoles of either Cy3 or Cy5 was added to each sample and 2400 pmol of Cy2 was added to the internal standard. Each sample pool was combined with 4.5 µl of 100×ampholytes (Bio-Rad, Richmond, CA, USA.), 5.6 µl Destreak reagent (GE Healthcare) and made up to 450 µl in 2D Buffer. Samples were loaded onto 24 cm, pH 3–10 immobilised pH gradient strips (Bio-Rad) in rehydration trays and left at room temperature for 4 h for the strips to absorb the sample. Following absorption, the strips were transferred to a new focussing tray, overlayed with mineral oil and allowed to rehydrate overnight at room temperature. For first dimension separation, the strips were transferred to a dry protean iso-electric focusing tray. The strips were overlayed with fresh mineral oil and iso-electric focussing was performed using the following run conditions: 50 μA per strip, 250 V for 15 min, 1000 V for 5 h, 10,000 V for 4 h and 500 V to reach a total of 80,000 V h^−1^. Focus strips were equilibrated for 15 min in buffer I containing 6 M Urea, 0.375 M Tris–HCl pH 8.8, 2% [w/v] SDS, 20% [v/v] glycerol and 2% [w/v] DTT and then for 15 min in buffer II containing 6 M Urea, 0.375 M Tris–HCl, 2% [w/v] SDS, 20% [v/v] glycerol and 2.5% [w/v] Iodoacetamide. The strips were placed on 12% acrylamide gels cast in 24 cm optically clear plates. Electrophoresis was performed in a Protean^®^ Plus Dodeca cell (Bio-Rad) at 5 mA/gel for 15 min, 10 mA/gel for 15 min and 30 mA/gel until the dye front reached the bottom of the gel.

### 1.3 Imaging and processing

*A. stephensi* gels were scanned using a Typhoon FLA-9400 fluorescent imager (GE Healthcare) at 100 µm pixel resolution, using the 520 band pass (BP) 40 filter for Cy2, 580 BP 30 filter for Cy3 and the 670 BP 30 filter for Cy5 emission. *A. gambiae* gels were scanned using a FLA-9000 Starion fluorescent imager (GE Healthcare) at 100 µm pixel resolution using the BPB1 (530DF20) filter for Cy2, DGR1 (570DF20/665LP) filter for Cy3 and LPR (665LP) filter for Cy5. Fluorescent images were processed and analysed using Delta2D version 4.0 software (Decodon, Greifswald, Germany). Spots on gel images scanned from the same gel were then connected by direct warping and the spots on all gels were aligned and normalised for intensity using the Cy2 channel (Internal standard images) on each gel with the match vectors tool. All aligned images were fused and thereafter spot validations were transferred to all other images from this fused image [3]. All spots that were visible on 2D-DIGE images for both species are shown in Supplementary tables 1–4.

### 1.4 Sample preparation for MS

Protein spots for identification were manually excised from 2D gels using the method of Hugo and colleagues [2]. The gels were stained with colloidal Coomassie 0.12% [w/v] brilliant blue G250 stain (Sigma Chemical Co., Ltd.), 10% [w/v] NH_4_SO_4_, 10% [v/v] phosphoric acid and 20% [v/v] methanol in distilled water. The differentially expressed spots were excised from these gels using a glass plug cutter and destained overnight in 300 µl of MS fix solution containing 40% [v/v] ethanol and 10% [v/v] acetic acid. The solution was discarded and 100 µl of 25 mM ammonium bicarbonate (Sigma Chemical Co., Ltd.), pH 8, was added and the samples were placed on an orbital shaker at room temperature for 15 min. The solution was discarded and the ammonium bicarbonate wash was repeated twice more. Gel plugs were dried in a vacuum concentrator for 30 min. The gels were rehydrated in 4 µl of 1 µg/µl proteomics grade trypsin (Sigma Chemical Co., Ltd.) in 40 mM ammonium bicarbonate containing 10% [v/v] acetonitrile (ACN) and incubated for 1 h at room temperature. A further 35 µl of 40 mM ammonium bicarbonate containing 10% [v/v] ACN was added and the plugs were incubated overnight at 37 °C. Tryptic extracts bathing the plugs from equivalent spot samples were pooled into a single tube. 20 µl of 50% [v/v] ACN containing 0.1% [v/v] Triflouroacetic acid (TFA) (Sigma Chemical Co., Ltd.) was added to the gel plugs and incubated at room temperature, shaking for 1 h. The solution bathing the gels was combined with the previous extracts. Pooled extracts were lyophilised, rehydrated in 30 μl of 0.1% [v/v] TFA and concentrated using C18 Zip-Tips (Millipore, Billerica, MA, USA.) according to the manufacturer׳s instructions. In-gel tryptic digests were analysed by either MALDI-TOF/TOF MS [4] or separated by CapHPLC and sprayed directly into the ion source of LTQ-Orbitrap XL hybrid MS [5].

### 1.5 MALDI-TOF/TOF analysis

All mass spectra from the in-gel tryptic digests were acquired in positive ion mode on an Ultraflex III MALDI-TOF/TOF MS (Bruker Daltonik GmbH, Bremen, Germany). Reflectron mode was used to measure the spectra with typical resolution within the range of 15,000 and 20,000. Mass accuracies of within 50 ppm for MS measurements and between 60 and 250 ppm for tandem MS (MS/MS) measurements were obtained. Saturated α-Cyano-4-hydroxy cinnamic acid (α-CHCA) matrix was prepared in 97% [v/v] acetone containing 0.3 mM ammonium dihydrogen phosphate and 0.1% [v/v] TFA, subsequently diluted 15 times in 6:3:1 ethanol:acetone:10 mM ammonium dihydrogen phosphate and 0.1% [v/v] TFA. This was used as matrix for all MS and MS/MS analyses at a sample to matrix ratio of 1:2. MS/MS spectra were automatically acquired with 40% higher laser intensity than that used for MS analysis. Spectra were calibrated using a peptide calibration standard mixture (Bruker Daltonics. Kit number 208241) of nine peptides in the mass range of *m*/*z*=1046 and *m*/*z*=3147, with 62.5 fmol of each peptide applied to calibration spots. High precision MS/MS calibration was achieved using fragment ions derived from all the nine peptides in the MS calibration kit and the associated calibration coefficients were applied to the method file used to acquire MS/MS data. The data were automatically acquired and processed in a batch mode by Bruker Daltonics Flex-series software (FlexControl and FlexAnalysis version 2.4), and searched using MASCOT search engine with a custom made database using Bio-tools version 3.1. Instrument settings for MS were: ion source 1 potential, 25.00 kV; ion source 2 potential, 21.70 kV; reflectron 1 potential, 26.30 kV; reflectron 2 potential, 13.85 kV and for MS/MS were: ion source 1 potential, 8.00 kV; ion source 2 potential, 7.20 kV; reflectron 1 potential, 29.50 kV; reflectron 2 potential, 13.75 kV; LIFT 1 voltage, 19.00 kV; LIFT 2 voltage, 3.00 kV. Ion selector resolution was set at 0.5% of the mass of the precursor ion [6]. All protein spots identified by MALDI-TOF/TOF mass spectrometry are reported in our accompanying manuscript [1].

### 1.6 Liquid chromatography (LC)–MS/MS analysis

**Fig. 1:**
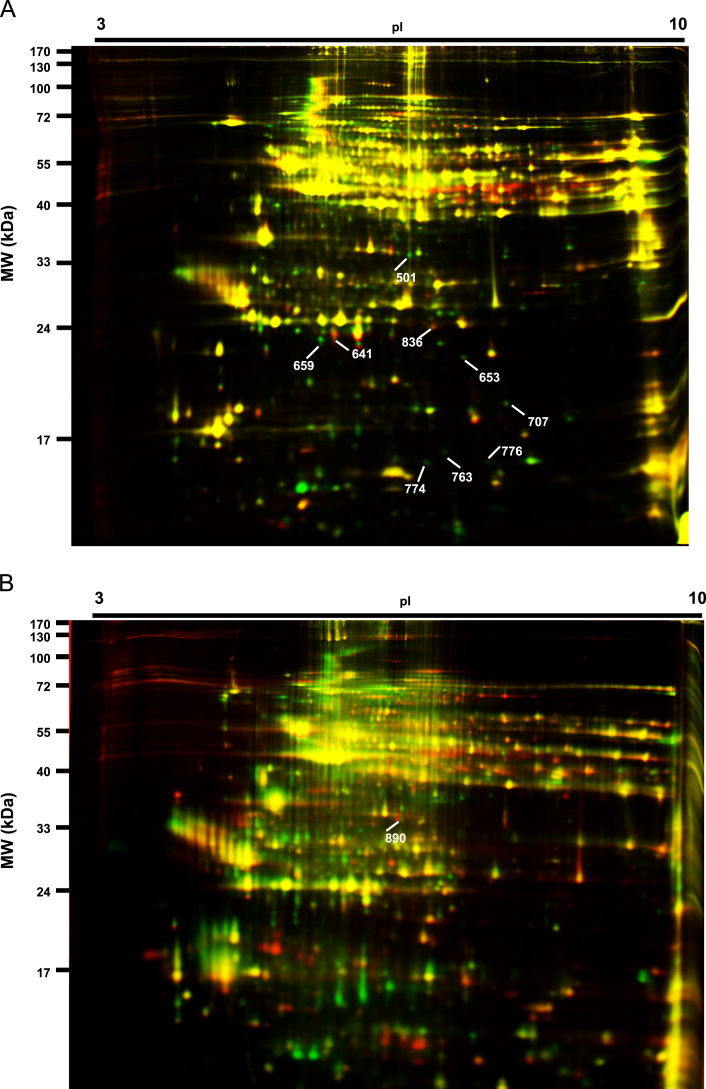
Protein spots detected on 2D-DIGE images. Candidate aging biomarker proteins identified by CapHPLC LTQ-Orbitrap XL hybrid MS for *A. stephensi* (**Panel A)** and *A. gambiae***(Panel B)**.

In-gel tryptic digests were fractionated by CapHPLC using a Shimadzu Prominence HPLC system (Shimadzu) and were introduced directly into the LTQ-Orbitrap XL hybrid MS (Thermo Fisher Scientific, Bremen, Germany) equipped with a dynamic nanoelectrospray ion source (Proxeon, Odense, Denmark) and distal coated silica emitters (30 μm i.d., 20 μm tip i.d; New Objective, Woburn, MA, USA.). Acidified samples were loaded onto a 120 Å, 3 μm particle size, 300 μm by10 mm C18-AQ Reprosil-Pur trap column (SGE Australia Pty., Ltd.) at 30 μl/min in 98% solvent A (0.1% [v/v] aqueous formic acid) and 2% solvent B (80% [v/v] acetonitrile containing 0.1% [v/v] formic acid) for 3 min at 40 °C, and were subsequently gradient eluted onto a pre-equilibrated self-packed analytical column (Dr. Maisch GmbH Reprosil-Pur C18-AQ, 120 Å, 150 μm by 200 mm) using a flow rate of 900 nl/min. The LTQ-Orbitrap was controlled using Xcalibur 2.0 SR1 (Thermo Fisher Scientific). Analyses were carried out in data-dependent acquisition mode, whereby the survey full scan mass spectra (*m*/*z* 300–2000) were acquired in the Orbitrap FT mass analyser at a resolution of 60,000 (at 400*m*/*z*) after accumulating ions to an automatic gain control target value of 5.0×10^5^ charges in the LTQ mass analyser. MS/MS mass spectra were concurrently acquired on the eight most intense ions in the full scan mass spectra in the LTQ mass analyser to an automatic gain control target value of 3.0×10^4^ charges. Charge state filtering, where unassigned precursor ions were not selected for fragmentation, and dynamic exclusion (repeat count, 1; repeat duration, 30 s; exclusion list size, 500; and exclusion duration, 90 s) were used. Fragmentation conditions in the LTQ were: 35% normalised collision energy, activation q of 0.25, isolation width of 3.0 Da, 30 ms activation time, and minimum ion selection intensity 500 counts. Maximum ion injection times were 500 ms for survey full scans and 100 ms for MS/MS [3,5–7]. All spots identified by LC-MS/MS are shown in Fig. 1 and their identities are presented in Table 1.

### 1.7 Data analysis

Protein identifications were performed by searching peptide peak lists detected by TOF/TOF MS and MS/MS against in-silico databases of theoretical trypsin digests of published proteins using an in-house MASCOT database search engine (Version 2.2, Matrix Sciences, London) using Biotools version 3.1 (Bruker Daltonics). The LC-MS/MS data were processed and searched against the same database using an in-house MASCOT database search engine integrated in Proteome Discoverer (version 1.4.0.288, Thermo Fisher Scientific) software. The database was compiled from UniProtKB database on 22/05/2012 and from VectorBase database on 26/05/14. A mass tolerance of 100 ppm for peptide precursor ions and 0.8 Da for fragment ions was applied in all searches except for Orbitrap dataset where a precursor ion tolerance of 20 ppm was applied. Database search parameters for both MALDI-TOF/TOF and LTQ-Orbitrap datasets were as follows: enzymatic cleavage, tryptic; fixed modifications, S-carboxamidomethylation of cysteine residues; variable modifications, methionine oxidation, deamidation of asparagine and glutamine; and missed cleavages. Peptides identified by TOF/TOF mass spectrometry were reported as significant scoring peptides at a MASCOT probability based score threshold of *p*<0.05 (<5% chance to be a false positive hit). For the Orbitrap datasets, to estimate the false discovery rate (FDR) at which the identification was considered correct (*q*-Value), a Percolator algorithm implemented in Proteome Discoverer (version 1.4.0.288, Thermo Fisher Scientific) was used and proteins with peptides having a confidence threshold *q*-Value<0.01 (1% FDR) were considered to be valid. A minimum of two confident peptide identifications were required to assign a protein identity. Putative function of hypothetical proteins was inferred using the VectorBase database.

## Figures and Tables

**Table 1:** Identities of age associated *A. stephensi* and *A. gambiae* spots shown in Fig. 1. Identities were determined using CapHPLC LTQ-Orbitrap XL hybrid MS/MS and MASCOT database searching.

**Spot #**	**Protein name**	**Score**	**Coverage**	**No. of Proteins**	**No. of Unique peptides**	**No. of PSMs**	**No. of AA**	**Mw [kDa]**	**Calc. pI**
641^a^	ASTEI05637-PA hypothetical protein	950.92	68.02	1	13	68	172	19.5	5.95
	ASTEI06099-PA hypothetical protein	311.69	33.90	1	8	35	177	20.4	5.91
	ASTEI09076-PA hypothetical protein	36.01	14.29	2	2	2	189	21.4	5.73
707^a^	ASTE011623-PA hypothetical protein	37.91	14.04	2	2	2	178	19.3	8.18
	ASTEI01010-PA hypothetical protein	69.26	27.65	2	4	7	217	23.7	9.20
763^a^	ASTEI10910-PA hypothetical protein	64.46	21.26	1	2	2	127	13.9	6.38
	ASTE002628-PA hypothetical protein	52.76	18.25	3	2	3	137	14.3	7.84
774^a^	ASTEI052400-PA hypothetical protein	48.74	21.74	2	2	3	92	10.2	11.18
659^a^	ASTEI05637-PA hypothetical protein	646.94	55.23	1	8	30	172	19.5	5.95
	ASTEI09076-PA hypothetical protein	78.65	13.23	2	2	2	189	21.4	5.73
	ASTEI06099-PA hypothetical protein	73.27	15.82	1	4	6	177	20.4	5.91
653^a^	ASTEI06860-PA hypothetical protein	387.50	49.50	1	8	38	202	21.8	8.29
501^a^	ASTEI09467-PA hypothetical protein	514.91	29.55	2	2	38	352	38.1	6.95
	ASTEI07484-PA hypothetical protein	88.05	16.25	2	4	7	277	31.9	5.86
	ASTEI06665-PA hypothetical protein	75.28	10.84	1	3	5	286	30.9	6.99
	ASTE001036-PA hypothetical protein	69.42	12.94	2	4	4	309	34.2	6.74
	ASTEI00995-PA hypothetical protein	41.85	10.43	2	2	2	163	18.5	8.62
	ASTE002048-PA hypothetical protein	41.39	8.85	3	2	2	192	21.9	8.40
	ASTE011344-PA hypothetical protein	42.68	6.60	5	2	2	318	34.6	6.54
836^a^	ASTEI01727-PA hypothetical protein	70.13	20.11	2	3	4	184	21.1	4.79
	ASTEI05226-PA hypothetical protein	49.86	8.97	3	2	2	223	25.1	6.21
890^b^	AGAP004031-PA Mitochondrial electron transfer flavour protein subunit alpha	187.85	24.40	2	6	9	332	34.6	8.57
	AGAP005627-PC creatine kinase	48.78	7.32	7	2	2	355	39.7	6.47
	AGAP009833-PA Voltage-dependent anion-selective channel protein 2	39.70	8.51	4	2	2	282	30.7	8.56
	AGAP008278-PA Long form D7 salivary protein	31.25	6.11	1	2	3	311	35.6	5.90
	AGAP006936-PB Mitochondrial Cytochrome c1 haem protein	0.00	8.08	4	2	2	297	32.7	8.46

A mass tolerance of 20 ppm for peptide precursor ions was applied in all searches. Multiple protein hits were typically achieved from spot identifications using CapHPLC; possibly due to inefficient isoelectric focusing or electrophoresis and/or high sensitivity of the LTQ-Orbitrap. Therefore, only protein hits with estimated size and charge states within arbitrary ranges of±5 kDa and±1 pI from the actual spot positions were included in the table. At least two peptide identifications were required to assign protein identification.

a *A. stephensi* proteins pots as shown in Fig. 1A.

b *A. gambiae* protein spot as shown in Fig. 1B.

## Appendix A Supplementary materials

**Supplementary material: Supplementary Table 1:** Normalised spot volume data for all spots detected on 2D-DIGE images of cohort 1 of *A. gambiae*. Spots highlighted in yellow were found to be age-associated. **Supplementary Table 2:** Normalised spot volume data for all spots detected on 2D-DIGE images of cohort 2 of *A. gambiae*. Spots highlighted in yellow were found to be age-associated. **Supplementary Table 3:** Normalised spot volume data for all spots detected on 2D-DIGE images of cohort 1 of *A. stephensi*. Spots highlighted in yellow were found to be age-associated. **Supplementary Table 4:** Normalised spot volume data for all spots detected on 2D-DIGE images of cohort 2 of *A. stephensi*. Spots highlighted in yellow were found to be age-associated. **External repository data:** The mass spectrometry proteomics data have been deposited to the ProteomeXchange Consortium [8] via the PRIDE partner repository with the dataset identifier PXD002153 and 10.6019/PXD002153.

## References

[1] Sikulu M.T., Monkman J., Dave K.A., Hastie M.L., Dale P.E., Kitching R.L., Killeen G.F., Kay B.H., Gorman J.J., Hugo L.E. (2015). Proteomic changes occurring in the malaria mosquitoes *Anopheles gambiae* and *Anopheles stephensi* during aging. J. Prot..

[2] Hugo L.E., Monkman J., Dave K.A., Wockner L.F., Birrell G.W., Norris E.L., Kienzle V.J., Sikulu M.T., Ryan P.A., Gorman J.J. (2013). Proteomic biomarkers for ageing the mosquito *Aedes aegypti* to determine risk of pathogen transmission. PloS One.

[3] Hastie M.L., Headlam M.J., Patel N.B., Bukreyev A.A., Buchholz U.J., Dave K.A., Norris E.L., Wright C.L., Spann K.M., Collins P.L. (2012). The human respiratory syncytial virus nonstructural protein 1 regulates type I and type II interferon pathways. Mol. Cell Prot..

[4] Dave K.A., Headlam M.J., Wallis T.P., Gorman J.J. Preparation and analysis of proteins and peptides using MALDI TOF/TOF mass spectrometry, in: Proceedings of the Current Protocol in Protein Science. vol. Chapter 16, Unit 16.13, 2011. 10.1002/0471140864.ps1613s63 21400691

[5] Joubert D.A., Blasdell K.R., Audsley M.D., Trinidad L., Monaghan P., Dave K.A., Lieu K.G., Amos-Ritchie R., Jans D.A., Moseley G.W. (2014). Bovine ephemeral fever rhabdovirus α1 protein has viroporin-like properties and binds importin β1 and importin 7. J. virol..

[6] Dave K.A., Hamilton B.R., Wallis T.P., Furness S.G., Whitelaw M.L., Gorman J.J. (2007). Identification of N, Nε-dimethyl-lysine in the murine dioxin receptor using MALDI-TOF/TOF-and ESI-LTQ-Orbitrap-FT-MS. Int. J. Mass Spec..

[7] Dave K.A., Norris E.L., Bukreyev A.A., Headlam M.J., Buchholz U.J., Singh T., Collins P.L., Gorman J.J. (2014). A comprehensive proteomic view of responses of A549 type II alveolar epithelial cells to human respiratory syncytial virus infection. Mol. Cell Prot..

[8] Vizcaíno J.A., Deutsch E.W., Wang R., Csordas A., Reisinger F., Rios D., Dianes J.A., Sun Z., Farrah T., Bandeira N. (2014). ProteomeXchange provides globally coordinated proteomics data submission and dissemination. Nat. Biotech..

