# Supplementary material for: Mass spectrometry identification of age-associated proteins from the malaria mosquitoes Anopheles gambiae s.s. and Anopheles stephensi
Source: Data Brief. 2015 Jul 16;4:461–7. doi: 10.1016/j.dib.2015.07.007 (PMC4534588; doi:10.1016/j.dib.2015.07.007)
Supplement: Supplementary file 1 — Supplementary material: Supplementary Table 1: Normalised spot volume data for all spots detected on 2D-DIGE images of cohort 1 of A. gambiae. Spots highlighted in yellow were found to be age-associated. Supplementary Table 2: Normalised spot volume data for all spots detected on 2D-DIGE images of cohort 2 of A. gambiae. Spots highlighted in yellow were found to be age-associated. Supplementary Table 3: Normalised spot volume data for all spots detected on 2D-DIGE images of cohort 1 of A. stephensi. Spots highlighted in yellow were found to be age-associated. Supplementary Table 4: Normalised spot volume data for all spots detected on 2D-DIGE images of cohort 2 of A. stephensi. Spots highlighted in yellow were found to be age-associated. External repository data: The mass spectrometry proteomics data have been deposited to the ProteomeXchange Consortium [8] via the PRIDE partner repository with the dataset identifier PXD002153 and 10.6019/PXD002153. [file mmc1.zip › Supplimentary file/Supplementary Table 1.doc]

**Table 1**

| **Spot ID^a^** | **Normalized Vol. D1a** | **Normalized Vol. D1b** | **Normalized Vol. D1c** | **Normalized Vol. D1d** | **Normalized Vol. D9a** | **Normalized Vol.**  **D9b** | **Normalized Vol. D9c** | **Normalized Vol. D9d** | **Normalized Vol. D17a** | **Normalized Vol. D17b** | **Normalized Vol. D17c** | **Normalized Vol. D17d** |
| --- | --- | --- | --- | --- | --- | --- | --- | --- | --- | --- | --- | --- |
| ID168097 | 56.3 | 85.3 | 112.9 | 73.3 | 126.6 | 167.8 | 141.7 | 144.0 | 84.3 | 178.6 | 158.1 | 102.0 |
| ID168113 | 73.3 | 114.4 | 112.6 | 77.8 | 158.0 | 112.9 | 109.1 | 115.5 | 55.7 | 103.5 | 141.5 | 80.6 |
| ID168310 | 148.8 | 114.2 | 127.0 | 204.1 | 26.4 | 50.1 | 28.7 | 31.6 | 27.7 | 22.1 | 9.9 | 41.3 |
| ID168563 | 120.5 | 124.3 | 107.3 | 94.5 | 114.1 | 82.7 | 93.0 | 91.2 | 52.7 | 79.4 | 72.7 | 80.3 |
| ID168647 | 170.8 | 239.0 | 243.4 | 171.2 | 134.1 | 77.2 | 102.4 | 82.6 | 52.5 | 53.1 | 72.1 | 47.0 |
| ID170581 |  | 527.8 | 475.8 | 1092.1 |  | 202.0 | 77.4 | 170.7 | 19.8 | 174.4 | 47.2 | 49.4 |
| ID51310 | 130.1 | 143.6 | 244.9 | 117.5 | 136.8 | 121.4 | 329.6 | 119.2 | 96.8 | 104.6 | 144.1 | 142.4 |
| ID51334 | 52.0 | 116.1 | 91.1 | 47.4 | 86.1 | 89.3 | 102.0 | 128.7 | 100.8 | 122.6 | 111.8 | 75.9 |
| ID51341 | 92.2 | 132.0 | 105.6 | 110.7 | 94.3 | 112.6 | 118.1 | 125.1 | 105.8 | 108.9 | 136.3 | 116.4 |
| ID51352 | 82.7 | 115.3 | 94.3 | 93.8 | 84.1 | 103.8 | 87.2 | 138.8 | 111.2 | 112.4 | 119.9 | 106.9 |
| ID51355 | 55.3 | 144.8 | 122.9 | 108.0 | 109.4 | 95.3 | 89.0 | 196.2 | 97.3 | 91.4 | 139.6 | 104.0 |
| ID51356 | 52.2 | 100.4 | 60.6 | 66.8 | 83.1 | 83.0 | 93.6 | 123.8 | 122.4 | 186.8 | 98.3 | 85.4 |
| ID51358 | 128.8 | 107.1 | 72.2 | 86.0 | 104.2 | 121.6 | 69.0 | 93.4 | 111.7 | 118.6 | 125.0 | 93.5 |
| ID51360 | 50.6 | 81.1 | 80.3 | 55.8 | 90.9 | 87.8 | 89.5 | 133.9 | 117.0 | 120.6 | 111.3 | 82.8 |
| ID51366 | 22.7 | 14.8 | 141.3 | 134.5 | 21.5 | 113.8 | 107.0 | 216.6 | 17.6 | 121.6 | 160.6 | 132.6 |
| ID51367 | 72.3 | 87.5 | 104.5 | 70.4 | 106.3 | 95.2 | 100.8 | 106.4 | 95.9 | 129.6 | 98.1 | 81.1 |
| ID51375 | 32.0 | 103.8 | 157.5 | 130.6 | 37.9 | 115.9 | 139.8 | 536.7 | 58.5 | 116.4 | 160.2 | 260.0 |
| ID51398 | 14.9 | 5.0 | 67.5 | 128.7 | 10.9 | 113.3 | 93.5 | 118.4 | 18.6 | 145.8 | 166.1 | 108.1 |
| ID51399 | 63.9 | 76.1 | 95.5 | 44.5 | 99.0 | 82.2 | 104.6 | 114.1 | 80.5 | 117.6 | 114.2 | 70.5 |
| ID51400 | 66.0 | 48.4 | 85.9 | 54.3 | 86.0 | 87.9 | 116.1 | 125.1 | 89.9 | 148.8 | 100.7 | 80.0 |
| ID51426 | 100.7 | 107.5 | 67.4 | 88.5 | 114.7 | 122.5 | 71.6 | 112.8 | 103.4 | 108.0 | 133.8 | 116.6 |
| ID51430 | 86.4 | 89.3 | 65.9 | 99.5 | 110.0 | 110.2 | 59.8 | 125.8 | 109.3 | 104.0 | 101.3 | 114.4 |
| ID51460 | 18.3 | 88.7 | 51.4 | 88.4 | 80.5 | 115.8 | 51.5 | 49.3 | 69.1 | 108.2 | 102.4 | 32.1 |
| ID51471 | 209.3 | 125.2 | 34.1 | 48.2 | 107.2 | 77.0 | 22.3 | 60.8 | 48.8 | 91.9 | 60.2 | 179.5 |
| ID51473 | 146.7 | 56.2 | 64.5 | 89.4 | 113.7 | 586.6 | 61.4 | 36.5 | 39.2 | 325.8 | 64.0 | 43.8 |
| ID51474 | 278.2 | 144.8 | 74.6 | 88.8 | 174.0 | 106.2 | 49.0 | 62.4 | 54.7 | 95.3 | 96.3 | 230.9 |
| ID51476 | 299.1 | 190.6 | 80.1 | 90.2 | 164.0 | 107.2 | 28.0 | 43.5 | 44.6 | 81.9 | 93.9 | 175.6 |
| ID51480 | 237.9 | 163.5 | 76.7 | 96.3 | 150.2 | 134.2 | 35.6 | 39.8 | 70.2 | 86.4 | 111.2 | 192.5 |
| ID51481 | 338.9 | 143.2 | 42.1 | 46.4 | 160.2 | 94.4 | 23.9 | 49.6 | 46.8 | 91.1 | 72.2 | 183.2 |
| ID51486 | 103.9 | 27.9 | 125.0 | 111.5 | 113.8 | 79.2 | 148.5 | 206.2 | 28.4 | 44.4 | 102.3 | 311.3 |
| ID51487 | 212.3 | 189.7 | 121.8 | 173.3 | 103.5 | 92.4 | 20.9 | 33.7 | 25.8 | 50.0 | 70.6 | 157.9 |
| ID51491 | 22.7 | 473.8 | 473.1 | 222.1 | 173.4 | 297.4 | 117.9 | 97.5 | 624.5 | 410.7 | 204.6 | 61.5 |
| ID51492 | 132.7 | 121.4 | 86.2 | 124.3 | 124.9 | 144.3 | 29.5 | 51.4 | 57.6 | 82.7 | 112.6 | 257.5 |
| ID51493 | 366.1 | 335.9 | 152.8 | 185.0 | 137.5 | 95.9 | 51.3 | 25.6 | 35.8 | 51.0 | 65.7 | 113.6 |
| ID51501 | 104.6 | 132.9 | 104.9 | 75.9 | 132.7 | 48.7 | 113.0 | 245.0 | 119.6 | 18.9 | 39.3 | 791.4 |
| ID51516 | 142.6 | 109.9 | 81.5 | 119.2 | 116.0 | 132.2 | 33.9 | 40.4 | 61.6 | 77.3 | 110.5 | 362.2 |
| ID51517 | 171.8 | 113.4 | 328.9 | 424.8 | 87.4 | 86.5 | 96.3 | 195.7 | 59.8 | 95.4 | 87.8 | 28.4 |
| ID51518 | 253.1 | 235.3 | 123.8 | 193.1 | 89.7 | 82.1 | 20.6 | 24.7 | 22.4 | 44.5 | 68.4 | 149.7 |
| ID51525 | 82.3 | 62.9 | 127.0 | 203.8 | 117.0 | 149.8 | 148.1 | 245.9 | 104.3 | 156.3 | 59.7 | 10.2 |
| ID51533 | 263.0 | 199.9 | 147.9 | 144.2 | 370.6 | 130.0 | 129.9 | 128.7 | 364.8 | 105.4 | 150.8 | 126.0 |
| ID51539 | 105.3 | 93.7 | 122.1 | 103.8 | 109.6 | 87.6 | 102.4 | 125.6 | 109.3 | 113.7 | 116.8 | 94.4 |
| ID51543 | 179.7 | 187.2 | 109.9 | 191.6 | 132.3 | 133.6 | 121.8 | 188.4 | 107.4 | 150.4 | 68.1 | 61.5 |
| ID51544 | 84.5 | 75.1 | 83.4 | 77.3 | 96.8 | 91.4 | 90.2 | 120.5 | 98.4 | 104.5 | 103.9 | 105.0 |
| ID51548 | 43.4 | 80.6 | 101.2 | 80.3 | 183.9 | 119.7 | 159.8 | 212.3 | 125.4 | 138.0 | 137.4 | 120.8 |
| ID51550 | 268.4 | 267.3 | 132.4 | 211.2 | 107.5 | 84.4 | 45.3 | 38.4 | 30.2 | 56.6 | 58.0 | 127.8 |
| ID51551 | 132.3 | 154.7 | 432.5 | 322.4 | 179.8 | 108.1 | 293.9 | 755.5 | 199.1 | 255.4 | 679.7 | 84.3 |
| ID51552 | 54.8 | 50.5 | 90.0 | 70.8 | 106.4 | 108.0 | 118.0 | 186.5 | 119.8 | 149.8 | 107.8 | 118.3 |
| ID51556 | 203.5 | 164.5 | 107.0 | 161.9 | 101.0 | 97.8 | 34.2 | 33.5 | 40.3 | 65.4 | 80.8 | 215.3 |
| ID51557 | 131.2 | 144.3 | 214.2 | 97.7 | 407.0 | 171.7 | 201.7 | 349.0 | 150.4 | 356.4 | 158.6 | 129.0 |
| ID51558 | 159.5 | 256.3 | 197.1 | 193.6 | 120.5 | 44.5 | 147.9 | 721.2 | 494.3 | 206.7 | 163.5 | 195.8 |
| ID51559 | 66.5 | 51.7 | 80.4 | 71.5 | 90.1 | 103.5 | 112.3 | 175.4 | 139.7 | 154.3 | 99.8 | 119.4 |
| ID51560 | 88.2 | 45.5 | 87.0 | 68.8 | 101.8 | 86.3 | 103.2 | 194.9 | 170.3 | 180.0 | 99.3 | 134.6 |
| ID51561 | 150.0 | 179.7 | 255.6 | 250.6 | 57.7 | 51.1 | 80.2 | 117.1 | 96.5 | 69.9 | 108.4 | 56.2 |
| ID51562 | 112.6 | 103.9 | 74.9 | 109.6 | 124.0 | 142.9 | 38.4 | 50.6 | 54.2 | 81.8 | 112.2 | 319.9 |
| ID51566 | 89.2 | 88.0 | 67.8 | 80.4 | 117.8 | 122.8 | 66.1 | 90.3 | 71.4 | 86.3 | 122.4 | 202.1 |
| ID51573 | 159.6 | 103.0 | 99.6 | 80.5 | 98.0 | 81.5 | 133.9 | 108.6 | 111.4 | 25.0 | 66.6 | 258.3 |
| ID51577 | 131.2 | 202.9 | 348.2 | 256.2 | 219.5 | 74.1 | 235.3 | 425.7 | 181.6 | 57.6 | 273.2 | 73.1 |
| ID51583 | 213.3 | 186.4 | 135.9 | 179.8 | 95.3 | 66.5 | 74.9 | 175.8 | 111.7 | 98.4 | 222.2 | 182.1 |
| ID51585 | 180.4 | 153.1 | 118.8 | 168.1 | 90.5 | 79.7 | 47.1 | 45.5 | 59.6 | 64.3 | 78.7 | 108.2 |
| ID51586 | 88.6 | 86.8 | 81.3 | 93.4 | 92.9 | 90.4 | 88.8 | 82.6 | 75.6 | 60.8 | 85.7 | 104.0 |
| ID51587 | 174.6 | 167.4 | 157.7 | 151.3 | 117.9 | 75.2 | 86.2 | 166.6 | 78.9 | 62.2 | 81.9 | 90.0 |
| ID51588 | 183.6 | 168.7 | 123.9 | 173.1 | 89.5 | 80.8 | 43.7 | 43.2 | 51.9 | 58.9 | 76.5 | 112.7 |
| ID51589 | 116.8 | 56.3 | 80.9 | 64.0 | 89.0 | 73.4 | 85.9 | 87.9 | 73.1 | 73.3 | 87.7 | 111.5 |
| ID51590 | 178.8 | 142.8 | 161.5 | 159.8 | 98.4 | 61.9 | 75.2 | 150.8 | 79.7 | 80.9 | 74.3 | 81.5 |
| ID51591 | 287.3 | 152.5 | 192.7 | 194.5 | 175.9 | 87.6 | 102.4 | 157.1 | 79.6 | 130.8 | 136.7 | 95.7 |
| ID51592 | 93.4 | 130.9 | 131.7 | 101.3 | 72.7 | 96.7 | 81.9 | 108.8 | 94.6 | 68.2 | 86.1 | 131.1 |
| ID51593 | 83.6 | 87.5 | 84.3 | 88.8 | 95.2 | 91.3 | 80.4 | 116.7 | 73.3 | 84.4 | 91.1 | 116.8 |
| ID51594 | 88.8 | 77.4 | 66.1 | 90.8 | 87.6 | 105.8 | 69.2 | 88.2 | 83.9 | 88.0 | 77.7 | 100.6 |
| ID51598 | 73.9 | 86.1 | 100.0 | 90.5 | 95.4 | 84.9 | 97.0 | 109.5 | 107.9 | 115.9 | 93.1 | 92.6 |
| ID51599 | 218.0 | 153.3 | 161.5 | 157.6 | 144.9 | 71.7 | 77.7 | 191.9 | 76.3 | 95.9 | 84.4 | 82.8 |
| ID51600 | 84.1 | 81.4 | 51.3 | 73.2 | 88.0 | 100.8 | 76.8 | 76.9 | 62.8 | 56.2 | 72.9 | 123.6 |
| ID51601 | 90.8 | 79.6 | 52.9 | 77.7 | 84.3 | 90.2 | 72.1 | 67.9 | 61.3 | 51.7 | 79.6 | 148.1 |
| ID51604 | 136.4 | 116.8 | 81.2 | 117.4 | 104.4 | 112.1 | 46.2 | 50.8 | 55.5 | 70.9 | 101.0 | 228.6 |
| ID51605 | 101.8 | 134.9 | 115.2 | 106.0 | 79.0 | 79.7 | 88.2 | 77.2 | 62.0 | 56.8 | 68.0 | 93.9 |
| ID51610 | 89.1 | 111.4 | 100.0 | 75.3 | 59.6 | 52.9 | 70.0 | 95.6 | 76.5 | 40.9 | 82.6 | 120.2 |
| ID51611 | 52.5 | 53.5 | 50.7 | 57.1 | 70.0 | 55.5 | 77.7 | 107.5 | 136.2 | 90.3 | 122.4 | 188.6 |
| ID51613 | 163.6 | 139.5 | 95.1 | 141.3 | 106.0 | 94.8 | 43.2 | 42.9 | 45.3 | 61.0 | 91.5 | 167.7 |
| ID51617 | 92.4 | 78.1 | 118.4 | 98.3 | 111.6 | 101.4 | 99.0 | 101.6 | 80.9 | 92.5 | 103.3 | 88.2 |
| ID51618 | 70.2 | 62.7 | 67.5 | 80.4 | 27.1 | 52.4 | 71.0 | 69.1 | 79.7 | 21.3 | 62.3 | 143.2 |
| ID51619 | 148.8 | 129.3 | 91.2 | 128.2 | 96.8 | 79.0 | 54.0 | 41.4 | 77.7 | 82.2 | 85.9 | 127.1 |
| ID51620 | 131.4 | 79.7 | 101.5 | 127.1 | 87.3 | 64.1 | 86.7 | 76.5 | 61.6 | 57.2 | 47.2 | 92.5 |
| ID51621 | 94.4 | 105.1 | 130.4 | 109.6 | 74.7 | 90.4 | 85.6 | 103.3 | 88.7 | 75.0 | 96.3 | 107.5 |
| ID51624 | 82.2 | 114.5 | 116.1 | 98.7 | 90.2 | 84.2 | 93.6 | 104.3 | 96.9 | 74.7 | 92.0 | 95.9 |
| ID51626 | 87.5 | 78.3 | 60.3 | 92.0 | 86.7 | 104.1 | 74.0 | 79.6 | 68.2 | 63.4 | 88.1 | 147.1 |
| ID51629 | 98.1 | 77.8 | 46.2 | 74.9 | 87.7 | 109.2 | 65.4 | 66.6 | 62.0 | 61.7 | 71.8 | 165.5 |
| ID51632 | 89.5 | 93.4 | 111.0 | 108.9 | 78.9 | 72.4 | 80.8 | 92.0 | 84.9 | 66.7 | 84.6 | 90.8 |
| ID51633 | 91.7 | 93.5 | 129.3 | 102.7 | 81.2 | 77.6 | 91.3 | 95.7 | 78.5 | 71.5 | 98.4 | 93.8 |
| ID51635 | 81.5 | 80.7 | 45.4 | 87.0 | 82.1 | 125.0 | 72.9 | 104.7 | 66.1 | 79.2 | 78.1 | 127.6 |
| ID51637 | 75.1 | 78.2 | 36.4 | 69.6 | 73.2 | 95.9 | 75.4 | 74.5 | 59.6 | 56.0 | 76.5 | 112.4 |
| ID51644 | 108.4 | 74.7 | 93.9 | 83.3 | 116.9 | 109.6 | 88.4 | 82.1 | 74.9 | 78.1 | 93.7 | 70.9 |
| ID51645 | 95.9 | 110.6 | 126.4 | 102.3 | 81.8 | 70.6 | 86.5 | 92.1 | 89.7 | 69.0 | 90.0 | 88.6 |
| ID51650 | 149.5 | 134.0 | 178.2 | 191.0 | 47.0 | 50.3 | 63.5 | 85.8 | 69.6 | 70.5 | 50.8 | 48.6 |
| ID51651 | 83.5 | 79.7 | 74.8 | 93.1 | 92.0 | 95.3 | 78.2 | 101.8 | 73.6 | 99.0 | 90.4 | 114.1 |
| ID51653 | 89.4 | 139.1 | 100.9 | 124.2 | 67.0 | 57.6 | 68.2 | 76.1 | 76.7 | 53.3 | 57.3 | 96.1 |
| ID51655 | 74.6 | 87.6 | 70.2 | 144.2 | 68.1 | 57.6 | 64.4 | 32.3 | 90.9 | 37.0 | 39.7 | 132.2 |
| ID51656 | 64.5 | 74.5 | 78.5 | 70.0 | 85.0 | 104.4 | 81.4 | 119.3 | 96.1 | 157.2 | 80.3 | 110.9 |
| ID51661 | 73.9 | 81.7 | 69.4 | 70.3 | 92.1 | 113.5 | 76.2 | 105.7 | 93.7 | 137.2 | 79.9 | 108.5 |
| ID51662 | 66.3 | 77.2 | 78.2 | 67.1 | 86.6 | 99.3 | 81.5 | 125.1 | 112.0 | 160.0 | 80.5 | 107.4 |
| ID51664 | 108.8 | 155.4 | 136.3 | 155.2 | 79.8 | 67.4 | 63.4 | 81.5 | 61.7 | 77.7 | 77.3 | 68.0 |
| ID51665 | 74.3 | 79.8 | 71.1 | 73.6 | 100.6 | 101.9 | 81.6 | 104.1 | 81.6 | 119.4 | 81.8 | 113.5 |
| ID51666 | 80.1 | 89.5 | 73.9 | 75.2 | 96.8 | 104.2 | 82.5 | 126.3 | 75.8 | 125.8 | 82.4 | 114.5 |
| ID51670 | 80.7 | 85.4 | 77.2 | 79.7 | 109.2 | 111.7 | 91.8 | 125.5 | 69.8 | 120.3 | 92.9 | 113.1 |
| ID51679 | 159.5 | 153.6 | 135.9 | 145.7 | 99.4 | 85.8 | 57.0 | 64.2 | 54.1 | 68.6 | 80.4 | 82.0 |
| ID51680 | 178.8 | 145.5 | 115.8 | 134.9 | 107.9 | 86.8 | 49.5 | 52.1 | 46.1 | 66.4 | 88.7 | 123.7 |
| ID51681 | 82.1 | 89.1 | 85.5 | 81.1 | 101.1 | 98.3 | 83.8 | 119.3 | 79.3 | 115.4 | 88.4 | 103.1 |
| ID51686 | 62.0 | 125.7 | 140.3 | 93.2 | 95.9 | 180.0 | 99.0 | 106.8 | 85.8 | 166.9 | 132.7 | 129.0 |
| ID51687 | 129.5 | 121.1 | 110.5 | 117.3 | 103.2 | 90.6 | 54.3 | 69.3 | 58.1 | 79.0 | 95.4 | 136.8 |
| ID51688 | 86.5 | 121.2 | 160.8 | 235.4 | 128.3 | 109.2 | 116.0 | 163.7 | 95.2 | 91.3 | 207.7 | 160.1 |
| ID51689 | 122.0 | 117.9 | 122.1 | 121.0 | 103.3 | 92.6 | 68.0 | 86.0 | 77.5 | 87.1 | 105.1 | 124.7 |
| ID51690 | 121.5 | 140.8 | 115.7 | 106.0 | 128.1 | 90.4 | 80.7 | 100.7 | 77.7 | 89.7 | 113.0 | 95.0 |
| ID51691 | 121.9 | 134.6 | 122.1 | 106.1 | 117.3 | 89.5 | 93.6 | 100.5 | 78.1 | 91.1 | 100.2 | 86.3 |
| ID51693 | 94.7 | 100.2 | 104.0 | 97.4 | 100.6 | 93.8 | 79.6 | 107.9 | 108.8 | 109.5 | 110.5 | 90.5 |
| ID51694 | 72.9 | 92.5 | 95.1 | 114.8 | 124.8 | 114.0 | 123.2 | 139.4 | 49.1 | 83.8 | 128.4 | 78.1 |
| ID51695 | 99.1 | 104.3 | 87.8 | 126.9 | 114.3 | 110.7 | 125.9 | 130.0 | 63.1 | 72.4 | 141.4 | 219.0 |
| ID51696 | 73.6 | 99.1 | 93.2 | 96.6 | 138.1 | 101.8 | 124.3 | 141.0 | 60.5 | 79.2 | 103.9 | 96.9 |
| ID51697 | 87.2 | 101.4 | 78.4 | 101.6 | 109.8 | 110.3 | 80.0 | 101.1 | 63.4 | 86.6 | 92.2 | 100.7 |
| ID51698 | 80.2 | 91.1 | 79.1 | 105.2 | 125.1 | 121.0 | 79.4 | 133.7 | 60.3 | 71.3 | 121.5 | 134.6 |
| ID51699 | 106.5 | 105.1 | 125.1 | 115.8 | 110.1 | 88.1 | 80.1 | 104.4 | 98.0 | 97.2 | 95.8 | 88.4 |
| ID51700 | 109.0 | 76.1 | 79.5 | 89.4 | 119.9 | 103.5 | 74.5 | 123.0 | 82.8 | 119.5 | 139.3 | 126.1 |
| ID51702 | 101.1 | 113.0 | 108.9 | 104.0 | 121.9 | 90.7 | 88.3 | 104.8 | 87.4 | 90.3 | 94.9 | 97.8 |
| ID51703 | 96.8 | 116.2 | 109.7 | 96.8 | 116.9 | 98.9 | 85.8 | 107.3 | 88.5 | 98.2 | 91.2 | 98.0 |
| ID51704 | 93.1 | 114.3 | 111.8 | 95.8 | 117.5 | 97.0 | 96.7 | 112.2 | 92.6 | 98.0 | 93.3 | 98.0 |
| ID51705 | 92.6 | 114.6 | 106.9 | 97.4 | 134.3 | 104.5 | 109.6 | 118.3 | 69.7 | 88.8 | 108.6 | 91.6 |
| ID51709 | 90.6 | 113.0 | 104.2 | 92.6 | 122.3 | 88.7 | 99.2 | 120.1 | 94.4 | 90.6 | 99.5 | 100.4 |
| ID51710 | 89.3 | 114.8 | 109.9 | 92.7 | 125.7 | 95.3 | 95.6 | 112.1 | 83.6 | 98.7 | 115.4 | 100.1 |
| ID51711 | 110.8 | 112.9 | 151.8 | 99.3 | 139.4 | 110.8 | 141.2 | 129.6 | 63.9 | 119.7 | 113.6 | 89.8 |
| ID51712 | 103.5 | 122.4 | 139.2 | 95.0 | 133.2 | 112.0 | 103.5 | 127.5 | 68.9 | 106.1 | 95.5 | 102.8 |
| ID51716 | 63.1 | 118.1 | 437.7 | 116.3 | 120.6 | 137.3 | 239.2 | 118.9 | 67.0 | 118.0 | 136.0 | 93.4 |
| ID51717 | 61.1 | 139.2 | 167.1 | 92.7 | 110.4 | 81.9 | 99.4 | 90.7 | 89.2 | 109.7 | 96.4 | 57.7 |
| ID51719 | 106.3 | 58.5 | 80.7 | 83.2 | 108.5 | 97.8 | 77.2 | 100.7 | 113.2 | 141.1 | 152.7 | 131.4 |
| ID51720 | 127.3 | 192.0 | 144.1 | 159.0 | 79.9 | 71.6 | 65.0 | 72.6 | 60.9 | 74.7 | 74.1 | 67.2 |
| ID51721 | 85.0 | 40.1 | 69.1 | 47.6 | 101.1 | 102.6 | 82.4 | 120.1 | 125.9 | 176.8 | 127.4 | 125.1 |
| ID51722 | 105.8 | 37.0 | 78.5 | 52.8 | 79.6 | 80.2 | 71.2 | 115.4 | 185.5 | 189.4 | 115.0 | 135.0 |
| ID51723 | 76.9 | 78.4 | 75.5 | 69.0 | 143.9 | 127.2 | 119.8 | 129.7 | 71.5 | 137.3 | 121.2 | 113.1 |
| ID51724 | 130.7 | 82.8 | 48.3 | 66.3 | 120.8 | 103.1 | 55.8 | 113.7 | 122.4 | 156.3 | 104.1 | 131.6 |
| ID51727 | 68.9 | 58.9 | 67.4 | 54.2 | 122.1 | 99.6 | 96.9 | 130.1 | 89.6 | 129.5 | 127.8 | 126.4 |
| ID51728 | 123.1 | 69.3 | 54.1 | 85.6 | 97.7 | 82.9 | 56.7 | 99.0 | 136.2 | 139.2 | 158.7 | 141.5 |
| ID51729 | 120.1 | 55.1 | 42.1 | 51.7 | 109.1 | 95.8 | 59.0 | 126.2 | 179.3 | 183.8 | 86.9 | 138.7 |
| ID51736 | 131.5 | 130.1 | 104.9 | 100.5 | 116.0 | 76.1 | 71.9 | 88.3 | 71.4 | 75.2 | 91.8 | 95.5 |
| ID51737 | 100.4 | 103.6 | 90.2 | 89.9 | 109.6 | 107.1 | 85.9 | 98.3 | 92.5 | 105.3 | 95.7 | 101.0 |
| ID51738 | 81.1 | 88.7 | 78.6 | 74.8 | 141.8 | 124.9 | 88.3 | 111.4 | 75.5 | 129.8 | 126.7 | 110.0 |
| ID51739 | 105.4 | 79.7 | 30.3 | 85.5 | 82.4 | 139.9 | 47.9 | 90.6 | 96.5 | 173.1 | 85.1 | 104.5 |
| ID51741 | 98.7 | 149.7 | 118.9 | 108.7 | 126.2 | 95.0 | 90.5 | 107.7 | 68.1 | 66.7 | 100.1 | 81.7 |
| ID51743 | 252.4 | 176.9 | 207.4 | 176.4 | 62.1 | 62.2 | 40.5 | 68.5 | 45.5 | 59.4 | 60.5 | 65.7 |
| ID51746 | 157.8 | 265.0 | 162.0 | 114.1 | 84.6 | 61.9 | 73.2 | 141.9 | 17.5 | 71.5 | 109.4 | 38.2 |
| ID51747 | 90.4 | 114.8 | 101.6 | 85.7 | 133.8 | 106.2 | 85.3 | 105.0 | 91.0 | 101.2 | 117.1 | 104.1 |
| ID51748 | 106.4 | 126.3 | 75.7 | 79.9 | 174.9 | 92.0 | 124.2 | 106.3 | 110.0 | 86.7 | 90.5 | 88.9 |
| ID51752 | 198.9 | 410.0 | 348.5 | 190.4 | 37.0 | 44.4 | 36.6 | 63.8 | 46.7 | 55.6 | 52.4 | 36.8 |
| ID51753 | 94.0 | 113.5 | 105.7 | 88.0 | 119.2 | 98.1 | 82.9 | 87.8 | 100.9 | 102.1 | 110.1 | 118.3 |
| ID51754 | 96.0 | 106.7 | 81.8 | 77.3 | 124.9 | 89.3 | 105.3 | 96.6 | 110.0 | 94.3 | 97.0 | 92.8 |
| ID51755 | 140.0 | 97.1 | 108.5 | 104.2 | 65.0 | 78.2 | 76.2 | 92.7 | 112.6 | 135.6 | 110.7 | 99.1 |
| ID51756 | 47.1 | 28.6 | 31.8 | 35.6 | 141.5 | 136.9 | 142.7 | 99.2 | 122.8 | 221.2 | 152.2 | 127.6 |
| ID51757 | 49.8 | 36.6 | 53.2 | 40.4 | 140.1 | 134.2 | 136.8 | 142.4 | 145.1 | 198.9 | 115.0 | 136.1 |
| ID51758 | 221.2 | 164.5 | 126.0 | 212.3 | 97.2 | 88.8 | 29.1 | 37.1 | 32.2 | 40.4 | 66.3 | 110.1 |
| ID51759 | 71.1 | 110.0 | 101.8 | 88.7 | 298.7 | 80.0 | 128.9 | 105.0 | 147.5 | 71.3 | 114.2 | 109.6 |
| ID51760 | 82.2 | 120.8 | 121.3 | 88.8 | 130.9 | 97.4 | 106.2 | 141.1 | 55.0 | 46.7 | 85.8 | 49.6 |
| ID51761 | 91.7 | 95.8 | 87.9 | 115.4 | 146.7 | 123.1 | 41.3 | 52.6 | 64.9 | 67.0 | 109.6 | 269.3 |
| ID51763 | 147.6 | 118.6 | 108.6 | 164.7 | 110.7 | 100.4 | 34.6 | 52.8 | 41.8 | 57.5 | 84.8 | 196.2 |
| ID51764 | 122.6 | 53.0 | 78.2 | 67.3 | 45.2 | 133.2 | 39.8 | 45.9 | 41.8 | 178.6 | 60.3 | 50.6 |
| ID51765 | 181.0 | 99.3 | 145.1 | 138.5 | 79.6 | 80.7 | 79.1 | 97.0 | 89.7 | 93.2 | 83.9 | 79.5 |
| ID51766 | 148.6 | 175.5 | 205.5 | 261.3 | 102.4 | 28.7 | 24.8 | 38.0 | 6.1 | 55.3 | 223.1 | 80.3 |
| ID51769 | 104.8 | 127.8 | 108.3 | 99.2 | 93.9 | 104.8 | 90.7 | 100.0 | 110.9 | 122.0 | 89.2 | 122.6 |
| ID51771 | 173.0 | 71.2 | 87.9 | 88.7 | 72.8 | 83.3 | 83.1 | 98.3 | 112.3 | 114.6 | 89.4 | 95.8 |
| ID51772 | 145.4 | 123.5 | 110.5 | 105.6 | 76.4 | 106.9 | 75.9 | 96.2 | 132.9 | 116.7 | 78.1 | 100.9 |
| ID51773 | 88.7 | 102.0 | 82.9 | 78.2 | 119.6 | 98.7 | 83.2 | 98.0 | 141.9 | 129.7 | 110.0 | 111.5 |
| ID51775 | 143.0 | 71.4 | 144.5 | 110.4 | 84.2 | 86.7 | 78.9 | 110.0 | 135.2 | 199.6 | 67.7 | 92.8 |
| ID51779 | 181.4 | 129.3 | 136.7 | 157.1 | 67.8 | 60.3 | 37.5 | 64.5 | 79.9 | 55.6 | 79.9 | 96.1 |
| ID51781 | 99.1 | 212.0 | 175.9 | 130.8 | 52.4 | 73.9 | 96.2 | 98.9 | 99.6 | 82.9 | 77.1 | 24.3 |
| ID51782 | 120.4 | 142.7 | 97.5 | 106.3 | 97.7 | 74.1 | 67.1 | 87.3 | 139.7 | 134.3 | 85.1 | 98.9 |
| ID51784 | 123.8 | 96.0 | 77.8 | 68.1 | 84.8 | 98.3 | 72.1 | 113.8 | 256.9 | 160.2 | 85.3 | 115.9 |
| ID51785 | 104.8 | 47.5 | 31.0 | 22.7 | 54.6 | 81.9 | 46.6 | 11.9 | 61.9 | 133.6 | 70.6 | 29.3 |
| ID51786 | 60.6 | 88.5 | 143.8 | 101.6 | 122.9 | 83.9 | 104.9 | 116.6 | 41.5 | 78.7 | 127.4 | 64.6 |
| ID51787 | 66.9 | 62.9 | 88.6 | 73.8 | 116.7 | 102.2 | 104.3 | 132.6 | 97.9 | 135.1 | 124.3 | 92.4 |
| ID51788 | 73.3 | 70.5 | 114.8 | 71.7 | 283.9 | 78.0 | 100.1 | 136.2 | 74.7 | 121.9 | 132.6 | 96.2 |
| ID51789 | 60.4 | 57.9 | 101.3 | 113.1 | 325.6 | 157.4 | 20.9 | 113.2 | 67.0 | 86.8 | 104.2 | 472.6 |
| ID51791 | 79.3 | 85.0 | 117.9 | 87.7 | 114.9 | 94.0 | 96.6 | 121.7 | 92.6 | 105.5 | 117.9 | 81.5 |
| ID51792 | 71.0 | 98.2 | 94.7 | 76.0 | 118.2 | 93.9 | 90.2 | 103.9 | 60.8 | 110.0 | 134.3 | 75.9 |
| ID51793 | 89.3 | 84.3 | 93.2 | 79.5 | 110.4 | 106.3 | 88.3 | 117.1 | 91.6 | 130.9 | 120.0 | 82.1 |
| ID51795 | 77.3 | 63.9 | 82.1 | 64.1 | 129.4 | 104.2 | 105.7 | 108.0 | 89.2 | 100.9 | 123.6 | 99.4 |
| ID51796 | 71.1 | 69.3 | 86.7 | 77.8 | 104.8 | 81.9 | 85.7 | 113.7 | 87.0 | 99.5 | 107.3 | 77.9 |
| ID51799 | 219.1 | 154.1 | 79.6 | 107.8 | 132.9 | 88.1 | 24.1 | 24.2 | 67.4 | 54.5 | 108.6 | 298.2 |
| ID51800 | 12.3 | 15.3 | 44.7 | 82.7 | 163.5 | 133.8 | 106.2 | 116.0 | 173.6 | 143.9 | 124.1 | 157.7 |
| ID51801 | 68.4 | 60.3 | 85.5 | 71.7 | 134.8 | 122.1 | 116.4 | 116.8 | 112.5 | 102.0 | 114.2 | 105.5 |
| ID51802 | 57.0 | 63.3 | 75.1 | 60.7 | 126.6 | 91.0 | 101.0 | 135.0 | 69.2 | 123.0 | 128.3 | 94.1 |
| ID51803 | 66.2 | 117.6 | 96.4 | 77.1 | 110.2 | 74.7 | 90.6 | 96.4 | 60.0 | 80.2 | 126.3 | 78.0 |
| ID51804 | 69.5 | 92.8 | 99.1 | 83.7 | 119.0 | 86.3 | 87.8 | 107.9 | 74.8 | 102.9 | 122.3 | 77.7 |
| ID51805 | 61.2 | 86.4 | 109.5 | 92.1 | 130.5 | 109.4 | 89.1 | 119.7 | 86.2 | 130.9 | 144.9 | 78.4 |
| ID51806 | 45.6 | 71.4 | 68.1 | 54.9 | 144.5 | 105.8 | 73.1 | 82.0 | 52.7 | 116.5 | 115.1 | 66.4 |
| ID51807 | 594.4 | 389.6 | 75.9 | 85.3 | 133.6 | 68.1 | 15.3 | 140.8 | 116.0 | 335.1 | 135.7 | 107.8 |
| ID51808 | 62.0 | 39.8 | 73.9 | 52.1 | 163.3 | 119.8 | 135.1 | 130.1 | 131.5 | 102.7 | 122.3 | 117.0 |
| ID51809 | 73.6 | 71.2 | 72.8 | 45.1 | 137.3 | 77.4 | 89.4 | 109.1 | 55.3 | 90.7 | 102.4 | 76.8 |
| ID51810 | 355.3 | 275.2 | 102.8 | 83.4 | 70.1 | 64.6 | 49.9 | 56.6 | 114.8 | 92.4 | 117.6 | 208.1 |
| ID51811 | 60.1 | 72.4 | 81.6 | 59.3 | 146.6 | 97.4 | 94.0 | 125.6 | 90.8 | 121.0 | 130.3 | 86.7 |
| ID51813 | 92.2 | 53.0 | 86.0 | 31.1 | 127.0 | 72.6 | 56.7 | 36.7 | 29.2 | 100.8 | 84.9 | 65.8 |
| ID51815 | 56.0 | 78.7 | 83.8 | 53.1 | 153.2 | 91.7 | 90.7 | 120.5 | 72.8 | 111.7 | 138.6 | 87.3 |
| ID51816 | 126.7 | 122.2 | 124.6 | 131.4 | 103.4 | 62.6 | 84.1 | 98.1 | 70.2 | 83.3 | 88.4 | 49.1 |
| ID51817 | 35.1 | 56.7 | 65.8 | 38.4 | 213.2 | 143.0 | 129.6 | 138.5 | 34.9 | 142.9 | 126.7 | 95.3 |
| ID51818 | 53.0 | 57.3 | 89.1 | 77.8 | 109.8 | 82.5 | 98.2 | 125.9 | 90.0 | 105.8 | 106.1 | 87.1 |
| ID51819 | 73.0 | 61.9 | 92.4 | 70.9 | 123.5 | 92.0 | 74.3 | 120.4 | 99.5 | 142.9 | 118.0 | 88.3 |
| ID51821 | 88.5 | 62.9 | 125.7 | 79.5 | 123.6 | 76.1 | 51.5 | 108.7 | 138.3 | 107.6 | 102.4 | 85.7 |
| ID51823 | 84.3 | 100.4 | 110.5 | 111.0 | 101.7 | 86.1 | 84.5 | 102.9 | 83.1 | 102.5 | 100.6 | 80.2 |
| ID51824 | 84.1 | 92.2 | 98.6 | 62.4 | 94.0 | 69.3 | 95.1 | 83.8 | 93.1 | 110.4 | 105.2 | 92.6 |
| ID51825 | 68.4 | 69.8 | 108.1 | 89.8 | 97.9 | 83.1 | 94.5 | 118.5 | 102.0 | 115.6 | 102.0 | 89.8 |
| ID51827 | 70.5 | 69.3 | 122.3 | 115.9 | 84.4 | 78.7 | 81.7 | 112.6 | 118.6 | 119.7 | 109.2 | 87.6 |
| ID51830 | 70.1 | 74.8 | 114.3 | 96.9 | 99.3 | 95.3 | 104.7 | 108.4 | 115.3 | 103.5 | 107.2 | 90.4 |
| ID51831 | 64.5 | 68.1 | 103.3 | 86.5 | 97.1 | 87.6 | 106.6 | 101.6 | 110.8 | 98.0 | 104.2 | 91.2 |
| ID51832 | 76.6 | 67.8 | 90.8 | 83.0 | 98.2 | 96.5 | 90.8 | 111.8 | 101.1 | 103.7 | 104.1 | 84.8 |
| ID51833 | 72.1 | 87.5 | 102.0 | 92.4 | 130.7 | 109.7 | 100.6 | 124.7 | 85.7 | 103.7 | 124.2 | 79.5 |
| ID51835 | 105.1 | 135.6 | 128.1 | 120.8 | 74.2 | 58.7 | 53.8 | 67.7 | 45.4 | 70.0 | 85.8 | 56.8 |
| ID51836 | 52.1 | 62.2 | 93.0 | 70.4 | 129.6 | 110.6 | 108.0 | 142.1 | 95.2 | 147.6 | 111.4 | 101.1 |
| ID51837 | 68.0 | 124.3 | 96.5 | 114.0 | 44.6 | 90.6 | 40.3 | 54.5 | 41.0 | 55.0 | 59.3 | 34.0 |
| ID51838 | 62.1 | 57.5 | 98.6 | 83.6 | 116.1 | 96.3 | 109.0 | 133.7 | 93.8 | 122.0 | 101.0 | 89.9 |
| ID51839 | 64.8 | 73.7 | 89.0 | 63.9 | 132.3 | 110.6 | 91.8 | 120.0 | 86.7 | 114.6 | 127.1 | 84.8 |
| ID51843 | 18.6 | 27.1 | 61.8 | 51.4 | 136.6 | 76.2 | 110.6 | 128.1 | 84.8 | 94.5 | 100.2 | 83.5 |
| ID51844 | 55.1 | 73.2 | 65.2 | 69.1 | 95.3 | 92.0 | 83.2 | 140.0 | 101.8 | 126.3 | 98.7 | 103.5 |
| ID51845 | 59.6 | 72.1 | 69.3 | 71.2 | 76.8 | 65.1 | 82.5 | 113.1 | 124.8 | 109.8 | 96.5 | 81.0 |
| ID539 | 54.1 | 66.6 | 69.2 | 69.3 | 85.8 | 73.2 | 89.9 | 126.7 | 135.9 | 104.6 | 104.9 | 97.3 |
| ID51847 | 44.1 | 61.2 | 72.2 | 54.7 | 122.0 | 87.2 | 115.8 | 138.2 | 60.9 | 94.8 | 107.4 | 85.6 |
| ID51848 | 63.1 | 77.2 | 96.5 | 73.3 | 103.6 | 78.9 | 95.2 | 123.5 | 87.0 | 117.5 | 111.9 | 90.2 |
| ID51849 | 14.4 | 6.5 | 51.9 | 21.1 | 172.7 | 90.7 | 128.8 | 159.0 | 68.0 | 112.7 | 118.5 | 79.6 |
| ID51851 | 92.7 | 95.4 | 117.7 | 93.3 | 129.0 | 106.7 | 96.2 | 109.9 | 77.2 | 102.1 | 126.9 | 91.2 |
| ID51853 | 33.5 | 56.8 | 48.2 | 45.3 | 123.3 | 69.4 | 84.2 | 129.8 | 56.9 | 66.6 | 94.0 | 82.4 |
| ID51854 | 82.9 | 77.5 | 91.6 | 83.2 | 105.6 | 104.0 | 95.6 | 105.6 | 106.4 | 103.3 | 102.3 | 95.7 |
| ID51855 | 88.1 | 92.0 | 124.7 | 103.4 | 105.4 | 97.2 | 93.6 | 113.3 | 77.6 | 105.1 | 109.1 | 77.2 |
| ID51856 | 55.4 | 46.4 | 61.2 | 60.5 | 87.4 | 75.5 | 81.1 | 121.2 | 105.7 | 125.2 | 98.6 | 87.4 |
| ID51857 | 27.6 | 42.8 | 59.1 | 49.6 | 133.7 | 90.0 | 117.2 | 137.4 | 44.4 | 94.6 | 99.2 | 76.5 |
| ID51858 | 42.6 | 64.4 | 64.6 | 52.7 | 112.8 | 108.3 | 86.7 | 119.6 | 66.3 | 119.9 | 104.3 | 85.8 |
| ID51860 | 46.5 | 69.4 | 74.3 | 59.0 | 103.9 | 86.4 | 97.9 | 126.6 | 67.7 | 104.5 | 102.7 | 90.9 |
| ID51865 | 71.4 | 77.5 | 85.4 | 75.0 | 132.7 | 114.3 | 105.0 | 112.6 | 78.8 | 88.7 | 123.5 | 94.6 |
| ID51866 | 163.4 | 141.7 | 182.3 | 143.0 | 77.7 | 88.8 | 65.6 | 79.3 | 83.7 | 80.5 | 100.2 | 59.5 |
| ID51867 | 60.6 | 84.4 | 97.8 | 58.8 | 154.6 | 110.1 | 116.8 | 115.2 | 83.2 | 94.9 | 136.4 | 90.1 |
| ID51869 | 70.9 | 72.8 | 114.0 | 93.8 | 94.9 | 80.2 | 94.9 | 125.8 | 105.0 | 135.3 | 113.3 | 93.2 |
| ID51877 | 127.1 | 180.5 | 161.1 | 203.3 | 60.7 | 71.6 | 60.3 | 90.5 | 105.3 | 109.6 | 87.5 | 49.3 |
| ID51878 | 92.2 | 76.1 | 118.9 | 91.4 | 98.1 | 78.2 | 82.3 | 103.7 | 116.5 | 131.3 | 103.8 | 80.8 |
| ID51880 | 85.3 | 106.2 | 101.8 | 106.7 | 49.8 | 67.7 | 48.0 | 51.7 | 55.7 | 47.6 | 53.2 | 48.4 |
| ID51881 | 63.8 | 75.3 | 66.5 | 47.7 | 101.9 | 60.2 | 79.5 | 98.2 | 77.2 | 100.7 | 92.4 | 91.4 |
| ID51883 | 185.1 | 197.0 | 172.1 | 115.1 | 98.0 | 66.3 | 74.0 | 80.5 | 54.4 | 85.2 | 88.8 | 57.1 |
| ID51886 | 79.5 | 46.7 | 72.1 | 64.0 | 79.3 | 69.6 | 84.3 | 128.1 | 134.3 | 166.3 | 70.3 | 75.8 |
| ID51892 | 88.6 | 92.6 | 110.3 | 84.8 | 110.0 | 75.1 | 97.0 | 90.6 | 56.4 | 105.2 | 111.1 | 68.5 |
| ID51893 | 103.0 | 77.7 | 100.7 | 93.4 | 93.1 | 98.8 | 90.0 | 99.9 | 115.9 | 110.0 | 93.5 | 93.0 |
| ID51894 | 112.0 | 60.1 | 100.5 | 88.6 | 76.6 | 77.0 | 93.4 | 123.2 | 126.7 | 154.1 | 91.8 | 87.9 |
| ID51895 | 99.6 | 92.9 | 109.9 | 102.7 | 94.4 | 101.6 | 90.6 | 95.4 | 109.0 | 111.3 | 95.5 | 86.5 |
| ID51898 | 79.2 | 76.7 | 109.5 | 99.1 | 95.2 | 89.4 | 94.3 | 111.0 | 106.3 | 97.4 | 91.7 | 70.4 |
| ID51899 | 102.7 | 114.5 | 133.4 | 75.7 | 125.8 | 75.6 | 71.3 | 66.6 | 54.1 | 90.6 | 101.7 | 54.6 |
| ID51903 | 81.1 | 89.7 | 110.7 | 89.8 | 119.8 | 110.9 | 96.9 | 97.8 | 91.6 | 127.9 | 99.0 | 72.4 |
| ID51904 | 67.0 | 73.6 | 100.8 | 72.4 | 108.0 | 94.5 | 104.3 | 125.9 | 121.1 | 143.2 | 140.0 | 115.6 |
| ID51905 | 69.5 | 110.4 | 110.7 | 79.3 | 137.2 | 86.6 | 98.1 | 98.5 | 65.5 | 92.9 | 95.7 | 82.2 |
| ID51907 | 119.9 | 112.5 | 165.3 | 140.9 | 109.7 | 79.7 | 90.8 | 106.8 | 82.8 | 95.5 | 96.2 | 60.0 |
| ID51908 | 75.0 | 81.8 | 125.3 | 82.7 | 121.1 | 82.6 | 118.5 | 114.2 | 90.7 | 108.1 | 123.4 | 92.7 |
| ID51911 | 57.5 | 73.8 | 88.9 | 73.9 | 167.6 | 92.4 | 102.6 | 97.8 | 64.0 | 107.9 | 76.8 | 78.8 |
| ID51917 | 70.3 | 91.9 | 162.7 | 91.2 | 120.7 | 88.8 | 103.5 | 110.6 | 78.6 | 104.6 | 121.1 | 80.8 |
| ID51919 | 88.4 | 118.7 | 157.8 | 106.2 | 110.2 | 98.9 | 104.5 | 112.7 | 88.6 | 75.1 | 105.4 | 86.1 |
| ID51920 | 84.4 | 122.5 | 111.4 | 90.9 | 92.3 | 74.6 | 139.6 | 120.1 | 126.3 | 59.8 | 144.9 | 85.8 |
| ID51922 | 92.3 | 86.7 | 106.5 | 96.8 | 116.8 | 86.2 | 107.0 | 111.5 | 117.7 | 117.1 | 119.7 | 92.8 |
| ID51924 | 70.2 | 96.2 | 110.2 | 94.2 | 90.5 | 78.4 | 120.4 | 121.6 | 101.4 | 98.3 | 123.9 | 84.3 |
| ID51925 | 78.1 | 132.7 | 119.7 | 96.8 | 107.2 | 80.3 | 120.3 | 129.6 | 101.4 | 82.8 | 127.7 | 86.7 |
| ID51926 | 60.0 | 97.6 | 98.5 | 79.7 | 128.5 | 87.4 | 135.6 | 149.6 | 79.0 | 85.3 | 139.4 | 98.9 |
| ID51927 | 99.6 | 85.8 | 101.2 | 83.6 | 80.7 | 70.8 | 78.9 | 73.8 | 102.4 | 95.5 | 82.0 | 88.2 |
| ID51928 | 89.6 | 95.7 | 114.4 | 98.6 | 112.5 | 102.8 | 113.8 | 113.2 | 101.4 | 83.6 | 111.3 | 89.8 |
| ID51929 | 71.5 | 97.1 | 108.8 | 87.4 | 119.5 | 94.0 | 134.3 | 150.4 | 83.0 | 93.9 | 141.4 | 109.5 |
| ID51930 | 104.2 | 128.5 | 192.1 | 167.6 | 120.4 | 59.9 | 167.3 | 85.7 | 168.1 | 56.1 | 96.2 | 58.9 |
| ID51931 | 96.8 | 78.3 | 109.3 | 99.7 | 98.4 | 85.3 | 93.1 | 87.0 | 121.2 | 107.4 | 113.4 | 97.6 |
| ID51932 | 101.9 | 116.4 | 118.0 | 104.5 | 101.0 | 73.7 | 112.1 | 133.7 | 91.8 | 80.5 | 109.9 | 96.8 |
| ID51933 | 112.4 | 90.5 | 105.7 | 98.8 | 95.3 | 97.2 | 86.8 | 99.7 | 107.6 | 99.6 | 93.7 | 93.4 |
| ID51934 | 77.8 | 147.3 | 151.5 | 102.7 | 107.8 | 95.1 | 133.8 | 134.0 | 91.9 | 97.8 | 143.5 | 94.3 |
| ID51938 | 91.0 | 90.6 | 108.1 | 103.2 | 111.8 | 88.4 | 109.5 | 132.8 | 97.3 | 109.6 | 137.1 | 117.5 |
| ID51940 | 102.2 | 94.8 | 94.2 | 82.7 | 95.6 | 70.5 | 74.3 | 84.6 | 88.3 | 102.1 | 102.4 | 94.8 |
| ID51941 | 80.9 | 138.9 | 153.1 | 116.7 | 100.9 | 95.1 | 125.5 | 136.2 | 94.5 | 94.4 | 143.5 | 103.2 |
| ID51944 | 88.7 | 96.2 | 81.2 | 75.3 | 118.4 | 79.9 | 91.4 | 93.4 | 67.0 | 85.2 | 82.3 | 81.5 |
| ID51945 | 158.7 | 119.0 | 169.8 | 151.8 | 77.2 | 75.0 | 76.0 | 74.0 | 93.3 | 78.9 | 77.9 | 65.1 |
| ID51946 | 83.3 | 64.4 | 115.4 | 94.0 | 87.0 | 63.3 | 115.3 | 148.1 | 118.1 | 94.3 | 110.6 | 102.9 |
| ID51950 | 115.1 | 92.0 | 124.7 | 113.9 | 114.2 | 94.1 | 101.2 | 102.2 | 88.3 | 95.7 | 88.0 | 78.2 |
| ID51951 | 105.6 | 266.5 | 233.9 | 115.4 | 161.8 | 122.4 | 257.9 | 134.0 | 70.7 | 48.0 | 176.4 | 75.8 |
| ID51952 | 83.9 | 114.8 | 97.5 | 70.5 | 140.2 | 100.8 | 103.4 | 115.5 | 72.5 | 75.3 | 117.9 | 93.5 |
| ID51953 | 105.9 | 69.1 | 95.2 | 100.4 | 97.6 | 93.1 | 106.5 | 133.6 | 143.2 | 131.5 | 122.8 | 124.3 |
| ID51955 | 78.4 | 76.3 | 85.6 | 70.7 | 117.8 | 91.3 | 100.6 | 112.7 | 90.7 | 98.7 | 116.6 | 98.1 |
| ID51957 | 106.9 | 99.6 | 122.0 | 122.9 | 166.7 | 70.5 | 110.3 | 138.7 | 261.4 | 77.3 | 234.3 | 116.3 |
| ID51958 | 102.2 | 102.1 | 100.1 | 95.3 | 145.5 | 120.4 | 99.7 | 101.9 | 108.7 | 92.2 | 132.0 | 95.8 |
| ID51959 | 88.7 | 87.4 | 98.2 | 86.9 | 108.9 | 94.4 | 94.5 | 103.3 | 87.2 | 93.0 | 103.4 | 92.8 |
| ID51960 | 82.7 | 75.0 | 85.9 | 74.3 | 111.7 | 88.4 | 101.1 | 112.5 | 95.4 | 97.3 | 107.4 | 101.1 |
| ID51961 | 114.0 | 106.6 | 113.6 | 109.1 | 96.2 | 99.5 | 85.8 | 102.2 | 111.5 | 104.6 | 92.9 | 93.9 |
| ID51962 | 128.9 | 124.1 | 149.3 | 145.8 | 167.2 | 134.8 | 138.8 | 115.8 | 139.2 | 69.5 | 197.3 | 95.6 |
| ID51963 | 113.2 | 107.7 | 111.9 | 104.9 | 145.8 | 104.2 | 110.0 | 108.2 | 118.3 | 91.8 | 120.6 | 101.4 |
| ID51964 | 113.8 | 116.2 | 118.2 | 111.3 | 102.8 | 99.5 | 81.1 | 95.6 | 115.4 | 112.0 | 95.8 | 98.2 |
| ID51967 | 96.0 | 52.4 | 86.9 | 91.3 | 89.9 | 85.6 | 101.0 | 127.5 | 109.8 | 120.0 | 113.1 | 123.5 |
| ID51970 | 97.9 | 37.4 | 87.6 | 94.2 | 96.0 | 88.8 | 83.4 | 120.2 | 93.3 | 126.4 | 106.1 | 110.0 |
| ID51972 | 40.8 | 31.5 | 41.3 | 41.9 | 136.6 | 100.2 | 155.7 | 146.2 | 150.7 | 117.5 | 141.0 | 147.0 |
| ID51974 | 149.1 | 130.8 | 114.1 | 109.3 | 100.7 | 84.6 | 66.1 | 64.3 | 102.6 | 112.1 | 89.3 | 86.9 |
| ID51980 | 109.6 | 98.7 | 95.1 | 89.3 | 134.0 | 88.3 | 83.3 | 82.4 | 115.8 | 137.1 | 118.3 | 128.4 |
| ID51981 | 65.5 | 41.0 | 40.9 | 50.6 | 141.4 | 97.7 | 151.6 | 134.1 | 146.6 | 118.1 | 147.4 | 158.1 |
| ID51985 | 71.1 | 57.8 | 123.7 | 91.7 | 161.6 | 123.2 | 145.6 | 191.5 | 148.8 | 196.9 | 113.2 | 139.9 |
| ID51990 | 161.1 | 115.3 | 98.3 | 108.2 | 101.5 | 70.4 | 61.5 | 55.6 | 97.9 | 115.9 | 88.1 | 91.5 |
| ID51991 | 111.7 | 83.5 | 93.1 | 95.4 | 113.4 | 87.3 | 126.2 | 90.9 | 49.8 | 102.3 | 95.5 | 81.7 |
| ID51993 | 55.3 | 40.3 | 44.6 | 52.3 | 143.1 | 131.8 | 141.3 | 125.8 | 137.8 | 137.7 | 144.6 | 139.9 |
| ID51997 | 62.8 | 61.0 | 76.5 | 61.7 | 197.2 | 166.9 | 178.9 | 201.3 | 158.0 | 188.0 | 166.5 | 180.8 |
| ID51999 | 48.0 | 38.4 | 34.8 | 42.1 | 140.9 | 115.7 | 135.4 | 123.5 | 144.8 | 135.6 | 148.4 | 148.1 |
| ID52003 | 113.0 | 67.1 | 81.5 | 83.2 | 135.8 | 91.9 | 92.2 | 96.9 | 141.0 | 163.9 | 130.8 | 136.7 |
| ID52004 | 89.7 | 65.8 | 113.9 | 82.0 | 103.2 | 121.1 | 102.8 | 132.0 | 142.9 | 192.4 | 126.3 | 124.7 |
| ID52008 | 49.4 | 45.2 | 91.6 | 82.7 | 79.8 |  | 107.6 | 134.7 | 91.2 | 123.1 | 90.8 | 93.2 |
| ID52009 | 82.5 | 72.3 | 121.4 | 102.7 | 118.8 | 132.0 | 125.3 | 147.4 | 89.3 | 144.1 | 113.5 | 86.1 |
| ID52012 | 37.0 | 61.1 | 66.3 | 43.5 | 163.7 | 226.7 | 173.5 | 171.5 | 101.3 | 227.6 | 221.8 | 131.4 |
| ID52015 | 100.3 | 114.7 | 136.4 | 91.3 | 134.8 | 130.2 | 130.5 | 145.2 | 108.0 | 158.3 | 128.0 | 115.1 |
| ID52019 | 156.4 | 190.1 | 188.1 | 148.5 | 93.2 | 95.9 | 68.4 | 84.6 | 85.8 | 94.7 | 94.1 | 68.1 |
| ID52020 | 117.5 | 153.9 | 190.3 | 112.8 | 139.4 | 86.0 | 135.4 | 222.4 | 109.0 | 115.5 | 144.9 | 162.0 |
| ID52021 | 101.1 | 127.8 | 98.5 | 72.3 | 108.1 | 90.8 | 89.8 | 95.5 | 92.5 | 90.5 | 100.4 | 86.2 |
| ID52022 | 61.8 | 74.6 | 90.7 | 67.0 | 191.5 | 126.5 | 116.9 | 105.4 | 119.0 | 146.7 | 165.6 | 121.5 |
| ID52023 | 33.5 | 45.5 | 53.9 | 39.8 | 116.0 | 127.9 | 129.3 | 139.1 | 130.1 | 177.3 | 181.3 | 135.8 |
| ID52024 | 114.1 | 151.7 | 202.8 | 194.8 | 66.4 | 247.9 | 185.1 | 102.3 | 157.7 | 110.8 | 60.8 | 146.1 |
| ID52026 | 100.8 | 69.9 | 115.9 | 84.3 | 124.7 | 83.5 | 74.2 | 105.8 | 92.6 | 91.7 | 95.0 | 89.0 |
| ID52027 | 103.0 | 107.5 | 92.0 | 71.5 | 226.1 | 138.8 | 127.8 | 143.6 | 81.8 | 112.8 | 138.7 | 103.5 |
| ID52028 | 119.9 | 37.1 | 75.0 | 73.2 | 95.5 | 78.5 | 97.1 | 132.7 | 160.1 | 169.4 | 77.8 | 106.6 |
| ID52029 | 152.7 | 162.5 | 180.1 | 160.2 | 87.5 | 61.0 | 72.8 | 107.7 | 80.1 | 73.6 | 97.1 | 100.1 |
| ID52031 | 87.1 | 57.1 | 85.5 | 74.6 | 116.0 | 94.6 | 111.4 | 130.9 | 108.3 | 128.4 | 99.0 | 102.2 |
| ID52033 | 220.2 | 202.3 | 232.9 | 235.7 | 54.3 | 168.6 | 85.0 | 131.8 | 159.2 | 228.3 | 92.0 | 90.8 |
| ID52035 | 64.3 | 41.7 | 76.1 | 58.3 | 140.2 | 132.8 | 100.5 | 135.0 | 111.5 | 147.6 | 125.5 | 98.4 |
| ID52042 | 178.2 | 173.6 | 216.5 | 206.9 | 92.0 | 107.2 | 75.9 | 107.7 | 80.9 | 95.6 | 74.2 | 58.0 |
| ID52043 | 107.4 | 79.0 | 100.7 | 88.1 | 103.5 | 104.0 | 94.4 | 110.5 | 134.7 | 146.2 | 124.9 | 111.9 |
| ID52045 | 1105.1 | 509.0 | 208.3 | 227.3 | 70.3 | 75.3 | 116.0 | 172.2 | 145.8 | 198.0 | 88.9 | 158.2 |
| ID52049 | 148.2 | 126.2 | 81.9 | 72.2 | 148.6 | 111.5 | 101.8 | 85.9 | 105.8 | 127.2 | 103.0 | 92.1 |
| ID52051 | 98.2 | 96.3 | 83.9 | 78.0 | 158.4 | 192.8 | 116.6 | 144.6 | 115.1 | 271.2 | 128.8 | 119.7 |
| ID52052 | 113.7 | 117.8 | 117.1 | 130.2 | 90.1 | 130.8 | 138.8 | 166.6 | 129.2 | 121.3 | 85.4 | 89.3 |
| ID52054 | 94.9 | 88.1 | 110.3 | 89.8 | 107.6 | 89.0 | 91.9 | 99.0 | 110.9 | 142.3 | 115.9 | 107.3 |
| ID52056 | 82.5 | 162.2 | 94.5 | 90.5 | 99.8 | 264.8 | 147.6 | 210.6 | 141.5 | 152.1 | 88.0 | 147.4 |
| ID52059 | 127.2 | 94.5 | 102.7 | 73.3 | 161.1 | 76.1 | 103.1 | 125.0 | 93.0 | 121.7 | 139.2 | 111.9 |
| ID52060 | 154.9 | 37.4 | 93.2 | 86.9 | 183.4 | 58.4 | 81.9 | 109.3 | 25.3 | 95.6 | 122.2 | 93.2 |
| ID52061 | 1027.9 | 459.5 | 285.8 | 196.1 | 87.5 | 124.3 | 69.8 | 33.3 | 74.5 | 79.7 | 69.7 | 133.7 |
| ID52064 | 105.6 | 102.0 | 121.5 | 105.2 | 99.7 | 87.9 | 93.3 | 115.3 | 92.2 | 137.7 | 98.8 | 86.6 |
| ID52065 | 154.0 | 88.8 | 365.8 | 105.8 | 169.2 | 68.9 | 507.1 | 114.3 | 78.7 | 104.2 | 125.8 | 96.8 |
| ID52066 | 818.3 | 555.1 | 115.6 | 142.3 | 103.3 | 121.7 | 86.1 | 261.5 | 150.0 | 285.8 | 74.6 | 122.4 |
| ID52069 | 82.4 | 118.2 | 121.5 | 83.6 | 115.3 | 105.4 | 102.9 | 119.5 | 83.7 | 113.8 | 124.1 | 97.7 |
| ID52072 | 86.1 | 112.4 | 116.2 | 80.2 | 121.4 | 122.8 | 104.3 | 114.8 | 86.9 | 131.7 | 118.4 | 94.0 |
| ID52073 | 110.2 | 317.6 | 214.7 | 199.9 | 149.9 | 178.7 | 240.3 | 180.5 | 237.2 | 253.4 | 185.4 | 174.0 |
| ID52074 | 123.0 | 132.7 | 103.3 | 89.2 | 105.5 | 98.7 | 87.9 | 98.7 | 79.9 | 110.3 | 106.7 | 90.0 |
| ID52076 | 78.0 | 113.9 | 115.3 | 86.5 | 105.5 | 103.7 | 101.2 | 116.1 | 91.1 | 115.2 | 116.9 | 92.3 |
| ID52077 | 78.7 | 109.9 | 111.4 | 87.2 | 111.3 | 122.8 | 101.5 | 123.5 | 90.6 | 147.1 | 114.9 | 92.9 |
| ID52081 | 126.6 | 81.6 | 115.2 | 101.8 | 91.6 | 72.4 | 85.9 | 111.4 | 103.3 | 145.4 | 79.6 | 77.1 |
| ID52084 | 881.3 | 681.2 | 237.9 | 75.5 | 180.6 | 117.4 | 164.6 | 131.4 | 73.6 | 377.8 | 23.3 | 18.6 |
| ID52085 | 90.9 | 86.8 | 108.2 | 101.6 | 90.7 | 76.5 | 93.1 | 99.3 | 72.1 | 97.2 | 90.2 | 88.1 |
| ID52089 | 114.5 | 89.8 | 147.6 | 150.5 | 66.8 | 99.2 | 119.2 | 167.6 | 138.4 | 111.1 | 85.3 | 83.1 |
| ID52091 | 97.3 | 89.2 | 91.3 | 86.3 | 94.8 | 94.8 | 95.5 | 96.0 | 104.0 | 92.3 | 95.7 | 94.2 |
| ID52093 | 37.0 | 24.3 | 38.8 | 40.8 | 93.7 | 130.2 | 133.9 | 173.6 | 114.7 | 85.7 | 128.1 | 211.9 |
| ID52094 | 142.7 | 91.1 | 80.1 | 105.3 | 97.4 | 69.0 | 92.2 | 110.3 | 154.0 | 166.9 | 83.4 | 119.0 |
| ID52095 | 144.8 | 110.4 | 120.4 | 105.2 | 140.3 | 85.6 | 93.9 | 97.6 | 67.0 | 153.5 | 108.1 | 81.3 |
| ID52096 | 41.9 | 33.9 | 37.6 | 42.6 | 97.9 | 135.5 | 121.4 | 161.0 | 115.8 | 88.1 | 118.5 | 194.0 |
| ID52097 | 427.5 | 229.4 | 238.5 | 235.6 | 42.9 | 46.9 | 54.4 | 82.9 | 58.1 | 53.7 | 48.9 | 92.8 |
| ID52098 | 406.4 | 131.4 | 215.1 | 236.8 | 25.7 | 49.5 | 100.7 | 141.8 | 85.7 | 131.0 | 52.5 | 101.4 |
| ID52099 | 100.3 | 84.9 | 72.6 | 71.8 | 97.6 | 119.7 | 99.8 | 141.8 | 126.7 | 96.3 | 99.5 | 160.2 |
| ID52100 | 146.5 | 94.3 | 68.5 | 79.1 | 54.6 | 79.6 | 91.7 | 162.9 | 85.8 | 92.9 | 99.9 | 252.9 |
| ID52101 | 264.9 | 310.8 | 120.0 | 89.5 | 86.2 | 73.6 | 66.7 | 77.3 | 121.7 | 65.5 | 75.2 | 126.6 |
| ID52102 | 2622.7 | 784.4 | 64.0 | 144.7 | 535.6 | 329.6 | 4.8 | 54.1 | 1.8 | 23.1 | 48.0 | 85.5 |
| ID52105 | 103.6 | 129.6 | 101.8 | 77.3 | 92.8 | 96.3 | 97.0 | 111.2 | 99.4 | 84.6 | 89.8 | 115.5 |
| ID52107 | 97.8 | 97.8 | 162.5 | 125.3 | 165.8 | 94.5 | 118.0 | 89.7 | 92.5 | 55.5 | 114.1 | 87.7 |
| ID52108 | 33.2 | 30.2 | 49.3 | 30.8 | 97.5 | 139.5 | 52.6 | 113.8 | 330.8 | 343.9 | 46.2 | 191.3 |
| ID52109 | 143.1 | 96.9 | 107.4 | 78.5 | 41.4 | 37.8 | 71.3 | 113.1 | 180.1 | 145.7 | 57.5 | 111.5 |
| ID52110 | 840.6 | 945.0 | 257.1 | 169.9 | 193.2 | 386.1 | 17.2 | 62.4 | 3.1 | 40.6 | 26.1 | 21.2 |
| ID52111 | 234.6 | 198.7 | 217.6 | 204.2 | 73.5 | 60.7 | 50.6 | 78.1 | 64.4 | 54.3 | 51.0 | 63.7 |
| ID52112 | 174.6 | 126.9 | 191.3 | 160.4 | 65.0 | 123.7 | 86.5 | 189.0 | 219.0 | 193.1 | 85.4 | 124.6 |
| ID52114 | 116.1 | 200.1 | 129.4 | 122.2 | 77.5 | 94.9 | 78.6 | 71.0 | 98.0 | 73.0 | 89.1 | 69.9 |
| ID52115 | 124.4 | 82.6 | 147.7 | 100.7 | 99.2 | 72.1 | 92.1 | 81.1 | 88.5 | 133.8 | 82.0 | 83.7 |
| ID52117 | 34.7 | 40.5 | 47.0 | 86.8 | 177.9 | 184.2 | 235.2 | 74.8 | 141.4 | 112.2 | 129.7 | 172.8 |
| ID52118 | 121.8 | 157.9 | 428.1 | 603.5 | 15.5 | 57.6 | 56.7 | 107.5 | 82.1 | 9.3 | 16.6 | 27.8 |
| ID52123 | 126.5 | 138.2 | 206.9 | 93.0 | 70.1 | 55.4 | 107.1 | 225.5 | 101.2 | 79.1 | 77.6 | 49.3 |
| ID52124 | 168.2 | 109.6 | 656.1 | 1294.0 | 34.6 | 31.1 | 84.7 | 119.3 | 134.4 | 15.0 | 51.2 | 103.5 |
| ID52126 | 108.6 | 81.0 | 103.4 | 92.4 | 86.2 | 77.9 | 79.1 | 83.2 | 130.5 | 100.9 | 66.2 | 107.1 |
| ID52127 | 534.5 | 953.9 | 1588.8 | 1119.9 | 6.5 | 5.9 | 4.4 | 43.1 | 18.0 | 4.0 | 15.0 | 11.9 |
| ID52129 | 92.1 | 70.6 | 94.9 | 73.2 | 150.3 | 121.1 | 122.9 | 109.3 | 117.6 | 177.8 | 86.8 | 140.5 |
| ID52130 | 92.3 | 60.7 | 109.5 | 86.3 | 100.9 | 92.0 | 106.7 | 106.5 | 116.3 | 146.0 | 81.9 | 126.5 |
| ID52131 | 88.8 | 60.1 | 77.9 | 68.8 | 134.3 | 111.2 | 108.2 | 103.2 | 103.8 | 164.5 | 84.1 | 151.8 |
| ID52132 | 497.6 | 372.0 | 651.7 | 445.9 | 22.8 | 92.5 | 6.0 | 67.5 | 12.2 | 3.6 | 8.9 | 10.4 |
| ID52134 | 92.4 | 77.7 | 89.3 | 87.2 | 93.7 | 105.8 | 110.2 | 98.3 | 108.5 | 89.9 | 86.1 | 96.5 |
| ID52135 | 52.7 | 69.4 | 313.4 | 259.6 | 138.7 | 54.0 | 244.6 | 181.5 | 130.6 | 84.0 | 277.0 | 189.4 |
| ID52137 | 205.6 | 221.9 | 248.8 | 281.0 | 49.8 | 39.5 | 33.2 | 62.5 | 25.7 | 31.9 | 33.1 | 20.7 |
| ID52138 | 214.0 | 101.7 | 223.5 | 125.0 | 66.4 | 75.3 | 83.4 | 82.4 | 153.8 | 187.2 | 65.1 | 109.8 |
| ID52141 | 171.7 | 138.4 | 98.4 | 144.1 | 45.1 | 95.7 | 53.9 | 84.5 | 135.2 | 223.0 | 75.1 | 79.4 |
| ID52142 | 159.6 | 143.9 | 121.9 | 220.1 | 217.6 | 140.7 | 107.8 | 117.4 | 120.7 | 164.1 | 302.4 | 100.4 |
| ID52145 | 89.5 | 85.2 | 80.9 | 74.6 | 91.5 | 86.5 | 96.7 | 102.9 | 87.1 | 89.5 | 84.7 | 101.1 |
| ID52146 | 210.7 | 214.8 | 259.0 | 223.1 | 76.6 | 58.1 | 74.3 | 63.9 | 89.5 | 71.1 | 58.1 | 54.9 |
| ID52147 | 102.7 | 64.0 | 85.2 | 94.0 | 96.1 | 90.0 | 112.3 | 104.6 | 121.7 | 108.9 | 91.1 | 101.8 |
| ID52148 | 88.2 | 53.0 | 60.7 | 48.1 | 109.0 | 88.8 | 96.7 | 126.1 | 96.0 | 91.3 | 50.8 | 115.7 |
| ID52151 | 194.9 | 174.9 | 173.2 | 201.4 | 90.7 | 170.2 | 73.1 | 125.3 | 129.8 | 183.3 | 171.6 | 99.3 |
| ID52152 | 71.8 | 99.5 | 66.5 | 103.3 | 113.6 | 98.8 | 112.6 | 109.5 | 85.6 | 83.1 | 72.7 | 95.3 |
| ID52154 | 169.1 | 203.8 | 161.7 | 478.1 | 83.3 | 132.0 | 109.4 | 50.5 | 53.1 | 41.1 | 61.0 | 77.9 |
| ID52155 | 130.2 | 54.8 | 114.0 | 144.0 | 61.0 | 83.4 | 73.7 | 93.7 | 67.5 | 92.5 | 41.2 | 91.4 |
| ID52156 | 272.5 | 140.6 | 213.8 | 282.6 | 74.3 | 67.1 | 49.4 | 141.2 | 33.0 | 89.1 | 49.5 | 69.7 |
| ID52157 | 16.0 | 79.2 | 81.6 | 26.7 | 283.1 | 119.8 | 163.6 | 224.2 | 343.1 | 261.7 | 269.5 | 194.9 |
| ID52158 | 94.3 | 68.7 | 83.4 | 90.1 | 78.9 | 80.3 | 110.9 | 118.3 | 149.3 | 124.9 | 91.4 | 106.2 |
| ID52160 | 129.5 | 113.0 | 133.4 | 130.5 | 87.4 | 81.9 | 87.4 | 110.1 | 153.0 | 105.6 | 80.7 | 91.0 |
| ID52161 | 48.6 | 55.4 | 52.3 | 119.3 | 90.0 | 114.3 | 81.8 | 85.3 | 57.5 | 115.3 | 83.4 | 107.9 |
| ID52162 | 73.2 | 25.6 | 102.4 | 116.5 | 98.8 | 93.1 | 134.1 | 129.4 | 115.8 | 124.7 | 74.8 | 139.5 |
| ID52163 | 75.7 | 51.9 | 53.3 | 116.3 | 55.1 | 91.2 | 58.5 | 71.8 | 65.6 | 94.2 | 62.3 | 94.0 |
| ID52165 | 95.6 | 81.2 | 118.8 | 99.6 | 157.1 | 91.7 | 120.3 | 147.6 | 143.6 | 126.7 | 120.7 | 114.4 |
| ID52166 | 6.9 | 142.2 | 76.0 | 38.2 | 45.7 | 66.8 | 79.1 | 63.6 | 173.0 | 90.7 | 81.8 | 86.5 |
| ID52169 | 100.3 | 92.3 | 88.4 | 134.6 | 86.9 | 121.2 | 130.7 | 81.0 | 87.3 | 67.1 | 65.8 | 103.3 |
| ID52170 | 123.0 | 73.3 | 128.5 | 136.2 | 77.8 | 84.8 | 102.7 | 99.1 | 103.4 | 100.7 | 99.0 | 94.7 |
| ID66139 | 58.1 | 60.2 | 63.3 | 85.0 | 71.4 | 112.1 | 83.3 | 112.0 | 123.5 | 146.1 | 115.4 | 123.5 |
| ID52174 | 62.7 | 78.0 | 50.4 | 186.5 | 921.2 | 172.5 | 37.8 | 88.6 | 249.6 | 189.2 | 130.3 | 228.9 |
| ID52176 | 125.9 | 283.6 | 76.7 | 165.9 | 844.0 | 137.5 | 27.9 | 114.4 | 264.2 | 138.7 | 94.2 | 255.0 |
| ID52179 | 66.1 | 122.1 | 84.7 | 63.1 | 158.8 | 92.6 | 114.3 | 121.1 | 48.2 | 59.2 | 110.2 | 86.2 |
| ID52180 | 104.3 | 104.1 | 99.9 | 138.1 | 97.5 | 117.0 | 139.0 | 97.9 | 87.9 | 75.3 | 116.2 | 94.1 |
| ID52181 | 63.9 | 46.1 | 56.5 | 56.4 | 46.1 | 64.1 | 89.2 | 106.8 | 153.1 | 87.1 | 73.2 | 100.9 |
| ID52182 | 81.5 | 87.7 | 116.3 | 163.3 | 109.2 | 101.7 | 87.8 | 109.8 | 37.4 | 73.4 | 97.6 | 91.3 |
| ID52184 | 152.9 | 186.8 | 185.2 | 173.8 | 43.1 | 87.4 | 89.2 | 105.9 | 195.3 | 111.2 | 72.5 | 89.8 |
| ID52185 | 122.1 | 44.5 | 60.1 | 118.4 | 47.8 | 93.9 | 59.4 | 102.4 | 94.4 | 127.1 | 82.7 | 131.3 |
| ID52186 | 74.8 | 101.8 | 105.6 | 71.4 | 123.4 | 90.3 | 93.7 | 105.2 | 90.6 | 99.7 | 110.9 | 91.4 |
| ID52188 | 81.9 | 84.3 | 86.1 | 89.3 | 106.9 | 110.4 | 136.8 | 112.7 | 116.3 | 89.0 | 132.9 | 112.5 |
| ID52189 | 203.7 | 200.7 | 191.8 | 187.8 | 50.3 | 52.8 | 53.2 | 58.1 | 76.7 | 59.7 | 45.9 | 52.8 |
| ID52190 | 167.1 | 162.0 | 154.7 | 264.2 | 45.9 | 79.4 | 98.3 | 49.7 | 46.3 | 23.8 | 25.3 | 63.0 |
| ID52191 | 61.4 | 71.3 | 91.7 | 65.0 | 101.5 | 78.8 | 93.6 | 107.5 | 163.2 | 109.6 | 88.6 | 95.9 |
| ID52192 | 116.3 | 63.4 | 31.2 | 43.1 | 36.0 | 25.4 | 50.9 | 109.3 | 263.9 | 172.9 | 92.0 | 144.9 |
| ID52193 | 26.9 | 127.4 | 53.3 | 38.8 | 155.2 | 88.7 | 111.1 | 112.9 | 46.4 | 47.1 | 127.2 | 78.2 |
| ID52194 | 163.1 | 202.2 | 97.2 | 160.4 | 132.0 | 147.0 | 51.8 | 76.9 | 106.2 | 120.9 | 86.6 | 121.7 |
| ID52195 | 138.6 | 112.4 | 119.9 | 216.7 | 56.0 | 93.9 | 108.8 | 59.0 | 62.7 | 41.2 | 39.8 | 77.6 |
| ID52196 | 116.6 | 331.4 | 96.8 | 123.5 | 128.6 | 169.4 | 79.0 | 105.3 | 185.1 | 102.4 | 98.6 | 111.9 |
| ID52197 | 75.4 | 82.3 | 87.5 | 85.7 | 105.6 | 112.5 | 121.3 | 108.6 | 114.4 | 95.9 | 119.2 | 108.9 |
| ID52198 | 75.9 | 86.4 | 86.0 | 72.0 | 105.4 | 89.6 | 88.7 | 107.4 | 82.8 | 106.4 | 105.1 | 95.5 |
| ID52199 | 61.5 | 114.1 | 131.6 | 81.7 | 100.4 | 74.6 | 98.0 | 116.8 | 75.3 | 85.6 | 115.2 | 93.3 |
| ID52200 | 53.3 | 94.9 | 122.8 | 69.5 | 100.0 | 64.9 | 91.5 | 137.3 | 80.5 | 73.7 | 100.4 | 81.3 |
| ID52204 | 197.2 | 327.0 | 106.3 | 158.2 | 151.5 | 155.5 | 39.7 | 71.9 | 101.1 | 123.3 | 82.8 | 109.8 |
| ID52206 | 140.4 | 243.4 | 110.3 | 119.1 | 72.9 | 86.8 | 100.3 | 72.8 | 128.7 | 79.8 | 76.0 | 88.6 |
| ID52207 | 95.9 | 93.8 | 105.8 | 95.6 | 90.0 | 82.3 | 88.2 | 81.7 | 77.9 | 70.3 | 79.0 | 78.9 |
| ID52208 | 108.4 | 113.2 | 109.1 | 137.5 | 101.3 | 86.3 | 121.8 | 140.1 | 71.2 | 77.1 | 62.8 | 84.5 |
| ID52209 | 86.2 | 82.3 | 87.1 | 83.1 | 96.5 | 89.4 | 88.7 | 102.1 | 102.5 | 100.7 | 87.9 | 83.2 |
| ID52210 | 610.6 | 347.5 | 504.4 | 488.9 | 1.3 | 34.8 | 15.5 | 20.5 | 8.6 | 168.6 | 35.3 | 62.0 |
| ID52211 | 74.8 | 75.7 | 80.9 | 85.8 | 101.3 | 91.8 | 96.9 | 89.3 | 93.8 | 61.2 | 81.8 | 85.5 |
| ID52215 | 102.1 | 151.8 | 137.2 | 100.2 | 67.8 | 66.5 | 50.8 | 50.2 | 43.0 | 49.4 | 64.6 | 34.5 |
| ID52216 | 131.1 | 103.0 | 105.7 | 137.9 | 67.6 | 79.6 | 83.3 | 95.4 | 102.9 | 76.2 | 67.8 | 78.5 |
| ID52219 | 108.9 | 99.8 | 114.3 | 108.8 | 87.9 | 69.2 | 78.4 | 74.2 | 63.9 | 59.0 | 74.4 | 77.8 |
| ID52220 | 94.2 | 96.8 | 95.4 | 87.9 | 96.2 | 88.3 | 114.5 | 94.9 | 95.9 | 87.0 | 89.3 | 97.6 |
| ID52222 | 86.1 | 74.9 | 84.7 | 89.0 | 96.6 | 107.5 | 98.0 | 95.2 | 107.2 | 100.2 | 95.5 | 100.3 |
| ID52224 | 146.7 | 154.7 | 93.1 | 106.5 | 75.1 | 81.2 | 70.3 | 69.6 | 78.2 | 67.8 | 65.5 | 81.8 |
| ID52225 | 164.6 | 206.7 | 109.3 | 117.0 | 100.8 | 92.0 | 64.9 | 69.7 | 72.2 | 80.5 | 72.9 | 94.3 |
| ID52226 | 91.9 | 42.7 | 79.4 | 64.9 | 75.4 | 77.2 | 76.2 | 88.8 | 122.6 | 137.8 | 92.2 | 103.2 |
| ID52227 | 96.5 | 83.8 | 91.2 | 84.8 | 103.3 | 91.2 | 92.4 | 109.3 | 103.2 | 76.4 | 91.3 | 105.1 |
| ID52228 | 184.8 | 217.6 | 128.1 | 124.9 | 77.7 | 72.2 | 58.0 | 64.6 | 58.8 | 71.0 | 59.2 | 71.4 |
| ID52229 | 114.5 | 100.8 | 117.6 | 115.0 | 92.3 | 102.3 | 87.0 | 76.8 | 89.4 | 104.6 | 85.6 | 82.7 |
| ID52230 | 139.7 | 122.5 | 100.3 | 120.2 | 69.0 | 81.1 | 84.5 | 78.8 | 94.2 | 75.0 | 74.0 | 82.9 |
| ID52231 | 104.3 | 118.4 | 102.9 | 111.9 | 116.6 | 96.8 | 91.5 | 109.9 | 85.3 | 77.2 | 78.4 | 114.9 |
| ID52232 | 62.1 | 55.7 | 59.8 | 77.0 | 83.9 | 119.8 | 128.9 | 90.1 | 111.3 | 85.8 | 81.9 | 122.4 |
| ID52233 | 190.9 | 215.3 | 123.6 | 125.2 | 5.9 | 6.3 | 8.3 | 12.2 | 10.2 | 4.2 | 9.0 | 21.1 |
| ID52234 | 109.1 | 109.9 | 111.1 | 100.8 | 83.2 | 82.6 | 102.2 | 83.0 | 86.8 | 73.0 | 79.1 | 87.2 |
| ID52235 | 63.9 | 57.7 | 63.8 | 72.1 | 91.0 | 106.6 | 120.2 | 89.5 | 107.8 | 85.9 | 84.5 | 122.5 |
| ID52236 | 100.5 | 104.1 | 80.7 | 66.8 | 108.6 | 83.6 | 87.1 | 87.7 | 63.3 | 66.6 | 69.9 | 70.6 |
| ID52237 | 111.4 | 114.0 | 68.3 | 81.5 | 73.9 | 79.6 | 78.9 | 83.5 | 95.1 | 75.2 | 87.8 | 103.7 |
| ID52238 | 71.0 | 65.1 | 76.0 | 85.8 | 80.7 | 94.2 | 114.3 | 89.5 | 111.9 | 86.0 | 94.3 | 115.3 |
| ID52239 | 96.8 | 98.2 | 114.1 | 113.8 | 86.9 | 97.5 | 110.0 | 89.2 | 87.7 | 70.3 | 81.0 | 86.0 |
| ID52241 | 87.2 | 93.7 | 74.7 | 66.1 | 122.2 | 106.1 | 112.8 | 93.4 | 96.4 | 89.8 | 84.9 | 92.9 |
| ID52242 | 116.0 | 111.1 | 129.9 | 93.0 | 87.3 | 90.2 | 87.4 | 84.6 | 126.4 | 106.1 | 84.2 | 71.2 |
| ID52243 | 103.2 | 107.8 | 104.6 | 96.4 | 79.2 | 79.9 | 80.0 | 73.0 | 78.4 | 68.9 | 73.3 | 74.9 |
| ID52247 | 92.6 | 84.2 | 88.3 | 87.8 | 87.5 | 95.5 | 98.9 | 91.4 | 98.8 | 85.8 | 84.4 | 86.8 |
| ID52249 | 533.2 | 197.3 | 64.3 | 94.0 | 46.5 | 359.9 | 80.7 | 64.3 | 75.8 | 26.6 | 8.3 | 551.8 |
| ID52250 | 90.8 | 80.9 | 87.9 | 88.7 | 87.0 | 80.7 | 93.0 | 98.8 | 90.3 | 75.5 | 75.3 | 88.3 |
| ID52252 | 131.6 | 109.2 | 75.6 | 114.8 | 78.1 | 72.0 | 44.1 | 57.9 | 75.8 | 60.7 | 63.7 | 164.5 |
| ID52255 | 164.8 | 135.0 | 140.5 | 154.6 | 87.6 | 84.5 | 84.6 | 104.1 | 91.8 | 63.4 | 51.9 | 71.5 |
| ID52256 | 106.9 | 114.1 | 142.1 | 157.2 | 68.3 | 60.6 | 52.7 | 80.3 | 77.9 | 63.0 | 62.8 | 70.3 |
| ID52257 | 107.8 | 84.1 | 93.7 | 107.0 | 94.8 | 89.1 | 90.3 | 93.7 | 110.2 | 82.7 | 80.4 | 114.4 |
| ID52258 | 83.0 | 70.4 | 83.9 | 99.4 | 93.2 | 110.4 | 117.9 | 118.8 | 84.3 | 79.8 | 75.7 | 86.9 |
| ID52261 | 177.8 | 149.0 | 132.7 | 123.9 | 92.6 | 96.2 | 112.0 | 72.3 | 62.9 | 74.8 | 61.0 | 85.7 |
| ID52262 | 139.8 | 158.7 | 135.7 | 128.6 | 87.6 | 71.5 | 57.1 | 65.0 | 124.0 | 61.9 | 65.7 | 84.8 |
| ID52264 | 123.4 | 114.4 | 71.7 | 94.6 | 131.3 | 101.7 | 65.5 | 82.3 | 77.1 | 62.1 | 64.9 | 117.9 |
| ID52265 | 113.3 | 101.1 | 100.7 | 138.8 | 71.3 | 63.8 | 52.4 | 97.4 | 130.5 | 78.1 | 71.7 | 76.7 |
| ID52266 | 441.0 | 223.3 | 95.2 | 389.3 | 7.7 | 10.8 | 8.0 | 23.0 | 3.9 | 4.8 | 10.3 | 61.8 |
| ID52267 | 123.8 | 111.3 | 82.7 | 116.9 | 97.2 | 106.0 | 75.7 | 78.1 | 90.7 | 86.8 | 71.9 | 104.1 |
| ID52268 | 119.6 | 178.8 | 189.6 | 95.2 | 88.1 | 111.0 | 90.7 | 71.5 | 129.8 | 106.8 | 81.4 | 76.2 |
| ID52270 | 98.4 | 74.2 | 83.5 | 70.2 | 86.5 | 89.6 | 83.3 | 101.3 | 140.5 | 129.0 | 99.9 | 125.9 |
| ID52271 | 76.6 | 106.7 | 89.1 | 92.9 | 128.8 | 113.7 | 122.7 | 107.4 | 66.8 | 67.3 | 115.8 | 88.2 |
| ID52272 | 89.7 | 81.2 | 91.9 | 89.6 | 73.3 | 76.3 | 97.1 | 86.8 | 105.2 | 87.2 | 80.1 | 115.2 |
| ID52273 | 72.7 | 55.7 | 70.2 | 98.8 | 133.1 | 227.5 | 261.3 | 146.9 | 299.9 | 124.8 | 143.0 | 276.6 |
| ID52274 | 94.4 | 88.1 | 90.3 | 88.6 | 101.8 | 102.3 | 113.2 | 94.1 | 88.8 | 81.4 | 91.9 | 84.5 |
| ID52275 | 92.5 | 45.9 | 101.7 | 43.8 | 50.4 | 48.6 | 65.8 | 46.1 | 31.5 | 65.7 | 37.0 | 51.9 |
| ID52277 | 141.8 | 198.5 | 66.2 | 98.2 | 269.5 | 129.9 | 72.6 | 77.6 | 70.4 | 95.3 | 77.7 | 234.4 |
| ID52278 | 230.6 | 225.9 | 53.0 | 75.0 | 203.5 | 108.2 | 65.2 | 58.9 | 50.6 | 84.2 | 50.1 | 222.4 |
| ID52280 | 167.0 | 132.1 | 91.5 | 60.2 | 93.2 | 101.3 | 62.1 | 62.6 | 66.0 | 102.0 | 50.5 | 62.9 |
| ID52282 | 334.6 | 198.6 | 68.7 | 72.1 | 157.5 | 128.3 | 102.0 | 82.1 | 55.8 | 73.7 | 52.7 | 94.9 |
| ID52283 | 94.9 | 95.8 | 86.1 | 78.8 | 104.3 | 96.6 | 107.5 | 99.2 | 73.9 | 82.1 | 91.3 | 87.5 |
| ID52284 | 147.8 | 136.8 | 92.1 | 102.8 | 97.8 | 94.9 | 106.6 | 115.5 | 98.2 | 88.0 | 90.7 | 107.6 |
| ID52285 | 131.4 | 94.8 | 90.5 | 101.2 | 125.6 | 139.1 | 127.7 | 110.0 | 133.8 | 101.9 | 96.6 | 175.5 |
| ID52287 | 212.4 | 151.3 | 119.7 | 260.6 | 143.6 | 136.0 | 132.2 | 90.6 | 111.3 | 59.6 | 83.8 | 119.2 |
| ID52289 | 149.8 | 95.5 | 165.0 | 144.4 | 61.9 | 72.9 | 80.0 | 141.1 | 84.4 | 88.5 | 64.8 | 104.3 |
| ID52290 | 124.0 | 124.7 | 109.6 | 106.2 | 90.5 | 98.5 | 96.3 | 90.4 | 104.2 | 85.5 | 83.4 | 91.2 |
| ID52291 | 138.1 | 110.3 | 91.3 | 162.6 | 131.5 | 109.3 | 118.8 | 98.4 | 98.5 | 71.4 | 106.6 | 96.6 |
| ID52292 | 85.5 | 100.5 | 119.5 | 107.5 | 90.5 | 96.2 | 111.6 | 106.9 | 175.8 | 108.2 | 105.8 | 132.9 |
| ID52293 | 146.4 | 121.9 | 94.9 | 98.6 | 116.3 | 109.3 | 125.4 | 100.0 | 83.0 | 80.6 | 76.8 | 83.5 |
| ID52294 | 80.4 | 76.7 | 72.0 | 101.5 | 111.6 | 98.0 | 99.0 | 93.7 | 131.7 | 80.8 | 106.7 | 145.4 |
| ID52299 | 135.8 | 146.2 | 105.3 | 112.1 | 127.9 | 115.5 | 115.2 | 121.7 | 101.2 | 96.9 | 92.5 | 98.7 |
| ID52300 | 110.0 | 126.9 | 50.2 | 74.3 | 78.8 | 89.2 | 135.1 | 116.5 | 142.6 | 83.1 | 101.1 | 103.1 |
| ID52302 | 129.2 | 135.9 | 115.6 | 131.1 | 80.9 | 84.7 | 100.6 | 71.9 | 90.3 | 82.0 | 53.0 | 105.0 |
| ID52305 | 107.8 | 95.7 | 86.1 | 92.4 | 111.6 | 102.6 | 104.3 | 94.9 | 65.1 | 79.6 | 69.0 | 97.1 |
| ID52306 | 105.2 | 108.6 | 112.3 | 111.8 | 94.0 | 97.7 | 96.1 | 86.3 | 82.9 | 79.5 | 86.5 | 85.3 |
| ID52307 | 89.9 | 83.3 | 73.5 | 77.4 | 94.4 | 111.2 | 124.8 | 97.6 | 93.5 | 80.4 | 87.0 | 94.7 |
| ID52308 | 71.5 | 63.7 | 66.8 | 151.0 | 110.3 | 105.1 | 101.6 | 72.9 | 112.8 | 69.6 | 90.9 | 128.4 |
| ID52312 | 85.9 | 74.9 | 80.8 | 59.2 | 61.8 | 78.9 | 81.9 | 93.9 | 151.7 | 161.8 | 83.5 | 147.5 |
| ID52314 | 131.3 | 175.9 | 263.2 | 174.4 | 85.1 | 92.6 | 81.3 | 83.1 | 94.3 | 81.7 | 86.9 | 77.1 |
| ID52316 | 83.9 | 64.7 | 35.8 | 59.1 | 96.2 | 64.2 | 64.6 | 83.9 | 131.1 | 97.3 | 99.1 | 136.3 |
| ID52320 | 103.7 | 119.4 | 108.5 | 95.2 | 98.2 | 85.6 | 89.9 | 89.8 | 65.8 | 77.3 | 85.9 | 84.0 |
| ID52322 | 131.4 | 112.5 | 112.9 | 115.3 | 95.7 | 104.6 | 101.9 | 93.8 | 77.1 | 89.5 | 85.1 | 104.7 |
| ID52323 | 96.4 | 114.9 | 93.0 | 76.6 | 116.1 | 80.1 | 110.1 | 101.2 | 62.1 | 73.0 | 76.2 | 85.5 |
| ID52324 | 73.7 | 36.2 | 76.8 | 60.1 | 56.0 | 75.4 | 71.7 | 130.0 | 139.1 | 146.1 | 79.0 | 195.9 |
| ID1073 | 134.2 | 149.8 | 159.4 | 132.0 | 105.7 | 73.9 | 98.0 | 84.8 | 64.2 | 55.3 | 70.7 | 70.2 |
| ID52326 | 107.3 | 78.9 | 87.3 | 88.8 | 89.4 | 99.5 | 104.1 | 88.8 | 80.2 | 79.5 | 83.6 | 100.6 |
| ID52327 | 113.4 | 137.2 | 116.3 | 100.9 | 93.4 | 71.7 | 74.8 | 73.9 | 55.8 | 74.5 | 82.8 | 74.7 |
| ID52334 | 94.0 | 68.6 | 70.7 | 116.1 | 57.5 | 105.8 | 72.1 | 37.2 | 47.5 | 57.9 | 70.8 | 107.2 |
| ID52335 | 76.3 | 107.0 | 110.1 | 146.0 | 91.5 | 97.4 | 102.5 | 86.5 | 144.4 | 70.4 | 101.9 | 215.4 |
| ID52336 | 90.6 | 221.7 | 23.7 | 74.1 | 9.3 | 14.3 | 5.7 | 32.6 | 32.6 | 3.1 | 8.7 | 52.9 |
| ID52337 | 90.0 | 93.9 | 105.3 | 83.8 | 74.5 | 90.4 | 89.8 | 95.2 | 149.0 | 121.5 | 92.0 | 120.1 |
| ID52338 | 201.4 | 157.5 | 69.1 | 148.5 | 162.6 | 121.8 | 72.2 | 41.5 | 82.8 | 46.9 | 83.1 | 210.5 |
| ID52339 | 102.8 | 90.5 | 46.9 | 139.4 | 109.0 | 73.3 | 50.4 | 43.9 | 75.4 | 42.6 | 88.4 | 119.1 |
| ID52340 | 114.6 | 107.1 | 79.9 | 93.8 | 153.5 | 88.4 | 75.2 | 63.5 | 86.5 | 66.9 | 98.3 | 106.1 |
| ID52342 | 42.6 | 71.6 | 21.8 | 68.4 | 79.9 | 428.6 | 45.5 | 37.3 | 30.5 | 59.1 | 40.7 | 84.8 |
| ID52344 | 110.2 | 105.1 | 102.3 | 103.7 | 100.1 | 94.9 | 90.8 | 86.8 | 69.1 | 81.0 | 84.7 | 75.4 |
| ID52345 | 92.2 | 92.6 | 89.0 | 96.2 | 88.1 | 86.7 | 92.0 | 88.3 | 95.6 | 89.0 | 89.4 | 105.4 |
| ID52346 | 138.6 | 132.0 | 129.4 | 129.8 | 88.3 | 83.0 | 86.1 | 79.8 | 77.8 | 75.7 | 71.1 | 82.5 |
| ID52348 | 89.1 | 132.9 | 59.5 | 76.2 | 172.8 | 72.7 | 74.3 | 64.6 | 101.8 | 53.6 | 96.4 | 123.7 |
| ID52349 | 77.8 | 80.3 | 84.2 | 148.1 | 80.1 | 65.1 | 76.2 | 58.4 | 101.0 | 49.9 | 98.1 | 109.8 |
| ID1130 | 140.2 | 108.5 | 117.9 | 132.9 | 77.7 | 70.6 | 89.2 | 64.4 | 77.1 | 67.6 | 75.5 | 77.9 |
| ID52353 | 171.8 | 94.2 | 30.8 | 91.6 | 173.7 | 70.8 | 54.4 | 35.3 | 69.4 | 30.7 | 54.4 | 144.3 |
| ID52354 | 123.9 | 184.9 | 89.1 | 99.7 | 124.4 | 105.4 | 74.8 | 53.1 | 71.7 | 85.8 | 60.2 | 91.1 |
| ID52355 | 98.2 | 97.7 | 99.9 | 98.5 | 81.5 | 86.5 | 94.1 | 99.7 | 145.7 | 127.1 | 89.6 | 122.6 |
| ID52358 | 128.9 | 120.8 | 89.6 | 97.6 | 135.9 | 85.8 | 80.3 | 68.2 | 76.3 | 72.3 | 85.3 | 95.4 |
| ID52359 | 109.5 | 87.2 | 138.6 | 158.2 | 119.2 | 91.7 | 75.1 | 82.8 | 62.5 | 66.5 | 103.7 | 125.2 |
| ID52360 | 78.4 | 100.3 | 42.0 | 85.3 | 62.1 | 45.1 | 26.2 | 30.4 | 80.6 | 17.3 | 35.6 | 96.8 |
| ID52364 | 224.7 | 236.4 | 308.3 | 668.4 | 366.7 | 296.8 | 350.9 | 235.1 | 277.7 | 327.2 | 856.1 | 231.6 |
| ID52368 | 90.5 | 79.8 | 51.0 | 83.3 | 127.1 | 116.0 | 54.5 | 84.5 | 71.6 | 98.1 | 72.5 | 112.0 |
| ID52369 | 173.6 | 22.1 | 30.0 |  | 19.7 | 939.7 | 234.9 | 16.9 | 56.4 | 47.9 |  | 92.5 |
| ID52371 | 43.8 | 65.8 | 41.2 | 153.3 | 37.3 | 20.9 | 37.6 | 195.7 | 95.3 | 414.1 | 261.4 | 322.7 |
| ID52372 | 214.7 | 306.9 | 61.7 | 75.5 | 136.2 | 99.0 | 55.7 | 35.9 | 46.4 | 71.7 | 59.9 | 108.3 |
| ID52373 | 119.9 | 98.6 | 91.0 | 131.8 | 141.9 | 145.1 | 87.6 | 55.0 | 34.9 | 102.4 | 120.0 | 113.7 |
| ID52376 | 62.2 | 75.3 | 83.8 | 118.3 | 134.7 | 131.8 | 285.5 | 5.5 | 25.0 | 16.2 | 77.2 | 10.0 |
| ID52381 | 136.9 | 198.3 | 76.7 | 66.9 | 123.8 | 89.9 | 74.7 | 55.3 | 92.9 | 79.2 | 74.4 | 113.4 |
| ID52382 | 91.1 | 108.8 | 139.5 | 90.2 | 105.0 | 86.3 | 122.5 | 53.9 | 121.7 | 66.5 | 56.1 | 109.0 |
| ID52383 | 109.1 | 98.8 | 87.1 | 82.4 | 127.4 | 117.9 | 117.6 | 79.6 | 38.3 | 100.1 | 72.5 | 130.8 |
| ID52384 | 111.4 | 152.4 | 66.1 | 37.4 | 128.6 | 89.7 | 55.9 | 37.5 | 90.6 | 67.5 | 50.3 | 86.3 |
| ID52385 | 186.9 | 203.1 | 87.4 | 88.5 | 201.2 | 54.3 | 419.8 | 9.7 | 138.1 | 4.8 | 13.1 | 23.6 |
| ID52386 | 55.2 | 262.7 | 157.6 | 122.6 | 95.5 | 109.9 | 180.8 | 125.5 | 98.0 | 64.8 | 87.3 | 73.8 |
| ID52387 | 121.7 | 806.5 | 163.7 | 44.6 | 90.0 | 42.9 | 54.6 | 169.9 | 112.6 | 48.0 | 163.3 | 206.1 |
| ID52388 | 79.6 | 88.3 | 59.7 | 69.2 | 95.2 | 112.1 | 58.9 | 88.2 | 56.6 | 105.7 | 56.6 | 118.6 |
| ID52389 | 93.9 | 111.0 | 120.2 | 129.8 | 162.9 | 116.7 | 132.1 | 83.4 | 21.7 | 40.0 | 116.8 | 129.4 |
| ID52392 | 76.0 | 97.8 | 82.3 | 122.6 | 103.8 | 117.4 | 145.2 | 85.3 | 54.3 | 27.6 | 74.1 | 62.1 |
| ID52393 | 79.2 | 179.9 | 40.7 | 15.3 | 160.9 | 125.5 | 58.3 | 58.5 | 84.1 | 69.8 | 165.1 | 305.4 |
| ID52394 | 85.8 | 81.4 | 69.4 | 83.8 | 116.2 | 127.4 | 75.9 | 70.7 | 79.1 | 124.9 | 64.6 | 106.7 |
| ID52395 | 64.1 | 42.6 | 54.4 | 22.4 | 72.5 | 80.5 | 66.0 | 68.8 | 56.1 | 67.7 | 11.2 | 130.3 |
| ID52396 | 94.4 | 57.1 | 63.9 | 105.9 | 57.1 | 99.3 | 81.8 | 103.7 | 97.8 | 79.4 | 73.1 | 142.0 |
| ID52397 | 15.1 | 49.4 | 184.6 | 14.3 | 174.1 | 92.6 | 111.0 | 152.0 | 82.1 | 62.9 | 92.6 | 97.5 |
| ID52398 | 67.1 | 74.6 | 56.6 | 64.7 | 99.7 | 95.9 | 97.3 | 84.8 | 68.9 | 60.1 | 84.4 | 82.4 |
| ID52399 | 62.7 | 122.1 | 83.2 | 119.6 | 100.3 | 88.7 | 115.8 | 103.1 | 104.6 | 72.0 | 85.3 | 82.9 |
| ID52400 | 46.5 | 68.6 | 12.1 | 31.7 | 137.2 | 237.7 | 73.9 | 91.9 | 120.3 | 128.9 | 176.5 | 146.9 |
| ID52401 | 185.2 | 175.3 | 85.3 | 17.2 | 177.4 | 48.2 | 80.3 | 40.9 | 32.5 | 121.8 | 87.4 | 94.5 |
| ID52404 | 109.9 | 122.4 | 124.6 | 124.8 | 105.9 | 89.2 | 113.8 | 94.9 | 83.4 | 76.0 | 74.1 | 88.1 |
| ID52406 | 84.9 | 34.9 | 47.2 | 89.4 | 32.1 | 70.0 | 49.4 | 22.0 | 32.1 | 37.1 | 53.5 | 52.2 |
| ID52407 | 86.2 | 76.8 | 82.9 | 83.0 | 75.1 | 93.9 | 96.4 | 84.6 | 117.5 | 91.0 | 89.5 | 100.9 |
| ID52408 | 63.3 | 45.8 | 35.7 | 80.1 | 34.4 | 64.3 | 57.0 | 38.1 | 42.3 | 28.7 | 40.5 | 69.3 |
| ID52409 | 97.3 | 138.3 | 152.8 | 150.8 | 51.8 | 74.3 | 66.4 | 67.0 | 94.1 | 68.5 | 51.1 | 73.0 |
| ID52413 | 45.0 | 43.2 | 58.9 | 61.2 | 64.2 | 86.7 | 56.7 | 51.1 | 40.1 | 52.4 | 64.9 | 62.7 |
| ID52415 | 199.1 | 361.7 | 29.4 | 85.3 | 24.2 | 30.2 | 36.7 | 20.1 | 34.6 | 15.1 | 23.5 | 56.9 |
| ID52416 | 115.8 | 353.5 | 145.1 | 521.4 | 30.6 | 47.1 | 20.6 | 46.4 | 63.9 | 101.7 | 207.5 | 45.2 |
| ID52417 | 75.1 | 178.9 | 84.1 | 555.2 | 109.5 | 128.8 | 88.8 | 163.8 | 166.2 | 238.1 | 1061.7 | 177.7 |
| ID52418 | 87.8 | 75.2 | 77.9 | 66.3 | 68.2 | 87.9 | 96.5 | 83.8 | 63.0 | 56.0 | 74.9 | 83.4 |
| ID52421 | 686.1 | 280.8 | 15.3 | 2.3 | 354.4 | 94.3 | 17.8 | 13.6 | 15.3 | 432.7 | 46.6 | 77.4 |
| ID52422 | 74.9 | 36.3 | 34.8 | 69.8 | 31.7 | 58.6 | 90.0 | 51.7 | 132.1 | 52.1 | 45.0 | 122.7 |
| ID52424 | 29.9 | 47.3 | 31.6 | 69.8 | 42.4 | 63.4 | 75.6 | 39.3 | 25.4 | 6.6 | 41.9 | 28.8 |
| ID52426 | 262.5 | 174.4 | 103.9 | 114.9 | 38.1 | 51.7 | 98.2 | 22.0 | 68.6 | 36.9 | 35.4 | 90.3 |
| ID52428 | 141.4 | 163.0 | 105.2 | 150.9 | 59.3 | 57.8 | 57.9 | 48.7 | 69.1 | 43.1 | 50.4 | 61.7 |
| ID52430 | 92.6 | 303.0 | 145.3 | 131.5 | 43.0 | 46.4 | 47.8 | 35.4 | 54.5 | 55.8 | 59.7 | 29.4 |
| ID52432 | 179.5 | 151.4 | 112.5 | 124.3 | 184.8 | 125.6 | 246.5 | 38.2 | 108.5 | 44.1 | 41.4 | 98.0 |
| ID52433 | 18.5 | 11.4 | 17.4 | 51.7 | 21.3 | 52.5 | 63.6 | 38.4 | 103.9 | 36.8 | 38.2 | 78.3 |
| ID52434 | 30.7 | 35.3 | 96.9 | 67.2 | 83.6 | 113.5 | 112.3 | 86.0 | 114.9 | 59.9 | 69.0 | 125.3 |
| ID52436 | 87.5 | 311.5 | 146.4 | 820.5 | 68.7 | 186.1 | 68.9 | 144.6 | 92.2 | 198.4 | 645.7 | 144.8 |
| ID52437 | 1853.3 | 758.6 | 20.7 | 0.0 | 522.3 | 107.5 | 7.9 | 9.2 | 9.8 | 401.3 | 92.8 | 126.9 |
| ID52440 | 113.6 | 126.1 | 106.6 | 93.1 | 125.0 | 116.3 | 128.2 | 93.9 | 88.4 | 89.9 | 107.5 | 100.6 |
| ID52441 | 673.7 | 584.2 | 601.5 | 190.7 | 969.6 | 108.2 | 682.7 | 35.6 | 236.6 | 84.8 | 140.7 | 59.3 |
| ID52443 | 31.5 | 40.2 | 67.4 | 63.1 | 27.2 | 46.5 | 55.4 | 29.6 | 67.3 | 30.0 | 25.4 | 61.8 |
| ID52446 | 201.5 | 225.6 | 141.5 | 93.9 | 182.5 | 102.7 | 127.4 | 71.8 | 72.3 | 71.3 | 109.1 | 64.2 |
| ID52449 | 1210.0 | 428.9 | 13.1 | 0.8 | 19.9 | 40.6 | 9.2 | 36.2 | 2.4 | 26.1 | 16.7 | 59.7 |
| ID52450 | 171.3 | 220.4 | 122.1 | 84.3 | 243.1 | 121.0 | 141.8 | 74.5 | 69.3 | 70.8 | 112.3 | 66.9 |
| ID52451 | 36.7 | 59.6 | 70.0 | 92.2 | 132.0 | 371.5 | 99.5 | 156.7 | 97.5 | 286.5 | 282.8 | 125.7 |
| ID52452 | 123.4 | 129.1 | 142.8 | 205.0 | 59.2 | 100.5 | 70.4 | 75.4 | 46.6 | 101.9 | 119.3 | 68.4 |
| ID52453 | 20.8 | 29.9 | 45.6 | 27.4 | 113.4 | 523.0 | 144.3 | 114.0 | 81.8 | 391.3 | 213.0 | 97.9 |
| ID52457 | 65.9 | 40.3 | 137.1 | 122.7 | 26.8 | 75.6 | 101.6 | 93.6 | 293.4 | 75.7 | 24.0 | 82.9 |
| ID52458 | 86.0 | 75.9 | 84.7 | 98.0 | 89.2 | 103.9 | 114.6 | 89.8 | 120.4 | 82.2 | 86.1 | 109.4 |
| ID52459 | 86.8 | 148.6 | 125.3 | 123.3 | 117.6 | 119.8 | 92.9 | 79.8 | 142.9 | 116.0 | 119.0 | 93.6 |
| ID52461 | 51.6 | 73.9 | 74.0 | 63.4 | 82.1 | 78.3 | 93.1 | 85.9 | 70.6 | 84.4 | 99.7 | 111.0 |
| ID52464 | 34.6 | 35.0 | 28.6 | 37.2 | 86.4 | 155.6 | 122.6 | 100.6 | 112.2 | 108.5 | 115.2 | 100.1 |
| ID52465 | 194.1 | 153.6 | 109.0 | 194.8 | 236.9 | 207.0 | 128.0 | 109.3 | 181.1 | 216.6 | 256.3 | 124.1 |
| ID52468 | 62.0 | 51.6 | 124.8 | 135.1 | 61.2 | 73.6 | 94.1 | 102.4 | 49.5 | 58.1 | 49.3 | 72.0 |
| ID52472 | 143.4 | 222.2 | 229.7 | 195.2 | 107.3 | 109.4 | 132.2 | 123.1 | 77.6 | 96.1 | 133.5 | 62.2 |
| ID52473 | 164.1 | 212.4 | 102.3 | 79.0 | 256.8 | 144.4 | 149.0 | 76.4 | 58.4 | 79.3 | 104.7 | 72.4 |
| ID52474 | 384.4 | 561.1 | 281.4 | 489.1 | 296.3 | 393.7 | 480.1 | 226.9 | 333.6 | 224.8 | 182.0 | 396.5 |
| ID52475 | 155.6 | 235.8 | 163.7 | 162.5 | 89.0 | 78.5 | 69.4 | 38.9 | 86.0 | 54.4 | 73.6 | 48.4 |
| ID52477 | 93.2 | 100.3 | 164.5 | 120.8 | 139.8 | 178.2 | 212.5 | 445.9 | 140.4 | 197.1 | 119.6 | 179.5 |
| ID52478 | 160.6 | 206.6 | 222.2 | 219.1 | 156.0 | 124.2 | 123.8 | 105.9 | 255.7 | 228.3 | 219.5 | 116.0 |
| ID52479 | 168.8 | 83.0 | 129.7 | 147.4 | 79.6 | 93.4 | 110.4 | 128.9 | 96.9 | 94.7 | 84.3 | 100.5 |
| ID52481 | 14.6 | 19.6 | 12.4 | 6.9 | 105.3 | 102.6 | 152.1 | 105.9 | 155.7 | 78.3 | 100.5 | 146.6 |
| ID52482 | 82.0 | 76.3 | 61.9 | 87.7 | 79.0 | 156.9 | 123.1 | 89.0 | 90.9 | 106.5 | 65.9 | 190.9 |
| ID52483 | 14.2 | 19.4 | 25.2 | 27.8 | 70.0 | 105.8 | 135.4 | 114.6 | 144.9 | 78.0 | 104.3 | 156.6 |
| ID52484, ID52516, ID167848 | 30.6 | 113.6 | 29.3 | 1429.4 | 24.0 | 107.5 | 149.4 | 72.6 | 253.4 | 14.1 | 33.7 | 138.3 |
| ID52485 | 89.8 | 68.8 | 41.9 | 68.4 | 73.1 | 179.0 | 141.8 | 77.3 | 240.3 | 53.9 | 14.7 | 207.9 |
| ID52486 | 62.7 | 67.5 | 12.2 | 34.9 | 385.8 | 492.8 | 7.6 | 27.0 | 118.4 | 350.8 | 295.2 | 210.1 |
| ID52489 | 28.1 | 90.3 | 137.1 | 149.1 | 190.9 | 139.5 | 189.7 | 120.3 | 120.2 | 84.7 | 192.9 | 83.2 |
| ID52490 | 92.9 | 74.3 | 140.2 | 193.1 | 42.8 | 70.7 | 74.7 | 131.1 | 189.8 | 135.5 | 82.2 | 140.9 |
| ID52491 | 216.6 | 170.0 | 152.5 | 165.2 | 141.1 | 105.7 | 122.9 | 79.1 | 52.2 | 69.6 | 133.2 | 59.2 |
| ID52493 | 192.4 | 174.0 | 48.6 | 56.8 | 351.9 | 284.8 | 88.5 | 65.6 | 45.3 | 154.7 | 146.2 | 111.4 |
| ID52494 | 71.5 | 114.6 | 107.5 | 179.0 | 12.2 | 26.5 | 11.0 | 53.0 | 41.5 | 19.2 | 11.6 | 94.8 |
| ID52495 | 138.7 | 68.6 | 69.4 | 104.7 | 61.5 | 84.9 | 91.5 | 79.2 | 107.9 | 98.6 | 61.5 | 116.7 |
| ID52496 | 161.6 | 123.9 | 72.7 | 153.7 | 182.0 | 40.0 | 73.6 | 60.4 | 74.7 | 14.7 | 92.2 | 77.2 |
| ID52497 | 124.5 | 126.5 | 98.5 | 131.0 | 105.0 | 100.9 | 121.4 | 111.7 | 69.7 | 77.2 | 94.8 | 95.0 |
| ID52503 | 105.7 | 80.5 | 59.2 | 67.3 | 78.5 | 83.7 | 111.4 | 79.7 | 107.7 | 84.4 | 98.6 | 132.1 |
| ID52504 | 63.3 | 76.0 | 62.5 | 60.3 | 142.2 | 81.6 | 90.2 | 89.0 | 88.1 | 53.6 | 99.4 | 143.8 |
| ID52506 | 132.5 | 125.8 | 97.8 | 131.2 | 48.2 | 82.6 | 91.5 | 78.1 | 88.9 | 61.7 | 56.1 | 80.4 |
| ID52507 | 111.4 | 143.1 | 64.1 | 76.6 | 206.5 | 75.0 | 104.6 | 71.1 | 64.2 | 25.5 | 114.8 | 105.0 |
| ID52508 | 64.2 | 59.5 | 70.4 | 64.1 | 99.4 | 93.5 | 95.4 | 86.4 | 85.2 | 68.9 | 89.5 | 97.2 |
| ID52509 | 59.4 | 39.4 | 38.2 | 42.3 | 68.6 | 98.1 | 174.5 | 123.8 | 156.9 | 66.6 | 71.2 | 137.3 |
| ID52510 | 244.1 | 328.0 | 75.1 | 68.7 | 143.5 | 93.1 | 95.6 | 38.7 | 59.1 | 83.5 | 89.5 | 86.9 |
| ID1324 | 150.7 | 146.5 | 115.0 | 115.0 | 89.8 | 87.4 | 87.2 | 74.7 | 72.8 | 64.0 | 70.8 | 72.9 |
| ID52513 | 186.1 | 363.4 | 128.2 | 119.3 | 137.7 | 100.4 | 94.5 | 87.5 | 155.8 | 100.4 | 104.0 | 106.6 |
| ID52514 | 91.3 | 114.3 | 78.4 | 123.7 | 47.2 | 76.7 | 81.8 | 76.8 | 40.9 | 45.1 | 63.0 | 98.8 |
| ID52515 | 177.5 | 253.4 | 102.1 | 92.2 | 103.2 | 85.6 | 106.6 | 98.3 | 27.9 | 47.4 | 81.6 | 85.2 |
| ID52519 | 137.0 | 167.3 | 91.1 | 112.5 | 142.0 | 48.8 | 112.3 | 57.4 | 54.2 | 21.6 | 106.7 | 77.8 |
| ID52520 | 85.6 | 79.4 | 67.1 | 79.6 | 79.4 | 92.8 | 113.3 | 89.7 | 106.6 | 111.7 | 113.0 | 133.1 |
| ID52521 | 91.4 | 98.1 | 72.2 | 83.5 | 111.5 | 108.7 | 119.4 | 89.7 | 80.7 | 87.8 | 80.6 | 148.1 |
| ID52522 | 175.4 | 138.3 | 106.4 | 114.5 | 75.1 | 78.6 | 95.1 | 69.3 | 77.3 | 81.1 | 75.5 | 95.6 |
| ID52524 | 88.6 | 100.6 | 82.7 | 86.4 | 101.2 | 108.7 | 113.5 | 104.5 | 92.2 | 103.4 | 102.8 | 112.3 |
| ID52525 | 171.0 | 116.8 | 97.4 | 136.5 | 63.4 | 62.7 | 70.7 | 68.4 | 86.6 | 69.2 | 59.7 | 72.6 |
| ID52528 | 260.7 | 165.8 | 145.7 | 525.1 | 202.7 | 195.1 | 151.5 | 104.8 | 175.4 | 170.3 | 359.7 | 117.1 |
| ID52529 | 143.8 | 127.8 | 112.3 | 119.3 | 79.8 | 88.3 | 90.8 | 87.9 | 77.4 | 80.8 | 61.2 | 109.4 |
| ID52530 | 69.2 | 92.8 | 138.4 | 95.7 | 115.6 | 87.6 | 266.2 | 72.5 | 172.6 | 141.6 | 163.2 | 114.7 |
| ID52531 | 75.5 | 66.4 | 69.7 | 88.1 | 71.9 | 89.9 | 85.9 | 66.0 | 135.4 | 111.7 | 90.0 | 114.0 |
| ID52532 | 125.1 | 109.9 | 82.8 | 103.2 | 95.9 | 80.4 | 82.6 | 53.2 | 77.4 | 65.6 | 80.2 | 91.0 |
| ID52533 | 125.4 | 139.3 | 107.7 | 93.8 | 71.7 | 50.8 | 57.8 | 63.2 | 58.3 | 77.3 | 72.0 | 71.9 |
| ID52534 | 234.4 | 228.0 | 136.9 | 422.8 | 110.4 | 280.2 | 271.0 | 131.1 | 262.5 | 155.5 | 110.1 | 303.8 |
| ID52535 | 78.4 | 72.4 | 65.7 | 74.5 | 64.3 | 63.4 | 84.7 | 48.7 | 109.0 | 95.8 | 84.4 | 105.8 |
| ID52537 | 102.5 | 118.5 | 112.6 | 110.6 | 88.5 | 103.8 | 95.6 | 78.1 | 80.7 | 81.5 | 86.6 | 100.4 |
| ID52538 | 15.7 | 181.8 | 12.9 | 169.4 | 10.6 | 128.7 | 36.2 | 47.0 | 316.5 | 16.0 | 10.1 | 123.2 |
| ID52539 | 208.7 | 108.8 | 115.0 | 89.9 | 115.2 | 90.1 | 96.8 | 91.4 | 115.4 | 105.5 | 66.9 | 103.2 |
| ID52540 | 165.4 | 103.3 | 96.6 | 93.5 | 98.1 | 76.6 | 109.5 | 81.2 | 101.1 | 89.7 | 70.5 | 99.5 |
| ID52542 | 104.6 | 104.4 | 124.1 | 127.8 | 67.9 | 104.8 | 133.5 | 63.8 | 55.6 | 71.6 | 55.1 | 107.6 |
| ID52543 | 98.8 | 92.2 | 86.4 | 115.0 | 57.2 | 99.4 | 77.6 | 58.9 | 54.6 | 67.7 | 47.0 | 94.5 |
| ID52544 | 101.5 | 90.3 | 76.4 | 86.7 | 70.2 | 80.6 | 83.1 | 76.3 | 106.7 | 77.4 | 68.9 | 99.0 |
| ID52546 | 63.4 | 71.5 | 69.3 | 79.9 | 78.0 | 88.5 | 88.2 | 64.4 | 60.7 | 68.8 | 64.0 | 83.5 |
| ID52547 | 86.9 | 65.2 | 64.2 | 96.7 | 56.6 | 57.3 | 78.4 | 64.1 | 63.6 | 44.3 | 34.1 | 119.5 |
| ID52550 | 317.2 | 395.0 | 149.5 | 161.6 | 69.7 | 36.3 | 27.4 | 25.6 | 29.9 | 46.6 | 29.3 | 49.5 |
| ID52551 | 149.1 | 138.1 | 99.4 | 130.0 | 55.0 | 71.6 | 73.7 | 53.4 | 85.0 | 78.5 | 47.6 | 87.8 |
| ID52552 | 84.9 | 34.3 | 46.7 | 49.8 | 96.8 | 89.5 | 155.9 | 75.9 | 117.4 | 103.3 | 95.7 | 166.9 |
| ID52555 | 56.9 | 52.4 | 63.3 | 77.9 | 53.7 | 84.4 | 79.4 | 57.8 | 129.5 | 87.3 | 60.5 | 105.2 |
| ID52557 | 57.9 | 58.5 | 61.4 | 84.3 | 56.1 | 91.1 | 81.7 | 52.0 | 96.9 | 94.3 | 45.6 | 108.1 |
| ID52558 | 61.8 | 63.1 | 45.8 | 97.1 | 59.0 | 86.9 | 78.9 | 52.2 | 110.9 | 91.7 | 50.9 | 109.6 |
| ID52561 | 392.9 | 308.9 | 100.0 | 149.9 | 100.5 | 40.6 | 15.6 | 12.6 | 32.0 | 52.2 | 38.5 | 62.2 |
| ID52562 | 93.3 | 127.5 | 51.4 | 39.9 | 84.7 | 77.3 | 56.6 | 48.1 | 84.9 | 98.7 | 47.3 | 86.2 |
| ID52563 | 357.9 | 300.3 | 159.8 | 245.6 | 22.8 | 22.0 | 22.9 | 18.0 | 27.7 | 26.6 | 23.6 | 39.9 |
| ID52564 | 91.4 | 128.2 | 98.9 | 135.3 | 91.4 | 121.4 | 120.4 | 71.7 | 83.9 | 83.3 | 82.1 | 108.6 |
| ID52569 | 151.3 | 148.2 | 46.1 | 133.1 | 92.7 | 279.5 | 101.8 | 197.4 | 149.2 | 334.9 | 215.9 | 296.4 |
| ID52570 | 147.7 | 327.9 | 90.1 | 107.9 | 58.0 | 127.2 | 45.5 | 113.4 | 112.3 | 108.2 | 126.0 | 140.8 |
| ID52572 | 79.8 | 80.4 | 72.0 | 70.0 | 93.8 | 90.1 | 116.7 | 91.3 | 113.6 | 96.1 | 111.2 | 123.2 |
| ID52573 | 315.8 | 291.9 | 129.3 | 195.4 | 32.5 | 40.5 | 18.9 | 28.9 | 25.7 | 31.8 | 27.5 | 40.6 |
| ID52574 | 121.3 | 98.1 | 82.7 | 95.4 | 80.0 | 84.1 | 77.2 | 65.0 | 64.7 | 66.9 | 66.5 | 73.6 |
| ID52576 | 46.7 | 53.6 | 45.7 | 73.9 | 72.4 | 165.5 | 195.4 | 48.7 | 147.4 | 29.3 | 23.8 | 199.8 |
| ID52578 | 46.5 | 47.1 | 30.6 | 26.3 | 130.3 | 163.9 | 182.5 | 114.4 | 116.4 | 165.2 | 101.7 | 177.3 |
| ID52580 | 113.9 | 183.4 | 97.7 | 200.3 | 56.5 | 93.7 | 81.1 | 34.1 | 55.5 | 43.1 | 41.1 | 68.7 |
| ID52581 | 39.7 | 99.4 | 62.9 | 51.3 | 112.9 | 63.8 | 71.6 | 92.4 | 63.6 | 72.4 | 99.2 | 96.9 |
| ID52583 | 59.9 | 63.9 | 62.9 | 46.6 | 130.8 | 70.1 | 89.4 | 72.4 | 70.8 | 72.5 | 78.5 | 114.2 |
| ID52586 | 78.0 | 68.5 | 60.5 | 63.5 | 127.9 | 71.4 | 127.7 | 64.7 | 132.7 | 73.3 | 95.4 | 123.8 |
| ID52591 | 40.7 | 32.4 | 42.4 | 23.4 | 98.8 | 87.2 | 82.8 | 72.3 | 44.1 | 75.7 | 101.2 | 85.5 |
| ID52592 | 93.7 | 145.7 | 98.9 | 100.8 | 111.3 | 105.2 | 109.4 | 99.5 | 106.3 | 100.7 | 93.7 | 108.2 |
| ID52593 | 58.0 | 36.1 | 45.1 | 121.1 | 64.3 | 135.0 | 148.5 | 168.5 | 165.7 | 275.6 | 219.4 | 317.0 |
| ID1452 | 375.0 | 137.6 | 168.5 | 293.5 | 171.0 | 42.5 | 45.1 | 54.8 | 3.7 | 19.5 | 24.5 | 26.0 |
| ID52596 | 245.1 | 230.7 | 177.4 | 381.2 | 177.1 | 234.1 | 178.8 | 97.9 | 178.0 | 215.2 | 305.0 | 128.8 |
| ID52599 | 43.4 | 49.3 | 39.4 | 43.3 | 112.7 | 114.2 | 158.9 | 79.4 | 123.5 | 134.2 | 77.9 | 146.0 |
| ID52600 | 56.6 | 37.4 | 8.7 | 66.3 | 65.8 | 221.8 | 35.9 | 96.2 | 92.3 | 199.6 | 185.9 | 192.7 |
| ID52601 | 58.5 | 59.0 | 24.9 | 58.6 | 82.0 | 223.3 | 50.2 | 85.4 | 77.9 | 202.8 | 135.2 | 187.2 |
| ID52602 | 83.9 | 85.7 | 34.5 | 95.6 | 64.4 | 158.9 | 26.4 | 71.1 | 96.4 | 149.8 | 170.1 | 138.8 |
| ID52603 | 39.8 | 82.9 | 68.4 | 46.5 | 93.5 | 50.0 | 92.5 | 60.7 | 138.6 | 114.9 | 203.0 | 235.1 |
| ID52605 | 120.0 | 136.8 | 122.9 | 98.9 | 105.4 | 118.5 | 126.0 | 90.5 | 114.0 | 140.8 | 93.7 | 112.0 |
| ID52607 | 32.3 | 97.2 | 27.9 | 112.6 | 98.8 | 119.5 | 150.4 | 128.3 | 253.4 | 230.7 | 132.0 | 229.2 |
| ID52608 | 148.3 | 143.1 | 136.4 | 149.8 | 81.3 | 88.8 | 94.5 | 73.5 | 66.7 | 59.4 | 56.9 | 70.9 |
| ID52609 | 143.8 | 282.8 | 153.2 | 126.2 | 129.9 | 179.2 | 178.3 | 120.2 | 173.3 | 144.3 | 105.1 | 211.5 |
| ID52611 | 256.4 | 209.7 | 199.2 | 312.4 | 61.1 | 29.8 | 49.7 | 77.4 | 87.0 | 33.4 | 38.3 | 62.4 |
| ID52613 | 40.4 | 57.7 | 65.3 | 67.7 | 97.0 | 110.1 | 130.2 | 129.1 | 91.8 | 107.4 | 138.0 | 122.3 |
| ID52614 | 349.6 | 460.4 | 272.9 | 162.5 | 290.4 | 140.6 | 217.3 | 82.3 | 60.7 | 88.9 | 185.0 | 81.7 |
| ID52615 | 108.6 | 144.3 | 94.1 | 99.2 | 97.4 | 113.1 | 119.1 | 99.9 | 113.1 | 101.9 | 99.1 | 110.8 |
| ID52616 | 65.1 | 85.7 | 101.7 | 101.4 | 89.3 | 94.7 | 126.9 | 109.6 | 79.5 | 81.7 | 105.6 | 94.9 |
| ID52617 | 94.9 | 118.0 | 108.7 | 109.4 | 101.7 | 91.6 | 107.7 | 96.6 | 58.1 | 60.1 | 89.3 | 76.1 |
| ID52620 | 63.8 | 70.8 | 37.7 | 106.3 | 22.4 | 42.6 | 38.7 | 17.6 | 36.6 | 25.2 | 15.5 | 55.4 |
| ID52621 | 183.7 | 203.6 | 263.7 | 274.4 | 54.6 | 79.6 | 74.8 | 66.0 | 27.3 | 36.9 | 31.2 | 36.5 |
| ID52622 | 37.4 | 75.5 | 83.8 | 82.4 | 111.1 | 94.6 | 123.7 | 111.8 | 74.4 | 75.8 | 113.7 | 92.0 |
| ID52623 | 62.6 | 110.3 | 114.7 | 130.1 | 103.5 | 116.0 | 156.3 | 142.6 | 57.6 | 81.7 | 158.7 | 96.2 |
| ID52624 | 53.3 | 89.6 | 84.0 | 72.8 | 85.1 | 77.8 | 84.7 | 78.7 | 72.5 | 74.6 | 70.6 | 98.2 |
| ID52625 | 43.5 | 71.2 | 77.4 | 57.7 | 93.3 | 82.7 | 89.7 | 83.3 | 64.2 | 72.6 | 77.7 | 90.4 |
| ID52626 | 47.5 | 75.5 | 100.0 | 69.8 | 88.3 | 70.1 | 90.1 | 80.9 | 74.4 | 79.5 | 82.6 | 101.5 |
| ID52627 | 359.6 | 239.2 | 258.6 | 553.7 | 386.2 | 364.8 | 380.3 | 146.9 | 310.3 | 297.0 | 560.2 | 173.5 |
| ID52628 | 55.5 | 102.8 | 97.1 | 104.3 | 112.4 | 118.4 | 152.0 | 132.1 | 56.0 | 95.6 | 156.2 | 110.6 |
| ID52629 | 91.5 | 103.1 | 123.2 | 143.3 | 101.8 | 109.1 | 125.8 | 120.8 | 63.7 | 85.1 | 102.8 | 83.2 |
| ID52631 | 97.1 | 160.0 | 117.3 | 139.8 | 142.7 | 196.5 | 105.3 | 182.3 | 94.2 | 230.2 | 235.5 | 416.6 |
| ID52634 | 33.4 | 33.2 | 74.8 | 35.3 | 83.7 | 79.6 | 86.3 | 75.7 | 86.1 | 110.9 | 91.4 | 95.4 |
| ID52635 | 75.1 | 82.6 | 64.7 | 89.4 | 54.8 | 138.9 | 82.0 | 60.2 | 61.8 | 59.5 | 42.0 | 103.0 |
| ID52636 | 119.3 | 119.9 | 76.5 | 92.9 | 59.5 | 94.3 | 111.4 | 55.3 | 124.0 | 74.8 | 58.0 | 109.2 |
| ID52638 | 152.3 | 347.6 | 193.2 | 337.4 | 145.4 | 351.8 | 253.0 | 319.1 | 640.2 | 423.8 | 245.7 | 389.6 |
| ID52639 | 84.6 | 102.9 | 158.6 | 168.0 | 61.4 | 75.2 | 112.7 | 71.7 | 65.2 | 60.2 | 52.0 | 110.4 |
| ID52640 | 55.2 | 91.1 | 64.2 | 60.2 | 98.3 | 282.4 | 96.8 | 92.7 | 204.9 | 239.6 | 153.4 | 188.7 |
| ID52644 | 85.9 | 119.8 | 78.1 | 81.4 | 96.4 | 116.4 | 125.1 | 145.4 | 86.2 | 115.5 | 103.5 | 155.7 |
| ID52647 | 171.6 | 166.0 | 115.6 | 178.2 | 31.6 | 126.8 | 94.0 | 16.8 | 47.1 | 17.4 | 6.1 | 64.7 |
| ID52651 | 172.8 | 170.6 | 129.9 | 147.5 | 59.4 | 65.7 | 75.1 | 76.2 | 77.2 | 91.9 | 67.4 | 83.5 |
| ID52652 | 81.9 | 164.7 | 83.5 | 100.0 | 94.9 | 146.8 | 111.3 | 122.4 | 181.3 | 121.9 | 73.9 | 132.0 |
| ID52653 | 67.3 | 60.5 | 96.8 | 121.5 | 68.9 | 89.6 | 72.5 | 145.5 | 100.1 | 112.6 | 83.8 | 111.1 |
| ID52654 | 166.9 | 287.6 | 71.4 | 70.0 | 94.8 | 78.9 | 76.0 | 63.0 | 37.9 | 69.6 | 93.6 | 72.9 |
| ID52656 | 79.4 | 108.7 | 87.6 | 58.8 | 106.2 | 88.3 | 93.0 | 88.1 | 57.3 | 93.1 | 93.8 | 89.4 |
| ID52657 | 107.3 | 100.3 | 93.0 | 84.5 | 71.7 | 84.4 | 94.3 | 104.5 | 94.7 | 124.6 | 90.4 | 119.9 |
| ID52658 | 140.3 | 200.9 | 116.8 | 102.6 | 89.8 | 88.5 | 99.4 | 78.1 | 94.2 | 73.4 | 81.9 | 81.7 |
| ID52659 | 240.1 | 428.8 | 196.8 | 105.7 | 36.4 | 36.0 | 41.7 | 16.7 | 40.1 | 30.3 | 33.2 | 35.4 |
| ID52660 | 240.1 | 435.2 | 159.9 | 275.7 | 41.6 | 35.5 | 34.1 | 26.4 | 37.3 | 33.2 | 26.0 | 33.3 |
| ID52661 | 77.6 | 46.2 | 57.5 | 39.6 | 59.7 | 67.1 | 94.6 | 96.8 | 124.0 | 130.8 | 88.6 | 129.9 |
| ID52662 | 81.6 | 37.4 | 60.3 | 44.8 | 73.3 | 83.8 | 84.5 | 98.6 | 135.4 | 134.4 | 82.4 | 130.8 |
| ID66139 | 252.4 | 176.9 | 207.3 | 176.3 | 62.1 | 62.1 | 40.5 | 68.6 | 45.5 | 59.4 | 60.5 | 65.6 |
| ID52663 | 212.3 | 393.6 | 250.3 | 210.1 | 99.9 | 54.8 | 93.0 | 86.7 | 62.8 | 48.7 | 129.2 | 122.1 |
| ID52664 | 145.1 | 261.1 | 113.9 | 88.1 | 157.0 | 95.0 | 100.1 | 57.0 | 108.6 | 81.9 | 106.9 | 76.7 |
| ID52665 | 97.9 | 95.0 | 78.6 | 91.5 | 112.6 | 146.2 | 103.9 | 84.7 | 118.0 | 117.8 | 109.8 | 100.6 |
| ID52666 | 92.2 | 96.2 | 87.5 | 68.8 | 94.4 | 113.7 | 107.6 | 84.5 | 100.0 | 113.3 | 73.9 | 98.6 |
| ID52667 | 103.6 | 110.1 | 112.7 | 77.9 | 95.5 | 84.2 | 88.2 | 102.7 | 71.5 | 83.5 | 90.5 | 97.9 |
| ID52670 | 95.6 | 117.9 | 85.4 | 74.6 | 96.9 | 102.1 | 85.0 | 73.0 | 66.3 | 103.5 | 82.0 | 98.0 |
| ID52671 | 152.7 | 228.9 | 106.4 | 82.5 | 145.3 | 102.5 | 102.1 | 63.8 | 77.8 | 88.8 | 78.5 | 112.3 |
| ID52672 | 107.5 | 79.4 | 79.0 | 62.8 | 83.9 | 81.3 | 75.8 | 89.2 | 130.0 | 136.0 | 66.2 | 126.8 |
| ID52673 | 116.1 | 128.3 | 91.1 | 52.2 | 101.6 | 117.6 | 99.7 | 57.3 | 91.9 | 110.9 | 50.8 | 115.6 |
| ID52675 | 109.0 | 160.2 | 101.9 | 88.0 | 132.1 | 87.1 | 121.1 | 91.3 | 124.5 | 102.9 | 153.8 | 152.4 |
| ID52676 | 118.0 | 151.5 | 109.5 | 108.4 | 86.5 | 89.7 | 106.7 | 88.3 | 69.5 | 120.3 | 146.9 | 99.9 |
| ID52678 | 116.6 | 177.7 | 108.2 | 94.7 | 97.4 | 72.6 | 98.1 | 71.4 | 93.7 | 63.5 | 85.5 | 71.7 |
| ID52684 | 81.4 | 92.8 | 103.2 | 105.9 | 131.9 | 118.1 | 152.6 | 120.6 | 68.6 | 89.5 | 114.3 | 103.6 |
| ID52685 | 149.1 | 146.4 | 118.9 | 122.2 | 76.8 | 88.8 | 80.2 | 66.3 | 68.2 | 79.2 | 52.4 | 79.0 |
| ID52689 | 63.6 | 62.1 | 80.2 | 62.2 | 74.1 | 128.0 | 133.5 | 171.0 | 119.4 | 147.3 | 104.5 | 229.8 |
| ID52690 | 149.7 | 152.6 | 100.5 | 123.6 | 65.5 | 77.3 | 77.4 | 64.9 | 73.4 | 67.1 | 54.8 | 69.9 |
| ID52691 | 40.4 | 43.1 | 55.6 | 35.9 | 78.5 | 82.9 | 129.5 | 119.5 | 82.5 | 131.9 | 108.1 | 113.5 |
| ID52692 | 94.2 | 169.4 | 112.6 | 84.8 | 120.6 | 133.1 | 88.5 | 87.9 | 73.1 | 104.8 | 103.3 | 111.9 |
| ID52693 | 78.1 | 140.0 | 38.5 | 47.9 | 83.2 | 113.2 | 50.9 | 63.4 | 51.6 | 86.6 | 34.4 | 77.6 |
| ID52694 | 162.1 | 264.9 | 129.0 | 81.1 | 91.4 | 61.0 | 74.9 | 44.0 | 75.2 | 53.9 | 70.5 | 53.8 |
| ID52697 | 66.8 | 28.4 | 52.9 | 43.7 | 83.6 | 98.8 | 90.0 | 98.8 | 107.2 | 137.0 | 73.2 | 114.5 |
| ID52698 | 76.5 | 136.3 | 75.6 | 100.2 | 75.7 | 135.6 | 89.2 | 75.7 | 116.7 | 99.5 | 68.8 | 106.7 |
| ID52699 | 68.3 | 76.8 | 32.2 | 51.5 | 65.9 | 132.8 | 53.0 | 52.2 | 98.2 | 102.2 | 30.9 | 107.5 |
| ID52701 | 87.4 | 187.8 | 65.8 | 91.4 | 41.7 | 196.4 | 81.7 | 32.2 | 97.7 | 65.8 | 11.0 | 80.5 |
| ID52702 | 47.8 | 79.5 | 20.6 | 34.2 | 32.5 | 54.9 | 41.1 | 48.8 | 60.0 | 130.0 | 25.2 | 90.2 |
| ID52703 | 65.2 | 133.9 | 55.5 | 58.4 | 59.9 | 105.5 | 73.1 | 82.1 | 88.0 | 78.5 | 48.7 | 88.5 |
| ID52706 | 56.9 | 97.6 | 49.8 | 50.3 | 86.4 | 100.2 | 89.2 | 69.0 | 96.9 | 127.8 | 54.5 | 99.9 |
| ID66393 | 33.5 | 45.5 | 53.9 | 39.8 | 116.0 | 127.8 | 129.2 | 139.2 | 130.1 | 177.4 | 181.3 | 135.6 |
| ID52709 | 46.2 | 167.5 | 10.2 | 30.5 | 31.8 | 4.2 | 28.9 | 26.5 | 34.8 | 8.2 | 11.4 | 33.4 |
| ID52711 | 34.5 | 121.7 | 20.8 | 47.0 | 15.8 | 157.7 | 36.6 | 27.9 | 94.0 | 69.8 | 9.7 | 61.7 |
| ID52712 | 40.9 | 119.8 | 37.9 | 41.5 | 39.2 | 116.2 | 50.5 | 77.9 | 78.0 | 102.1 | 35.0 | 87.2 |
| ID52714 | 62.3 | 195.7 | 13.9 | 35.5 | 30.6 | 52.2 | 29.9 | 18.4 | 29.3 | 68.1 | 12.6 | 37.6 |
| ID52717 | 30.5 | 129.3 | 14.9 | 34.0 | 23.1 | 97.8 | 32.4 | 40.4 | 130.8 | 73.4 | 16.2 | 58.9 |
| ID52720 | 34.7 | 155.4 | 9.0 | 34.7 | 14.3 | 14.1 | 32.6 | 40.8 | 118.4 | 56.8 | 11.0 | 68.5 |
| ID52721 | 36.3 | 168.1 | 16.1 | 43.3 | 17.7 | 124.8 | 33.8 | 25.9 | 101.1 | 62.1 | 9.4 | 51.3 |
| ID52722 | 93.3 | 670.7 | 141.9 | 264.8 | 86.6 |  | 202.7 | 391.7 | 605.4 |  | 289.9 | 494.7 |
| ID52727 | 33.6 | 116.3 | 14.5 | 30.9 | 21.7 | 80.4 | 32.0 | 63.6 | 104.6 | 89.8 | 15.9 | 73.2 |
| ID52731 | 43.3 | 118.3 | 22.6 | 31.5 | 29.2 | 64.8 | 39.7 | 85.0 | 91.0 | 90.8 | 21.4 | 67.5 |
| ID52760 | 110.8 | 98.4 | 83.2 | 76.5 | 99.7 | 82.8 | 85.6 | 96.6 | 64.4 | 77.8 | 81.5 | 100.2 |
| ID52769 | 83.6 | 104.9 | 86.5 | 71.7 | 103.2 | 80.6 | 94.8 | 97.5 | 66.5 | 72.9 | 75.4 | 93.3 |
| ID52778 | 127.5 | 86.4 | 67.4 | 85.5 | 67.7 | 80.9 | 90.1 | 81.7 | 114.1 | 88.8 | 58.5 | 115.2 |
| ID52819 | 81.6 | 56.0 | 109.0 | 74.7 | 83.5 | 93.0 | 90.1 | 125.9 | 141.8 | 153.7 | 111.4 | 122.6 |
| ID52828 | 150.0 | 102.4 | 103.9 | 133.8 | 140.7 | 61.9 | 90.9 | 123.3 | 80.4 | 79.2 | 125.6 | 101.4 |
| ID52844 | 129.8 | 148.6 | 122.3 | 78.4 | 253.1 | 122.1 | 123.4 | 170.9 | 113.5 | 151.7 | 165.0 | 137.3 |
| ID52859 | 106.9 | 133.1 | 112.5 | 262.0 | 154.1 | 90.8 | 125.5 | 274.2 | 1032.1 | 131.9 | 463.3 | 305.9 |
| ID52871 | 144.3 | 119.4 | 149.3 | 152.1 | 171.9 | 68.6 | 119.9 | 163.0 | 115.4 | 77.7 | 174.4 | 133.1 |
| ID52887 | 82.8 | 198.2 | 74.4 | 78.3 | 107.8 | 89.0 | 140.7 | 69.9 | 34.1 | 86.0 | 52.6 | 79.0 |
| ID66134 | 127.3 | 192.0 | 144.0 | 158.9 | 79.9 | 71.5 | 65.0 | 72.7 | 60.9 | 74.8 | 74.1 | 67.1 |
| ID52929 | 105.4 | 86.3 | 69.9 | 102.8 | 87.4 | 108.3 | 112.0 | 91.3 | 97.9 | 86.7 | 81.1 | 114.8 |
| ID53020 | 266.2 | 393.4 | 75.5 | 80.0 | 107.6 | 87.0 | 72.7 | 82.1 | 125.8 | 98.8 | 77.7 | 108.3 |
| ID53041 | 252.1 | 362.0 | 151.3 | 142.9 | 64.3 | 70.0 | 41.7 | 45.6 | 37.6 | 56.4 | 44.9 | 52.6 |
| ID53066 | 129.1 | 85.7 | 90.8 | 85.1 | 100.2 | 87.9 | 88.7 | 91.4 | 116.7 | 140.3 | 76.7 | 131.3 |
| ID53078 | 103.3 | 93.5 | 109.5 | 95.8 | 99.2 | 85.6 | 85.0 | 89.3 | 118.8 | 122.9 | 73.6 | 118.2 |
| ID53093 | 97.5 | 77.8 | 95.6 | 86.6 | 61.3 | 63.4 | 76.2 | 87.7 | 166.7 | 147.8 | 71.6 | 131.6 |
| ID53103 | 82.1 | 114.6 | 125.4 | 108.7 | 105.5 | 71.7 | 119.5 | 136.5 | 104.5 | 73.3 | 134.3 | 106.5 |
| ID53139 | 83.5 | 103.4 | 112.9 | 109.4 | 102.6 | 75.4 | 104.9 | 133.8 | 97.1 | 82.9 | 126.5 | 102.1 |
| ID53380 | 45.7 |  | 55.5 | 42.8 | 102.9 | 104.2 | 116.1 | 123.1 | 105.3 | 151.5 | 131.3 | 123.5 |
| ID53481 | 122.6 | 44.4 | 42.2 | 65.8 | 72.5 | 95.2 | 98.8 | 115.1 | 158.0 | 133.8 | 88.4 | 97.9 |
| ID53492 | 266.8 | 69.4 | 65.9 | 60.1 | 90.0 | 74.8 | 73.2 | 100.9 | 93.1 | 92.1 | 98.8 | 98.7 |
| ID53503 | 73.9 | 52.9 | 20.3 | 81.7 | 2716.8 | 139.3 | 59.0 | 82.3 | 96.4 | 69.4 | 128.9 | 205.7 |
| ID53560 | 60.8 | 58.6 | 58.9 | 61.0 | 94.4 | 93.0 | 110.3 | 122.0 | 78.4 | 109.6 | 120.0 | 108.5 |
| ID53875 | 79.7 | 75.8 | 84.3 | 81.3 | 91.3 | 91.6 | 93.9 | 88.7 | 115.6 | 105.9 | 102.5 | 103.7 |
| ID53907 | 98.9 | 81.1 | 87.9 | 76.4 | 86.2 | 89.7 | 82.0 | 85.7 | 124.2 | 112.8 | 92.7 | 114.5 |

^a^ Due to limited space, only age-associated spot ID’s (spots highlighted in yellow) are shown on the 2D-DIGE image presented in Figure 1 . Should you require information on the location on 2-D-DIGE Image of other spot ID’s in this table, please contact the corresponding author

**Reference:** [1]	Sikulu MT, Monkman J, Dave KA, Hastie ML, Dale PE, Kitching RL, Killeen GF, Kay BH, Gorman JJ, Hugo L**E**. Proteomic changes occurring in the malaria mosquitoes Anopheles gambiae and Anopheles stephensi during aging. Journal of Proteomi*c*s 2015; 126:234-44.
