# Supplementary material for: Mass spectrometry identification of age-associated proteins from the malaria mosquitoes Anopheles gambiae s.s. and Anopheles stephensi
Source: Data Brief. 2015 Jul 16;4:461–7. doi: 10.1016/j.dib.2015.07.007 (PMC4534588; doi:10.1016/j.dib.2015.07.007)
Supplement: Supplementary file 1 — Supplementary material: Supplementary Table 1: Normalised spot volume data for all spots detected on 2D-DIGE images of cohort 1 of A. gambiae. Spots highlighted in yellow were found to be age-associated. Supplementary Table 2: Normalised spot volume data for all spots detected on 2D-DIGE images of cohort 2 of A. gambiae. Spots highlighted in yellow were found to be age-associated. Supplementary Table 3: Normalised spot volume data for all spots detected on 2D-DIGE images of cohort 1 of A. stephensi. Spots highlighted in yellow were found to be age-associated. Supplementary Table 4: Normalised spot volume data for all spots detected on 2D-DIGE images of cohort 2 of A. stephensi. Spots highlighted in yellow were found to be age-associated. External repository data: The mass spectrometry proteomics data have been deposited to the ProteomeXchange Consortium [8] via the PRIDE partner repository with the dataset identifier PXD002153 and 10.6019/PXD002153. [file mmc1.zip › Supplimentary file/Supplementary Table 2.doc]

**Table 2**

| **Spot ID^a^** | **Normalized Vol. D1b** | **Normalized Vol. D1c** | **Normalized Vol. D1d** | **Normalized Vol. D9a** | **Normalized Vol. D9b** | **Normalized Vol. D9c** | **Normalized Vol. 9d.** | **Normalized Vol. D17a** | **Normalized Vol. D17b** | **Normalized Vol. D17c** | **Normalized Vol. D17d** |
| --- | --- | --- | --- | --- | --- | --- | --- | --- | --- | --- | --- |
| ID65843 | 546.9 | 1167.2 | 1054.9 |  | 274.4 | 1110.1 |  | 755.1 | 313.7 | 709.5 |  |
| ID65959 | 2.4 |  | 96.2 | 4.4 |  |  |  | 37.3 |  | 19.2 |  |
| ID65965 | 84.7 | 66.8 | 73.1 | 83.4 | 34.2 | 66.8 | 35.9 | 69.4 | 57.7 | 66.9 | 45.3 |
| ID65986 | 36.7 | 59.6 | 62.6 | 102.2 | 109.2 | 93.3 | 109.4 | 101.3 | 144.1 | 137.0 | 102.5 |
| ID65987 | 28.3 | 55.0 | 71.6 | 122.2 | 126.6 | 110.5 | 128.4 | 112.2 | 151.0 | 165.5 | 130.7 |
| ID66001 | 106.2 | 92.1 | 86.8 | 80.0 | 111.1 | 86.6 | 87.3 | 102.1 | 227.0 | 111.0 | 80.5 |
| ID66002 | 159.5 | 149.4 | 106.2 | 76.0 | 72.2 | 71.6 | 76.1 | 72.5 | 75.2 | 72.0 | 73.2 |
| ID66004 | 135.8 | 118.7 | 88.5 | 129.5 | 82.1 | 91.8 | 122.8 | 94.2 | 133.3 | 118.2 | 82.9 |
| ID66005 | 142.5 | 132.9 | 90.3 | 85.5 | 81.6 | 90.1 | 87.2 | 82.9 | 89.7 | 81.9 | 69.4 |
| ID66006 | 143.9 | 130.6 | 94.7 | 121.9 | 69.3 | 91.0 | 113.1 | 84.7 | 96.3 | 116.4 | 82.8 |
| ID1073 | 120.7 | 116.5 | 125.2 | 85.0 | 85.9 | 85.7 | 87.2 | 74.4 | 74.6 | 71.4 | 79.4 |
| ID66007 | 186.5 | 148.4 | 92.4 | 79.2 | 75.8 | 84.6 | 90.2 | 97.0 | 79.6 | 75.3 | 71.3 |
| ID66011 | 164.7 | 149.2 | 86.8 | 81.3 | 69.6 | 86.9 | 99.8 | 84.3 | 82.6 | 86.0 | 74.5 |
| ID66012 | 175.9 | 153.7 | 113.3 | 72.6 | 74.6 | 72.5 | 88.1 | 82.3 | 89.5 | 85.8 | 77.5 |
| ID66028 | 124.1 | 136.7 | 83.6 | 64.4 | 64.8 | 86.5 | 110.8 | 77.0 | 84.2 | 114.8 | 74.3 |
| ID66036 | 133.6 | 123.8 | 76.5 | 73.3 | 32.5 | 72.0 | 77.2 | 65.5 | 121.9 | 104.4 | 63.0 |
| ID66037 | 187.5 | 131.2 | 87.6 | 82.3 | 79.1 | 78.2 | 81.6 | 99.8 | 73.7 | 80.3 | 73.1 |
| ID66050 | 201.6 | 157.2 | 162.0 | 31.3 | 83.1 | 98.5 | 66.5 | 126.6 | 62.8 | 46.3 | 66.9 |
| ID66051 | 146.0 | 151.1 | 154.7 | 46.7 | 101.5 | 105.9 | 72.4 | 140.6 | 84.6 | 59.1 | 73.8 |
| ID66052 | 98.1 | 91.8 | 109.7 | 114.7 | 63.0 | 56.6 | 67.4 | 83.0 | 91.4 | 102.1 | 61.5 |
| ID66053 | 282.1 | 191.3 | 170.5 | 34.9 | 58.5 | 74.8 | 48.4 | 91.5 | 50.3 | 45.8 | 53.3 |
| ID1130 | 118.3 | 91.7 | 118.5 | 111.6 | 86.7 | 94.6 | 115.7 | 82.3 | 83.4 | 58.5 | 60.6 |
| ID66056 | 142.5 | 127.2 | 82.0 | 104.0 | 83.9 | 88.6 | 97.8 | 95.4 | 103.9 | 96.2 | 81.7 |
| ID66063 | 159.2 | 122.4 | 80.6 | 92.0 | 79.7 | 84.5 | 94.9 | 101.0 | 115.1 | 97.2 | 70.0 |
| ID66064 | 138.2 | 122.1 | 81.6 | 95.9 | 79.6 | 86.0 | 98.1 | 90.1 | 109.1 | 97.6 | 74.6 |
| ID66065 | 140.0 | 124.0 | 85.7 | 86.6 | 75.5 | 87.5 | 95.2 | 83.3 | 100.8 | 93.6 | 71.9 |
| ID66068 | 93.3 | 23.7 | 80.0 | 432.8 | 72.8 | 11.1 | 29.4 | 36.7 | 45.9 | 49.5 | 25.7 |
| ID66069 | 268.8 | 235.3 | 139.9 | 55.4 | 33.1 | 36.1 | 35.1 | 49.9 | 42.5 | 49.4 | 39.8 |
| ID66070 | 154.9 | 154.4 | 162.0 | 40.9 | 81.8 | 108.1 | 70.0 | 144.4 | 67.5 | 55.0 | 72.2 |
| ID66071 | 126.6 | 113.1 | 80.2 | 89.4 | 70.9 | 72.3 | 72.3 | 97.5 | 154.3 | 107.0 | 70.2 |
| ID66072 | 139.3 | 114.3 | 83.3 | 91.2 | 74.5 | 74.7 | 75.9 | 111.2 | 146.6 | 113.5 | 71.5 |
| ID66073 | 109.0 | 109.8 | 83.7 | 97.9 | 76.9 | 78.7 | 78.1 | 106.1 | 176.0 | 134.0 | 72.8 |
| ID66077 | 127.6 | 119.2 | 111.7 | 95.8 | 89.2 | 85.1 | 114.7 | 112.7 | 109.8 | 107.4 | 81.2 |
| ID66078 | 146.0 | 151.0 | 95.8 | 76.9 | 58.9 | 70.6 | 68.6 | 80.5 | 122.0 | 82.6 | 70.3 |
| ID66079 | 134.0 | 130.7 | 90.8 | 78.6 | 67.0 | 73.3 | 70.7 | 97.2 | 132.9 | 99.7 | 69.0 |
| ID66087 | 168.5 | 172.5 | 128.2 | 83.3 | 60.6 | 78.6 | 67.7 | 84.5 | 78.8 | 94.2 | 73.6 |
| ID66088 | 191.2 | 171.1 | 128.1 | 78.9 | 59.7 | 72.6 | 63.6 | 89.8 | 85.9 | 93.4 | 69.9 |
| ID66089 | 170.8 | 146.8 | 109.2 | 71.3 | 83.3 | 86.2 | 103.9 | 90.2 | 86.2 | 87.7 | 76.7 |
| ID66091 | 188.0 | 153.0 | 129.6 | 78.2 | 62.1 | 69.8 | 66.0 | 71.7 | 78.9 | 73.6 | 56.7 |
| ID66093 | 163.8 | 142.9 | 131.1 | 78.8 | 72.4 | 72.3 | 80.8 | 91.3 | 93.5 | 84.7 | 62.8 |
| ID1324 | 123.1 | 113.5 | 107.6 | 98.0 | 92.0 | 77.6 | 91.6 | 81.8 | 80.5 | 77.8 | 83.6 |
| ID66094 | 79.6 | 93.1 | 76.2 | 90.0 | 112.3 | 99.3 | 156.3 | 75.7 | 117.0 | 118.7 | 91.6 |
| ID66097 | 145.5 | 128.3 | 85.8 | 79.1 | 92.9 | 87.9 | 139.2 | 92.1 | 96.6 | 103.9 | 86.8 |
| ID66098 | 198.6 | 161.5 | 101.5 | 82.1 | 70.1 | 78.4 | 78.2 | 93.1 | 86.4 | 82.9 | 71.1 |
| ID66100 | 214.3 | 246.7 | 175.7 | 57.5 | 52.2 | 51.2 | 69.5 | 61.0 | 62.3 | 76.2 | 65.7 |
| ID66106 | 173.0 | 156.7 | 118.1 | 89.3 | 76.9 | 79.9 | 87.2 | 87.2 | 99.2 | 83.4 | 72.2 |
| ID66107 | 182.2 | 178.8 | 118.1 | 71.4 | 67.8 | 66.7 | 99.3 | 75.9 | 89.7 | 86.9 | 78.3 |
| ID66109 | 200.5 | 180.7 | 112.3 | 82.7 | 71.7 | 74.3 | 73.6 | 90.4 | 92.2 | 70.4 | 67.8 |
| ID66110 | 214.3 | 170.2 | 108.0 | 80.7 | 69.5 | 76.4 | 69.7 | 92.9 | 86.9 | 77.3 | 66.4 |
| ID66111 | 142.5 | 131.9 | 89.7 | 99.8 | 91.0 | 110.3 | 120.6 | 93.1 | 128.1 | 99.1 | 96.7 |
| ID66112 | 201.8 | 163.4 | 123.1 | 82.5 | 74.8 | 75.0 | 96.9 | 86.2 | 92.3 | 85.4 | 77.2 |
| ID66113 | 159.2 | 162.6 | 135.8 | 71.7 | 75.4 | 69.5 | 113.4 | 83.2 | 104.9 | 100.7 | 87.9 |
| ID66116 | 179.4 | 174.1 | 134.7 | 81.6 | 58.9 | 80.8 | 68.0 | 105.2 | 84.1 | 91.1 | 75.7 |
| ID66119 | 198.3 | 255.7 | 183.8 | 40.4 | 46.6 | 57.0 | 44.8 | 58.2 | 48.6 | 56.6 | 56.4 |
| ID66120 | 157.7 | 188.2 | 172.1 | 69.0 | 70.1 | 80.0 | 67.1 | 96.3 | 74.2 | 88.6 | 80.0 |
| ID66121 | 154.1 | 183.3 | 153.3 | 69.1 | 79.6 | 69.9 | 120.8 | 85.6 | 115.9 | 109.7 | 88.3 |
| ID66124 | 150.4 | 167.6 | 145.7 | 93.7 | 71.3 | 87.2 | 80.2 | 103.6 | 92.2 | 94.7 | 100.8 |
| ID66125 | 168.9 | 195.6 | 184.2 | 61.7 | 81.3 | 65.3 | 109.1 | 90.2 | 111.4 | 103.7 | 81.1 |
| ID66131 | 177.4 | 178.3 | 175.9 | 72.5 | 65.1 | 86.8 | 65.0 | 115.8 | 73.5 | 84.0 | 73.5 |
| ID66132 | 287.1 | 181.9 | 123.1 | 74.0 | 63.0 | 67.5 | 64.2 | 101.6 | 87.4 | 68.0 | 64.7 |
| ID66134 | 245.0 | 169.5 | 118.7 | 66.9 | 54.4 | 61.0 | 63.8 | 89.3 | 74.3 | 76.9 | 65.7 |
| ID66135 | 347.0 | 197.3 | 131.0 | 50.1 | 51.4 | 48.2 | 46.0 | 72.2 | 63.7 | 49.6 | 46.2 |
| ID66136 | 200.5 | 179.0 | 126.2 | 72.7 | 64.5 | 70.4 | 56.4 | 78.0 | 101.8 | 82.2 | 66.9 |
| ID66137 | 231.3 | 217.7 | 178.9 | 37.9 | 54.6 | 49.8 | 39.1 | 47.7 | 56.1 | 38.0 | 66.7 |
| ID66139 | 216.9 | 173.1 | 123.7 | 75.2 | 63.4 | 67.1 | 57.8 | 82.0 | 98.2 | 79.2 | 71.1 |
| ID66140 | 362.8 | 205.7 | 128.6 | 64.2 | 62.4 | 73.2 | 51.7 | 87.1 | 71.3 | 52.5 | 51.3 |
| ID66141 | 191.2 | 155.9 | 108.7 | 76.0 | 69.5 | 68.5 | 60.4 | 90.0 | 140.8 | 97.5 | 84.7 |
| ID66142 | 134.2 | 124.1 | 100.9 | 80.3 | 85.1 | 72.7 | 76.8 | 97.9 | 210.8 | 140.9 | 126.4 |
| ID66143 | 122.0 | 104.6 | 85.0 | 83.2 | 93.4 | 75.6 | 83.6 | 114.2 | 242.6 | 188.0 | 189.1 |
| ID66148 | 180.8 | 176.4 | 126.5 | 78.4 | 79.8 | 86.8 | 62.0 | 96.9 | 83.3 | 75.8 | 63.4 |
| ID66151 | 149.3 | 139.5 | 109.9 | 89.8 | 82.4 | 89.5 | 86.7 | 90.8 | 99.9 | 105.5 | 87.0 |
| ID66152 | 174.4 | 122.3 | 105.3 | 90.8 | 84.3 | 82.4 | 71.2 | 114.0 | 167.3 | 112.3 | 112.3 |
| ID66155 | 155.1 | 136.6 | 105.0 | 91.2 | 79.2 | 75.8 | 80.7 | 84.9 | 110.1 | 108.1 | 78.2 |
| ID66156 | 217.9 | 163.3 | 127.4 | 74.0 | 64.2 | 71.0 | 56.0 | 91.2 | 125.6 | 95.3 | 77.0 |
| ID66158 | 257.9 | 194.6 | 130.0 | 81.1 | 52.9 | 58.0 | 54.2 | 64.0 | 61.5 | 52.4 | 52.2 |
| ID66159 | 165.3 | 132.4 | 98.2 | 85.7 | 73.7 | 87.1 | 82.6 | 94.4 | 105.8 | 98.8 | 80.0 |
| ID66160 | 311.4 | 126.8 | 103.9 | 104.5 | 56.1 | 58.5 | 48.2 | 52.1 | 69.2 | 75.9 | 40.5 |
| ID1452 | 149.2 | 158.9 | 125.0 | 82.6 | 141.9 | 63.2 | 73.2 | 48.2 | 29.8 | 28.2 | 48.0 |
| ID66164 | 199.5 | 150.3 | 117.6 | 85.8 | 64.8 | 104.8 | 81.7 | 95.2 | 114.9 | 99.3 | 85.0 |
| ID66168 | 156.1 | 24.7 | 37.4 | 89.6 | 130.1 | 78.3 | 91.4 | 115.4 | 117.4 | 130.6 | 99.4 |
| ID66170 | 267.0 | 205.8 | 152.8 | 46.6 | 57.5 | 92.0 | 46.8 | 100.4 | 47.0 | 38.1 | 52.1 |
| ID66172 | 297.6 | 179.5 | 125.1 | 72.6 | 52.0 | 56.2 | 55.8 | 62.6 | 73.2 | 60.0 | 52.9 |
| ID66173 | 422.1 | 197.3 | 141.8 | 40.4 | 35.5 | 56.0 | 36.5 | 68.7 | 35.5 | 24.5 | 34.2 |
| ID66174 | 293.2 | 174.5 | 151.6 | 54.0 | 56.0 | 85.7 | 49.3 | 96.3 | 52.8 | 37.2 | 49.1 |
| ID66175 | 213.4 | 150.5 | 155.3 | 53.5 | 63.0 | 100.9 | 54.2 | 110.7 | 52.7 | 39.3 | 56.1 |
| ID66176 | 265.0 | 165.7 | 111.5 | 73.8 | 60.2 | 77.1 | 67.7 | 130.5 | 131.1 | 115.6 | 82.7 |
| ID66177 | 382.5 | 222.0 | 99.5 | 54.8 | 43.0 | 47.3 | 39.3 | 56.8 | 47.3 | 47.4 | 37.3 |
| ID66179 | 315.1 | 205.8 | 111.7 | 64.5 | 66.4 | 61.1 | 51.5 | 76.6 | 68.3 | 75.6 | 54.5 |
| ID66180 | 41.9 | 46.0 | 55.7 | 131.8 | 113.1 | 110.2 | 154.0 | 89.3 | 124.5 | 129.8 | 103.2 |
| ID66181 | 203.5 | 112.8 | 178.3 | 131.2 | 190.0 | 97.9 | 137.0 | 122.9 | 236.8 | 314.1 | 91.9 |
| ID66182 | 477.8 | 219.6 | 95.7 | 54.1 | 32.9 | 38.8 | 38.0 | 57.7 | 43.2 | 36.6 | 30.2 |
| ID66183 | 63.8 | 66.6 | 79.6 | 154.8 | 132.3 | 141.3 | 189.6 | 140.6 | 143.8 | 143.0 | 113.3 |
| ID66184 | 74.8 | 85.3 | 89.0 | 120.2 | 114.6 | 98.6 | 141.0 | 98.1 | 116.8 | 136.1 | 106.7 |
| ID66185 | 39.3 | 48.6 | 59.2 | 133.8 | 125.3 | 120.7 | 154.9 | 103.7 | 143.3 | 154.1 | 106.7 |
| ID66186 | 81.8 | 89.2 | 89.2 | 97.1 | 97.3 | 86.0 | 120.8 | 96.7 | 119.3 | 133.9 | 94.3 |
| ID66189 | 220.8 | 145.2 | 164.6 | 59.8 | 91.0 | 174.7 | 67.9 | 236.9 | 64.5 | 53.3 | 65.4 |
| ID66190 | 105.9 | 67.5 | 70.1 | 145.3 | 108.0 | 107.2 | 181.4 | 87.9 | 137.6 | 117.3 | 116.1 |
| ID66191 | 105.2 | 98.0 | 71.4 | 123.4 | 97.8 | 86.8 | 123.1 | 91.3 | 108.0 | 129.1 | 96.7 |
| ID66192 | 106.8 | 127.8 | 78.8 | 94.4 | 87.3 | 72.6 | 114.3 | 75.3 | 103.1 | 111.1 | 87.4 |
| ID66194 | 65.8 | 77.1 | 93.0 | 116.8 | 116.3 | 112.4 | 117.9 | 120.4 | 129.4 | 132.6 | 115.5 |
| ID66195 | 116.3 | 141.6 | 112.3 | 106.7 | 95.4 | 88.1 | 131.8 | 102.2 | 110.7 | 126.7 | 94.1 |
| ID66196 | 47.4 | 60.4 | 74.9 | 134.9 | 147.2 | 123.6 | 153.3 | 120.8 | 150.6 | 151.8 | 105.4 |
| ID66198 | 82.1 | 82.0 | 44.7 | 131.4 | 103.0 | 102.1 | 107.3 | 109.0 | 140.8 | 148.3 | 97.7 |
| ID66199 | 80.9 | 86.7 | 73.0 | 90.5 | 95.7 | 77.3 | 131.1 | 96.2 | 129.6 | 144.6 | 112.2 |
| ID539 | 57.1 | 46.5 | 53.8 | 107.6 | 140.7 | 105.5 | 86.5 | 137.6 | 197.7 | 190.6 | 133.2 |
| ID66200 | 82.6 | 86.3 | 75.5 | 111.6 | 100.8 | 103.4 | 107.8 | 109.9 | 125.0 | 130.0 | 99.4 |
| ID66201 | 101.2 | 87.9 | 70.8 | 102.9 | 99.6 | 104.3 | 134.7 | 107.7 | 130.7 | 115.4 | 86.3 |
| ID66203 | 23.9 | 40.0 | 84.6 | 141.0 | 123.5 | 162.8 | 188.4 | 140.8 | 148.4 | 162.7 | 196.8 |
| ID66204 | 98.7 | 103.2 | 88.0 | 138.3 | 109.9 | 108.5 | 138.9 | 120.5 | 127.4 | 134.4 | 95.3 |
| ID66205 | 97.0 | 109.9 | 60.6 | 119.8 | 115.8 | 92.4 | 102.6 | 136.1 | 167.8 | 198.5 | 107.2 |
| ID66206 | 76.9 | 65.7 | 67.4 | 126.3 | 105.8 | 111.3 | 123.7 | 100.7 | 129.3 | 125.5 | 101.9 |
| ID66207 | 52.9 | 46.4 | 65.2 | 145.3 | 134.2 | 126.1 | 135.0 | 116.8 | 153.5 | 140.8 | 119.2 |
| ID66208 | 56.1 | 70.5 | 58.2 | 135.8 | 93.7 | 87.9 | 117.1 | 91.6 | 119.8 | 115.0 | 87.1 |
| ID66209 | 88.4 | 93.8 | 82.6 | 156.1 | 128.0 | 112.3 | 146.9 | 131.5 | 159.5 | 148.9 | 104.7 |
| ID66210 | 56.9 | 72.1 | 67.9 | 159.0 | 111.5 | 119.9 | 147.3 | 141.2 | 143.8 | 148.4 | 113.0 |
| ID66211 | 99.4 | 86.6 | 50.4 | 110.2 | 92.6 | 70.2 | 150.4 | 89.5 | 116.5 | 123.9 | 97.0 |
| ID66212 | 68.8 | 73.9 | 72.7 | 132.7 | 103.1 | 88.1 | 116.0 | 98.1 | 92.3 | 112.2 | 86.6 |
| ID66213 | 36.9 | 68.4 | 158.1 | 81.5 | 92.0 | 98.6 | 79.7 | 170.4 | 205.8 | 194.7 | 114.7 |
| ID66214 | 86.9 | 79.9 | 83.9 | 119.2 | 108.3 | 104.0 | 118.1 | 108.1 | 130.8 | 130.4 | 104.5 |
| ID66215 | 83.7 | 86.7 | 64.0 | 142.8 | 95.4 | 90.9 | 117.7 | 101.8 | 118.2 | 109.9 | 86.6 |
| ID66216 | 49.4 | 58.5 | 53.8 | 139.9 | 98.7 | 95.6 | 129.3 | 108.4 | 132.1 | 132.3 | 104.7 |
| ID66217 | 83.7 | 63.0 | 64.6 | 173.7 | 111.1 | 119.0 | 177.7 | 165.3 | 122.1 | 123.1 | 98.2 |
| ID66218 | 101.8 | 82.2 | 72.1 | 162.6 | 112.6 | 104.4 | 136.8 | 134.4 | 140.4 | 116.0 | 93.3 |
| ID66219 | 88.3 | 75.6 | 69.8 | 205.7 | 123.8 | 137.5 | 178.6 | 167.2 | 141.6 | 137.0 | 104.9 |
| ID66220 | 247.2 | 157.8 | 84.1 | 160.0 | 61.6 | 106.6 | 120.8 | 111.9 | 70.5 | 42.4 | 121.7 |
| ID66221 | 60.0 | 91.7 | 84.6 | 126.5 | 95.7 | 107.1 | 113.3 | 97.1 | 132.6 | 117.4 | 92.7 |
| ID66222 | 45.3 | 63.2 | 76.1 | 128.8 | 106.0 | 110.5 | 130.4 | 97.8 | 112.3 | 105.2 | 111.5 |
| ID66223 | 67.1 | 70.4 | 66.6 | 174.2 | 162.9 | 133.9 | 200.7 | 168.8 | 161.5 | 134.3 | 133.7 |
| ID66224 | 95.7 | 73.1 | 81.3 | 127.0 | 117.7 | 113.6 | 112.5 | 108.2 | 157.0 | 125.2 | 107.3 |
| ID66226 | 56.1 | 55.1 | 77.0 | 141.0 | 116.9 | 120.3 | 128.3 | 104.3 | 122.7 | 110.9 | 113.9 |
| ID66228 | 121.0 | 102.9 | 98.5 | 105.0 | 96.0 | 85.2 | 118.1 | 110.3 | 121.0 | 132.2 | 92.6 |
| ID66229 | 76.2 | 95.3 | 92.8 | 154.6 | 114.3 | 103.5 | 131.9 | 129.6 | 124.0 | 129.4 | 104.6 |
| ID66232 | 149.5 | 124.0 | 155.0 | 76.5 | 98.7 | 107.2 | 65.1 | 86.0 | 94.1 | 62.6 | 98.9 |
| ID66233 | 129.2 | 98.0 | 96.5 | 100.9 | 89.9 | 81.6 | 119.9 | 111.9 | 115.2 | 139.6 | 97.3 |
| ID66236 | 93.4 | 100.2 | 91.4 | 180.3 | 132.9 | 130.1 | 138.2 | 120.5 | 164.7 | 121.6 | 113.0 |
| ID66237 | 38.9 | 46.3 | 65.7 | 130.5 | 117.6 | 107.5 | 124.0 | 107.8 | 141.5 | 138.4 | 115.6 |
| ID66238 | 140.5 | 106.6 | 100.6 | 113.1 | 65.9 | 78.8 | 84.3 | 82.6 | 94.0 | 96.2 | 71.6 |
| ID66239 | 80.1 | 95.9 | 101.7 | 99.1 | 96.6 | 88.6 | 102.2 | 97.7 | 115.9 | 117.6 | 91.5 |
| ID66240 | 49.1 | 36.5 | 64.1 | 140.7 | 122.5 | 119.1 | 132.9 | 121.0 | 162.4 | 145.7 | 129.1 |
| ID66241 | 55.3 | 27.2 | 49.5 | 149.4 | 106.3 | 119.2 | 141.4 | 102.9 | 127.7 | 128.5 | 117.0 |
| ID66242 | 137.8 | 105.7 | 89.9 | 130.6 | 102.0 | 104.7 | 92.9 | 104.4 | 121.1 | 126.9 | 85.2 |
| ID66243 | 11.5 | 6.2 | 25.3 | 141.5 | 107.9 | 153.2 | 155.6 | 125.2 | 144.5 | 161.5 | 144.2 |
| ID66244 | 46.7 | 64.2 | 51.5 | 136.5 | 93.5 | 102.9 | 106.4 | 139.3 | 181.8 | 184.3 | 125.8 |
| ID66245 | 117.1 | 128.0 | 116.7 | 74.3 | 96.4 | 78.6 | 101.2 | 99.7 | 92.6 | 109.6 | 92.8 |
| ID66246 | 47.9 | 67.9 | 60.6 | 109.3 | 83.1 | 84.8 | 113.7 | 79.6 | 114.4 | 118.3 | 87.4 |
| ID66247 | 156.8 | 153.4 | 91.4 | 147.7 | 94.0 | 107.3 | 141.3 | 119.3 | 119.9 | 86.8 | 120.5 |
| ID66248 | 61.9 | 70.6 | 64.6 | 118.8 | 97.0 | 93.2 | 119.0 | 93.1 | 126.5 | 125.7 | 94.5 |
| ID66250 | 48.6 | 57.9 | 60.7 | 137.5 | 105.5 | 87.7 | 105.5 | 130.3 | 193.9 | 185.0 | 137.2 |
| ID66253 | 89.4 | 76.8 | 63.8 | 125.2 | 94.5 | 87.8 | 123.5 | 87.6 | 115.5 | 120.8 | 98.3 |
| ID66255 | 174.7 | 171.2 | 96.0 | 79.5 | 90.5 | 67.8 | 94.4 | 86.3 | 93.6 | 100.1 | 84.7 |
| ID66256 | 105.4 | 104.8 | 145.2 | 104.0 | 107.3 | 123.5 | 113.9 | 171.5 | 138.7 | 138.8 | 103.4 |
| ID66257 | 49.5 | 55.3 | 59.8 | 124.6 | 104.1 | 97.9 | 136.3 | 99.9 | 133.0 | 141.8 | 105.5 |
| ID66258 | 44.0 | 97.3 | 80.6 | 101.4 | 74.6 | 79.4 | 94.1 | 80.4 | 100.0 | 90.3 | 89.2 |
| ID66259 | 65.4 | 78.4 | 63.0 | 122.4 | 91.8 | 86.4 | 102.8 | 98.6 | 134.7 | 141.1 | 126.2 |
| ID66260 | 32.8 | 60.3 | 52.2 | 106.1 | 82.4 | 78.3 | 98.3 | 69.4 | 109.5 | 111.4 | 94.2 |
| ID66261 | 37.3 | 42.7 | 52.9 | 113.3 | 98.5 | 83.7 | 110.2 | 109.0 | 133.3 | 132.9 | 94.3 |
| ID66264 | 50.4 | 59.8 | 62.5 | 118.8 | 99.4 | 90.9 | 122.4 | 86.9 | 113.8 | 119.3 | 96.5 |
| ID66265 | 57.0 | 64.1 | 63.1 | 115.8 | 94.8 | 88.0 | 118.3 | 89.5 | 112.2 | 113.9 | 95.1 |
| ID66266 | 52.8 | 60.7 | 55.1 | 105.5 | 87.5 | 81.8 | 107.7 | 96.7 | 112.0 | 113.7 | 88.5 |
| ID66267 | 48.8 | 63.5 | 65.7 | 98.7 | 85.0 | 81.7 | 111.5 | 70.0 | 104.7 | 109.2 | 89.6 |
| ID66269 | 65.2 | 47.3 | 73.7 | 113.3 | 110.6 | 117.7 | 118.8 | 115.8 | 131.8 | 128.0 | 124.9 |
| ID66270 | 55.4 | 71.3 | 76.0 | 115.2 | 83.2 | 89.4 | 122.0 | 78.2 | 105.6 | 112.7 | 96.4 |
| ID66272 | 53.6 | 55.2 | 61.8 | 122.3 | 108.6 | 98.5 | 134.5 | 115.7 | 127.8 | 143.2 | 114.4 |
| ID66273 | 43.0 | 54.6 | 66.4 | 128.8 | 120.8 | 101.7 | 148.6 | 117.6 | 142.0 | 160.3 | 128.3 |
| ID66275 | 169.0 | 115.3 | 98.0 | 161.9 | 124.6 | 103.2 | 106.9 | 155.4 | 155.7 | 201.4 | 101.8 |
| ID66278 | 72.6 | 24.5 | 25.0 | 129.9 | 125.7 | 119.9 | 108.6 | 167.3 | 179.3 | 172.4 | 120.0 |
| ID66279 | 49.6 | 65.3 | 68.8 | 132.4 | 116.4 | 97.9 | 136.5 | 115.2 | 137.0 | 152.2 | 118.5 |
| ID66280 | 81.7 | 80.8 | 79.1 | 146.8 | 132.2 | 108.7 | 144.2 | 128.8 | 163.3 | 165.5 | 122.4 |
| ID66282 | 108.8 | 122.1 | 97.1 | 78.8 | 94.1 | 76.7 | 111.5 | 100.3 | 89.5 | 113.5 | 95.0 |
| ID66283 | 92.6 | 55.0 | 36.9 | 129.7 | 99.5 | 107.2 | 87.6 | 164.5 | 170.3 | 165.5 | 101.3 |
| ID66284 | 111.3 | 147.3 | 151.6 | 59.7 | 93.9 | 73.0 | 110.0 | 98.9 | 81.0 | 108.1 | 91.6 |
| ID66285 | 125.0 | 136.1 | 149.3 | 77.3 | 100.3 | 83.4 | 114.0 | 125.3 | 91.7 | 119.9 | 90.7 |
| ID66286 | 82.9 | 100.9 | 74.0 | 141.7 | 96.3 | 97.6 | 119.5 | 102.2 | 130.2 | 127.1 | 87.9 |
| ID66287 | 204.1 | 188.5 | 121.8 | 207.1 | 64.9 | 81.8 | 162.6 | 91.1 | 90.4 | 85.7 | 135.8 |
| ID66291 | 47.7 | 42.4 | 101.3 | 78.7 | 127.9 | 120.6 | 112.0 | 121.8 | 87.6 | 87.6 | 108.8 |
| ID66292 | 70.0 | 106.5 | 98.3 | 120.5 | 107.5 | 93.9 | 119.5 | 116.3 | 151.0 | 144.8 | 97.4 |
| ID66293 | 68.5 | 83.1 | 94.0 | 118.0 | 118.3 | 108.4 | 116.6 | 121.3 | 111.8 | 131.9 | 113.2 |
| ID66294 | 172.0 | 123.3 | 136.8 | 107.2 | 95.2 | 110.9 | 139.6 | 102.4 | 103.5 | 81.1 | 82.1 |
| ID66295 | 161.3 | 147.5 | 126.0 | 88.1 | 75.6 | 69.3 | 100.8 | 71.5 | 88.4 | 76.8 | 63.0 |
| ID66297 | 95.4 | 108.6 | 96.8 | 113.2 | 95.1 | 87.4 | 119.2 | 81.9 | 105.1 | 95.9 | 81.2 |
| ID66298 | 80.8 | 84.7 | 85.9 | 138.9 | 106.6 | 106.0 | 134.6 | 90.6 | 113.6 | 100.6 | 91.7 |
| ID66299 | 52.3 | 57.8 | 70.0 | 135.6 | 116.6 | 115.3 | 147.7 | 101.9 | 122.3 | 118.2 | 112.0 |
| ID66300 | 55.6 | 35.1 | 79.5 | 86.8 | 159.5 | 152.2 | 114.0 | 118.2 | 133.2 | 85.4 | 156.8 |
| ID66301 | 55.8 | 58.4 | 68.0 | 128.6 | 106.1 | 103.4 | 125.4 | 107.6 | 120.5 | 118.5 | 103.0 |
| ID66303 | 60.1 | 86.3 | 88.4 | 121.1 | 103.0 | 103.4 | 134.5 | 97.1 | 127.1 | 117.0 | 105.3 |
| ID66304 | 60.4 | 61.8 | 83.3 | 120.1 | 116.0 | 103.3 | 129.6 | 114.5 | 135.7 | 141.1 | 110.0 |
| ID66308 | 134.5 | 74.7 | 79.9 | 101.5 | 118.9 | 110.0 | 90.3 | 91.3 | 90.1 | 73.8 | 92.9 |
| ID66309 | 105.1 | 85.3 | 68.2 | 114.3 | 118.5 | 105.5 | 92.1 | 112.5 | 121.5 | 97.1 | 103.1 |
| ID66310 | 80.4 | 63.2 | 71.3 | 133.7 | 120.7 | 109.8 | 115.9 | 109.2 | 121.7 | 100.0 | 101.2 |
| ID66311 | 49.9 | 47.5 | 60.6 | 139.4 | 111.9 | 118.1 | 134.5 | 110.9 | 131.7 | 116.3 | 116.2 |
| ID66314 | 58.1 | 56.4 | 68.1 | 131.8 | 107.2 | 118.2 | 119.5 | 104.5 | 116.4 | 104.2 | 107.4 |
| ID66315 | 101.9 | 123.3 | 107.1 | 90.9 | 90.3 | 88.8 | 84.4 | 94.7 | 91.6 | 105.3 | 89.8 |
| ID66316 | 57.9 | 68.5 | 68.4 | 122.7 | 96.3 | 105.8 | 104.9 | 98.6 | 120.7 | 104.4 | 93.3 |
| ID66317 | 63.9 | 82.6 | 69.3 | 106.8 | 96.2 | 111.5 | 90.8 | 93.7 | 152.2 | 130.3 | 127.1 |
| ID66319 | 125.1 | 97.1 | 107.1 | 97.7 | 89.3 | 86.6 | 83.6 | 101.3 | 87.8 | 100.2 | 82.0 |
| ID66320 | 71.5 | 66.0 | 72.5 | 110.6 | 89.9 | 97.1 | 99.5 | 93.7 | 114.3 | 108.9 | 100.2 |
| ID66321 | 51.4 | 57.3 | 61.1 | 131.6 | 105.0 | 113.6 | 107.8 | 119.0 | 147.2 | 110.7 | 92.8 |
| ID66322 | 79.6 | 83.8 | 86.7 | 112.7 | 74.8 | 92.2 | 106.1 | 107.2 | 133.0 | 124.8 | 118.3 |
| ID66324 | 128.3 | 97.1 | 83.9 | 110.1 | 116.6 | 104.2 | 91.1 | 107.6 | 111.0 | 86.9 | 95.2 |
| ID66325 | 68.4 | 61.7 | 86.6 | 105.4 | 122.5 | 112.1 | 120.7 | 111.2 | 118.3 | 128.6 | 118.2 |
| ID66326 | 65.5 | 69.6 | 74.9 | 126.4 | 92.5 | 114.1 | 144.2 | 105.9 | 113.4 | 115.7 | 112.0 |
| ID66328 | 60.2 | 51.5 | 65.6 | 116.4 | 97.8 | 103.8 | 118.0 | 121.5 | 133.6 | 122.9 | 113.5 |
| ID66329 | 59.3 | 66.9 | 64.8 | 128.1 | 97.6 | 112.9 | 131.9 | 98.5 | 109.2 | 112.5 | 101.2 |
| ID66331 | 65.8 | 75.9 | 72.6 | 117.0 | 100.5 | 108.5 | 139.3 | 96.8 | 115.2 | 123.1 | 108.2 |
| ID66336 | 41.3 | 53.4 | 63.8 | 132.3 | 116.9 | 122.3 | 108.6 | 97.8 | 150.6 | 102.8 | 92.6 |
| ID66337 | 68.7 | 81.3 | 69.1 | 106.5 | 95.9 | 111.7 | 96.5 | 93.1 | 116.8 | 101.2 | 116.7 |
| ID66338 | 61.3 | 63.8 | 75.1 | 121.1 | 94.3 | 107.7 | 103.7 | 102.6 | 122.7 | 106.0 | 94.5 |
| ID66341 | 127.1 | 120.4 | 96.9 | 108.3 | 105.6 | 85.0 | 84.8 | 76.3 | 109.8 | 89.2 | 83.1 |
| ID66344 | 96.9 | 81.8 | 78.6 | 118.9 | 102.2 | 107.8 | 137.8 | 110.1 | 112.0 | 118.1 | 104.0 |
| ID66345 | 41.2 | 93.0 | 73.5 | 146.8 | 133.6 | 118.3 | 149.3 | 57.0 | 131.7 | 99.1 | 97.2 |
| ID66346 | 85.5 | 66.5 | 65.0 | 118.6 | 114.4 | 105.9 | 129.9 | 132.8 | 144.8 | 120.0 | 103.3 |
| ID66347 | 41.3 | 66.9 | 70.7 | 60.3 | 115.1 | 126.6 | 66.2 | 151.7 | 90.2 | 95.3 | 212.2 |
| ID66348 | 87.2 | 75.4 | 79.8 | 114.8 | 111.6 | 109.9 | 148.7 | 111.6 | 129.3 | 134.1 | 109.3 |
| ID66352 | 283.5 | 326.0 | 169.3 | 133.4 | 105.3 | 159.2 | 87.1 | 142.9 | 115.5 | 73.6 | 87.5 |
| ID66353 | 132.0 | 121.2 | 76.7 | 102.7 | 81.2 | 85.4 | 72.5 | 70.6 | 101.0 | 94.4 | 71.7 |
| ID66354 | 197.7 | 211.6 | 125.2 | 63.1 | 53.4 | 55.1 | 47.0 | 54.8 | 55.3 | 66.9 | 60.9 |
| ID66357 | 228.9 | 214.6 | 102.2 | 87.5 | 90.1 | 80.8 | 84.8 | 73.9 | 100.7 | 90.8 | 75.8 |
| ID66358 | 173.8 | 140.6 | 116.9 | 89.6 | 115.3 | 79.0 | 77.8 | 67.9 | 113.2 | 88.5 | 81.4 |
| ID66360 | 77.4 | 79.5 | 68.3 | 193.3 | 104.7 | 239.6 | 109.4 | 166.9 | 114.1 | 117.0 | 111.0 |
| ID66361 | 238.7 | 215.4 | 110.9 | 91.6 | 81.8 | 79.9 | 68.5 | 69.8 | 93.7 | 77.8 | 66.6 |
| ID66362 | 80.4 | 76.3 | 61.8 | 113.8 | 105.0 | 100.5 | 89.1 | 100.6 | 127.5 | 118.4 | 94.2 |
| ID66363 | 87.6 | 66.4 | 78.4 | 111.5 | 109.4 | 102.9 | 145.2 | 124.6 | 121.6 | 133.5 | 116.3 |
| ID66364 | 250.9 | 196.0 | 57.8 | 110.8 | 95.9 | 182.5 | 103.5 | 126.1 | 107.1 | 106.2 | 99.8 |
| ID66365 | 113.0 | 77.9 | 75.2 | 118.5 | 100.6 | 106.9 | 102.5 | 166.3 | 146.0 | 112.3 | 101.0 |
| ID66366 | 90.3 | 83.5 | 98.3 | 111.0 | 111.7 | 111.4 | 144.0 | 118.2 | 130.5 | 141.8 | 115.9 |
| ID66367 | 98.5 | 86.9 | 76.3 | 107.5 | 105.9 | 100.5 | 99.3 | 105.1 | 119.2 | 111.4 | 94.6 |
| ID66368 | 129.5 | 121.6 | 104.0 | 77.2 | 92.0 | 84.3 | 99.6 | 102.1 | 93.2 | 104.7 | 87.7 |
| ID66369 | 138.9 | 156.2 | 145.1 | 56.9 | 86.4 | 73.9 | 97.8 | 94.9 | 76.9 | 95.7 | 83.3 |
| ID66370 | 214.2 | 117.7 | 137.4 | 31.6 | 77.8 | 92.7 | 27.6 | 66.4 | 22.5 | 23.9 | 69.4 |
| ID66371 | 165.1 | 170.8 | 139.8 | 59.6 | 86.0 | 73.9 | 93.7 | 94.1 | 82.5 | 92.8 | 81.6 |
| ID66372 | 95.8 | 75.5 | 72.7 | 104.0 | 107.5 | 106.4 | 132.4 | 121.8 | 122.1 | 124.4 | 115.3 |
| ID66373 | 252.0 | 211.0 | 131.3 | 56.3 | 73.0 | 64.8 | 72.4 | 102.2 | 78.8 | 85.0 | 72.3 |
| ID66375 | 108.3 | 82.3 | 83.1 | 111.4 | 79.5 | 97.3 | 132.9 | 111.6 | 65.0 | 119.9 | 102.7 |
| ID66376 | 260.2 | 264.2 | 134.8 | 51.1 | 53.9 | 56.5 | 62.1 | 76.4 | 67.1 | 70.5 | 63.1 |
| ID66377 | 212.7 | 255.1 | 141.4 | 64.9 | 67.1 | 71.4 | 76.4 | 105.5 | 90.1 | 92.5 | 80.4 |
| ID66378 | 159.0 | 179.9 | 124.9 | 63.6 | 76.1 | 84.0 | 82.5 | 97.6 | 94.4 | 85.5 | 83.2 |
| ID66379 | 130.9 | 124.9 | 147.7 | 90.9 | 100.3 | 116.6 | 101.0 | 124.5 | 134.5 | 137.4 | 121.7 |
| ID66380 | 184.8 | 202.7 | 124.7 | 71.7 | 76.1 | 78.5 | 79.5 | 102.4 | 102.0 | 101.6 | 84.2 |
| ID66381 | 196.3 | 134.4 | 101.2 | 89.8 | 111.9 | 98.1 | 108.0 | 159.1 | 142.2 | 120.9 | 94.4 |
| ID66383 | 132.4 | 117.8 | 97.0 | 103.6 | 99.2 | 97.0 | 104.8 | 114.4 | 130.7 | 133.6 | 93.1 |
| ID66384 | 66.0 | 57.4 | 66.0 | 124.8 | 88.0 | 93.5 | 122.2 | 120.1 | 155.0 | 135.0 | 91.7 |
| ID66386 | 60.3 | 61.1 | 55.0 | 126.7 | 102.5 | 99.5 | 122.7 | 138.4 | 208.7 | 205.9 | 113.3 |
| ID66387 | 201.8 | 190.2 | 93.2 | 106.5 | 72.8 | 76.5 | 83.4 | 56.2 | 100.5 | 85.7 | 73.1 |
| ID66390 | 165.4 | 148.1 | 123.1 | 107.1 | 93.9 | 99.9 | 95.0 | 100.9 | 112.1 | 99.1 | 90.8 |
| ID66392 | 87.6 | 85.9 | 58.9 | 116.4 | 105.5 | 91.8 | 89.7 | 120.3 | 184.0 | 176.8 | 99.0 |
| ID66393 | 72.3 | 51.9 | 42.3 | 107.9 | 108.5 | 99.9 | 122.5 | 139.8 | 132.0 | 181.2 | 124.8 |
| ID66394 | 85.9 | 76.2 | 68.2 | 104.9 | 96.4 | 92.5 | 110.1 | 102.7 | 132.2 | 125.3 | 95.4 |
| ID66395 | 63.1 | 75.9 | 67.8 | 123.4 | 106.7 | 101.9 | 126.9 | 98.3 | 125.1 | 124.3 | 110.0 |
| ID66397 | 382.1 | 288.4 | 191.8 | 88.2 | 78.0 | 71.8 | 59.9 | 98.1 | 88.9 | 92.9 | 52.8 |
| ID66398 | 172.2 | 85.9 | 65.9 | 118.7 | 93.5 | 80.0 | 54.6 | 104.1 | 174.3 | 195.1 | 105.1 |
| ID66399 | 57.9 | 43.9 | 38.1 | 128.6 | 112.3 | 119.7 | 97.1 | 146.0 | 142.8 | 147.8 | 112.3 |
| ID66400 | 77.3 | 114.0 | 80.7 | 106.8 | 117.0 | 131.6 | 139.0 | 179.6 | 158.7 | 143.8 | 142.0 |
| ID66401 | 97.5 | 66.9 | 70.1 | 127.4 | 119.2 | 96.3 | 108.0 | 156.9 | 245.1 | 250.6 | 134.8 |
| ID66402 | 64.1 | 107.2 | 91.6 | 117.3 | 123.2 | 111.6 | 140.3 | 109.4 | 135.8 | 135.9 | 107.0 |
| ID66403 | 105.6 | 117.0 | 113.1 | 109.6 | 105.3 | 100.6 | 124.0 | 102.6 | 129.8 | 121.6 | 90.4 |
| ID66404 | 121.2 | 126.2 | 85.8 | 98.5 | 84.2 | 93.4 | 146.7 | 94.7 | 110.2 | 100.8 | 83.0 |
| ID66405 | 108.3 | 122.3 | 100.9 | 111.0 | 116.6 | 100.7 | 120.9 | 107.7 | 139.6 | 140.2 | 99.8 |
| ID66407 | 18.9 | 8.0 | 24.4 | 147.6 | 153.0 | 163.7 | 121.8 | 162.8 | 144.5 | 150.3 | 132.0 |
| ID66408 | 167.6 | 166.8 | 91.7 | 84.7 | 69.1 | 84.8 | 132.8 | 80.1 | 99.2 | 82.2 | 71.7 |
| ID66409 | 136.3 | 190.1 | 107.2 | 135.2 | 81.5 | 58.6 | 118.8 | 84.6 | 142.1 | 141.3 | 84.0 |
| ID66410 | 74.7 | 77.2 | 71.1 | 110.7 | 99.8 | 102.7 | 144.6 | 111.5 | 127.0 | 118.6 | 97.7 |
| ID66411 | 62.0 | 54.9 | 78.8 | 123.4 | 116.7 | 112.7 | 167.2 | 135.4 | 142.5 | 140.4 | 122.6 |
| ID66412 | 61.3 | 97.7 | 87.7 | 111.3 | 126.5 | 92.9 | 130.2 | 104.8 | 140.1 | 128.4 | 104.8 |
| ID66413 | 203.7 | 171.6 | 100.1 | 89.1 | 76.1 | 63.5 | 91.0 | 94.7 | 127.9 | 132.6 | 81.9 |
| ID66415 | 107.2 | 120.1 | 81.3 | 111.7 | 67.0 | 60.6 | 99.7 | 73.3 | 97.1 | 123.4 | 95.5 |
| ID66416 | 162.0 | 179.5 | 132.4 | 85.8 | 48.7 | 44.8 | 51.9 | 83.8 | 119.4 | 119.7 | 68.7 |
| ID66418 | 5.6 | 12.0 | 27.4 | 76.4 | 127.9 | 134.6 | 72.6 | 151.6 | 112.0 | 106.4 | 121.9 |
| ID66419 | 116.8 | 70.9 | 44.8 | 120.9 | 103.8 | 98.7 | 128.8 | 183.8 | 140.0 | 198.3 | 135.0 |
| ID66420 | 275.0 | 225.2 | 218.2 | 71.0 | 68.9 | 71.4 | 52.5 | 59.3 | 90.4 | 89.9 | 53.9 |
| ID66422 | 382.8 | 275.6 | 190.4 | 76.3 | 41.9 | 95.6 | 74.4 | 73.3 | 135.5 | 85.4 | 54.7 |
| ID66423 | 62.1 | 75.4 | 61.6 | 115.8 | 113.2 | 117.0 | 158.2 | 154.8 | 138.0 | 122.0 | 122.1 |
| ID66424 | 138.6 | 134.7 | 93.2 | 85.4 | 51.3 | 73.1 | 77.8 | 93.2 | 83.5 | 90.5 | 70.8 |
| ID66426 | 488.2 | 502.3 | 218.3 | 84.3 | 60.6 | 63.4 | 36.6 | 62.3 | 128.8 | 136.7 | 43.0 |
| ID66427 | 149.1 | 132.8 | 119.7 | 112.1 | 117.2 | 96.3 | 135.1 | 118.9 | 145.8 | 121.3 | 89.5 |
| ID66428 | 166.4 | 160.2 | 89.2 | 99.3 | 111.9 | 77.7 | 127.0 | 119.7 | 147.2 | 138.9 | 110.3 |
| ID66429 | 123.2 | 163.2 | 107.1 | 121.3 | 86.9 | 94.0 | 113.5 | 102.1 | 128.8 | 147.7 | 98.4 |
| ID66430 | 224.9 | 209.5 | 158.5 | 108.0 | 111.0 | 92.3 | 116.7 | 108.1 | 142.6 | 110.6 | 71.5 |
| ID66432 | 77.5 | 74.1 | 67.9 | 104.7 | 95.1 | 106.2 | 83.4 | 87.3 | 105.0 | 91.7 | 101.2 |
| ID66434 | 66.6 | 74.4 | 73.6 | 119.2 | 95.1 | 100.6 | 124.6 | 80.5 | 102.0 | 102.3 | 83.9 |
| ID66435 | 301.3 | 326.2 | 175.4 | 53.0 | 37.3 | 34.0 | 42.0 | 24.2 | 56.1 | 38.4 | 30.4 |
| ID66436 | 134.3 | 134.6 | 63.8 | 121.6 | 140.3 | 145.1 | 125.5 | 113.8 | 129.4 | 159.6 | 96.5 |
| ID66438 | 88.6 | 87.7 | 83.6 | 129.1 | 102.8 | 105.1 | 131.5 | 106.2 | 108.4 | 107.3 | 89.2 |
| ID66439 | 142.8 | 134.0 | 107.6 | 103.1 | 107.1 | 89.4 | 96.6 | 103.7 | 119.8 | 100.9 | 79.0 |
| ID66440 | 66.3 | 71.4 | 73.9 | 110.6 | 126.7 | 110.1 | 137.4 | 121.0 | 126.2 | 141.5 | 110.2 |
| ID66441 | 59.5 | 64.2 | 80.6 | 120.4 | 97.9 | 102.4 | 132.0 | 79.9 | 107.5 | 95.6 | 90.4 |
| ID66442 | 232.4 | 239.5 | 157.3 | 83.2 | 120.6 | 97.9 | 73.7 | 75.3 | 74.4 | 114.0 | 60.3 |
| ID66443 | 112.0 | 98.8 | 97.5 | 75.1 | 98.7 | 78.0 | 92.4 | 76.6 | 99.0 | 99.9 | 81.6 |
| ID66444 | 227.4 | 246.5 | 191.1 | 93.8 | 60.0 | 67.4 | 80.5 | 62.0 | 78.1 | 52.4 | 43.4 |
| ID66445 | 204.0 | 281.9 | 214.4 | 90.6 | 41.3 | 52.4 | 93.6 | 55.5 | 56.7 | 44.3 | 52.2 |
| ID66446 | 125.9 | 121.2 | 105.7 | 97.7 | 88.5 | 89.4 | 101.7 | 79.4 | 103.4 | 96.3 | 77.3 |
| ID66447 | 217.0 | 159.5 | 118.2 | 117.8 | 53.3 | 74.8 | 68.5 | 115.7 | 120.0 | 111.8 | 90.4 |
| ID66450 | 78.7 | 77.8 | 90.8 | 121.6 | 100.7 | 101.4 | 134.9 | 94.3 | 117.2 | 121.2 | 101.9 |
| ID66451 | 96.9 | 98.6 | 103.3 | 81.3 | 91.1 | 92.9 | 86.2 | 89.9 | 89.4 | 86.0 | 98.1 |
| ID66452 | 179.6 | 172.2 | 142.7 | 128.4 | 87.5 | 79.8 | 117.2 | 62.6 | 107.3 | 112.4 | 73.0 |
| ID66453 | 156.9 | 177.0 | 90.6 | 102.3 | 108.4 | 83.0 | 118.6 | 98.4 | 113.7 | 119.1 | 93.4 |
| ID66454 | 135.2 | 179.7 | 109.2 | 102.3 | 87.0 | 84.7 | 115.9 | 71.7 | 98.8 | 97.2 | 93.2 |
| ID66456 | 140.1 | 138.5 | 112.0 | 63.5 | 95.7 | 75.9 | 110.6 | 108.6 | 110.6 | 124.8 | 92.8 |
| ID66457 | 143.4 | 147.3 | 106.4 | 91.6 | 78.7 | 73.1 | 99.0 | 78.6 | 91.0 | 93.6 | 81.1 |
| ID66458 | 99.8 | 119.3 | 105.7 | 108.4 | 92.7 | 84.2 | 134.6 | 84.5 | 113.5 | 117.6 | 94.9 |
| ID66459 | 58.5 | 78.2 | 73.4 | 126.2 | 122.6 | 97.3 | 149.7 | 104.8 | 117.0 | 143.1 | 139.1 |
| ID66460 | 158.1 | 175.7 | 126.7 | 105.8 | 97.3 | 75.2 | 116.5 | 77.5 | 110.5 | 116.3 | 92.9 |
| ID66461 | 150.2 | 163.7 | 121.9 | 98.7 | 82.5 | 78.5 | 129.8 | 62.7 | 85.2 | 85.5 | 91.6 |
| ID66463 | 105.1 | 98.4 | 93.5 | 108.2 | 88.6 | 88.7 | 117.3 | 78.8 | 96.8 | 83.7 | 80.2 |
| ID66465 | 71.9 | 86.4 | 98.0 | 106.4 | 90.1 | 89.8 | 121.2 | 78.9 | 104.7 | 104.7 | 98.9 |
| ID66466 | 175.3 | 143.4 | 118.4 | 121.4 | 112.9 | 96.6 | 123.5 | 98.5 | 109.2 | 94.5 | 83.7 |
| ID66467 | 97.3 | 96.5 | 98.5 | 133.6 | 114.7 | 100.1 | 143.7 | 116.5 | 144.4 | 132.5 | 99.2 |
| ID66468 | 406.2 | 565.4 | 385.6 | 37.6 | 0.2 | 18.3 | 27.0 | 1.5 | 3.5 | 19.6 | 35.7 |
| ID66470 | 75.3 | 66.9 | 117.4 | 148.8 | 153.8 | 120.0 | 111.0 | 128.5 | 252.6 | 229.0 | 132.1 |
| ID66471 | 25.3 | 27.9 | 22.2 | 123.5 | 143.4 | 171.8 | 119.7 | 145.1 | 59.0 | 138.5 | 117.6 |
| ID66472 | 120.6 | 82.8 | 43.6 | 90.6 | 138.4 | 114.5 | 82.2 | 118.3 | 64.4 | 111.9 | 77.0 |
| ID66473 | 194.4 | 58.0 | 235.1 | 1904.8 | 142.5 | 153.4 | 228.5 | 143.0 | 197.3 | 520.7 | 157.7 |
| ID66474 | 44.6 | 56.0 | 42.1 | 109.9 | 131.8 | 151.4 | 95.8 | 133.9 | 58.1 | 112.8 | 97.2 |
| ID66475 | 53.2 | 77.0 | 39.9 | 104.6 | 128.3 | 140.3 | 90.6 | 129.8 | 55.9 | 113.1 | 90.9 |
| ID66476 | 92.4 | 76.5 | 52.7 | 115.3 | 122.0 | 133.1 | 101.3 | 131.2 | 66.1 | 127.9 | 101.4 |
| ID66477 | 160.8 | 119.1 | 115.5 | 99.8 | 144.1 | 108.4 | 76.1 | 108.3 | 75.3 | 91.3 | 62.0 |
| ID66478 | 162.8 | 163.4 | 97.6 | 85.3 | 84.2 | 101.8 | 73.9 | 85.6 | 68.1 | 99.5 | 80.1 |
| ID66479 | 143.9 | 163.4 | 128.2 | 108.1 | 121.9 | 79.8 | 52.2 | 144.5 | 195.2 | 99.6 | 70.4 |
| ID66483 | 243.1 | 208.1 | 157.2 | 186.1 | 114.9 | 70.7 | 57.1 | 114.7 | 210.8 | 159.3 | 44.7 |
| ID66484 | 146.6 | 290.9 | 202.2 | 69.2 | 61.8 | 124.3 | 75.0 | 72.1 | 25.2 | 108.6 | 86.3 |
| ID66485 | 175.3 | 258.2 | 198.4 | 62.4 | 70.3 | 72.2 | 52.8 | 52.9 | 85.4 | 96.3 | 65.7 |
| ID66486 | 301.8 | 138.4 | 103.8 | 205.0 | 77.3 | 81.2 | 129.1 | 135.0 | 90.0 | 86.5 | 117.3 |
| ID66487 | 213.1 | 199.4 | 181.1 | 61.9 | 48.4 | 45.0 | 37.6 | 48.2 | 86.3 | 80.8 | 87.2 |
| ID66488 | 75.0 | 76.5 | 106.2 | 79.3 | 91.3 | 84.3 | 81.8 | 90.3 | 100.5 | 119.3 | 92.4 |
| ID66491 | 55.4 | 62.5 | 55.0 | 86.7 | 114.6 | 113.2 | 100.4 | 97.5 | 99.6 | 97.2 | 126.5 |
| ID66492 | 81.2 | 93.5 | 107.3 | 101.1 | 96.7 | 87.3 | 111.2 | 92.8 | 126.9 | 130.6 | 105.6 |
| ID66494 | 95.5 | 88.4 | 81.3 | 53.6 | 115.3 | 51.0 | 41.7 | 123.9 | 156.9 | 215.3 | 255.5 |
| ID66495 | 75.4 | 70.8 | 89.4 | 72.5 | 78.2 | 70.4 | 90.5 | 82.6 | 98.9 | 125.5 | 104.6 |
| ID66505 | 255.1 | 234.6 | 118.5 | 51.1 | 60.5 | 63.5 | 61.6 | 80.4 | 85.8 | 77.5 | 68.6 |
| ID66509 | 109.9 | 107.7 | 118.5 | 178.3 | 132.5 | 131.9 | 154.9 | 144.0 | 122.1 | 129.7 | 131.4 |
| ID66510 | 76.7 | 85.7 | 112.7 | 88.0 | 112.9 | 102.6 | 99.2 | 102.9 | 98.4 | 108.0 | 102.9 |
| ID66511 | 87.3 | 127.7 | 159.7 | 171.9 | 123.9 | 129.4 | 142.7 | 134.0 | 134.6 | 165.4 | 107.6 |
| ID66513 | 40.4 | 39.4 | 51.4 | 92.5 | 115.1 | 118.3 | 104.9 | 115.6 | 103.2 | 99.9 | 131.7 |
| ID66516 | 77.1 | 92.4 | 106.6 | 81.9 | 82.4 | 87.8 | 78.4 | 93.4 | 89.3 | 100.7 | 89.3 |
| ID66517 | 481.7 | 252.8 | 212.6 | 31.9 | 54.4 | 91.2 | 25.3 | 54.0 | 27.0 | 16.2 | 29.5 |
| ID66527 | 40.1 | 37.8 | 56.6 | 99.4 | 117.9 | 121.4 | 106.1 | 108.9 | 98.8 | 95.8 | 128.1 |
| ID66528 | 44.3 | 40.3 | 93.2 | 90.7 | 100.6 | 103.6 | 103.4 | 97.1 | 121.3 | 127.4 | 128.4 |
| ID66529 | 80.3 | 109.4 | 116.5 | 93.6 | 103.5 | 92.1 | 93.6 | 88.2 | 122.1 | 117.2 | 106.1 |
| ID66532 | 214.2 | 120.8 | 189.1 | 104.3 | 65.8 | 85.1 | 69.0 | 61.3 | 55.4 | 70.3 | 90.2 |
| ID66534 | 94.0 | 70.1 | 80.8 | 76.3 | 108.6 | 88.2 | 94.4 | 131.9 | 135.5 | 150.1 | 126.9 |
| ID66537 | 167.8 | 164.7 | 138.2 | 96.3 | 84.7 | 90.9 | 71.7 | 85.6 | 88.8 | 75.9 | 75.0 |
| ID66543 | 51.7 | 56.3 | 80.0 | 100.0 | 107.7 | 115.7 | 97.2 | 102.8 | 83.7 | 83.5 | 118.9 |
| ID66544 | 83.2 | 71.4 | 113.1 | 103.8 | 93.6 | 97.5 | 70.4 | 98.9 | 86.6 | 88.7 | 89.5 |
| ID66552 | 51.3 | 43.6 | 61.3 | 110.5 | 108.7 | 128.0 | 96.3 | 95.7 | 88.4 | 82.6 | 113.7 |
| ID66553 | 50.8 | 42.1 | 89.8 | 134.1 | 120.6 | 145.7 | 119.7 | 102.8 | 89.1 | 77.3 | 107.8 |
| ID66554 | 154.4 | 102.4 | 95.6 | 106.1 | 83.6 | 80.1 | 80.3 | 89.9 | 114.9 | 87.3 | 93.9 |
| ID66556 | 160.0 | 129.1 | 85.7 | 124.7 | 113.8 | 129.6 | 98.8 | 123.6 | 118.7 | 111.9 | 117.3 |
| ID66557 | 191.7 | 169.7 | 128.2 | 79.5 | 66.4 | 68.7 | 45.2 | 83.5 | 84.0 | 57.0 | 66.1 |
| ID66561 | 144.1 | 59.6 | 58.1 | 184.4 | 80.4 | 176.5 | 105.9 | 158.8 | 128.7 | 114.2 | 106.5 |
| ID66564 | 138.6 | 135.5 | 88.0 | 72.3 | 79.8 | 96.1 | 64.8 | 100.3 | 77.2 | 87.4 | 65.2 |
| ID66565 | 75.7 | 110.9 | 73.3 | 93.7 | 76.7 | 85.0 | 83.7 | 61.8 | 74.1 | 72.2 | 91.0 |
| ID66568 | 105.8 | 124.7 | 84.9 | 91.0 | 74.8 | 78.0 | 92.6 | 68.0 | 77.8 | 81.0 | 81.6 |
| ID66571 | 96.0 | 89.7 | 97.7 | 87.8 | 99.2 | 94.7 | 98.5 | 88.5 | 93.1 | 84.9 | 90.3 |
| ID66574 | 126.4 | 124.3 | 115.0 | 84.7 | 96.1 | 94.5 | 68.4 | 88.1 | 81.5 | 79.0 | 84.2 |
| ID66577 | 117.5 | 126.7 | 121.5 | 77.2 | 84.4 | 83.8 | 75.0 | 88.6 | 77.5 | 90.5 | 88.9 |
| ID66578 | 86.4 | 100.2 | 77.0 | 82.8 | 79.8 | 101.8 | 89.6 | 82.9 | 95.3 | 80.9 | 89.8 |
| ID66579 | 113.1 | 113.9 | 96.2 | 83.2 | 82.4 | 82.2 | 89.9 | 75.3 | 88.1 | 75.4 | 82.8 |
| ID66580 | 100.6 | 108.1 | 107.3 | 108.3 | 115.2 | 95.4 | 90.4 | 141.6 | 122.4 | 93.8 | 91.5 |
| ID66581 | 136.7 | 145.9 | 115.9 | 67.7 | 83.3 | 92.9 | 65.5 | 81.0 | 63.5 | 67.6 | 69.0 |
| ID66582 | 153.8 | 206.9 | 141.4 | 54.0 | 54.8 | 49.6 | 35.0 | 59.4 | 55.2 | 67.3 | 69.1 |
| ID66588 | 102.1 | 110.8 | 86.9 | 89.4 | 74.1 | 78.3 | 87.3 | 82.9 | 90.1 | 91.6 | 86.6 |
| ID66589 | 100.4 | 90.2 | 88.8 | 93.1 | 94.4 | 91.0 | 94.3 | 103.0 | 102.9 | 98.9 | 99.7 |
| ID66590 | 163.5 | 153.1 | 136.6 | 101.4 | 77.4 | 81.9 | 83.0 | 104.2 | 88.6 | 120.2 | 109.6 |
| ID66593 | 182.7 | 256.3 | 179.0 | 51.4 | 50.3 | 50.6 | 50.3 | 56.6 | 54.6 | 61.4 | 57.8 |
| ID66594 | 112.1 | 99.7 | 94.0 | 74.1 | 89.8 | 87.6 | 81.0 | 115.5 | 85.0 | 81.2 | 89.0 |
| ID66595 | 137.2 | 111.1 | 75.1 | 71.2 | 72.9 | 61.6 | 73.2 | 75.9 | 60.9 | 69.3 | 77.4 |
| ID66596 | 111.9 | 103.9 | 69.5 | 74.7 | 70.5 | 64.0 | 74.4 | 65.0 | 62.6 | 67.4 | 82.8 |
| ID66597 | 112.6 | 88.5 | 62.6 | 74.0 | 68.4 | 68.3 | 82.1 | 83.4 | 58.6 | 74.6 | 85.7 |
| ID66598 | 147.8 | 160.1 | 126.8 | 77.8 | 86.5 | 127.8 | 51.7 | 92.1 | 61.2 | 44.7 | 47.5 |
| ID66599 | 105.6 | 109.9 | 104.3 | 79.9 | 89.5 | 89.9 | 82.8 | 106.5 | 99.1 | 78.7 | 93.6 |
| ID66603 | 92.8 | 124.2 | 96.1 | 74.0 | 64.9 | 62.8 | 77.8 | 56.2 | 69.8 | 62.4 | 63.2 |
| ID66605 | 81.7 | 120.5 | 95.2 | 91.1 | 79.4 | 77.1 | 89.4 | 93.8 | 105.7 | 93.6 | 94.9 |
| ID66606 | 217.1 | 209.9 | 114.7 | 57.3 | 57.2 | 60.9 | 59.1 | 68.0 | 63.4 | 64.0 | 55.4 |
| ID66607 | 151.2 | 123.8 | 120.6 | 208.7 | 87.3 | 102.3 | 157.5 | 133.2 | 105.3 | 157.9 | 172.6 |
| ID66608 | 111.3 | 119.1 | 124.9 | 78.1 | 100.2 | 89.5 | 72.1 | 83.2 | 87.0 | 91.4 | 82.6 |
| ID66612 | 84.8 | 65.4 | 114.9 | 76.1 | 77.6 | 92.2 | 83.0 | 83.5 | 68.8 | 76.9 | 111.3 |
| ID66613 | 199.4 | 110.6 | 115.3 | 86.9 | 100.9 | 88.3 | 108.0 | 110.7 | 119.9 | 97.2 | 109.7 |
| ID66614 | 215.2 | 165.0 | 134.4 | 40.8 | 57.6 | 87.4 | 33.9 | 54.2 | 35.8 | 20.5 | 28.5 |
| ID66615 | 106.1 | 89.0 | 119.0 | 109.2 | 95.7 | 89.3 | 104.4 | 86.2 | 127.3 | 116.6 | 286.6 |
| ID66619 | 162.4 | 106.6 | 110.2 | 73.7 | 83.6 | 80.8 | 84.7 | 102.4 | 85.4 | 79.2 | 81.8 |
| ID66621 | 277.0 | 174.1 | 128.3 | 51.8 | 60.8 | 81.1 | 40.7 | 68.7 | 46.1 | 37.8 | 45.2 |
| ID66623 | 239.5 | 135.5 | 129.9 | 57.7 | 65.7 | 79.5 | 49.9 | 78.8 | 53.5 | 61.0 | 60.3 |
| ID66624 | 167.7 | 127.4 | 130.7 | 72.6 | 84.2 | 90.2 | 70.7 | 72.5 | 64.9 | 70.1 | 64.5 |
| ID66625 | 104.3 | 100.4 | 105.9 | 71.9 | 88.9 | 85.0 | 80.7 | 94.1 | 86.2 | 89.6 | 78.2 |
| ID66626 | 130.5 | 113.9 | 117.2 | 84.3 | 80.0 | 94.3 | 68.8 | 100.7 | 96.0 | 91.4 | 79.3 |
| ID66628 | 198.3 | 189.2 | 146.9 | 57.3 | 54.5 | 84.8 | 49.6 | 75.6 | 65.7 | 54.9 | 65.5 |
| ID66631 | 97.9 | 108.2 | 93.4 | 164.0 | 128.8 | 134.7 | 86.1 | 133.3 | 107.2 | 88.8 | 91.7 |
| ID66632 | 374.3 | 218.6 | 159.0 | 55.4 | 47.5 | 107.0 | 30.2 | 59.0 | 25.0 | 19.2 | 35.5 |
| ID66637 | 281.5 | 196.5 | 175.0 | 160.8 | 110.0 | 148.6 | 143.6 | 170.9 | 109.6 | 94.2 | 92.6 |
| ID66638 | 46.2 | 84.2 | 127.1 | 110.3 | 129.8 | 124.2 | 102.4 | 127.2 | 112.2 | 121.9 | 170.4 |
| ID66639 | 338.0 | 207.4 | 157.6 | 45.2 | 47.7 | 87.1 | 26.9 | 67.2 | 31.9 | 19.2 | 44.9 |
| ID66640 | 79.6 | 63.9 | 82.2 | 94.2 | 15.1 | 76.1 | 105.5 | 79.2 | 22.8 | 86.0 | 107.2 |
| ID66642 | 91.5 | 102.4 | 97.3 | 101.6 | 119.3 | 116.9 | 97.4 | 110.9 | 101.9 | 94.1 | 103.4 |
| ID66643 | 275.3 | 202.2 | 166.4 | 58.6 | 66.7 | 71.2 | 62.2 | 106.2 | 67.8 | 63.9 | 105.4 |
| ID66644 | 152.5 | 109.6 | 88.4 | 101.6 | 107.0 | 109.4 | 89.7 | 182.0 | 107.6 | 107.4 | 166.6 |
| ID66645 | 429.0 | 297.6 | 206.0 | 45.9 | 39.8 | 51.4 | 29.7 | 55.4 | 34.6 | 35.0 | 41.1 |
| ID66646 | 61.2 | 35.6 | 90.4 | 171.5 | 214.3 | 269.7 | 198.9 | 200.2 | 153.5 | 142.1 | 343.3 |
| ID66647 | 207.6 | 145.1 | 113.6 | 77.2 | 70.2 | 65.8 | 44.5 | 91.1 | 90.4 | 73.6 | 62.2 |
| ID66649 | 134.9 | 108.4 | 51.9 | 80.1 | 148.4 | 111.0 | 79.8 | 142.7 | 142.2 | 127.5 | 93.0 |
| ID66650 | 100.6 | 93.5 | 92.5 | 102.2 | 97.4 | 102.1 | 109.0 | 98.3 | 94.3 | 86.9 | 83.1 |
| ID66651 | 81.1 | 71.3 | 81.5 | 98.0 | 110.1 | 124.6 | 84.7 | 97.7 | 97.1 | 90.3 | 111.1 |
| ID66652 | 154.5 | 131.9 | 95.7 | 106.4 | 115.1 | 126.6 | 84.9 | 121.5 | 102.7 | 83.9 | 83.1 |
| ID66653 | 79.0 | 90.2 | 79.1 | 105.7 | 107.4 | 110.8 | 103.4 | 106.1 | 100.3 | 99.8 | 112.8 |
| ID66655 | 89.1 | 86.4 | 86.0 | 120.1 | 108.2 | 118.3 | 110.9 | 93.1 | 96.7 | 88.5 | 96.8 |
| ID66656 | 212.9 | 189.5 | 174.3 | 106.1 | 85.5 | 89.6 | 68.0 | 108.2 | 67.0 | 61.6 | 74.5 |
| ID66658 | 57.4 | 70.7 | 80.4 | 100.5 | 110.1 | 110.0 | 99.6 | 97.0 | 95.9 | 88.8 | 109.0 |
| ID66661 | 107.9 | 66.5 | 94.5 | 96.8 | 114.8 | 126.9 | 93.1 | 103.5 | 104.7 | 96.8 | 118.4 |
| ID66663 | 141.3 | 80.3 | 92.7 | 90.0 | 106.4 | 111.1 | 92.8 | 106.3 | 96.4 | 96.4 | 88.9 |
| ID66664 | 169.7 | 134.0 | 293.7 | 152.5 | 189.0 | 227.2 | 136.5 | 132.7 | 73.6 | 65.6 | 175.9 |
| ID66667 | 109.2 | 91.1 | 93.2 | 92.0 | 86.1 | 90.2 | 101.3 | 94.1 | 81.5 | 82.5 | 73.2 |
| ID66671 | 124.6 | 115.9 | 92.7 | 97.8 | 81.6 | 91.7 | 90.5 | 78.5 | 74.8 | 67.4 | 74.0 |
| ID66673 | 155.7 | 126.2 | 99.6 | 89.9 | 85.6 | 95.1 | 105.4 | 91.2 | 86.1 | 100.7 | 110.4 |
| ID66674 | 121.9 | 98.7 | 111.5 | 118.6 | 105.4 | 116.7 | 103.9 | 109.7 | 103.8 | 110.1 | 98.8 |
| ID66675 | 100.8 | 110.9 | 113.7 | 100.8 | 101.7 | 105.9 | 94.8 | 89.7 | 77.7 | 72.5 | 100.1 |
| ID66676 | 127.5 | 149.1 | 139.1 | 138.9 | 101.8 | 109.7 | 120.6 | 118.1 | 113.8 | 112.4 | 102.5 |
| ID66677 | 106.7 | 105.7 | 119.4 | 92.5 | 107.3 | 96.1 | 100.9 | 100.0 | 79.3 | 89.0 | 98.1 |
| ID66678 | 116.8 | 137.5 | 131.8 | 76.0 | 84.6 | 76.7 | 78.8 | 96.0 | 92.9 | 104.6 | 92.8 |
| ID66681 | 119.7 | 115.2 | 104.9 | 91.8 | 88.8 | 86.4 | 92.7 | 82.2 | 74.0 | 80.4 | 77.5 |
| ID66682 | 73.3 | 109.6 | 96.7 | 114.9 | 121.3 | 116.3 | 111.2 | 105.5 | 128.9 | 119.4 | 127.2 |
| ID66683 | 88.6 | 70.2 | 108.1 | 82.5 | 106.8 | 98.9 | 74.1 | 97.1 | 82.2 | 76.1 | 96.3 |
| ID66684 | 119.0 | 98.6 | 128.8 | 107.6 | 92.0 | 106.2 | 84.9 | 71.5 | 69.8 | 64.2 | 68.9 |
| ID66686 | 180.7 | 127.9 | 99.2 | 89.6 | 90.8 | 90.8 | 72.8 | 108.4 | 84.5 | 69.0 | 71.0 |
| ID66689 | 115.1 | 109.7 | 99.3 | 103.8 | 90.1 | 93.0 | 100.0 | 104.5 | 88.1 | 83.8 | 86.6 |
| ID66694 | 117.6 | 126.6 | 117.3 | 81.4 | 90.7 | 94.1 | 80.1 | 84.2 | 80.6 | 73.3 | 97.3 |
| ID66695 | 97.7 | 96.4 | 116.1 | 86.7 | 101.4 | 92.4 | 98.0 | 144.3 | 89.0 | 112.0 | 161.4 |
| ID66703 | 122.1 | 143.7 | 135.2 | 94.6 | 100.7 | 88.2 | 93.7 | 91.3 | 89.7 | 92.2 | 96.1 |
| ID66705 | 169.7 | 156.0 | 137.9 | 74.2 | 85.8 | 94.1 | 82.1 | 92.6 | 80.4 | 77.2 | 82.1 |
| ID66706 | 95.1 | 109.9 | 107.9 | 80.7 | 86.1 | 75.3 | 58.3 | 103.4 | 94.5 | 96.4 | 104.1 |
| ID66707 | 115.7 | 89.0 | 107.6 | 129.4 | 104.5 | 104.0 | 90.4 | 101.3 | 94.7 | 104.6 | 111.5 |
| ID66710 | 145.8 | 125.2 | 115.4 | 98.4 | 83.2 | 94.8 | 82.4 | 93.5 | 84.4 | 75.4 | 74.4 |
| ID66712 | 85.6 | 95.5 | 103.8 | 117.3 | 113.5 | 119.2 | 104.7 | 105.1 | 106.3 | 104.3 | 122.8 |
| ID66713 | 77.3 | 90.7 | 87.6 | 97.6 | 94.9 | 102.4 | 93.4 | 91.1 | 101.7 | 92.5 | 99.6 |
| ID66714 | 86.9 | 47.4 | 84.9 | 75.5 | 116.2 | 106.1 | 67.2 | 87.0 | 87.1 | 86.2 | 122.5 |
| ID66715 | 108.8 | 129.0 | 95.4 | 88.9 | 86.5 | 96.1 | 91.4 | 93.8 | 99.8 | 91.0 | 94.8 |
| ID66717 | 136.9 | 125.0 | 104.5 | 77.6 | 75.7 | 86.5 | 79.4 | 79.6 | 86.7 | 79.2 | 75.9 |
| ID66720 | 59.2 | 74.4 | 85.4 | 94.6 | 108.8 | 114.7 | 83.7 | 99.2 | 105.5 | 106.4 | 127.0 |
| ID66721 | 81.5 | 84.1 | 86.9 | 86.6 | 81.7 | 85.7 | 77.9 | 82.5 | 86.6 | 85.4 | 96.0 |
| ID66722 | 98.6 | 124.1 | 123.0 | 86.3 | 112.9 | 116.6 | 94.7 | 62.8 | 82.6 | 59.0 | 84.4 |
| ID66726 | 123.2 | 131.9 | 99.3 | 100.0 | 93.6 | 89.2 | 110.2 | 112.4 | 109.0 | 99.9 | 109.5 |
| ID66728 | 174.4 | 114.1 | 130.6 | 104.6 | 114.2 | 142.9 | 85.1 | 120.8 | 78.5 | 65.0 | 121.2 |
| ID66730 | 77.5 | 90.1 | 108.2 | 74.6 | 89.0 | 88.5 | 68.7 | 93.2 | 79.1 | 74.1 | 108.5 |
| ID66731 | 325.1 | 182.4 | 151.8 | 87.3 | 90.4 | 79.5 | 48.4 | 126.8 | 61.7 | 61.4 | 103.8 |
| ID66732 | 99.5 | 116.0 | 105.2 | 122.4 | 93.0 | 82.7 | 161.8 | 84.9 | 137.4 | 120.0 | 117.3 |
| ID66733 | 258.1 | 176.0 | 133.1 | 70.6 | 83.4 | 115.4 | 41.7 | 106.1 | 41.9 | 37.0 | 81.8 |
| ID66738 | 265.0 | 139.6 | 63.5 | 122.3 | 84.6 | 99.1 | 67.2 | 92.7 | 73.0 | 65.7 | 78.2 |
| ID66742 | 117.4 | 74.5 | 112.7 | 82.4 | 115.0 | 107.9 | 82.9 | 123.3 | 96.7 | 100.3 | 106.1 |
| ID66743 | 47.5 | 54.3 | 56.4 | 99.8 | 99.4 | 106.0 | 83.5 | 89.6 | 84.4 | 77.8 | 105.0 |
| ID66747 | 268.1 | 188.6 | 194.0 | 700.5 | 176.2 | 121.7 | 355.9 | 207.6 | 149.0 | 169.6 | 333.0 |
| ID66749 | 208.5 | 123.2 | 181.1 | 77.6 | 85.7 | 68.9 | 51.9 | 65.6 | 62.8 | 74.6 | 77.0 |
| ID66752 | 38.9 | 39.0 | 60.3 | 105.6 | 132.3 | 138.6 | 99.9 | 101.4 | 85.0 | 71.5 | 115.2 |
| ID66759 | 88.2 | 138.2 | 116.1 | 168.2 | 139.7 | 149.9 | 177.3 | 124.4 | 127.4 | 120.6 | 142.5 |
| ID66761 | 155.1 | 98.8 | 130.5 | 101.3 | 104.5 | 119.9 | 79.7 | 97.2 | 85.1 | 54.6 | 110.7 |
| ID66764 | 375.3 | 472.9 | 305.4 | 45.8 | 44.7 | 47.8 | 23.7 | 42.7 | 23.9 | 16.6 | 36.7 |
| ID66768 | 69.5 | 101.5 | 100.7 | 120.2 | 119.8 | 115.2 | 91.7 | 88.4 | 94.0 | 81.2 | 96.8 |
| ID66770 | 110.1 | 109.3 | 160.0 | 127.4 | 180.1 | 273.3 | 92.7 | 198.5 | 109.8 | 82.1 | 253.2 |
| ID66773 | 315.4 | 218.1 | 289.9 | 524.1 | 418.9 | 204.7 | 510.6 | 367.7 | 416.4 | 336.8 | 605.8 |
| ID66775 | 236.9 | 276.4 | 179.2 | 73.2 | 72.3 | 71.5 | 82.0 | 59.9 | 44.2 | 70.1 | 64.2 |
| ID66776 | 202.6 | 173.9 | 121.4 | 43.7 | 54.2 | 48.3 | 33.8 | 86.0 | 32.6 | 32.2 | 79.1 |
| ID66779 | 16.2 | 17.0 | 43.4 | 108.6 | 139.4 | 148.5 | 75.0 | 104.3 | 72.2 | 65.2 | 113.8 |
| ID66780 | 30.6 | 28.1 | 57.9 | 123.4 | 116.0 | 124.4 | 93.8 | 95.2 | 84.6 | 93.2 | 99.0 |
| ID66790 | 49.1 | 69.4 | 93.4 | 91.3 | 107.8 | 107.6 | 88.1 | 112.7 | 83.5 | 91.5 | 118.1 |
| ID66793 | 40.9 | 8.1 | 64.4 | 33.2 | 122.9 | 145.2 | 63.7 | 132.7 | 80.5 | 79.8 | 130.6 |
| ID66796 | 148.5 | 143.9 | 169.0 | 59.7 | 67.1 | 62.4 | 53.2 | 63.1 | 65.3 | 59.1 | 87.1 |
| ID66797 | 96.8 | 85.2 | 96.0 | 117.0 | 137.6 | 147.9 | 138.3 | 137.0 | 58.7 | 113.6 | 109.7 |
| ID66802 | 90.0 | 87.6 | 111.0 | 81.1 | 100.1 | 104.8 | 100.8 | 104.5 | 76.5 | 75.8 | 120.6 |
| ID66806 | 698.1 | 449.4 | 169.0 | 160.3 | 16.1 | 5.2 | 189.0 | 25.6 | 8.0 | 9.7 | 33.8 |
| ID66807 | 64.1 | 63.6 | 84.7 | 102.4 | 116.2 | 117.2 | 94.5 | 103.8 | 87.9 | 89.1 | 124.8 |
| ID66808 | 59.1 | 59.9 | 63.2 | 93.9 | 137.9 | 140.4 | 95.8 | 176.4 | 39.3 | 95.3 | 107.2 |
| ID66812 | 52.8 | 53.7 | 56.8 | 148.6 | 109.3 | 103.9 | 117.8 | 91.5 | 105.0 | 95.3 | 106.9 |
| ID66816 | 33.9 | 63.0 | 49.1 | 106.4 | 139.1 | 146.0 | 113.4 | 145.5 | 42.7 | 126.8 | 126.4 |
| ID66819 | 38.2 | 52.0 | 54.6 | 96.2 | 141.1 | 135.0 | 103.2 | 132.7 | 48.0 | 122.6 | 127.4 |
| ID66821 | 300.2 | 391.0 | 277.3 | 59.9 | 8.1 | 4.6 | 55.0 | 19.6 | 4.5 | 16.0 | 51.2 |
| ID66822 | 51.3 | 10.3 | 38.4 | 129.4 | 127.9 | 114.0 | 57.9 | 105.7 | 126.6 | 104.7 | 116.4 |
| ID66823 | 129.1 | 93.9 | 105.5 | 92.7 | 123.5 | 117.0 | 88.2 | 133.2 | 86.0 | 94.8 | 92.0 |
| ID66824 | 331.3 | 247.9 | 329.8 | 170.9 | 220.6 | 350.2 | 136.1 | 381.7 | 180.5 | 174.2 | 437.1 |
| ID66825 | 70.8 | 73.8 |  | 84.8 | 115.8 | 116.6 | 87.7 | 126.1 | 82.7 |  | 118.7 |
| ID66826 | 180.4 | 129.7 | 130.4 | 317.2 | 152.3 | 155.9 | 183.5 | 178.8 | 149.6 | 136.9 | 258.3 |
| ID66827 | 112.2 | 329.0 | 225.5 | 38.6 | 44.9 | 40.6 | 31.7 | 54.6 | 30.2 | 34.7 | 58.5 |
| ID66833 | 82.4 | 75.1 | 98.2 | 77.4 | 101.9 | 120.9 | 80.5 | 129.0 | 66.8 | 68.3 | 115.4 |
| ID66834 | 73.4 | 42.1 | 44.1 | 111.3 | 150.7 | 144.8 | 95.3 | 153.7 | 67.7 | 98.9 | 122.6 |
| ID66838 | 68.0 | 76.6 | 95.4 | 107.1 | 118.0 | 120.7 | 112.8 | 125.0 | 97.9 | 100.9 | 108.9 |
| ID66840 | 167.3 | 195.6 | 275.0 | 112.8 | 144.8 | 222.5 | 96.5 | 205.5 | 125.0 | 133.6 | 345.9 |
| ID66841 | 374.4 | 199.8 | 166.4 | 115.3 | 84.6 | 65.6 | 58.8 | 118.4 | 47.2 | 57.2 | 92.0 |
| ID66844 | 40.1 | 67.1 | 85.3 | 111.1 | 114.6 | 116.7 | 83.2 | 149.6 | 75.6 | 114.9 | 110.5 |
| ID66847 | 108.3 | 81.4 | 88.4 | 63.0 | 109.5 | 102.4 | 75.0 | 119.8 | 77.9 | 88.2 | 108.7 |
| ID66850 | 75.8 | 117.8 | 168.7 | 39.5 | 74.4 | 70.4 | 57.3 | 90.6 | 54.3 | 67.8 | 89.3 |
| ID66851 | 91.4 | 49.5 | 62.9 | 68.8 | 117.8 | 110.0 | 74.7 | 175.4 | 61.4 | 86.9 | 112.0 |
| ID66852 | 104.6 | 100.1 | 95.5 | 134.4 | 99.9 | 105.8 | 107.4 | 85.8 | 85.3 | 88.9 | 96.7 |
| ID66856 | 83.9 | 39.1 | 127.7 | 163.2 | 89.0 | 100.5 | 131.5 | 93.3 | 114.3 | 68.9 | 108.0 |
| ID66859 | 161.4 | 123.4 | 128.2 | 67.2 | 84.2 | 87.2 | 66.2 | 102.0 | 82.8 | 77.9 | 93.0 |
| ID66861 | 177.3 | 118.6 | 105.5 | 108.8 | 85.3 | 91.4 | 71.2 | 97.8 | 59.7 | 60.3 | 94.5 |
| ID66864 | 173.5 | 87.3 | 95.3 | 102.7 | 93.7 | 97.0 | 86.6 | 155.7 | 92.8 | 99.2 | 94.5 |
| ID66866 | 63.7 | 68.2 | 101.7 | 100.3 | 94.5 | 94.1 | 92.4 | 89.4 | 78.9 | 76.5 | 98.0 |
| ID66868 | 43.4 | 57.9 | 65.9 | 95.2 | 118.8 | 109.0 | 92.9 | 117.7 | 115.2 | 115.2 | 135.4 |
| ID66872 | 72.2 | 85.1 | 111.4 | 102.0 | 102.9 | 126.5 | 67.9 | 103.8 | 84.5 | 76.0 | 136.3 |
| ID66873 | 128.4 | 85.4 | 102.7 | 68.6 | 68.3 | 88.0 | 55.8 | 95.4 | 50.1 | 77.3 | 90.7 |
| ID66877 | 105.4 | 112.2 | 174.1 | 43.2 | 62.5 | 72.7 | 23.5 | 76.7 | 48.5 | 40.7 | 149.9 |
| ID66878 | 167.4 | 107.7 | 88.6 | 114.0 | 82.5 | 89.6 | 80.2 | 111.0 | 69.2 | 82.3 | 114.8 |
| ID66879 | 109.4 | 85.8 | 75.6 | 120.8 | 101.3 | 98.2 | 91.8 | 118.9 | 95.9 | 106.2 | 107.6 |
| ID66881 | 137.7 | 238.0 | 168.2 | 85.5 | 78.7 | 90.9 | 62.3 | 90.8 | 72.4 | 57.0 | 80.4 |
| ID66884 | 379.9 | 297.5 | 203.7 | 3.7 | 3.1 | 5.0 | 2.5 | 13.0 | 4.7 | 8.0 | 8.3 |
| ID66886 | 272.6 | 223.9 | 200.5 | 48.8 | 61.0 | 45.9 | 23.9 | 49.8 | 78.3 | 43.6 | 49.2 |
| ID66887 | 224.9 | 483.2 | 265.2 | 10.6 | 6.8 | 6.7 | 6.2 | 22.8 | 7.9 | 17.4 | 19.3 |
| ID66892 | 99.1 | 89.0 | 67.5 | 77.9 | 100.1 | 92.8 | 76.9 | 94.6 | 71.5 | 75.9 | 105.4 |
| ID66893 | 79.3 | 92.3 | 97.2 | 81.1 | 93.7 | 94.4 | 77.9 | 90.8 | 75.6 | 82.0 | 101.0 |
| ID66894 | 348.9 | 280.5 | 159.7 | 18.2 | 21.8 | 32.2 | 11.3 | 35.5 | 16.0 | 11.3 | 21.6 |
| ID66897 | 334.1 | 242.2 | 151.7 | 57.6 | 91.1 | 53.4 | 40.1 | 76.4 | 76.3 | 56.3 | 51.1 |
| ID66900 | 365.5 | 43.4 | 102.0 | 109.9 | 92.3 | 89.3 | 76.0 | 113.0 | 85.4 | 86.3 | 86.7 |
| ID66901 | 276.9 | 282.9 | 212.6 | 1.9 | 1.4 | 1.8 | 4.3 | 9.0 | 2.5 | 3.3 | 2.8 |
| ID66902 | 79.9 | 51.4 | 49.6 | 118.4 | 85.3 | 104.9 | 97.5 | 105.1 | 80.9 | 73.2 | 103.1 |
| ID66903 | 164.9 | 174.2 | 164.2 | 74.7 | 31.2 | 85.7 | 50.8 | 153.4 | 188.6 | 117.2 | 126.1 |
| ID66904 | 213.1 | 225.1 | 214.3 | 60.4 | 17.9 | 49.7 | 33.2 | 121.3 | 127.7 | 94.1 | 103.6 |
| ID66905 | 45.9 | 60.9 | 77.2 | 64.9 | 91.3 | 87.7 | 57.0 | 75.6 | 62.2 | 54.5 | 93.4 |
| ID66906 | 98.0 | 119.2 | 100.5 | 42.4 | 63.0 | 47.9 | 32.2 | 61.2 | 64.1 | 58.1 | 61.2 |
| ID66907 | 117.0 | 172.6 | 140.2 | 22.4 | 14.3 | 39.5 | 22.7 | 130.9 | 99.2 | 86.4 | 148.9 |
| ID66908 | 91.0 | 117.6 | 92.0 | 24.4 | 145.4 | 75.3 | 37.5 | 105.2 | 70.3 | 119.3 | 196.6 |
| ID66909 | 151.6 | 111.1 | 120.8 | 98.1 | 104.7 | 108.3 | 72.4 | 105.7 | 69.1 | 76.0 | 93.0 |
| ID66911 | 221.5 | 156.7 | 174.8 | 304.4 | 260.7 | 181.9 | 234.9 | 240.7 | 206.9 | 135.3 | 298.8 |
| ID66912 | 259.3 | 265.9 | 144.1 | 20.9 | 50.5 | 27.0 | 19.7 | 56.8 | 22.4 | 43.3 | 87.0 |
| ID66913 | 234.9 | 192.0 | 119.9 | 29.0 | 32.2 | 40.0 | 23.1 | 28.7 | 23.7 | 21.7 | 30.4 |
| ID66914 | 165.1 | 127.6 | 147.0 | 36.4 | 44.3 | 36.3 | 33.1 | 131.1 | 108.7 | 155.0 | 131.4 |
| ID66915 | 182.2 | 146.9 | 68.9 | 29.8 | 88.7 | 24.7 | 25.0 | 76.0 | 37.8 | 48.5 | 109.3 |
| ID66916 | 92.5 | 95.0 | 114.6 | 62.5 | 54.9 | 84.5 | 51.8 | 113.6 | 135.6 | 93.0 | 126.9 |
| ID66917 | 198.4 | 317.5 | 230.4 | 16.3 | 13.4 | 11.7 | 5.6 | 66.4 | 7.3 | 11.1 | 32.6 |
| ID66919 | 46.0 | 66.6 | 78.6 | 19.8 | 29.5 | 36.9 | 10.3 | 37.7 | 26.9 | 23.9 | 92.6 |
| ID66920 | 72.3 | 77.5 | 57.3 | 108.3 | 65.6 | 83.4 | 100.4 | 77.5 | 71.4 | 68.3 | 77.5 |
| ID66921 | 92.0 | 115.3 | 113.4 | 85.2 | 88.2 | 86.8 | 84.2 | 94.7 | 87.3 | 86.7 | 107.6 |
| ID66922 | 192.4 | 136.6 | 153.0 | 145.5 | 83.7 | 74.1 | 63.4 | 56.9 | 31.0 | 27.3 | 49.5 |
| ID66925 | 37.8 | 40.3 | 78.6 | 60.9 | 92.1 | 81.8 | 41.3 | 84.6 | 61.4 | 67.1 | 104.2 |
| ID66929 | 77.6 | 85.3 | 102.7 | 89.3 | 105.9 | 100.9 | 89.8 | 110.5 | 88.0 | 85.7 | 90.5 |
| ID66930 | 122.4 | 117.6 | 111.4 | 91.7 | 84.3 | 102.4 | 96.8 | 76.7 | 81.4 | 96.6 | 84.5 |
| ID66931 | 182.6 | 147.2 | 111.1 | 79.3 | 77.4 | 100.7 | 65.0 | 116.3 | 61.0 | 78.4 | 88.2 |
| ID66934 | 163.9 | 253.1 | 346.2 | 75.4 | 38.6 | 53.0 | 60.2 | 46.1 | 38.1 | 44.7 | 93.5 |
| ID66935 | 326.1 | 302.5 | 208.3 | 52.6 | 45.7 | 45.6 | 37.3 | 68.4 | 37.1 | 71.2 | 44.9 |
| ID66936 | 426.2 | 171.5 | 145.8 | 75.8 | 82.9 | 129.9 | 48.9 | 121.1 | 44.1 | 36.5 | 43.9 |
| ID66939 | 54.7 | 48.1 | 75.4 | 68.3 | 116.5 | 80.7 | 65.8 | 126.4 | 56.8 | 62.9 | 121.3 |
| ID890 | 91.5 | 36.2 | 70.9 | 135.4 | 95.2 | 99.3 | 104.1 | 102.1 | 186.4 | 167.1 | 107.6 |
| ID66942 | 58.7 | 58.3 | 66.1 | 122.5 | 121.1 | 126.8 | 138.6 | 116.8 | 103.1 | 100.4 | 125.2 |
| ID66943 | 31.6 | 47.4 | 33.3 | 123.2 | 132.6 | 134.3 | 174.7 | 155.6 | 87.9 | 137.5 | 127.3 |
| ID66944 | 383.7 | 236.4 | 295.0 | 299.0 | 147.5 | 95.6 | 201.6 | 144.7 | 132.9 | 141.7 | 305.5 |
| ID66945 | 342.8 | 173.3 | 165.3 | 55.0 | 80.6 | 73.5 | 43.9 | 79.7 | 47.0 | 34.9 | 32.5 |
| ID66946 | 36.5 | 52.0 | 42.2 | 135.9 | 123.9 | 126.7 | 146.9 | 85.0 | 101.5 | 104.1 | 105.4 |
| ID66947 | 145.8 | 115.3 | 143.6 | 92.7 | 85.7 | 86.0 | 95.7 | 67.2 | 76.8 | 70.5 | 106.6 |
| ID66948 | 91.2 | 73.7 | 75.2 | 136.8 | 103.5 | 117.8 | 121.2 | 121.0 | 86.2 | 94.8 | 99.0 |
| ID66949 | 75.8 | 58.0 | 80.9 | 119.5 | 95.9 | 106.2 | 107.6 | 85.6 | 99.2 | 86.6 | 96.5 |
| ID66951 | 332.6 | 129.7 | 106.3 | 82.7 | 104.2 | 95.6 | 77.1 | 159.3 | 51.7 | 104.4 | 57.4 |
| ID66952 | 74.0 | 83.4 | 80.9 | 100.2 | 113.8 | 108.8 | 117.0 | 117.9 | 99.0 | 96.5 | 108.8 |
| ID66953 | 53.7 | 47.6 | 50.7 | 134.4 | 106.2 | 115.7 | 136.9 | 100.9 | 106.9 | 106.8 | 123.8 |
| ID66955 | 126.3 | 113.1 | 95.8 | 90.5 | 72.4 | 76.0 | 82.2 | 116.3 | 82.9 | 80.6 | 82.7 |
| ID66956 | 319.0 | 193.5 | 183.9 | 31.4 | 31.1 | 22.7 |  | 50.0 | 21.4 | 30.2 |  |
| ID66957 | 76.7 | 65.5 | 56.9 | 137.1 | 110.0 | 105.0 | 99.8 | 112.7 | 92.1 | 98.3 | 98.9 |
| ID66959 | 101.2 | 72.6 | 69.8 | 125.8 | 98.1 | 113.0 | 112.7 | 116.7 | 90.0 | 89.7 | 100.1 |
| ID66960 | 14.2 | 12.6 | 90.6 | 12.6 | 52.1 | 78.2 |  | 175.6 | 12.3 | 6.4 |  |
| ID66961 | 61.8 | 56.5 | 58.8 | 144.3 | 87.3 | 99.1 | 120.8 | 103.4 | 72.4 | 85.3 | 101.9 |
| ID66962 | 89.5 | 89.3 | 122.4 | 112.3 | 111.3 | 104.9 | 124.6 | 58.3 | 52.8 | 53.7 | 92.5 |
| ID66963 | 95.6 | 62.9 | 69.6 | 103.2 | 78.4 | 95.3 | 1.6 | 94.9 | 76.5 | 83.4 | 26.9 |
| ID66964 | 118.1 | 117.2 | 93.1 | 103.8 | 95.5 | 90.1 | 70.1 | 75.3 | 49.8 | 61.2 | 61.3 |
| ID66965 | 142.8 | 113.9 | 159.7 | 103.2 | 81.6 | 115.5 |  | 100.4 | 74.4 | 108.9 |  |
| ID66966 | 113.7 | 64.7 | 65.3 | 94.7 | 77.3 | 89.6 | 103.2 | 108.3 | 65.4 | 74.4 | 89.2 |
| ID66967 | 126.1 | 104.3 | 73.0 | 102.8 | 73.3 | 93.2 | 89.9 | 100.9 | 93.4 | 104.2 | 86.1 |
| ID66968 | 139.2 | 70.3 | 86.9 | 95.4 | 77.8 | 91.3 | 93.2 | 113.0 | 73.4 | 87.8 | 83.9 |
| ID66969 | 267.4 | 155.4 | 125.7 | 55.4 | 48.8 | 46.8 |  | 126.9 | 34.4 | 52.2 |  |
| ID66970 | 104.7 | 36.4 | 39.2 | 66.2 | 74.6 | 46.9 |  | 133.4 | 54.8 | 38.0 |  |
| ID66971 | 129.9 | 121.5 | 160.1 | 81.5 | 88.0 | 86.5 |  | 121.0 | 83.2 | 130.7 |  |
| ID66972 | 127.4 | 123.5 | 124.6 | 74.8 | 59.7 | 76.4 |  | 152.9 | 48.5 | 64.8 |  |
| ID66973 | 107.1 | 88.2 | 72.6 | 85.1 | 59.0 | 79.4 | 76.0 | 75.8 | 63.4 | 102.1 | 74.8 |
| ID66974 | 242.4 | 124.4 | 108.0 | 82.2 | 61.1 | 62.5 |  | 121.1 | 59.9 | 80.0 |  |
| ID66975 | 49.9 | 11.3 | 102.3 | 67.9 | 46.1 | 53.7 | 99.9 | 98.3 | 88.9 | 92.5 | 119.2 |
| ID66976 | 109.9 | 113.5 | 167.3 | 74.8 | 79.3 | 62.1 |  | 122.2 | 85.2 | 93.8 |  |
| ID66977 | 107.7 | 155.7 | 304.8 | 67.9 | 23.8 | 37.8 | 80.7 | 72.7 | 82.6 | 95.5 | 100.1 |
| ID66979 | 77.6 | 107.9 | 119.2 | 96.1 | 78.8 | 58.5 |  | 127.7 | 90.0 | 116.5 |  |
| ID66980 | 182.1 | 522.2 |  | 101.6 | 91.0 | 87.8 |  | 101.5 | 64.6 |  |  |
| ID66981 | 443.1 | 182.8 | 324.0 | 271.2 | 191.5 | 154.4 | 196.5 | 424.1 | 168.3 | 212.8 | 235.9 |
| ID66982 | 61.3 | 201.2 | 0.0 | 63.6 | 103.2 | 142.1 |  | 86.3 | 68.5 | 400.2 |  |
| ID66983 | 213.5 | 99.7 | 57.7 | 93.5 | 62.7 | 76.8 |  | 163.8 | 53.0 | 67.1 |  |
| ID66984 | 80.9 | 134.5 | 258.4 | 72.7 | 68.8 | 58.7 |  | 111.6 | 82.0 | 75.3 |  |
| ID66985 | 450.2 | 255.6 | 232.6 | 23.9 | 17.6 | 60.8 |  | 20.1 | 17.1 | 23.9 |  |
| ID66987 | 260.7 | 251.9 | 232.9 | 19.4 | 18.1 | 46.1 |  | 27.9 | 16.8 | 49.3 |  |
| ID66988 | 93.2 | 22.6 |  | 54.8 | 82.1 | 82.1 |  | 69.7 | 44.1 |  |  |
| ID66989 | 139.8 | 138.9 | 0.0 | 77.6 | 76.6 | 134.1 |  | 100.9 | 66.7 | 0.0 |  |
| ID66990 | 12.0 | 192.6 |  | 12.1 | 33.1 | 1571.6 |  | 134.6 | 12.1 |  |  |
| ID66991 | 64.2 | 117.5 |  | 118.8 | 111.2 | 88.8 |  | 138.4 | 113.1 |  |  |
| ID66992 | 24.5 | 18.3 | 32.5 | 167.8 | 165.2 | 171.1 | 102.4 | 144.4 | 138.0 | 115.5 | 178.3 |
| ID67000 | 86.9 | 63.9 | 63.2 | 123.9 | 86.0 | 101.4 | 106.5 | 126.5 | 76.3 | 85.8 | 99.9 |
| ID67005 | 36.9 | 35.7 | 87.8 | 51.7 | 81.6 | 111.5 | 42.7 | 145.9 | 160.2 | 116.6 | 262.4 |
| ID67007 | 68.8 | 75.4 | 73.1 | 73.3 | 106.5 | 113.4 | 89.3 | 91.4 | 82.2 | 82.1 | 122.5 |
| ID67008 | 68.7 | 67.6 | 117.9 | 37.1 | 95.1 | 126.1 | 68.5 | 108.3 | 81.1 | 58.5 | 153.4 |
| ID67009 | 60.3 | 64.8 | 86.0 | 68.8 | 96.2 | 104.4 | 70.7 | 74.2 | 74.9 | 70.4 | 120.8 |

^a^ Due to limited space, only age-associated spot ID’s (spots highlighted in yellow) are shown on the 2D-DIGE image presented in Figure 1 . Should you require information on the location on 2-D-DIGE Image of other spot ID’s in this table, please contact the corresponding author

**Reference**

[1]	Sikulu MT, Monkman J, Dave KA, Hastie ML, Dale PE, Kitching RL, Killeen GF, Kay BH, Gorman JJ, Hugo L**E**. Proteomic changes occurring in the malaria mosquitoes Anopheles gambiae and Anopheles stephensi during aging. Journal of Proteomi*c*s 2015; 126:234-44.
