# Supplementary material for: Mass spectrometry identification of age-associated proteins from the malaria mosquitoes Anopheles gambiae s.s. and Anopheles stephensi
Source: Data Brief. 2015 Jul 16;4:461–7. doi: 10.1016/j.dib.2015.07.007 (PMC4534588; doi:10.1016/j.dib.2015.07.007)
Supplement: Supplementary file 1 — Supplementary material: Supplementary Table 1: Normalised spot volume data for all spots detected on 2D-DIGE images of cohort 1 of A. gambiae. Spots highlighted in yellow were found to be age-associated. Supplementary Table 2: Normalised spot volume data for all spots detected on 2D-DIGE images of cohort 2 of A. gambiae. Spots highlighted in yellow were found to be age-associated. Supplementary Table 3: Normalised spot volume data for all spots detected on 2D-DIGE images of cohort 1 of A. stephensi. Spots highlighted in yellow were found to be age-associated. Supplementary Table 4: Normalised spot volume data for all spots detected on 2D-DIGE images of cohort 2 of A. stephensi. Spots highlighted in yellow were found to be age-associated. External repository data: The mass spectrometry proteomics data have been deposited to the ProteomeXchange Consortium [8] via the PRIDE partner repository with the dataset identifier PXD002153 and 10.6019/PXD002153. [file mmc1.zip › Supplimentary file/Supplementary Table 3.doc]

Table 3

| **Spot ID^a^** | **Normalized Vol. D1a** | **Normalized Vol. D1c** | **Normalized Vol. D1b** | **Normalized Vol. D9b** | **Normalized Vol. D9a** | **Normalized Vol. D9c** | **Normalized Vol. D17b** | **Normalized Vol. D17c** | **Normalized Vol. D17a** | **Normalized Vol. D34a** | **Normalized Vol. D34b** | **Normalized Vol. D34c** |
| --- | --- | --- | --- | --- | --- | --- | --- | --- | --- | --- | --- | --- |
| ID1 | 127.8 | 152.0 | 78.8 | 182.0 | 79.0 | 153.5 | 96.4 | 98.9 | 130.3 | 77.0 | 140.5 | 319.3 |
| ID10 | 104.5 | 106.3 | 69.0 | 95.8 | 84.7 | 76.2 | 101.3 | 96.7 | 71.3 | 80.5 | 91.7 | 86.9 |
| ID100 | 59.5 | 83.6 | 76.0 | 114.8 | 66.5 | 95.1 | 91.7 | 74.6 | 60.2 | 164.9 | 134.7 | 87.8 |
| ID105 | 113.8 | 147.5 | 125.3 | 178.2 | 63.8 | 105.1 | 123.3 | 112.9 | 156.0 | 74.5 | 135.6 | 144.7 |
| ID108 | 29.4 | 54.3 | 51.5 | 82.8 | 88.5 | 41.2 | 80.3 | 67.5 | 60.5 | 151.0 | 203.8 | 237.6 |
| ID92 | 70.3 | 103.4 | 93.0 | 98.2 | 84.3 | 72.3 | 48.3 | 68.7 | 77.6 | 94.1 | 104.5 | 124.0 |
| ID109 | 51.1 | 23.9 | 54.4 | 41.0 | 54.0 | 64.9 | 415.4 | 101.8 | 49.5 | 152.0 | 179.0 | 89.1 |
| ID11 | 95.2 | 123.9 | 85.7 |  | 79.2 | 85.2 |  | 83.7 | 106.9 | 65.2 | 136.6 | 95.5 |
| ID110 | 32.8 | 36.0 | 64.0 | 53.4 | 52.3 | 66.0 | 145.2 | 49.9 | 45.5 | 182.3 | 149.5 | 116.6 |
| ID111 | 119.5 | 155.0 | 153.1 | 367.9 | 83.5 | 115.9 | 212.0 | 75.2 | 172.0 | 87.3 | 182.4 | 144.0 |
| ID112 | 30.8 | 60.7 | 0.0 | 4.0 | 44.7 | 0.4 | 738.7 | 75.3 | 0.4 | 367.1 | 472.7 | 288.1 |
| ID113 | 8.2 | 49.6 | 42.0 | 32.3 | 18.8 | 37.5 | 266.8 | 89.6 | 39.9 | 210.3 | 209.8 | 106.7 |
| ID114 | 62.2 | 74.2 | 49.5 | 22.8 | 60.2 | 54.6 | 430.0 | 48.3 | 59.0 | 146.7 | 178.6 | 112.7 |
| ID115 | 38.5 | 90.1 | 37.7 | 25.7 | 45.6 | 53.4 | 416.6 | 95.1 | 50.9 | 173.4 | 211.2 | 104.7 |
| ID116 | 137.8 | 95.1 | 143.1 | 454.1 | 104.0 | 86.4 | 126.3 | 107.3 | 134.1 | 102.5 | 162.2 | 87.4 |
| ID117 | 124.5 | 98.9 | 153.8 | 576.4 | 105.8 | 86.0 | 136.3 | 113.2 | 79.8 | 60.8 | 155.6 | 88.7 |
| ID118 | 44.2 | 84.8 | 98.7 | 75.7 | 70.8 | 103.3 | 127.5 | 73.8 | 89.0 | 93.5 | 87.7 | 108.1 |
| ID119 | 44.7 | 84.2 | 76.9 | 86.6 | 61.2 | 76.5 | 133.9 | 71.5 | 51.0 | 212.1 | 148.7 | 119.2 |
| ID12 | 113.8 | 130.4 | 87.8 | 154.8 | 79.6 | 75.7 | 97.9 | 87.0 | 92.8 | 69.2 | 136.6 | 163.3 |
| ID120 | 33.8 | 47.1 | 38.2 | 31.2 | 48.1 | 38.2 | 219.5 | 58.1 | 85.0 | 132.9 | 249.4 | 194.9 |
| ID121 | 59.4 | 70.1 | 51.8 | 115.0 | 77.5 | 51.9 | 60.6 | 84.2 | 96.8 | 100.2 | 166.2 | 262.1 |
| ID122 | 63.3 | 87.1 | 62.0 | 96.0 | 68.1 | 77.0 | 111.7 | 74.3 | 94.4 | 66.9 | 127.9 | 153.5 |
| ID123 | 40.2 | 59.8 | 54.8 | 57.4 | 64.9 | 62.8 | 171.5 | 71.3 | 53.5 | 196.7 | 188.0 | 138.0 |
| ID124 | 73.4 | 151.8 | 137.6 | 74.8 | 103.3 | 65.0 | 137.7 | 102.4 | 64.2 | 169.9 | 230.0 | 155.1 |
| ID125 | 98.0 | 85.9 | 98.5 | 79.3 | 76.3 | 90.5 | 98.8 | 80.6 | 102.6 | 74.6 | 109.2 | 143.4 |
| ID126 | 101.7 | 104.9 | 99.2 | 81.7 | 76.8 | 108.1 | 96.9 | 73.6 | 123.2 | 58.1 | 140.9 | 157.5 |
| ID127 | 129.2 | 161.4 | 93.9 | 201.4 | 77.1 | 92.4 | 106.8 | 88.7 | 147.9 | 89.2 | 149.0 | 134.4 |
| ID128 | 91.4 | 126.5 | 124.4 | 60.4 | 84.8 | 55.9 | 186.2 | 66.0 | 40.4 | 222.7 | 235.3 | 145.6 |
| ID129 | 204.6 | 137.5 | 92.4 | 237.3 | 117.3 | 81.8 | 94.9 | 83.1 | 151.7 | 85.5 | 94.6 | 104.7 |
| ID13 | 128.0 | 76.5 | 104.8 | 96.3 | 120.4 | 92.0 | 54.9 | 121.0 | 181.5 | 34.8 | 77.6 | 132.2 |
| ID130 | 33.4 | 138.1 | 62.2 | 18.9 | 23.4 | 62.2 | 345.6 | 81.7 | 48.5 | 139.1 | 155.6 | 98.3 |
| ID131 | 143.3 | 89.6 | 120.4 | 411.3 | 119.4 | 94.3 | 137.1 | 99.4 | 197.3 | 94.5 | 117.9 | 98.6 |
| ID132 | 126.6 | 91.5 | 111.1 | 237.7 | 101.3 | 99.5 | 115.3 | 101.5 | 273.4 | 99.2 | 138.8 | 116.4 |
| ID133 | 104.3 | 87.9 | 110.9 | 152.6 | 104.2 | 96.8 | 72.7 | 110.8 | 263.4 | 167.2 | 300.2 | 108.0 |
| ID134 | 59.7 | 63.7 | 41.9 | 38.0 | 45.4 | 65.5 | 317.3 | 79.5 | 51.0 | 225.3 | 176.4 | 143.1 |
| ID135 | 118.9 | 89.4 | 131.8 | 428.0 | 116.5 | 108.6 | 86.8 | 103.9 | 254.9 | 136.0 | 161.4 | 93.9 |
| ID136 | 90.0 | 159.3 | 149.6 | 161.0 | 117.5 | 132.7 | 47.3 | 118.0 | 124.5 | 92.6 | 72.8 | 68.6 |
| ID137 | 83.6 | 29.8 | 28.4 | 7.0 | 24.6 | 10.6 | 307.6 | 7.7 | 8.2 | 286.2 | 330.9 | 181.4 |
| ID138 | 44.1 | 47.8 | 45.6 | 37.3 | 75.3 | 37.2 | 143.6 | 81.0 | 37.0 | 213.7 | 193.7 | 225.6 |
| ID139 | 70.9 | 115.1 | 68.2 | 34.7 | 48.9 | 28.1 | 301.1 | 42.4 | 21.0 | 279.4 | 362.2 | 145.0 |
| ID14 | 138.9 | 82.8 | 16.3 | 11.3 | 114.0 | 35.1 | 319.4 | 100.0 | 0.0 | 79.6 | 148.8 | 121.5 |
| ID140 | 145.7 | 40.5 | 71.9 | 27.0 | 9.0 | 17.9 | 330.2 | 1.8 | 11.5 | 341.9 | 270.7 | 117.2 |
| ID141 | 155.8 | 184.3 | 219.4 | 171.0 | 121.3 | 141.9 | 20.9 | 138.9 | 408.8 | 11.2 | 91.5 | 96.5 |
| ID142 | 81.7 | 22.3 | 30.4 | 14.1 | 51.1 | 8.1 | 208.4 | 31.7 | 12.9 | 243.5 | 250.5 | 270.9 |
| ID143 | 93.0 | 12.9 | 68.1 | 12.2 | 1.3 | 3.8 | 671.7 | 0.9 | 0.1 | 597.9 | 260.5 | 110.3 |
| ID144 | 102.3 | 129.7 | 142.7 | 216.8 | 114.3 | 161.6 | 25.6 | 130.1 | 197.1 | 38.0 | 24.1 | 39.7 |
| ID145 | 122.3 | 75.0 | 110.9 | 117.9 | 142.9 | 102.2 | 56.1 | 88.7 | 92.8 | 70.3 | 85.1 | 159.0 |
| ID146 | 60.4 | 52.3 | 52.6 | 57.0 | 108.5 | 46.1 | 79.2 | 119.6 | 53.7 | 158.4 | 135.3 | 208.3 |
| ID147 | 190.3 | 19.4 | 205.5 | 49.5 | 85.4 | 35.7 | 98.7 | 26.9 | 48.9 | 51.8 | 59.9 | 217.9 |
| ID148 | 198.6 | 40.5 | 201.0 | 69.8 | 52.6 | 78.6 | 164.0 | 76.7 | 45.2 | 121.2 | 86.9 | 49.1 |
| ID149 | 170.1 | 56.1 | 92.1 | 39.4 | 203.9 | 79.2 | 99.8 | 159.9 | 51.3 | 170.5 | 134.2 | 402.5 |
| ID15 | 95.2 | 111.6 | 72.5 | 97.3 | 84.5 | 79.4 | 85.7 | 85.9 | 86.3 | 62.9 | 94.2 | 80.2 |
| ID150 | 110.7 | 127.4 | 101.5 | 89.9 | 124.2 | 117.7 | 11.9 | 145.8 | 98.5 | 27.0 | 47.7 | 77.6 |
| ID151 | 31.2 | 20.7 | 66.9 | 4.7 | 22.8 | 1.9 | 177.9 | 7.7 | 10.1 | 189.9 | 248.7 | 423.2 |
| ID152 | 77.7 | 218.8 | 48.4 | 20.3 | 81.5 | 39.6 | 146.5 | 152.1 | 45.1 | 203.0 | 190.2 | 305.6 |
| ID153 | 100.1 | 30.9 | 2.1 | 13.7 | 132.5 | 24.7 | 469.4 | 13.4 | 0.4 | 479.9 | 465.9 | 124.2 |
| ID154 | 405.6 | 101.4 | 100.1 | 43.2 | 42.5 | 8.1 | 1069.6 | 3.6 | 67.0 | 252.4 | 212.0 | 69.0 |
| ID155 | 73.9 | 108.7 | 71.5 | 32.9 | 39.3 | 31.8 | 302.1 | 60.4 | 23.1 | 243.2 | 328.4 | 116.2 |
| ID156 | 70.3 | 203.3 | 54.7 | 34.3 | 47.7 | 15.1 | 1839.6 | 137.6 | 30.1 | 308.3 | 228.8 | 288.6 |
| ID156948 | 117.0 | 148.2 | 138.8 | 170.6 | 132.4 | 161.3 | 146.0 | 128.6 | 132.9 | 159.2 | 138.5 | 88.3 |
| ID157 | 70.2 | 63.4 | 58.3 | 40.8 | 144.9 | 62.1 | 48.2 | 157.2 | 57.8 | 88.8 | 100.7 | 199.0 |
| ID158 | 88.4 | 90.8 | 72.6 | 49.4 | 127.2 | 64.2 | 54.1 | 157.3 | 61.1 | 88.6 | 101.6 | 182.6 |
| ID158727 | 98.4 | 59.2 | 73.9 | 45.8 | 41.0 | 52.3 | 289.9 | 39.6 | 65.9 | 106.0 | 190.0 | 97.8 |
| ID158781 | 120.4 | 62.0 | 59.9 | 67.3 | 66.6 | 52.6 | 212.2 | 48.9 | 59.7 | 128.9 | 151.3 | 91.2 |
| ID158809 | 160.7 | 67.9 | 93.7 | 74.6 | 78.6 | 78.2 | 187.5 | 77.8 | 74.9 | 65.3 | 93.5 | 99.1 |
| ID158832 | 4.6 | 20.8 | 30.8 | 33.9 | 25.4 | 63.4 | 445.0 | 120.9 | 46.9 | 216.9 | 195.0 | 169.5 |
| ID158880 | 130.2 | 130.1 | 134.7 | 121.0 | 109.2 | 144.3 | 30.3 | 114.8 | 108.7 | 33.1 | 36.1 | 56.1 |
| ID159 | 128.8 | 101.8 | 86.1 | 68.8 | 107.8 | 81.0 | 47.9 | 138.5 | 66.5 | 86.1 | 73.8 | 144.2 |
| ID159259 | 111.8 | 90.5 | 101.2 | 75.5 | 144.2 | 84.3 | 88.9 | 133.2 | 70.7 | 104.0 | 95.0 | 123.8 |
| ID159638 | 65.3 | 47.4 | 55.4 | 29.0 | 110.1 | 35.2 | 231.3 | 100.0 | 29.6 | 234.1 | 178.2 | 192.0 |
| ID16 | 100.3 | 98.7 | 90.5 | 103.7 | 74.5 | 199.4 | 86.6 | 69.6 | 92.1 | 58.7 | 147.0 | 64.7 |
| ID160 | 137.0 | 160.7 | 112.8 | 95.5 | 116.2 | 120.7 | 17.8 | 115.8 | 92.2 | 38.1 | 38.6 | 66.1 |
| ID160015 | 35.6 | 35.0 | 40.1 | 22.2 | 48.7 | 28.0 | 437.2 | 51.8 | 21.8 | 300.9 | 261.1 | 144.2 |
| ID160610 | 69.6 | 71.7 | 80.6 | 78.4 | 94.6 | 77.4 | 194.8 | 99.2 | 103.7 | 223.8 | 179.2 | 129.0 |
| ID160773 | 71.9 | 60.2 | 67.4 | 62.9 | 97.0 | 58.8 | 129.0 | 95.2 | 54.4 | 71.1 | 112.3 | 124.6 |
| ID160854 | 133.1 | 89.1 | 90.2 | 76.3 | 64.5 | 71.9 | 93.9 | 74.6 | 81.9 | 69.7 | 77.3 | 74.8 |
| ID161 | 133.6 | 155.5 | 155.6 | 159.8 | 82.8 | 187.3 | 7.1 | 87.8 | 137.5 | 12.0 | 6.3 | 26.1 |
| ID161072 | 18.6 | 34.5 | 52.0 | 30.9 | 26.9 | 26.5 | 396.3 | 56.7 | 5.1 | 297.9 | 256.3 | 357.7 |
| ID161269 | 145.0 | 120.4 | 162.5 | 120.0 | 72.5 | 99.3 | 180.2 | 41.1 | 78.0 | 177.6 | 103.5 | 61.3 |
| ID161340 | 39.1 | 37.8 | 126.3 | 51.8 | 74.4 | 45.1 | 308.0 | 42.6 | 25.9 | 304.0 | 94.8 | 309.6 |
| ID161348 | 29.9 | 44.8 | 52.2 | 44.6 | 44.5 | 49.4 | 233.3 | 54.5 | 25.7 | 264.4 | 186.2 | 122.5 |
| ID161389 | 33.3 | 21.5 | 30.6 | 33.8 | 39.2 | 40.8 | 267.0 | 30.4 | 57.1 | 142.6 | 219.8 | 108.0 |
| ID161407 | 106.4 | 38.9 | 130.8 | 637.9 | 87.7 | 168.5 | 0.0 | 71.4 | 98.4 | 53.1 | 21.9 | 56.9 |
| ID161439 | 166.5 | 73.1 | 254.9 | 164.5 | 72.0 | 171.6 | 24.3 | 42.0 | 91.8 | 83.2 | 0.0 | 34.7 |
| ID161482 | 38.2 | 39.4 | 48.9 | 55.4 | 48.9 | 42.9 | 206.5 | 47.0 | 23.6 | 278.1 | 224.9 | 141.9 |
| ID161550 | 50.0 | 34.9 | 54.2 | 32.2 | 49.2 | 70.4 | 295.3 | 36.4 | 87.3 | 102.6 | 191.3 | 104.3 |
| ID161848 | 40.1 | 25.0 | 49.6 | 51.6 | 28.3 | 28.5 | 347.5 | 17.8 | 7.5 | 238.8 | 286.4 | 112.6 |
| ID162 | 81.5 | 74.0 | 79.7 | 93.7 | 132.1 | 67.8 | 45.1 | 159.6 | 74.8 | 107.7 | 72.8 | 146.7 |
| ID162103 | 47.8 | 11.3 | 36.7 | 23.9 | 29.6 | 12.9 | 461.2 | 8.8 | 10.2 | 327.6 | 353.8 | 114.9 |
| ID162456 | 111.4 | 107.6 | 122.1 | 134.3 | 146.0 | 138.5 | 76.5 | 123.6 | 129.5 | 71.5 | 66.7 | 70.6 |
| ID162512 | 119.5 | 92.1 | 108.5 | 139.3 | 154.5 | 148.8 | 73.8 | 113.8 | 134.9 | 73.6 | 64.9 | 74.2 |
| ID162726 | 158.7 | 129.5 | 136.9 | 112.5 | 104.3 | 101.2 | 60.2 | 84.4 | 108.7 | 69.0 | 65.1 | 70.9 |
| ID163 | 109.6 | 148.2 | 151.8 | 231.3 | 107.5 | 166.0 | 28.2 | 110.0 | 219.8 | 56.7 | 46.2 | 63.1 |
| ID163271 | 92.2 | 46.9 | 1.8 | 33.9 | 108.1 | 16.5 | 108.2 | 11.2 | 52.0 | 11.5 | 26.6 | 21.9 |
| ID163655 | 94.1 | 87.0 | 86.7 | 87.2 | 83.4 | 82.6 | 105.2 | 77.2 | 88.6 | 96.9 | 89.0 | 85.3 |
| ID164512 | 155.9 | 152.1 | 170.9 | 156.9 | 149.7 | 165.5 | 31.2 | 121.4 | 162.4 | 33.4 | 32.3 | 44.8 |
| ID163888, | 114.5 | 99.8 | 104.9 | 78.9 | 93.2 | 111.3 | 141.5 | 81.8 | 61.5 | 274.0 | 127.4 | 88.8 |
| ID164 | 118.7 | 115.8 | 169.5 | 320.7 | 137.5 | 132.2 | 52.1 | 113.5 | 380.9 | 39.7 | 57.1 | 71.9 |
| ID164198 | 249.3 | 209.5 | 259.2 |  | 42.4 |  | 8.2 | 110.9 | 85.9 | 14.9 | 24.8 |  |
| ID165 | 168.4 | 127.8 | 72.9 | 103.4 | 135.6 | 140.5 | 609.0 | 108.3 | 93.6 | 296.7 | 393.4 | 103.9 |
| ID1666 | 109.8 | 133.7 | 133.8 | 145.1 | 170.9 | 117.4 | 132.4 | 128.6 | 147.1 | 134.8 | 130.0 | 120.0 |
| ID167 | 86.8 | 137.8 | 92.7 | 181.9 | 65.1 | 61.9 | 216.7 | 125.7 | 81.4 | 287.4 | 424.5 | 124.5 |
| ID168 | 94.3 | 93.2 | 107.5 | 131.3 | 129.7 | 106.4 | 23.7 | 160.0 | 106.8 | 62.2 | 37.8 | 87.2 |
| ID169 | 56.5 | 92.9 | 134.1 | 54.7 | 59.4 | 40.5 | 169.3 | 43.1 | 45.9 | 208.5 | 247.5 | 155.0 |
| ID17 | 160.3 | 111.5 | 135.2 | 66.0 | 134.6 | 101.6 | 100.2 | 112.8 | 113.7 | 97.6 | 51.3 | 98.8 |
| ID170 | 44.2 | 127.0 | 12.0 | 83.8 | 23.1 | 9.2 | 123.2 | 135.5 | 30.2 | 294.5 | 334.9 | 205.1 |
| ID171 | 149.5 | 108.0 | 82.7 | 148.9 | 102.4 | 86.1 | 76.3 | 94.8 | 124.3 | 71.9 | 111.8 | 115.2 |
| ID172 | 142.0 | 128.3 | 74.5 | 185.0 | 98.1 | 76.2 | 74.9 | 95.1 | 160.4 | 78.1 | 168.8 | 138.7 |
| ID1729 | 54.1 | 36.7 | 41.4 | 51.9 | 55.7 | 27.9 | 205.4 | 52.2 | 17.2 | 235.2 | 191.8 | 131.9 |
| ID173 | 158.2 | 123.0 | 77.3 | 160.1 | 101.8 | 83.6 | 75.3 | 105.6 | 126.4 | 73.4 | 132.2 | 129.3 |
| ID174 | 77.8 | 104.2 | 83.5 | 120.9 | 97.9 | 97.0 | 69.5 | 105.1 | 101.9 | 76.3 | 161.1 | 191.7 |
| ID175 | 99.2 | 119.4 | 124.9 | 151.7 | 105.8 | 133.0 | 16.7 | 127.4 | 122.8 | 34.3 | 26.4 | 40.5 |
| ID1750 | 41.5 | 55.7 | 65.8 | 62.4 | 60.6 | 65.8 | 183.5 | 88.3 | 55.3 | 247.8 | 185.7 | 181.3 |
| ID176 | 106.7 | 174.3 | 112.3 | 127.3 | 92.1 | 91.9 | 52.5 | 57.2 | 112.4 | 68.1 | 94.4 | 100.8 |
| ID177 | 167.7 | 170.7 | 192.6 | 149.3 | 98.0 | 101.0 | 35.5 | 90.7 | 118.9 | 59.2 | 58.2 | 77.5 |
| ID1777 | 75.3 | 112.9 | 95.9 | 126.2 | 117.0 | 79.2 | 82.9 | 84.7 | 98.5 | 80.7 | 71.0 | 67.5 |
| ID178 | 347.9 | 158.7 | 195.0 | 57.5 | 29.3 | 49.9 | 13.9 | 5.8 | 74.2 | 27.6 | 38.1 | 16.5 |
| ID179 | 266.8 | 201.9 | 245.0 | 117.2 | 49.3 | 101.6 | 9.2 | 31.1 | 94.7 | 2.2 | 47.0 | 13.6 |
| ID1795 | 177.0 | 99.0 | 116.6 | 86.4 | 100.1 | 76.1 | 130.0 | 76.5 | 94.7 | 112.2 | 109.1 | 97.6 |
| ID18 | 169.5 | 93.5 | 133.9 | 62.8 | 128.0 | 92.2 | 105.0 | 168.3 | 124.7 | 91.4 | 43.6 | 97.5 |
| ID180 | 183.7 | 127.7 | 106.8 | 172.0 | 70.0 | 99.1 | 63.2 | 97.6 | 198.6 | 80.1 | 165.1 | 187.4 |
| ID181 | 122.7 | 91.6 | 86.7 | 128.1 | 80.3 | 91.5 | 65.8 | 80.9 | 107.1 | 68.4 | 102.2 | 99.5 |
| ID182 | 215.2 | 166.2 | 222.0 | 102.8 | 88.9 | 99.8 | 5.6 | 74.2 | 99.7 | 12.8 | 28.2 | 39.3 |
| ID1828 | 190.1 | 142.5 | 141.4 | 106.2 | 83.9 | 105.7 | 123.1 | 92.4 | 109.1 | 87.2 | 85.4 | 94.1 |
| ID183 | 75.5 | 168.2 | 93.4 | 98.6 | 92.7 | 76.5 | 90.9 | 107.7 | 123.8 | 8.3 | 108.1 | 118.8 |
| ID184 | 71.0 | 102.2 | 64.1 | 101.5 | 78.5 | 72.3 | 120.9 | 121.4 | 79.8 | 134.5 | 211.7 | 256.0 |
| ID185 | 124.9 | 139.1 | 134.0 | 167.2 | 113.2 | 151.4 | 62.5 | 126.1 | 154.0 | 38.4 | 51.8 | 48.9 |
| ID1858 | 179.8 | 124.2 | 123.0 | 114.6 | 89.5 | 82.9 | 126.1 | 92.9 | 124.6 | 106.5 | 97.6 | 98.7 |
| ID186 | 210.7 | 174.8 | 166.0 | 118.1 | 86.1 | 174.5 | 7.2 | 99.8 | 114.3 | 7.8 | 8.4 | 33.1 |
| ID187 | 175.7 | 150.7 | 153.8 | 93.6 | 110.0 | 85.0 | 1.4 | 117.5 | 99.2 | 20.7 | 63.1 | 106.6 |
| ID188 | 67.2 | 52.1 | 78.8 | 35.4 | 34.2 | 30.8 | 235.5 | 32.6 | 32.2 | 217.9 | 288.1 | 124.5 |
| ID1884 | 121.5 | 112.4 | 124.5 | 117.9 | 113.9 | 112.4 | 110.5 | 101.8 | 125.5 | 128.7 | 139.1 | 121.0 |
| ID189 | 64.2 | 71.0 | 101.5 | 57.2 | 64.6 | 36.9 | 166.2 | 44.4 | 58.9 | 194.5 | 241.9 | 162.1 |
| ID19 | 159.0 | 104.2 | 102.2 | 67.3 | 124.5 | 135.9 | 93.7 | 134.6 | 116.5 | 109.8 | 88.6 | 104.1 |
| ID190 | 96.4 | 93.6 | 81.8 | 101.1 | 140.4 | 90.1 | 79.5 | 156.6 | 109.8 | 78.5 | 94.9 | 111.8 |
| ID191 | 73.4 | 102.5 | 88.8 | 94.0 | 83.2 | 106.2 | 111.5 | 121.0 | 114.6 | 116.5 | 142.6 | 149.7 |
| ID192 | 139.7 | 73.9 | 246.6 | 94.0 | 82.2 | 161.7 | 122.1 | 107.9 | 180.1 | 162.2 | 156.7 | 62.8 |
| ID1920 | 114.2 | 112.0 | 136.4 | 102.8 | 86.7 | 104.5 | 107.0 | 97.0 | 107.7 | 129.4 | 118.5 | 94.8 |
| ID193 | 61.1 | 119.0 | 101.7 | 38.0 | 72.6 | 95.6 | 126.8 | 136.5 | 117.5 | 220.4 | 174.1 | 120.5 |
| ID194 | 171.7 | 198.0 | 176.5 | 128.4 | 141.2 | 99.6 | 28.2 | 56.5 | 144.1 | 21.3 | 8.8 | 59.8 |
| ID196 | 125.0 | 102.7 | 99.0 | 106.7 | 84.5 | 98.3 | 61.5 | 67.6 | 103.8 | 78.8 | 76.4 | 72.1 |
| ID197 | 121.8 | 179.5 | 173.8 | 111.1 | 122.2 | 100.1 | 51.1 | 100.2 | 115.6 | 51.3 | 51.2 | 70.1 |
| ID198 | 56.2 | 51.9 | 42.8 | 52.5 | 90.4 | 44.4 | 187.6 | 97.9 | 63.7 | 169.8 | 215.3 | 169.6 |
| ID199 | 127.6 | 135.9 | 114.8 | 109.3 | 101.5 | 107.5 | 58.4 | 94.3 | 100.5 | 67.4 | 58.7 | 70.4 |
| ID1991 | 141.8 | 134.3 | 156.8 | 109.6 | 105.7 | 103.6 | 82.3 | 94.4 | 97.0 | 69.7 | 78.3 | 82.0 |
| ID2 | 118.0 | 106.1 | 87.5 |  | 81.1 | 103.3 |  | 70.2 | 112.8 | 91.3 | 144.5 | 172.4 |
| ID20 | 161.3 | 162.6 | 84.4 | 128.4 | 122.3 | 164.0 | 77.1 | 156.0 | 170.0 | 39.5 | 101.4 | 135.8 |
| ID200 | 45.9 | 30.2 | 57.6 | 43.2 | 41.5 | 28.4 | 278.2 | 48.2 | 30.6 | 257.3 | 249.5 | 168.2 |
| ID201 | 154.7 | 89.1 | 72.2 | 79.1 | 75.1 | 57.5 | 91.5 | 50.5 | 67.9 | 111.4 | 145.4 | 108.1 |
| ID202 | 129.1 | 151.4 | 127.0 | 110.5 | 107.6 | 103.2 | 65.8 | 93.1 | 100.3 | 85.4 | 70.9 | 75.5 |
| ID203 | 88.2 | 146.0 | 135.7 | 116.1 | 85.7 | 104.0 | 66.1 | 93.1 | 106.3 | 77.3 | 73.2 | 78.1 |
| ID204 | 113.6 | 56.1 | 75.7 | 26.9 | 34.7 | 25.3 | 449.0 | 25.1 | 56.8 | 279.7 | 228.9 | 161.9 |
| ID2047 | 73.2 | 93.4 | 86.1 | 110.3 | 112.6 | 96.2 | 90.7 | 102.3 | 102.9 | 113.6 | 128.8 | 108.0 |
| ID205 | 105.2 | 488.6 | 214.1 | 192.4 | 138.8 | 181.7 | 27.6 | 282.0 | 206.2 | 85.6 | 130.4 | 249.3 |
| ID206 | 386.0 | 209.3 | 120.6 | 120.3 | 36.2 | 95.8 | 24.0 | 70.5 | 39.0 | 23.9 | 354.9 | 278.9 |
| ID207 | 59.4 | 229.9 | 257.0 | 288.8 | 127.9 | 152.2 | 19.2 | 159.0 | 212.7 | 42.3 | 6.5 | 63.0 |
| ID208 | 78.1 | 92.7 | 91.8 | 83.2 | 102.1 | 98.0 | 132.9 | 116.1 | 91.7 | 120.1 | 127.5 | 117.7 |
| ID209 | 109.9 | 167.3 | 179.6 | 136.9 | 104.1 | 111.5 | 43.0 | 88.9 | 75.6 | 100.1 | 66.3 | 82.8 |
| ID21 | 82.9 | 51.0 | 39.4 | 58.0 | 85.2 | 30.1 | 127.8 | 82.7 | 67.7 | 63.3 | 115.7 | 142.8 |
| ID212 | 243.1 | 128.6 | 151.7 | 92.1 | 51.3 | 73.1 | 30.1 | 65.2 | 40.8 | 32.1 | 103.5 | 131.1 |
| ID213 | 98.5 | 83.6 | 241.1 | 93.7 | 94.1 | 112.4 | 89.4 | 104.9 | 160.6 | 20.5 | 30.7 | 72.1 |
| ID214 | 93.0 | 96.3 | 105.7 | 49.9 | 49.5 | 32.3 | 216.8 | 41.5 | 40.2 | 198.9 | 247.9 | 129.5 |
| ID215 | 16.2 | 183.9 | 43.0 | 75.0 | 29.5 | 35.8 | 290.9 | 134.1 | 54.7 | 307.8 | 302.1 | 162.0 |
| ID216 | 189.8 | 50.1 | 79.2 | 40.3 | 35.1 | 84.4 | 176.0 | 108.8 | 55.6 | 111.1 | 209.4 | 185.7 |
| ID217 | 64.0 | 158.8 | 159.0 | 167.1 | 113.5 | 106.6 | 33.8 | 104.7 | 148.3 | 49.4 | 63.5 | 83.3 |
| ID2175 | 119.4 | 135.4 | 133.2 | 148.0 | 123.5 | 112.4 | 59.7 | 94.9 | 114.3 | 68.5 | 92.2 | 84.7 |
| ID218 | 124.2 | 86.8 | 68.2 | 54.1 | 15.7 | 57.0 | 185.7 | 35.8 | 0.1 | 769.0 | 184.6 | 149.5 |
| ID2181 | 65.7 | 55.3 | 36.6 | 20.2 | 49.4 | 61.2 | 212.1 | 103.4 | 28.9 | 116.5 | 212.4 | 274.7 |
| ID2188 | 58.4 | 42.1 | 27.4 | 0.0 | 25.8 | 10.3 | 627.6 | 37.6 | 0.0 | 338.0 | 212.2 | 129.7 |
| ID219 | 104.9 | 202.5 | 124.1 | 89.7 | 132.3 | 198.6 | 138.6 | 176.6 | 216.7 | 46.6 | 84.4 | 73.1 |
| ID22 | 157.3 | 160.9 | 115.0 | 74.3 | 166.5 | 187.2 | 87.6 | 158.3 | 106.4 | 95.4 | 116.6 | 170.1 |
| ID220 | 607.8 | 224.7 | 186.1 | 84.0 | 40.9 | 58.8 | 20.6 | 70.6 | 129.7 | 51.0 | 54.5 | 116.9 |
| ID221 | 21.8 | 136.3 | 164.7 | 189.5 | 46.0 | 96.8 | 146.3 | 96.4 | 150.0 | 171.9 | 150.6 | 120.2 |
| ID222 | 107.6 | 67.6 | 160.5 | 50.2 | 85.1 | 99.6 | 205.7 | 47.4 | 94.4 | 218.2 | 305.1 | 200.8 |
| ID223 | 97.7 | 329.4 | 159.0 | 81.2 | 101.2 | 113.4 | 131.4 | 425.7 | 106.7 | 127.1 | 196.2 | 143.5 |
| ID224 | 89.5 | 100.2 | 89.8 | 79.1 | 106.8 | 87.1 | 133.5 | 155.9 | 106.4 | 84.2 | 105.9 | 119.3 |
| ID225 | 59.8 | 115.6 | 100.3 | 100.5 | 70.8 | 104.9 | 66.8 | 100.5 | 99.6 | 185.7 | 151.7 | 102.7 |
| ID226 | 97.1 | 210.4 | 108.9 | 238.3 | 143.2 | 116.7 | 64.4 | 156.9 | 193.8 | 121.2 | 181.5 | 125.9 |
| ID2271 | 17.7 | 14.8 | 18.7 | 4.6 | 10.7 | 8.4 | 201.1 | 20.4 | 4.3 | 367.4 | 354.6 | 148.7 |
| ID228 | 21.9 | 31.9 | 4.6 | 19.6 | 29.5 | 32.3 | 237.7 | 83.5 | 34.4 | 225.7 | 252.5 | 287.1 |
| ID230 | 0.0 | 10.7 | 4.1 | 79.6 | 3.3 | 78.3 | 129.3 | 105.2 | 87.7 | 100.4 | 168.6 | 144.2 |
| ID231 | 67.6 | 104.9 | 127.9 | 59.3 | 73.3 | 39.8 | 183.3 | 69.7 | 45.3 | 196.4 | 220.0 | 132.7 |
| ID232 | 0.7 | 1.6 | 14.7 | 2.1 | 12.1 | 26.7 | 268.2 | 74.9 | 6.9 | 201.3 | 220.7 | 208.2 |
| ID233 | 161.4 | 85.5 | 136.6 | 21.2 | 96.2 | 89.0 | 500.0 | 39.1 | 316.3 | 161.9 | 263.3 | 495.4 |
| ID2335 | 179.5 | 166.6 | 249.0 | 140.2 | 155.0 | 180.5 | 35.3 | 108.6 | 112.1 | 2.9 | 7.9 | 40.4 |
| ID234 | 127.0 | 124.8 | 125.3 | 64.4 | 98.1 | 152.8 | 157.7 | 108.7 | 144.8 | 26.2 | 31.0 | 33.6 |
| ID2346 | 58.3 | 70.6 | 87.0 | 63.5 | 72.2 | 82.4 | 153.3 | 106.9 | 88.5 | 128.1 | 131.0 | 144.1 |
| ID235 | 96.2 | 69.3 | 130.3 | 144.0 | 124.0 | 140.6 | 13.7 | 107.9 | 150.2 | 28.7 | 46.3 | 77.0 |
| ID236 | 38.3 | 18.3 | 294.7 | 187.6 | 29.1 | 129.6 | 250.4 | 10.3 | 327.0 | 347.6 | 425.9 | 440.1 |
| ID237 | 138.2 | 167.1 | 181.6 | 126.2 | 96.6 | 113.2 | 67.0 | 78.1 | 106.8 | 97.3 | 86.8 | 79.6 |
| ID238 | 67.6 | 233.8 | 142.7 | 303.6 | 182.5 | 171.6 | 412.7 | 127.1 | 207.5 | 166.5 | 307.3 | 92.0 |
| ID239 | 53.3 | 12.9 | 46.2 | 107.6 | 60.3 | 51.3 | 100.1 | 76.5 | 51.1 | 211.3 | 227.4 | 167.4 |
| ID24 | 79.8 | 108.8 | 98.7 | 106.1 | 59.3 | 90.7 | 95.4 | 67.4 | 87.5 | 34.7 | 57.5 | 73.8 |
| ID240 | 70.2 | 65.4 | 80.0 | 50.6 | 88.2 | 57.8 | 248.0 | 119.6 | 81.0 | 129.4 | 146.8 | 179.8 |
| ID241 | 3.5 | 41.6 | 86.7 | 76.3 | 15.9 | 69.5 | 327.2 | 22.5 | 93.7 | 325.7 | 369.1 | 213.5 |
| ID242 | 200.5 | 156.3 | 190.7 | 58.1 | 59.1 | 66.2 | 103.3 | 57.8 | 63.2 | 38.8 | 41.9 | 90.5 |
| ID243 | 67.1 | 56.9 | 77.0 | 70.4 | 87.5 | 79.4 | 155.6 | 64.6 | 72.7 | 127.8 | 145.3 | 133.7 |
| ID244 | 71.8 | 24.5 | 83.1 | 68.0 | 110.3 | 76.7 | 73.6 | 60.1 | 72.7 | 118.6 | 128.2 | 150.9 |
| ID245 | 24.8 | 22.5 | 26.1 | 50.4 | 52.1 | 35.5 | 200.4 | 64.7 | 35.0 | 241.3 | 267.4 | 213.9 |
| ID246 | 50.0 | 22.0 | 65.3 | 39.4 | 58.9 | 45.2 | 211.2 | 52.9 | 37.9 | 242.6 | 214.4 | 182.8 |
| ID247 | 95.3 | 49.4 | 105.4 | 76.5 | 0.0 | 83.1 | 29.2 | 11.4 | 24.8 | 277.5 | 360.1 | 106.1 |
| ID248 | 131.6 | 68.7 | 73.8 | 66.5 | 41.9 | 37.8 | 38.8 | 50.7 | 36.9 | 138.7 | 163.9 | 197.0 |
| ID249 | 116.4 | 118.8 | 141.0 | 167.4 | 85.0 | 164.0 | 8.7 | 87.8 | 141.9 | 21.6 | 32.4 | 30.6 |
| ID251 | 20.4 | 23.4 | 91.6 | 38.6 | 23.3 | 26.2 | 239.9 | 47.4 | 47.5 | 254.1 | 176.4 | 211.1 |
| ID252 | 125.3 | 88.4 | 142.1 | 150.1 | 99.5 | 154.9 | 32.8 | 112.6 | 140.8 | 37.5 | 40.1 | 38.8 |
| ID253 | 34.1 | 78.1 | 92.3 | 253.8 | 43.1 | 73.7 | 129.0 | 65.9 | 136.6 | 88.6 | 244.5 | 255.7 |
| ID254 | 119.4 | 106.1 | 134.3 | 67.2 | 130.1 | 76.9 | 109.4 | 111.5 | 74.8 | 119.5 | 105.5 | 98.8 |
| ID255 | 51.1 | 81.6 | 52.4 | 38.4 | 52.1 | 38.0 | 248.5 | 64.2 | 39.1 | 235.4 | 245.9 | 159.8 |
| ID256 | 0.9 | 0.0 | 57.8 | 0.0 | 11.6 | 2.1 | 621.7 | 9.5 | 32.5 | 570.8 | 487.9 | 264.1 |
| ID257 | 5.2 | 8.3 | 27.1 | 6.1 | 45.8 | 1.3 | 381.5 | 52.0 | 32.1 | 314.0 | 400.2 | 274.4 |
| ID259 | 54.9 | 42.4 | 51.8 | 29.4 | 115.2 | 27.1 | 201.4 | 108.6 | 39.9 | 190.0 | 286.1 | 206.5 |
| ID26 | 158.2 | 9.1 | 168.3 | 84.9 | 174.2 | 352.9 | 84.9 | 0.0 | 90.0 | 132.4 | 18.6 | 952.0 |
| ID260 | 59.5 | 37.0 | 42.3 | 37.8 | 63.5 | 42.6 | 358.1 | 89.1 | 46.5 | 279.1 | 196.5 | 143.9 |
| ID261 | 135.6 | 109.9 | 117.2 | 128.8 | 141.6 | 122.8 | 9.9 | 102.5 | 122.0 | 26.7 | 46.9 | 73.0 |
| ID262 | 94.7 | 100.9 | 117.9 | 94.9 | 103.6 | 141.3 | 96.3 | 97.7 | 132.9 | 59.4 | 86.5 | 79.8 |
| ID263 | 685.6 | 155.3 | 288.5 | 114.2 | 89.8 | 163.5 | 44.8 | 114.5 | 96.5 | 1.4 | 6.7 | 66.3 |
| ID264 | 102.0 | 77.9 | 128.8 | 50.4 | 59.5 | 91.9 | 309.5 | 38.6 | 129.5 | 43.9 | 106.6 | 175.8 |
| ID265 | 41.0 | 37.6 | 39.2 | 43.2 | 80.4 | 45.5 | 118.4 | 95.4 | 64.3 | 145.5 | 173.2 | 204.8 |
| ID266 | 109.2 | 98.5 | 108.3 | 27.5 | 72.5 | 112.8 | 374.3 | 95.1 | 97.5 | 103.7 | 113.9 | 61.3 |
| ID267 | 175.7 | 127.5 | 162.0 | 165.4 | 127.7 | 129.1 | 6.3 | 111.8 |  |  | 28.9 | 66.9 |
| ID268 | 88.1 | 20.1 | 91.9 | 110.6 | 89.0 | 129.2 | 72.2 | 58.2 | 107.3 | 82.2 | 93.1 | 73.9 |
| ID269 | 65.6 | 59.3 | 62.3 | 44.6 | 75.6 | 46.1 | 278.5 | 92.0 | 38.4 | 194.4 | 166.9 | 141.9 |
| ID2698 | 15.9 | 31.2 | 24.5 | 25.3 | 35.6 | 27.4 | 276.9 | 45.7 | 31.2 | 158.9 | 208.6 | 167.4 |
| ID270 | 104.7 | 82.8 | 96.9 | 152.2 | 126.0 | 115.5 | 19.2 | 135.2 | 173.8 | 37.1 | 33.4 | 71.4 |
| ID271 | 56.5 | 48.3 | 88.0 | 94.5 | 68.5 | 33.8 | 248.8 | 55.3 | 18.5 | 271.4 | 148.1 | 213.2 |
| ID2710 | 181.3 | 173.0 | 157.2 | 136.8 | 106.6 | 77.9 | 206.9 | 85.4 | 58.7 | 154.7 | 116.9 | 112.4 |
| ID2718 | 247.2 | 90.8 | 150.4 | 80.5 | 51.8 | 99.0 | 129.6 | 88.7 | 84.1 | 136.3 | 107.7 | 72.0 |
| ID272 | 43.2 | 25.2 | 40.1 | 38.3 | 67.0 | 44.6 | 256.9 | 69.0 | 44.5 | 238.8 | 238.1 | 189.4 |
| ID273 | 94.3 | 86.1 | 128.0 | 101.8 | 98.8 | 107.8 | 317.6 | 51.1 | 121.5 | 88.5 | 122.1 | 84.3 |
| ID2735 | 365.0 | 50.8 | 149.2 | 131.5 | 27.1 | 302.5 | 96.1 | 107.0 | 97.7 | 133.1 | 149.3 | 68.0 |
| ID274 | 33.7 | 92.0 | 47.1 | 30.7 | 58.1 | 29.5 | 217.0 | 83.4 | 39.6 | 222.4 | 214.1 | 198.1 |
| ID275 | 74.0 | 43.0 | 51.9 | 91.0 | 115.6 | 64.6 | 54.6 | 141.8 | 114.3 | 81.1 | 92.9 | 159.0 |
| ID2751 | 43.1 | 133.9 | 40.1 | 74.1 | 8.4 | 68.6 | 180.6 | 130.9 | 30.8 | 248.1 | 170.6 | 107.9 |
| ID277 | 103.9 | 90.4 | 100.8 | 89.1 | 67.2 | 64.9 | 202.0 | 43.6 | 73.4 | 220.6 | 122.0 | 147.8 |
| ID2777 | 66.5 | 93.8 | 69.5 | 87.1 | 26.0 | 77.8 | 134.9 | 116.2 | 51.8 | 235.0 | 150.6 | 98.6 |
| ID278 | 150.4 | 118.2 | 141.2 | 243.7 | 104.3 | 150.5 | 16.8 | 82.7 |  |  | 12.5 | 19.1 |
| ID279 | 113.7 | 128.7 | 109.5 | 269.7 | 103.3 | 135.7 | 27.2 | 98.9 | 244.4 | 28.8 | 25.7 | 36.7 |
| ID28 | 70.6 | 57.9 | 48.4 | 53.3 | 72.1 | 41.1 | 93.4 | 30.5 | 87.0 | 139.3 | 102.9 | 118.5 |
| ID280 | 53.1 | 286.1 | 50.6 | 43.0 | 64.9 | 45.8 | 244.6 | 151.3 | 38.8 | 260.5 | 227.8 | 175.4 |
| ID281 | 99.0 | 127.0 | 137.8 | 146.6 | 131.1 | 146.9 | 82.6 | 123.7 | 127.6 | 143.6 | 143.8 | 88.6 |
| ID2811 | 87.9 | 77.7 | 103.4 | 97.3 | 56.9 | 85.8 | 127.1 | 83.8 | 70.1 | 160.2 | 108.9 | 80.6 |
| ID282 | 201.2 | 169.6 | 209.9 | 230.8 | 342.0 | 152.7 | 13.5 | 144.1 | 236.8 | 48.9 | 84.8 | 89.3 |
| ID283 | 74.1 | 60.6 | 79.0 | 65.5 | 71.9 | 87.8 | 159.2 | 69.3 | 76.9 | 153.8 | 146.7 | 89.5 |
| ID284 | 162.9 | 150.4 | 159.7 | 94.2 | 80.6 | 98.3 | 87.5 | 80.6 | 74.5 | 187.3 | 96.5 | 78.3 |
| ID285 | 27.7 | 26.4 | 42.9 | 75.6 | 46.8 | 50.8 | 130.6 | 63.4 | 36.3 | 245.1 | 225.6 | 107.6 |
| ID2853 | 109.1 | 166.3 | 126.0 | 86.3 | 95.6 | 146.3 | 131.8 | 80.1 | 84.0 | 85.0 | 50.0 | 45.5 |
| ID286 | 122.8 | 132.1 | 132.6 | 134.6 | 83.4 | 135.0 | 59.5 | 81.1 | 129.1 | 46.1 | 60.2 | 60.7 |
| ID287 | 46.7 | 33.5 | 35.2 | 29.4 | 69.8 | 42.2 | 191.3 | 79.2 | 32.1 | 208.4 | 200.8 | 186.7 |
| ID288 | 139.4 | 84.7 | 103.6 | 28.7 | 106.8 | 48.8 | 138.9 | 89.9 | 65.4 | 91.5 | 152.8 | 182.8 |
| ID289 | 63.0 | 63.3 | 61.6 | 55.9 | 110.2 | 65.5 | 107.1 | 116.3 | 67.1 | 126.1 | 137.1 | 159.0 |
| ID29 | 161.8 | 107.1 | 54.8 | 79.1 | 103.7 | 5.0 | 243.6 | 94.5 | 93.8 | 121.5 | 201.2 | 154.3 |
| ID290 | 141.6 | 186.6 | 294.8 |  | 123.2 | 260.0 |  | 239.2 | 198.5 | 38.4 | 36.7 | 61.5 |
| ID2901 | 428.4 | 154.2 | 305.2 | 37.6 | 9.7 | 3.7 | 17.6 | 1.8 | 83.1 | 23.8 | 46.4 | 1.5 |
| ID291 | 107.0 | 105.8 | 114.7 | 111.6 | 123.5 | 121.6 | 61.2 | 104.5 | 112.5 | 76.3 | 67.2 | 92.8 |
| ID292 | 34.1 | 17.2 | 41.1 | 99.0 | 46.7 | 44.0 | 100.9 | 42.9 | 43.6 | 261.4 | 225.1 | 107.2 |
| ID293 | 117.2 | 137.3 | 124.4 | 132.5 | 109.1 | 136.8 | 66.3 | 106.3 | 133.1 | 77.3 | 80.7 | 78.3 |
| ID2938 | 161.1 | 138.5 | 119.4 | 164.5 | 299.3 | 118.6 | 42.4 | 124.1 | 193.9 | 61.7 | 150.6 | 141.1 |
| ID295 | 581.6 | 354.1 | 65.3 | 177.4 | 72.8 | 136.4 | 67.0 | 218.9 | 272.7 | 86.2 | 276.6 | 233.4 |
| ID2954 | 400.8 | 194.7 | 183.5 | 185.5 | 195.5 | 132.3 | 2.5 | 114.8 | 152.2 | 37.2 | 42.7 | 71.0 |
| ID296 | 78.6 | 82.1 | 84.5 | 93.2 | 103.9 | 88.6 | 147.3 | 86.2 | 98.5 | 132.2 | 137.7 | 123.5 |
| ID2968 | 45.2 | 3.7 | 56.7 | 0.0 | 76.5 | 45.7 | 430.8 | 91.9 | 22.2 | 155.1 | 303.9 | 162.7 |
| ID297 | 55.3 | 24.1 | 43.5 | 14.8 | 56.9 | 13.4 | 209.8 | 41.3 | 1.4 | 315.6 | 254.1 | 283.4 |
| ID298 | 77.1 | 72.9 | 90.5 | 83.4 | 114.3 | 75.2 | 114.1 | 95.9 | 82.9 | 137.7 | 109.0 | 137.5 |
| ID2989 | 408.2 | 212.1 | 172.2 | 85.0 | 51.1 | 122.9 | 6.7 | 67.0 | 125.3 | 12.7 | 15.1 | 32.1 |
| ID299 | 128.6 | 134.9 | 137.7 | 148.8 | 114.9 | 157.7 | 35.3 | 95.6 | 145.7 | 36.6 | 39.9 | 52.9 |
| ID3 | 190.7 | 138.3 | 83.1 | 179.7 | 101.0 | 115.1 | 117.5 | 101.8 | 453.8 | 62.0 | 163.3 | 296.5 |
| ID30 | 165.1 | 79.1 | 95.3 | 63.6 | 113.1 | 17.5 | 116.4 | 91.5 | 72.7 | 269.1 | 296.5 | 103.7 |
| ID300 | 104.1 | 96.8 | 102.3 | 95.1 | 142.9 | 108.2 | 35.8 | 152.4 | 116.2 | 45.6 | 54.5 | 93.1 |
| ID3007 | 18.6 | 4.7 | 0.0 | 0.2 | 0.0 | 0.9 | 384.8 | 15.5 | 68.1 | 1063.3 | 1721.4 | 860.3 |
| ID301 | 117.7 | 118.1 | 120.7 | 136.4 | 124.9 | 135.2 | 37.4 | 117.7 | 129.3 | 42.5 | 49.8 | 60.8 |
| ID302 | 138.2 | 108.7 | 120.4 | 80.9 | 118.7 | 103.5 | 51.2 | 105.5 | 94.7 | 40.1 | 53.8 | 79.9 |
| ID3026 | 100.2 | 86.9 | 117.7 | 741.2 | 106.3 | 110.3 | 0.0 | 101.5 | 107.7 | 51.6 | 52.2 | 85.0 |
| ID303 | 57.8 | 50.1 | 57.1 | 71.1 | 121.4 | 63.2 | 114.3 | 125.7 | 82.0 | 129.1 | 137.2 | 142.1 |
| ID304 | 52.9 | 52.0 | 58.2 | 56.7 | 86.5 | 54.4 | 226.3 | 71.2 | 52.0 | 188.6 | 198.4 | 134.9 |
| ID305 | 62.3 | 65.6 | 45.2 | 58.5 | 74.5 | 55.7 | 128.6 | 85.4 | 49.2 | 161.4 | 177.2 | 176.8 |
| ID306 | 78.4 | 70.2 | 72.9 | 50.1 | 109.8 | 71.5 | 87.3 | 120.2 | 65.8 | 97.5 | 99.7 | 124.0 |
| ID3062 | 103.4 | 126.4 | 93.9 | 97.1 | 82.6 | 77.9 | 38.9 | 66.1 | 94.2 | 38.2 | 58.7 | 71.5 |
| ID307 | 29.3 | 2.4 | 10.3 | 3.2 | 22.3 | 2.0 | 402.8 | 6.8 | 0.3 | 496.1 | 367.5 | 251.0 |
| ID308 | 30.8 | 36.9 | 35.9 | 43.7 | 68.4 | 28.8 | 199.0 | 76.9 | 36.9 | 234.7 | 206.6 | 170.7 |
| ID309 | 120.4 | 152.6 | 177.7 | 137.9 | 106.1 | 151.3 | 35.0 | 118.9 | 152.0 | 33.9 | 38.8 | 57.0 |
| ID31 | 87.6 | 140.0 | 81.2 | 64.4 | 50.9 | 55.2 | 1341.0 | 79.0 | 144.6 | 112.1 | 255.4 | 164.6 |
| ID310 | 124.7 | 131.0 | 142.2 | 151.3 | 119.1 | 141.2 | 42.5 | 120.2 | 132.5 | 35.0 | 44.1 | 51.2 |
| ID311 | 116.0 | 84.6 | 99.3 | 66.1 | 60.7 | 86.4 | 193.3 | 69.3 | 74.2 | 200.5 | 182.8 | 104.3 |
| ID312 | 66.4 | 57.0 | 79.6 | 73.5 | 53.0 | 64.3 | 214.4 | 58.8 | 39.0 | 215.7 | 189.1 | 108.8 |
| ID313 | 46.6 | 64.0 | 44.1 | 50.7 | 87.2 | 51.1 | 136.7 | 124.3 | 59.2 | 165.9 | 169.0 | 208.8 |
| ID314 | 101.2 | 92.2 | 77.4 | 71.3 | 113.0 | 65.8 | 90.0 | 114.9 | 64.8 | 93.4 | 113.3 | 146.6 |
| ID315 | 122.8 | 134.6 | 119.1 | 102.1 | 81.0 | 124.8 | 89.3 | 87.6 | 100.8 | 80.8 | 77.2 | 77.1 |
| ID3151 | 128.7 | 190.0 | 172.9 | 83.4 | 46.5 | 63.1 | 50.5 | 51.6 | 71.2 | 41.2 | 57.6 | 58.0 |
| ID316 | 110.6 | 125.4 | 134.3 | 102.4 | 113.5 | 111.1 | 56.4 | 134.8 | 112.1 | 46.0 | 54.6 | 80.2 |
| ID317 | 130.2 | 138.2 | 143.7 | 164.3 | 107.8 | 162.0 | 15.7 | 109.9 | 166.1 | 20.5 | 20.2 | 36.9 |
| ID319 | 78.4 | 72.1 | 74.9 | 68.5 | 100.3 | 90.8 | 121.0 | 135.2 | 82.8 | 122.0 | 107.8 | 101.9 |
| ID32 | 176.5 | 62.8 |  | 132.9 | 117.4 | 4.0 | 93.7 | 69.4 | 92.9 | 215.6 |  | 121.0 |
| ID320 | 119.2 | 124.4 | 128.8 | 121.9 | 90.7 | 117.4 | 51.7 | 89.8 | 115.4 | 47.4 | 47.3 | 78.4 |
| ID321 | 82.0 | 128.8 | 150.6 | 152.8 | 125.5 | 223.1 | 225.3 | 103.4 | 204.0 | 336.1 | 249.0 | 78.1 |
| ID3218 | 55.1 | 94.0 | 17.4 | 22.2 | 33.8 | 20.9 | 325.1 | 25.5 | 26.0 | 189.0 | 188.9 | 75.7 |
| ID322 | 126.7 | 155.1 | 147.8 | 175.7 | 103.7 | 164.3 | 21.6 | 118.8 | 198.6 | 35.3 | 17.4 | 32.3 |
| ID323 | 112.9 | 89.3 | 89.7 | 56.8 | 118.9 | 73.0 | 74.2 | 114.0 | 69.4 | 88.1 | 96.6 | 119.7 |
| ID324 | 60.0 | 71.5 | 51.3 | 57.7 | 45.1 | 59.8 | 185.3 | 64.0 | 51.9 | 172.9 | 185.7 | 156.7 |
| ID325 | 141.5 | 143.6 | 159.0 | 215.8 | 93.7 | 148.2 | 76.4 | 98.1 | 190.5 | 68.0 | 22.6 | 30.6 |
| ID326 | 9.8 | 9.8 | 9.7 | 20.1 | 24.5 | 7.7 | 296.9 | 34.3 | 15.2 | 309.7 | 272.4 | 151.7 |
| ID327 | 5.2 | 5.0 | 5.7 | 23.4 | 17.3 | 5.0 | 347.7 | 19.3 | 13.7 | 308.0 | 282.4 | 114.8 |
| ID328 | 87.6 | 97.7 | 170.8 | 211.6 | 106.2 | 146.7 | 149.4 | 106.6 | 235.8 | 92.1 | 180.2 | 96.3 |
| ID329 | 56.8 | 54.6 | 50.9 | 53.7 | 84.6 | 60.5 | 161.4 | 101.6 | 53.5 | 166.6 | 149.2 | 141.2 |
| ID33 | 195.0 | 92.3 | 3.6 | 97.0 | 127.3 | 42.5 | 150.1 | 79.5 | 96.3 | 155.1 | 282.5 | 103.9 |
| ID330 | 98.7 | 107.2 | 109.1 | 111.5 | 67.6 | 128.2 | 62.0 | 93.2 | 105.3 | 86.5 | 71.1 | 64.1 |
| ID331 | 138.2 | 133.5 | 164.9 | 110.8 | 99.0 | 127.6 | 84.8 | 102.2 | 120.1 | 71.4 | 91.7 | 73.0 |
| ID332 | 35.6 | 38.2 | 41.2 | 57.5 | 63.4 | 58.9 | 173.5 | 77.3 | 53.9 | 192.8 | 186.2 | 178.0 |
| ID333 | 125.1 | 89.8 | 100.5 | 209.0 | 107.8 | 95.4 | 92.0 | 95.7 | 154.6 | 105.9 | 123.2 | 113.2 |
| ID334 | 40.0 | 72.5 | 86.4 | 88.1 | 78.1 | 122.9 | 127.7 | 130.3 | 129.6 | 119.8 | 97.4 | 126.5 |
| ID335 | 30.0 | 88.4 | 102.1 | 100.9 | 55.3 | 127.9 | 114.3 | 102.3 | 113.4 | 115.6 | 86.5 | 143.6 |
| ID336 | 62.3 | 44.2 | 68.9 | 82.9 | 55.4 | 71.4 | 198.9 | 65.2 | 80.2 | 153.7 | 166.5 | 131.5 |
| ID337 | 23.6 | 12.5 | 47.3 | 86.3 | 40.1 | 76.7 | 126.1 | 31.4 | 85.5 | 130.3 | 151.9 | 136.1 |
| ID338 | 37.1 | 40.4 | 46.5 | 67.3 | 61.8 | 66.0 | 225.9 | 81.3 | 74.9 | 182.4 | 182.1 | 144.4 |
| ID339 | 115.5 | 110.7 | 87.9 | 179.8 | 94.3 | 82.4 | 99.9 | 93.7 | 130.9 | 86.6 | 116.4 | 111.5 |
| ID3397 | 113.6 | 165.9 | 151.5 | 87.8 | 60.0 | 75.4 | 53.1 | 71.6 | 53.3 | 45.6 | 74.8 | 65.9 |
| ID34 | 75.1 | 89.6 | 62.7 | 69.6 | 72.5 | 48.9 | 93.2 | 77.2 | 88.9 | 104.5 | 127.2 | 122.9 |
| ID340 | 58.8 | 40.4 | 45.4 | 68.0 | 75.9 | 62.7 | 176.2 | 87.9 | 51.6 | 136.8 | 151.2 | 134.3 |
| ID341 | 124.7 | 132.7 | 151.8 | 141.7 | 113.0 | 157.1 | 33.9 | 115.0 | 137.1 | 32.1 | 39.6 | 47.3 |
| ID342 | 161.0 | 130.5 | 88.8 | 172.0 | 97.3 | 80.9 | 99.5 | 91.7 | 162.9 | 95.8 | 155.8 | 134.1 |
| ID344 | 79.7 | 70.6 | 84.5 | 65.5 | 58.8 | 45.2 | 190.8 | 53.4 | 41.1 | 199.0 | 188.9 | 159.3 |
| ID345 | 80.3 | 77.1 | 96.9 | 90.4 | 92.8 | 115.0 | 121.5 | 133.0 | 130.4 | 120.3 | 123.6 | 149.4 |
| ID346 | 41.8 | 43.6 | 48.8 | 126.3 | 81.3 | 130.6 | 114.3 | 95.6 | 137.7 | 99.1 | 116.6 | 103.9 |
| ID347 | 129.0 | 126.2 | 124.4 | 160.9 | 122.7 | 148.1 | 88.3 | 102.9 | 153.2 | 78.7 | 73.4 | 69.4 |
| ID348 | 200.2 | 144.1 | 105.2 | 27.7 | 135.4 | 103.1 | 65.1 | 108.3 | 10200.3 | 8447.3 | 137.6 | 102.5 |
| ID349 | 190.0 | 166.2 | 148.2 | 237.2 | 90.6 | 92.8 | 76.2 | 115.5 | 205.9 | 90.6 | 229.8 | 165.1 |
| ID35 | 177.6 | 105.8 | 64.5 | 131.1 | 110.0 | 24.8 | 291.6 | 80.3 | 75.4 | 52.8 | 146.0 | 124.0 |
| ID350 | 82.1 | 58.1 | 57.4 | 83.1 | 90.1 | 82.9 | 76.1 | 123.2 | 81.5 | 82.4 | 131.3 | 104.7 |
| ID351 | 124.3 | 93.7 | 154.8 | 93.6 | 95.8 | 80.7 | 123.7 | 114.2 | 113.2 | 94.5 | 79.5 | 65.1 |
| ID352 | 50.1 | 50.2 | 48.8 | 66.9 | 75.8 | 72.8 | 131.4 | 92.2 | 60.2 | 145.8 | 165.9 | 136.2 |
| ID3521 | 33.4 | 47.2 | 86.2 | 74.3 | 15.2 | 61.6 | 63.1 | 50.0 | 26.3 | 62.1 | 126.6 | 59.5 |
| ID353 | 28.9 | 37.2 | 34.3 | 78.0 | 87.9 | 80.9 | 172.3 | 123.1 | 83.0 | 151.2 | 136.0 | 143.4 |
| ID354 | 756.9 | 466.2 | 264.0 | 319.3 | 132.6 | 162.8 | 81.4 | 99.0 | 102.1 | 97.1 | 1853.9 | 821.1 |
| ID355 | 71.1 | 87.0 | 72.2 | 84.7 | 66.6 | 67.0 | 209.9 | 65.4 | 80.1 | 188.2 | 193.4 | 139.2 |
| ID356 | 212.7 | 90.5 | 84.0 | 88.7 | 102.9 | 50.2 | 94.8 | 89.6 | 99.2 | 110.4 | 117.8 | 81.2 |
| ID357 | 82.0 | 120.5 | 120.9 | 132.2 | 68.4 | 127.4 | 10.9 | 96.6 | 123.2 | 19.1 | 12.8 | 28.0 |
| ID358 | 229.2 | 93.4 | 81.3 | 83.2 | 151.4 | 90.9 | 121.8 | 91.6 | 93.6 | 99.2 | 116.7 | 106.4 |
| ID359 | 54.0 | 64.0 | 65.1 | 118.6 | 77.7 | 134.4 | 53.0 | 110.0 | 123.3 | 54.1 | 78.4 | 73.7 |
| ID36 | 83.6 | 96.9 | 72.0 | 78.5 | 67.4 | 55.9 | 80.8 | 72.9 | 75.9 | 116.4 | 93.1 | 100.1 |
| ID360 | 48.4 | 43.8 | 44.6 | 69.8 | 103.5 | 77.9 | 95.3 | 125.6 | 85.2 | 111.7 | 121.6 | 167.1 |
| ID361 | 223.3 | 107.8 | 79.6 | 89.1 | 116.2 | 85.2 | 109.1 | 96.6 | 98.3 | 97.6 | 97.5 | 96.4 |
| ID362 | 59.1 | 61.7 | 58.0 | 61.7 | 74.7 | 70.5 | 138.5 | 95.0 | 67.3 | 138.5 | 134.1 | 145.3 |
| ID363 | 90.1 | 81.3 | 77.9 | 107.8 | 83.9 | 103.4 | 84.0 | 101.5 | 114.6 | 81.8 | 114.1 | 121.5 |
| ID364 | 80.9 | 73.2 | 73.2 | 95.2 | 80.5 | 94.2 | 121.4 | 80.2 | 93.5 | 110.8 | 120.8 | 103.3 |
| ID3643 | 125.4 | 115.1 | 133.5 | 177.2 | 136.1 | 145.6 | 103.0 | 132.6 | 126.9 | 90.2 | 74.1 | 90.5 |
| ID366 | 81.2 | 67.5 | 73.5 | 82.7 | 90.5 | 79.8 | 108.8 | 85.7 | 80.8 | 113.8 | 116.2 | 127.8 |
| ID367 | 10.3 | 67.0 | 28.8 | 43.2 | 9.9 | 48.4 | 227.9 | 45.5 | 116.5 | 105.7 | 56.1 | 45.0 |
| ID368 | 164.7 | 116.0 | 103.1 | 152.1 | 81.0 | 94.8 | 66.4 | 78.8 | 175.8 | 77.9 | 229.0 | 157.3 |
| ID369 | 159.8 | 81.5 | 67.4 | 53.8 | 89.6 | 73.8 | 115.1 | 82.6 | 68.9 | 104.9 | 78.6 | 92.1 |
| ID37 | 67.0 | 98.9 | 76.8 | 100.5 | 65.3 | 35.9 | 83.8 | 73.5 | 93.6 | 108.1 | 116.6 | 108.3 |
| ID370 | 95.7 | 85.8 | 94.0 | 92.3 | 94.4 | 92.5 | 84.9 | 122.9 | 96.3 | 101.2 | 96.1 | 126.9 |
| ID371 | 100.1 | 105.1 | 105.4 | 97.3 | 92.3 | 109.3 | 73.6 | 101.8 | 114.8 | 60.3 | 70.5 | 88.2 |
| ID372 | 90.4 | 82.4 | 89.5 | 91.3 | 112.9 | 105.4 | 46.3 | 146.6 | 114.3 | 54.7 | 56.1 | 108.0 |
| ID373 | 115.6 | 105.8 | 128.3 | 78.0 | 80.1 | 81.2 | 96.2 | 111.1 | 84.9 | 99.2 | 102.3 | 116.7 |
| ID374 | 22.5 | 39.1 | 49.6 | 161.4 | 65.7 | 90.7 | 327.7 | 95.6 | 117.5 | 193.3 | 209.9 | 180.0 |
| ID375 | 139.0 | 142.4 | 136.7 | 129.6 | 103.1 | 120.1 | 53.5 | 97.2 | 131.0 | 51.4 | 63.3 | 71.7 |
| ID376 | 92.2 | 89.7 | 89.8 | 97.8 | 95.6 | 90.8 | 100.3 | 90.6 | 72.0 | 137.3 | 108.6 | 107.1 |
| ID3766 | 128.4 | 129.1 | 142.8 | 131.6 | 135.3 | 153.7 | 84.0 | 143.0 | 127.8 | 86.0 | 66.8 | 86.6 |
| ID377 | 120.1 | 156.7 | 138.1 | 103.1 | 74.0 | 100.6 | 35.5 | 81.4 | 100.0 | 51.4 | 35.5 | 101.3 |
| ID378 | 19.6 | 35.2 | 44.6 | 89.0 | 75.5 | 65.5 | 337.0 | 99.9 | 99.2 | 220.7 | 217.1 | 148.7 |
| ID379 | 116.5 | 120.3 | 125.2 | 159.0 | 93.3 | 171.1 | 16.4 | 108.4 | 160.6 | 24.1 | 18.7 | 44.3 |
| ID38 | 148.8 | 122.9 | 100.1 | 284.1 | 88.1 | 113.6 | 3.5 | 83.6 |  |  | 65.6 | 87.7 |
| ID380 | 105.8 | 102.6 | 99.8 | 100.9 | 107.0 | 91.3 | 77.9 | 99.8 | 94.2 | 84.6 | 86.2 | 106.0 |
| ID381 | 126.1 | 122.8 | 122.0 | 131.9 | 97.9 | 123.3 | 34.8 | 93.1 | 118.3 | 38.7 | 43.4 | 59.7 |
| ID382 | 18.3 | 26.2 | 17.7 | 26.6 | 55.7 | 21.4 | 221.1 | 63.5 | 27.9 | 214.2 | 230.9 | 182.1 |
| ID383 | 1089.7 | 1049.6 | 535.8 | 896.0 | 249.3 | 806.5 | 152.7 | 111.9 | 44.7 | 94.1 | 2374.3 | 5710.6 |
| ID384 | 40.2 | 38.3 | 34.4 | 45.5 | 61.2 | 31.6 | 228.3 | 63.1 | 35.0 | 222.0 | 217.3 | 154.2 |
| ID386 | 110.3 | 121.3 | 104.0 | 99.3 | 89.4 | 100.6 | 77.8 | 91.2 | 116.5 | 81.3 | 86.7 | 102.4 |
| ID387 | 149.2 | 173.5 | 145.2 | 151.9 | 101.5 | 143.8 | 16.6 | 106.9 | 134.9 | 29.6 | 28.8 | 34.7 |
| ID388 | 125.5 | 149.2 | 114.7 | 125.8 | 99.2 | 131.5 | 72.3 | 87.7 | 131.1 | 66.0 | 144.2 | 64.3 |
| ID836 | 78.7 | 65.9 | 64.4 | 71.3 | 113.4 | 79.6 | 113.0 | 130.3 | 69.5 | 163.3 | 137.1 | 154.9 |
| ID389 | 85.5 | 89.5 | 73.3 | 82.0 | 74.6 | 74.1 | 148.1 | 72.7 | 70.1 | 157.0 | 184.0 | 114.3 |
| ID390 | 36.7 | 27.8 | 29.7 | 23.3 | 57.8 | 28.4 | 254.3 | 56.9 | 32.3 | 238.9 | 243.7 | 181.7 |
| ID391 | 48.4 | 54.4 | 46.4 | 71.2 | 85.6 | 49.5 | 145.8 | 96.8 | 78.7 | 156.0 | 166.9 | 168.7 |
| ID392 | 134.0 | 127.5 | 122.8 | 135.0 | 94.1 | 107.3 | 41.5 | 93.2 | 150.6 | 53.8 | 49.0 | 81.4 |
| ID393 | 39.5 | 34.6 | 34.0 | 45.1 | 44.8 | 31.1 | 268.5 | 48.6 | 48.4 | 191.0 | 219.2 | 130.9 |
| ID394 | 51.9 | 46.5 | 43.7 | 54.2 | 47.9 | 43.2 | 250.9 | 53.7 | 57.3 | 157.2 | 192.5 | 107.2 |
| ID395 | 71.0 | 68.6 | 65.9 | 73.9 | 83.1 | 64.6 | 151.4 | 84.1 | 35.0 | 219.1 | 150.1 | 129.8 |
| ID396 | 18.4 | 52.4 | 38.1 | 55.5 | 120.0 | 57.6 | 235.0 | 117.3 | 148.6 | 227.5 | 246.2 | 180.5 |
| ID397 | 82.8 | 58.2 | 62.6 | 70.0 | 62.0 | 52.7 | 209.5 | 62.2 | 58.5 | 164.5 | 168.4 | 107.6 |
| ID398 | 90.5 | 95.1 | 74.9 | 81.9 | 82.2 | 65.2 | 161.7 | 68.7 | 74.7 | 181.4 | 200.3 | 123.9 |
| ID399 | 68.8 | 105.9 | 122.5 | 228.2 | 48.2 | 161.4 | 57.6 | 90.8 | 130.6 | 91.3 | 26.2 | 42.1 |
| ID4 | 171.7 | 152.6 | 100.0 | 180.0 | 100.8 | 117.0 | 116.7 | 106.9 | 246.9 | 48.5 | 120.4 | 139.5 |
| ID40 | 133.6 | 137.0 | 159.1 | 141.2 | 87.0 | 313.2 | 25.5 | 112.9 | 138.8 | 33.5 | 53.1 | 57.1 |
| ID400 | 105.4 | 93.4 | 87.1 | 67.4 | 125.5 | 66.1 | 62.9 | 148.7 | 31.8 | 182.0 | 91.8 | 130.6 |
| ID401 | 134.9 | 120.2 | 87.9 | 74.4 | 54.7 | 79.2 | 123.3 | 59.9 | 69.3 | 110.3 | 119.6 | 108.6 |
| ID402 | 119.1 | 142.7 | 110.1 | 101.9 | 97.5 | 93.0 | 99.8 | 105.2 | 99.2 | 104.3 | 98.5 | 110.8 |
| ID403 | 63.3 | 64.0 | 62.0 | 69.0 | 100.3 | 72.7 | 89.6 | 109.9 | 89.0 | 91.1 | 110.7 | 128.8 |
| ID4035 | 101.6 | 98.6 | 103.7 | 70.5 | 100.7 | 87.5 | 105.7 | 121.8 | 78.9 | 107.1 | 93.6 | 113.4 |
| ID404 | 54.3 | 50.2 | 42.8 | 35.7 | 78.5 | 35.0 | 159.5 | 90.1 | 30.5 | 202.7 | 189.6 | 191.4 |
| ID405 | 124.5 | 126.8 | 168.1 | 103.3 | 96.8 | 115.6 | 81.5 | 95.7 | 121.9 | 67.6 | 107.0 | 117.4 |
| ID406 | 133.8 | 166.1 | 170.2 | 93.0 | 101.0 | 110.0 | 64.6 | 85.6 | 146.0 | 97.6 | 79.3 | 114.2 |
| ID407 | 154.9 | 162.9 | 150.4 | 86.3 | 106.8 | 77.3 | 95.9 | 77.7 | 122.9 | 100.0 | 101.0 | 99.7 |
| ID408 | 141.1 | 88.1 | 95.6 | 99.7 | 93.7 | 119.5 | 87.9 | 112.5 | 93.9 | 108.6 | 86.6 | 112.3 |
| ID409 | 154.5 | 149.7 | 117.5 | 89.4 | 94.9 | 53.1 | 100.3 | 65.4 | 121.1 | 111.5 | 141.3 | 111.2 |
| ID410 | 122.1 | 137.9 | 103.1 | 83.9 | 76.3 | 95.8 | 76.5 | 66.5 | 110.1 | 81.1 | 60.4 | 99.5 |
| ID411 | 31.1 | 24.0 | 26.2 | 46.0 | 63.0 | 62.6 | 132.5 | 82.5 | 69.0 | 125.1 | 136.3 | 135.4 |
| ID412 | 167.2 | 123.7 | 73.4 | 95.7 | 92.5 | 61.9 | 70.7 | 63.7 | 108.7 | 104.2 | 153.9 | 146.1 |
| ID413 | 170.4 | 122.1 | 109.1 | 173.7 | 101.2 | 103.1 | 157.4 | 100.6 | 260.5 | 88.8 | 165.2 | 144.2 |
| ID414 | 59.6 | 67.3 | 61.2 | 33.0 | 37.5 | 40.7 | 240.9 | 47.7 | 6.6 | 252.5 | 201.4 | 152.7 |
| ID415 | 145.8 | 101.4 | 89.8 | 107.1 | 93.0 | 91.2 | 69.3 | 97.4 | 115.4 | 73.8 | 98.5 | 106.8 |
| ID417 | 86.0 | 84.3 | 84.3 | 89.0 | 129.3 | 87.4 | 74.3 | 129.2 | 116.2 | 84.4 | 92.8 | 109.3 |
| ID418 | 37.1 | 40.7 | 44.9 | 78.5 | 64.9 | 62.6 | 131.3 | 82.1 | 69.1 | 170.7 | 151.0 | 143.6 |
| ID419 | 145.1 | 110.5 | 85.5 | 127.0 | 87.3 | 80.6 | 76.4 | 92.3 | 118.1 | 74.5 | 113.8 | 114.1 |
| ID42 | 146.2 | 147.9 | 127.1 | 125.0 | 36.2 | 31.2 | 25.7 | 95.8 | 176.6 | 19.1 | 38.4 | 192.1 |
| ID420 | 133.2 | 97.0 | 145.7 | 83.5 | 90.3 | 87.9 | 73.7 | 85.6 | 118.8 | 85.7 | 92.5 | 101.8 |
| ID421 | 94.9 | 75.0 | 78.6 | 68.1 | 105.2 | 71.9 | 193.9 | 115.6 | 70.9 | 224.0 | 199.6 | 132.8 |
| ID422 | 22.0 | 19.3 | 21.3 | 35.8 | 44.1 | 21.0 | 265.9 | 50.0 | 34.0 | 246.4 | 225.4 | 163.6 |
| ID423 | 104.8 | 118.4 | 119.4 | 148.0 | 99.4 | 139.9 | 18.1 | 97.2 | 147.2 | 31.9 | 35.7 | 42.9 |
| ID424 | 55.2 | 47.6 | 56.9 | 54.1 | 94.9 | 60.8 | 92.3 | 110.7 | 68.3 | 87.8 | 108.6 | 136.9 |
| ID425 | 103.6 | 104.2 | 98.7 | 131.8 | 93.6 | 125.6 | 31.4 | 99.7 | 152.6 | 48.0 | 41.4 | 60.8 |
| ID427 | 94.3 | 166.0 | 121.0 | 116.1 | 108.2 | 109.4 | 154.1 | 129.2 | 126.9 | 150.1 | 149.1 | 119.8 |
| ID428 | 772.0 | 374.3 | 324.1 | 517.7 | 181.4 | 218.2 | 198.8 | 108.9 | 100.1 | 93.4 | 1508.0 | 965.5 |
| ID429 | 172.9 | 122.3 | 85.3 | 124.8 | 83.9 | 77.5 | 93.6 | 88.8 | 115.7 | 70.1 | 140.8 | 134.7 |
| ID43 | 64.6 | 113.8 | 61.3 | 82.9 | 75.4 | 66.2 | 94.0 | 82.3 | 100.6 | 87.4 | 105.8 | 100.6 |
| ID430 | 24.4 | 28.1 | 24.1 | 15.3 | 10.4 | 13.3 | 372.5 | 26.9 | 47.0 | 197.9 | 259.1 | 149.3 |
| ID431 | 183.1 | 123.7 | 82.3 | 117.6 | 77.5 | 75.2 | 69.6 | 90.9 | 101.3 | 69.5 | 111.8 | 136.2 |
| ID4312 | 66.3 | 55.5 | 60.7 | 51.2 | 74.4 | 56.5 | 214.0 | 82.0 | 59.0 | 188.6 | 159.1 | 142.7 |
| ID432 | 182.3 | 142.0 | 148.6 | 178.5 | 80.5 | 92.9 | 80.4 | 97.9 | 195.3 | 78.6 | 263.9 | 161.6 |
| ID433 | 52.7 | 55.8 | 50.9 | 33.5 | 37.8 | 41.1 | 321.5 | 30.8 | 61.9 | 146.6 | 197.3 | 111.5 |
| ID434 | 97.8 | 59.2 | 77.2 | 50.5 | 110.4 | 50.2 | 90.3 | 92.0 | 59.7 | 79.2 | 118.5 | 78.8 |
| ID435 | 65.5 | 176.7 | 121.4 | 121.1 | 99.6 | 99.1 | 87.4 | 148.0 | 224.3 | 92.3 | 90.8 | 120.1 |
| ID436 | 57.5 | 88.4 | 102.9 | 160.0 | 69.4 | 100.7 | 71.9 | 98.2 | 196.7 | 44.3 | 59.9 | 91.9 |
| ID437 | 184.4 | 184.7 | 206.0 | 131.9 | 183.4 | 140.8 | 73.0 | 107.7 | 137.1 | 18.8 | 49.9 | 71.5 |
| ID438 | 129.0 | 55.0 | 56.3 | 57.2 | 79.2 | 40.9 | 163.4 | 50.5 | 50.4 | 141.7 | 148.1 | 99.4 |
| ID439 | 123.4 | 131.4 | 136.8 | 149.5 | 116.5 | 142.2 | 16.6 | 96.2 | 145.5 | 16.5 | 28.6 | 40.2 |
| ID44 | 75.0 | 139.5 | 147.0 | 141.8 | 103.8 | 119.4 | 54.6 | 103.0 | 129.2 | 76.7 | 55.6 | 92.9 |
| ID440 | 102.7 | 94.9 | 108.3 | 102.2 | 66.9 | 113.8 | 67.5 | 77.3 | 98.2 | 62.9 | 50.5 | 60.3 |
| ID441 | 202.4 | 71.9 | 113.5 | 128.9 | 112.1 | 154.1 | 10.3 | 65.1 | 136.7 | 11.4 | 11.3 | 9.2 |
| ID442 | 219.8 | 169.3 | 160.4 | 325.1 | 97.0 | 156.8 | 162.3 | 87.2 | 187.9 | 55.5 | 142.1 | 93.2 |
| ID443 | 166.9 | 119.5 | 236.9 | 270.7 | 96.4 | 95.3 | 116.8 | 82.6 | 107.6 | 121.6 | 208.7 | 112.7 |
| ID444 | 53.4 | 34.3 | 36.8 | 49.6 | 73.0 | 35.6 | 155.0 | 77.5 | 57.6 | 167.9 | 171.9 | 177.9 |
| ID445 | 77.8 | 2.5 | 13.4 | 12.1 | 75.0 | 29.4 | 460.5 | 34.0 | 35.8 | 451.8 | 328.2 | 147.1 |
| ID446 | 62.4 | 68.3 | 75.9 | 71.3 | 80.1 | 94.1 | 64.7 | 95.9 | 82.6 | 99.9 | 87.3 | 92.6 |
| ID447 | 62.8 | 25.8 | 34.8 | 19.4 | 72.8 | 22.7 | 182.6 | 70.9 | 16.3 | 208.7 | 187.5 | 234.3 |
| ID448 | 93.4 | 107.8 | 109.8 | 94.1 | 78.2 | 117.4 | 34.6 | 92.1 | 109.6 | 18.7 | 27.8 | 56.1 |
| ID45 | 58.3 | 93.4 | 75.4 | 84.0 | 84.1 | 142.8 | 96.0 | 89.9 | 91.4 | 89.7 | 111.5 | 102.5 |
| ID450 | 224.4 | 235.5 | 222.1 | 121.8 | 67.1 | 79.4 | 69.3 | 59.0 | 94.8 | 43.6 | 35.5 | 89.1 |
| ID451 | 118.8 | 100.0 | 99.1 | 89.1 | 64.0 | 98.6 | 87.1 | 91.7 | 86.3 | 55.3 | 49.2 | 78.7 |
| ID452 | 86.5 | 70.4 | 75.1 | 55.9 | 80.7 | 59.9 | 76.3 | 100.5 | 57.8 | 84.7 | 81.5 | 122.6 |
| ID453 | 78.3 | 62.7 | 72.6 | 112.8 | 65.3 | 78.0 | 137.3 | 52.4 | 97.5 | 111.5 | 139.8 | 117.9 |
| ID454 | 104.5 | 132.6 | 126.2 | 138.6 | 110.6 | 168.3 | 33.1 | 103.4 | 172.8 | 39.2 | 31.9 | 65.1 |
| ID455 | 66.4 | 29.4 | 38.2 | 22.6 | 44.3 | 24.1 | 305.3 | 50.3 | 33.6 | 209.5 | 207.7 | 185.4 |
| ID456 | 78.6 | 50.9 | 47.8 | 43.5 | 22.0 | 40.6 | 224.0 | 73.5 | 39.9 | 189.1 | 183.3 | 144.2 |
| ID457 | 139.4 | 102.8 | 167.5 | 81.1 | 120.9 | 104.9 | 52.5 | 110.8 | 86.5 | 26.6 | 50.2 | 54.2 |
| ID458 | 134.0 | 122.7 | 91.9 | 172.8 | 62.1 | 76.5 | 86.5 | 84.3 | 131.1 | 77.8 | 111.0 | 114.2 |
| ID459 | 193.0 | 120.6 | 188.8 | 103.9 | 74.9 | 75.8 | 42.1 | 49.9 | 80.3 | 51.0 | 68.4 | 72.6 |
| ID46 | 67.3 | 54.4 | 65.5 | 16.9 | 89.0 | 33.4 | 285.1 | 38.5 | 27.8 | 260.2 | 290.7 | 186.1 |
| ID460 | 107.9 | 126.0 | 126.5 | 115.5 | 79.9 | 166.0 | 11.6 | 113.6 | 141.7 | 23.1 | 5.6 | 39.7 |
| ID461 | 98.1 | 125.1 | 154.7 | 146.5 | 199.6 | 124.2 | 29.7 | 168.8 | 148.5 | 30.3 | 70.4 | 69.1 |
| ID462 | 53.0 | 32.9 | 28.2 | 4.6 | 1.1 | 8.8 | 220.8 | 37.4 | 5.1 | 191.3 | 281.2 | 221.5 |
| ID463 | 241.8 | 155.5 | 79.7 | 149.1 | 80.2 | 74.9 | 73.3 | 86.5 | 115.9 | 66.7 | 131.7 | 136.6 |
| ID464 | 108.0 | 51.6 | 86.8 | 35.1 | 66.8 | 56.8 | 110.0 | 68.6 | 56.7 | 89.5 | 128.6 | 144.7 |
| ID465 | 184.0 | 150.3 | 94.1 | 216.6 | 85.5 | 106.2 | 54.9 | 80.6 | 259.8 | 76.7 | 497.4 | 186.2 |
| ID466 | 1672.2 | 605.0 | 569.9 | 822.5 | 278.3 | 497.1 | 97.9 | 84.2 | 86.7 | 95.6 | 2937.4 | 2779.9 |
| ID468 | 118.2 |  | 62.4 | 23.1 | 78.8 | 24.3 | 295.4 |  | 9.9 | 278.8 | 204.7 | 221.8 |
| ID469 | 116.0 | 120.2 | 116.7 | 125.1 | 121.7 | 127.8 | 41.9 | 117.9 | 133.1 | 56.0 | 57.3 | 95.8 |
| ID47 | 67.4 | 118.9 | 70.9 | 77.8 | 70.2 | 69.8 | 105.0 | 80.6 | 95.2 | 94.5 | 102.7 | 95.6 |
| ID470 | 113.6 | 90.0 | 101.2 | 113.0 | 110.6 | 91.9 | 53.3 | 114.7 | 106.5 | 62.4 | 61.1 | 102.3 |
| ID471 | 52.1 | 54.5 | 57.5 | 57.2 | 99.3 | 78.6 | 144.7 | 103.6 | 70.2 | 158.9 | 167.8 | 164.7 |
| ID472 | 263.4 | 91.2 | 205.3 | 130.5 | 377.1 | 82.5 | 46.2 | 108.2 | 185.5 | 77.8 | 12.7 | 40.1 |
| ID473 | 1465.9 | 1209.3 | 148.2 | 117.4 | 1854.5 | 783.9 | 67.8 | 1159.9 | 97.1 | 109.8 | 3.6 | 168.2 |
| ID474 | 120.8 | 101.9 | 110.3 | 139.9 | 87.8 | 114.3 | 61.0 | 112.7 | 137.9 | 36.0 | 45.2 | 84.0 |
| ID475 | 358.7 | 345.9 | 736.6 | 163.4 | 78.3 | 215.8 | 48.8 | 126.0 | 106.8 | 39.0 | 12.1 | 106.8 |
| ID476 | 138.8 | 132.7 | 146.7 | 150.2 | 87.3 | 148.5 | 10.9 | 95.2 | 531.8 | 52.4 | 19.7 | 40.7 |
| ID477 | 71.2 | 28.6 | 97.8 | 47.3 | 80.6 | 120.2 | 148.8 | 68.8 | 83.5 | 118.7 | 63.8 | 75.2 |
| ID478 | 116.1 | 134.1 | 162.7 | 88.0 | 110.6 | 208.5 | 57.9 | 156.0 | 119.5 | 54.0 | 58.2 | 49.6 |
| ID479 | 274.6 | 238.5 | 296.0 | 183.8 | 75.1 | 146.8 | 0.0 | 114.9 | 126.1 | 3.0 | 0.0 | 27.9 |
| ID481 | 123.9 | 131.0 | 141.7 | 175.9 | 118.7 | 128.0 | 43.3 | 105.2 | 110.6 | 34.3 | 29.5 | 94.1 |
| ID482 | 147.3 | 198.5 | 279.7 | 151.2 | 101.5 | 105.3 | 27.3 | 114.7 | 153.5 | 15.4 | 2.9 | 14.9 |
| ID484 | 116.9 | 125.1 | 125.1 | 96.4 | 116.0 | 116.2 | 116.2 | 99.4 | 108.2 | 101.7 | 116.6 | 127.8 |
| ID485 | 129.7 | 76.0 | 76.0 | 82.7 | 82.0 | 69.1 | 65.2 | 92.8 | 89.4 | 44.8 | 44.6 | 99.8 |
| ID486 | 1266.8 | 691.5 | 617.3 | 920.3 | 246.9 | 467.1 | 113.5 | 71.9 | 91.2 | 94.9 | 2508.5 | 2110.4 |
| ID488 | 116.2 | 120.8 | 113.7 | 138.4 | 95.7 | 131.2 | 30.0 | 100.0 | 134.5 | 38.1 | 43.4 | 57.9 |
| ID489 | 365.2 | 322.9 | 339.6 | 146.7 | 52.1 | 229.3 | 41.4 | 110.5 | 187.3 | 65.6 | 22.9 | 13.8 |
| ID49 | 58.1 | 108.8 | 116.6 | 27.0 | 63.3 | 17.2 | 164.7 | 51.5 | 0.9 | 483.1 | 96.7 | 235.4 |
| ID490 | 131.2 | 97.6 | 128.5 | 124.5 | 141.6 | 145.4 | 58.6 | 138.9 | 136.1 | 69.6 | 57.8 | 76.3 |
| ID491 | 80.1 | 86.8 | 95.0 | 120.2 | 130.7 | 120.4 | 43.6 | 133.0 | 140.7 | 53.7 | 58.9 | 96.2 |
| ID492 | 118.2 | 109.8 | 108.5 | 127.0 | 76.3 | 107.6 | 23.1 | 88.6 | 146.8 | 18.6 | 33.5 | 62.1 |
| ID493 | 152.5 | 176.0 | 212.5 | 121.7 | 92.4 | 127.7 | 32.3 | 87.5 | 93.0 | 21.5 | 23.6 | 53.2 |
| ID494 | 64.6 | 55.1 | 54.6 | 57.1 | 102.6 | 56.9 | 102.8 | 112.7 | 65.4 | 136.8 | 128.5 | 153.2 |
| ID496 | 142.4 | 126.5 | 137.2 | 85.4 | 93.2 | 97.9 | 91.8 | 89.9 | 83.0 | 96.4 | 89.3 | 90.8 |
| ID497 | 116.3 | 94.4 | 105.0 | 102.3 | 101.8 | 107.9 | 88.1 | 102.3 | 102.9 | 95.9 | 101.2 | 89.8 |
| ID498 | 100.7 | 98.6 | 97.9 | 135.9 | 117.6 | 111.5 | 120.8 | 137.9 | 139.3 | 112.7 | 176.1 | 146.9 |
| ID499 | 47.9 | 48.9 | 50.9 | 40.2 | 61.7 | 33.1 | 181.6 | 64.7 | 41.4 | 210.5 | 188.3 | 162.2 |
| ID5 | 124.0 | 144.2 | 111.7 | 118.1 | 92.9 | 142.3 | 65.8 | 109.3 |  |  | 125.5 | 173.6 |
| ID50 | 82.7 | 114.1 | 84.7 | 84.5 | 100.9 | 77.3 | 98.2 | 81.6 | 92.5 | 110.5 | 105.1 | 102.9 |
| ID500 | 128.5 | 114.8 | 121.6 | 107.3 | 90.3 | 102.1 | 46.5 | 98.3 | 114.5 | 35.6 | 34.1 | 63.9 |
| ID501 | 161.4 | 161.6 | 170.8 | 101.3 | 58.6 | 86.0 | 62.1 | 63.9 | 73.6 | 40.6 | 28.6 | 29.5 |
| ID502 | 123.0 | 82.4 | 113.5 | 79.3 | 93.9 | 87.7 | 111.7 | 102.4 | 69.6 | 125.6 | 108.2 | 123.9 |
| ID503 | 998.2 | 530.7 | 478.5 | 722.0 | 156.2 | 344.8 | 154.6 | 155.8 | 138.3 | 111.8 | 1628.7 | 1005.8 |
| ID504 | 48.3 | 41.6 | 39.9 | 31.0 | 39.7 | 32.0 | 236.4 | 46.5 | 36.9 | 244.8 | 223.4 | 159.8 |
| ID506 | 55.0 | 48.6 | 109.0 | 80.9 | 92.0 | 17.3 | 222.7 | 38.8 | 138.3 | 339.0 | 591.0 | 113.7 |
| ID507 | 155.2 | 103.0 | 146.0 | 181.4 | 70.6 | 96.9 | 115.1 | 104.3 | 220.1 | 61.0 | 643.2 | 152.9 |
| ID508 | 1.7 | 79.1 | 59.7 | 8.4 | 20.7 | 24.1 | 292.0 | 81.4 | 0.0 | 2812.6 | 317.1 | 115.5 |
| ID509 | 73.8 | 52.5 | 65.3 | 48.8 | 55.1 | 68.5 | 190.2 | 45.6 | 68.1 | 153.4 | 139.8 | 89.9 |
| ID51 | 144.0 | 173.7 | 103.3 | 164.5 | 89.2 | 149.9 | 95.7 | 95.4 | 104.9 | 54.1 | 72.2 | 78.7 |
| ID511 | 78.1 | 47.4 | 49.0 | 34.9 | 66.3 | 51.8 | 203.0 | 54.1 | 63.5 | 154.8 | 165.4 | 108.0 |
| ID512 | 103.5 | 105.2 | 94.7 | 124.5 | 114.7 | 97.9 | 82.5 | 158.0 | 170.4 | 118.8 | 227.1 | 153.2 |
| ID513 | 109.8 | 45.8 | 109.1 | 106.0 | 103.7 | 128.5 | 126.3 | 108.7 | 84.5 | 170.6 | 232.7 | 136.1 |
| ID514 | 20.6 | 37.9 | 16.9 | 26.9 | 51.4 | 28.1 | 406.7 | 61.0 | 20.9 | 444.4 | 337.0 | 178.9 |
| ID515 | 191.8 | 136.7 | 143.3 | 67.5 | 76.9 | 58.6 | 90.4 | 50.9 | 38.7 | 46.8 | 44.2 | 49.3 |
| ID516 | 70.6 | 64.6 | 48.9 | 57.7 | 45.0 | 62.7 | 152.8 | 47.9 | 65.2 | 142.1 | 135.9 | 96.4 |
| ID517 | 20.5 | 10.6 | 7.0 | 15.6 | 41.1 | 15.1 | 313.0 | 37.6 | 12.2 | 270.8 | 253.3 | 163.8 |
| ID518 | 18.9 | 37.9 | 12.4 | 108.9 | 34.8 | 12.9 | 71.6 | 55.4 | 11.8 | 336.3 | 251.9 | 150.6 |
| ID519 | 99.8 | 90.4 | 87.8 | 103.6 | 74.8 | 91.6 | 156.2 | 76.4 | 87.9 | 128.6 | 123.2 | 85.7 |
| ID52 | 67.8 | 224.0 | 101.7 | 76.5 | 66.2 | 61.4 | 123.9 | 295.7 | 82.8 | 61.8 | 62.0 | 36.6 |
| ID520 | 60.6 | 69.6 | 63.0 | 42.4 | 50.5 | 37.2 | 332.8 | 45.9 | 52.2 | 283.1 | 257.7 | 156.3 |
| ID521 | 89.1 | 90.5 | 86.0 | 74.0 | 87.8 | 73.0 | 142.9 | 85.5 | 72.0 | 165.0 | 141.6 | 109.9 |
| ID522 | 120.5 | 149.2 | 129.4 | 55.6 | 30.1 | 63.0 | 63.1 | 37.6 | 56.8 | 50.1 | 71.6 | 64.7 |
| ID523 | 82.7 | 86.6 | 73.3 | 82.7 | 81.5 | 75.4 | 116.7 | 81.2 | 84.8 | 121.8 | 112.8 | 110.3 |
| ID524 | 32.5 | 27.8 | 32.6 | 16.6 | 82.0 | 32.9 | 268.0 | 83.4 | 31.4 | 255.2 | 229.4 | 179.1 |
| ID525 | 55.8 | 22.0 | 36.1 | 27.8 | 58.5 | 18.6 | 541.5 | 52.8 | 14.6 | 468.8 | 272.9 | 164.6 |
| ID526 | 52.2 | 24.1 | 52.2 | 69.4 | 91.0 | 47.5 | 68.7 | 120.0 | 45.3 | 140.6 | 125.4 | 183.2 |
| ID527 | 83.6 | 34.7 | 68.2 | 73.7 | 110.0 | 23.0 | 338.4 | 57.6 | 168.6 | 189.0 | 234.0 | 163.2 |
| ID528 | 120.0 | 89.1 | 133.2 | 111.3 | 129.5 | 127.6 | 39.7 | 85.0 | 107.9 | 26.9 | 28.0 | 49.6 |
| ID529 | 71.8 | 78.1 | 92.7 | 90.2 | 99.7 | 105.3 | 76.5 | 107.4 | 105.9 | 76.6 | 75.3 | 98.3 |
| ID53 | 112.4 | 166.7 | 153.8 | 226.3 | 98.1 | 130.0 | 34.1 | 127.5 | 178.1 | 50.3 | 23.5 | 114.1 |
| ID530 | 125.7 | 124.8 | 136.1 | 127.7 | 116.9 | 135.9 | 30.3 | 107.9 | 130.5 | 37.3 | 36.0 | 56.8 |
| ID531 | 83.7 | 93.2 | 112.1 | 86.2 | 97.4 | 100.3 | 64.4 | 105.7 | 104.5 | 69.8 | 75.5 | 113.9 |
| ID532 | 44.8 | 33.7 | 39.6 | 45.1 | 89.2 | 42.8 | 172.4 | 91.4 | 57.9 | 195.0 | 177.4 | 180.9 |
| ID533 | 82.6 | 94.5 | 124.1 | 244.2 | 120.2 | 109.1 | 417.3 | 103.7 | 167.8 | 63.5 | 63.4 | 102.0 |
| ID534 | 85.0 | 72.3 | 75.6 | 66.0 | 109.4 | 62.8 | 97.4 | 105.9 | 67.2 | 127.8 | 113.8 | 132.5 |
| ID536 | 113.2 | 119.7 | 133.5 | 137.6 | 134.2 | 150.7 | 87.2 | 114.4 | 135.0 | 75.4 | 75.6 | 74.2 |
| ID537 | 103.2 | 108.2 | 121.2 | 125.7 | 152.8 | 76.2 | 156.1 | 112.6 | 112.9 | 84.8 | 117.7 | 85.7 |
| ID538 | 98.0 | 98.8 | 125.5 | 140.9 | 116.8 | 129.8 | 15.3 | 102.2 | 148.0 | 52.2 | 51.9 | 88.0 |
| ID539 | 74.7 | 73.9 | 88.0 | 64.8 | 92.0 | 90.0 | 68.7 | 105.7 | 91.0 | 78.4 | 80.4 | 93.7 |
| ID54 | 71.7 | 107.6 | 96.2 | 103.9 | 86.2 | 106.6 | 80.6 | 83.3 | 109.3 | 54.9 | 63.4 | 89.2 |
| ID540 | 69.2 | 72.7 | 72.8 | 66.8 | 77.8 | 61.4 | 151.2 | 79.0 | 60.2 | 180.7 | 137.0 | 130.8 |
| ID541 | 102.4 | 79.3 | 86.3 | 98.3 | 134.1 | 88.2 | 70.2 | 129.1 | 87.6 | 94.3 | 77.2 | 106.1 |
| ID542 | 1384.3 | 294.8 | 98.7 | 520.4 | 182.3 | 46.6 | 61.0 | 23.5 | 96.0 | 107.4 | 1015.7 | 305.5 |
| ID543 | 98.1 | 102.6 | 114.2 | 102.7 | 134.0 | 100.9 | 137.4 | 115.6 | 108.2 | 151.9 | 124.0 | 123.0 |
| ID544 | 64.3 | 57.9 | 76.6 | 44.5 | 120.8 | 72.3 | 71.3 | 139.8 | 59.3 | 92.8 | 96.6 | 149.8 |
| ID545 | 104.5 | 118.4 | 105.7 | 122.0 | 108.0 | 108.6 | 82.3 | 98.7 | 113.2 | 85.3 | 85.7 | 80.8 |
| ID546 | 62.4 | 58.8 | 60.1 | 51.4 | 64.1 | 43.4 | 212.9 | 58.2 | 44.5 | 210.5 | 185.4 | 134.6 |
| ID547 | 103.5 | 102.7 | 128.0 | 167.0 | 93.7 | 134.2 | 17.4 | 104.9 | 111.3 | 38.7 | 37.6 | 58.4 |
| ID548 | 82.2 | 86.4 | 91.7 | 97.7 | 81.4 | 104.7 | 66.4 | 108.5 | 104.6 | 65.1 | 67.2 | 95.8 |
| ID549 | 170.3 | 130.0 | 138.3 | 156.5 | 150.9 | 152.7 | 78.9 | 117.9 | 103.7 | 48.9 | 32.8 | 50.0 |
| ID55 | 4.9 | 114.5 | 161.9 | 818.4 | 63.8 | 85.9 | 167.8 | 98.4 |  |  | 123.9 | 102.4 |
| ID551 | 91.6 | 62.9 | 68.2 | 59.8 | 98.7 | 65.0 | 131.1 | 95.5 | 98.2 | 89.7 | 66.6 | 132.3 |
| ID552 | 106.9 | 98.7 | 112.2 | 156.6 | 100.5 | 113.4 | 112.0 | 105.4 | 140.8 | 146.2 | 59.2 | 65.9 |
| ID553 | 70.7 | 67.4 | 78.8 | 77.0 | 64.6 | 100.1 | 57.8 | 89.6 | 67.7 | 49.6 | 47.4 | 82.5 |
| ID554 | 76.9 | 84.2 | 75.4 | 60.4 | 86.9 | 55.6 | 159.3 | 82.1 | 52.1 | 161.9 | 151.7 | 126.7 |
| ID555 | 147.2 | 151.2 | 172.9 | 113.0 | 100.9 | 128.4 | 35.2 | 94.4 | 84.0 | 37.9 | 39.8 | 63.3 |
| ID556 | 95.0 | 110.8 | 107.6 | 107.2 | 107.3 | 113.4 | 98.5 | 92.7 | 101.8 | 91.8 | 78.1 | 70.8 |
| ID557 | 81.5 | 107.5 | 135.1 | 100.4 | 87.0 | 108.5 | 35.0 | 102.3 | 107.7 | 42.3 | 48.8 | 94.3 |
| ID558 | 83.8 | 87.1 | 90.5 | 113.8 | 89.0 | 100.4 | 122.7 | 104.9 | 104.1 | 112.2 | 103.5 | 100.3 |
| ID559 | 106.6 | 83.9 | 81.3 | 130.0 | 104.4 | 101.0 | 69.1 | 107.4 | 94.9 | 84.9 | 56.2 | 77.4 |
| ID56 | 77.5 | 79.5 | 57.1 | 73.9 | 76.8 | 56.1 | 127.4 | 52.6 | 69.8 | 145.2 | 174.4 | 135.0 |
| ID560 | 148.2 | 137.3 | 137.5 | 57.7 | 62.4 | 50.6 | 141.2 | 50.3 | 56.4 | 116.2 | 121.9 | 82.7 |
| ID561 | 75.2 | 71.5 | 68.8 | 76.9 | 92.5 | 67.0 | 126.6 | 87.7 | 84.6 | 124.6 | 116.8 | 119.5 |
| ID562 | 67.5 | 58.4 | 75.1 | 30.9 | 87.5 | 33.0 | 204.1 | 87.4 | 45.7 | 153.3 | 168.6 | 199.0 |
| ID563 | 122.1 | 90.2 | 105.0 | 85.9 | 72.1 | 78.0 | 116.5 | 68.8 | 75.2 | 110.7 | 78.0 | 79.1 |
| ID564 | 100.1 | 99.7 | 122.2 | 99.3 | 89.4 | 141.0 | 72.8 | 107.7 | 116.7 | 62.1 | 60.3 | 78.9 |
| ID565 | 156.7 | 150.5 | 160.8 | 113.3 | 109.3 | 127.8 | 48.1 | 92.4 | 95.7 | 51.6 | 45.8 | 53.5 |
| ID566 | 93.3 | 105.5 | 126.0 | 118.6 | 95.5 | 139.3 | 46.8 | 111.4 | 36.2 | 54.1 | 31.5 | 42.9 |
| ID567 | 108.1 | 112.1 | 136.7 | 128.4 | 99.5 | 146.3 | 20.0 | 101.4 | 115.0 | 31.0 | 29.3 | 48.6 |
| ID568 | 145.9 | 134.8 | 146.2 | 64.6 | 54.5 | 77.1 | 57.0 | 64.7 | 44.8 | 61.4 | 49.9 | 70.4 |
| ID569 | 236.5 | 91.3 | 59.5 | 110.7 | 230.4 | 59.3 | 174.4 | 109.4 | 1080.2 | 231.1 | 64.4 | 75.8 |
| ID57 | 82.2 | 104.2 | 106.1 | 172.5 | 82.5 | 110.9 | 27.6 | 78.2 | 113.6 | 55.3 | 61.1 | 82.4 |
| ID570 | 431.8 | 338.4 | 240.2 | 452.5 | 64.8 | 74.6 | 57.2 | 72.1 | 462.0 | 232.4 | 1318.9 | 546.0 |
| ID571 | 88.5 | 82.4 | 87.2 | 103.6 | 104.9 | 107.3 | 103.2 | 103.0 | 95.6 | 108.7 | 106.6 | 102.6 |
| ID572 | 113.1 | 111.2 | 116.0 | 129.1 | 114.0 | 120.6 | 54.1 | 104.9 | 131.0 | 54.4 | 52.7 | 67.9 |
| ID573 | 103.1 | 70.5 | 69.3 | 94.2 | 43.1 | 61.5 | 86.7 | 44.5 | 59.1 | 52.2 | 78.5 | 77.9 |
| ID574 | 119.0 | 59.6 | 78.0 | 45.7 | 96.3 | 55.4 | 149.8 | 95.3 | 41.9 | 142.4 | 123.9 | 141.8 |
| ID575 | 17.2 | 39.3 | 61.9 | 101.2 | 3.8 | 64.8 | 86.1 | 32.3 | 92.7 | 65.5 | 74.7 | 64.7 |
| ID576 | 97.2 | 98.4 | 101.6 | 87.6 | 95.9 | 96.6 | 115.9 | 90.0 | 79.4 | 89.1 | 91.8 | 89.2 |
| ID577 | 74.8 | 73.0 | 46.5 | 252.3 | 177.2 | 159.8 | 70.6 | 187.8 | 337.1 | 83.3 | 167.7 | 234.5 |
| ID578 | 139.8 | 121.4 | 77.0 | 270.7 | 78.9 | 66.3 | 53.4 | 102.4 | 171.5 | 64.3 | 426.8 | 148.8 |
| ID579 | 271.4 | 184.2 | 116.7 | 302.8 | 229.9 | 237.5 | 73.6 | 262.6 | 564.4 | 75.6 | 467.4 | 600.2 |
| ID58 | 114.1 | 25.3 | 97.9 | 95.3 | 95.0 | 60.1 | 67.7 | 76.2 | 91.1 | 68.9 | 127.9 | 117.9 |
| ID580 | 62.3 | 18.3 | 45.3 | 36.1 | 30.9 | 40.0 | 371.2 | 51.5 | 22.4 | 273.9 | 193.4 | 164.8 |
| ID581 | 1021.5 | 301.8 | 214.9 | 333.7 | 153.9 | 87.7 | 48.7 | 76.9 | 90.2 | 99.3 | 778.4 | 307.3 |
| ID582 | 95.3 | 90.3 | 95.7 | 66.7 | 91.6 | 80.0 | 186.8 | 85.6 | 63.0 | 96.9 | 103.0 | 88.7 |
| ID583 | 142.4 | 114.8 | 119.7 | 120.8 | 94.6 | 132.7 | 33.7 | 97.3 | 103.7 | 49.1 | 47.5 | 58.5 |
| ID584 | 93.4 | 91.3 | 99.3 | 83.6 | 119.9 | 93.2 | 100.2 | 120.8 | 85.0 | 110.4 | 102.6 | 110.9 |
| ID585 | 70.3 | 123.6 | 84.9 | 133.7 | 90.8 | 86.7 | 85.4 | 80.9 | 139.3 | 90.7 | 93.1 | 93.4 |
| ID586 | 92.7 | 70.6 | 85.3 | 57.2 | 128.2 | 72.6 | 122.4 | 111.1 | 74.3 | 137.7 | 117.9 | 128.4 |
| ID587 | 92.9 | 92.7 | 89.2 | 97.3 | 174.1 | 79.0 | 242.2 | 98.3 | 86.2 | 208.2 | 189.1 | 120.4 |
| ID588 | 107.6 | 122.9 | 164.6 | 112.2 | 88.5 | 134.5 | 39.6 | 84.9 | 97.6 | 38.4 | 53.2 | 59.0 |
| ID589 | 59.8 | 45.6 | 43.8 | 36.4 | 107.8 | 42.3 | 146.5 | 100.3 | 42.0 | 152.4 | 150.4 | 149.0 |
| ID59 | 79.6 | 201.5 | 101.5 | 1092.9 | 77.0 | 165.4 | 7.6 | 228.8 | 111.1 | 35.7 | 51.0 | 56.5 |
| ID590 | 120.1 | 127.1 | 178.3 | 124.8 | 133.7 | 168.2 | 32.9 | 129.3 | 108.2 | 39.1 | 25.6 | 64.8 |
| ID591 | 91.2 | 95.2 | 200.9 | 123.3 | 102.2 | 146.9 | 33.6 | 123.0 | 116.2 | 42.3 | 32.7 | 75.6 |
| ID592 | 70.7 | 66.1 | 74.2 | 105.2 | 102.1 | 108.4 | 110.8 | 114.3 | 91.2 | 118.6 | 96.7 | 116.1 |
| ID593 | 87.5 | 87.0 | 92.9 | 103.8 | 116.1 | 122.9 | 91.5 | 126.1 | 106.6 | 98.8 | 79.3 | 102.7 |
| ID594 | 83.0 | 75.5 | 66.3 | 77.3 | 87.7 | 48.0 | 198.0 | 77.4 | 57.4 | 212.8 | 171.5 | 136.4 |
| ID595 | 302.6 | 155.7 | 232.3 | 94.9 | 73.6 | 107.5 | 2.2 | 30.5 | 21.1 | 3.0 | 2.9 | 43.4 |
| ID596 | 77.8 | 230.2 | 699.7 | 161.5 | 186.4 | 130.6 | 16.8 | 41.4 | 172.0 | 80.7 | 0.9 | 31.9 |
| ID597 | 113.8 | 125.3 | 259.1 | 95.2 | 60.9 | 127.0 | 19.1 | 53.3 | 51.2 | 6.8 | 0.1 | 15.8 |
| ID598 | 149.5 | 218.2 | 110.9 | 234.1 | 60.6 | 154.4 | 80.1 | 134.2 | 157.1 | 57.6 | 399.4 | 330.3 |
| ID599 | 165.1 | 176.5 | 232.0 | 151.4 | 135.1 | 174.3 | 76.8 | 173.5 | 186.4 | 20.4 | 32.4 | 74.4 |
| ID6 | 116.7 | 86.0 | 84.0 | 113.2 | 111.8 | 82.9 | 46.4 | 91.1 | 137.7 | 78.7 | 141.3 | 142.6 |
| ID60 | 0.0 | 4.9 | 21.1 | 55.1 | 0.5 | 18.5 | 164.8 | 55.9 | 466.3 | 83.4 | 2961.6 | 513.4 |
| ID600 | 110.0 | 144.0 | 114.9 | 150.7 | 113.3 | 110.9 | 148.0 | 71.7 | 99.3 | 124.8 | 107.5 | 74.2 |
| ID601 | 172.7 | 143.2 | 139.1 | 139.2 | 117.5 | 135.4 | 60.7 | 104.6 | 135.8 | 91.2 | 89.2 | 87.6 |
| ID602 | 905.4 | 170.7 | 77.6 | 180.7 | 124.1 | 75.6 | 209.7 | 60.5 | 69.8 | 86.0 | 666.6 | 249.8 |
| ID603 | 78.1 | 72.6 | 47.4 | 42.2 | 69.6 | 54.7 | 285.2 | 51.8 | 49.7 | 186.8 | 171.1 | 105.7 |
| ID604 | 182.2 | 200.5 | 214.2 | 122.6 | 110.8 | 90.4 | 21.2 | 74.9 | 77.5 | 20.9 | 15.2 | 54.2 |
| ID605 | 132.6 | 128.5 | 136.8 | 124.7 | 113.9 | 132.7 | 33.9 | 110.9 | 119.1 | 42.2 | 41.3 | 58.6 |
| ID606 | 110.6 | 95.2 | 155.6 | 119.6 | 111.0 | 154.7 | 47.6 | 125.3 | 106.6 | 50.9 | 54.8 | 66.4 |
| ID607 | 146.1 | 115.2 | 134.5 | 111.2 | 104.3 | 88.5 | 50.2 | 78.3 | 95.5 | 45.7 | 40.0 | 57.6 |
| ID608 | 114.5 | 116.2 | 129.4 | 146.2 | 117.8 | 164.7 | 21.5 | 119.5 | 147.5 | 30.4 | 33.4 | 56.3 |
| ID61 | 0.5 | 43.3 | 0.0 | 78.0 | 42.7 | 55.4 | 171.5 | 77.4 | 102.3 | 210.8 | 47636.8 | 107.4 |
| ID611 | 87.6 | 93.0 | 108.3 | 110.4 | 121.9 | 138.6 | 64.6 | 134.7 | 108.1 | 73.8 | 55.8 | 93.1 |
| ID612 | 2213.4 | 850.3 | 633.0 | 929.2 | 1769.9 | 166.3 | 455.9 | 609.2 | ###### | 176055.0 | 267.4 | 145.5 |
| ID613 | 91.8 | 85.9 | 81.3 | 84.9 | 75.0 | 95.2 | 234.2 | 75.1 | 72.7 | 196.2 | 172.0 | 89.5 |
| ID614 | 110.6 | 81.2 | 127.0 | 67.5 | 66.3 | 135.0 | 81.7 | 140.2 | 51.4 | 104.5 | 37.7 | 103.7 |
| ID615 | 104.7 | 210.7 | 188.0 | 236.3 | 136.9 | 172.6 | 38.3 | 99.7 | 120.1 | 66.1 | 31.4 | 35.4 |
| ID616 | 101.7 | 72.9 | 79.6 | 88.4 | 104.9 | 86.5 | 101.8 | 111.1 | 103.3 | 112.4 | 117.8 | 112.6 |
| ID617 | 111.4 | 120.2 | 122.1 | 132.5 | 125.7 | 133.9 | 71.1 | 107.5 | 128.5 | 65.1 | 68.5 | 69.0 |
| ID618 | 132.2 | 126.8 | 154.1 | 127.8 | 122.6 | 145.3 | 46.4 | 115.7 | 121.2 | 49.5 | 45.2 | 58.2 |
| ID619 | 117.5 | 118.0 | 136.7 | 109.8 | 116.0 | 116.3 | 85.4 | 104.8 | 107.4 | 101.2 | 84.7 | 93.4 |
| ID62 | 92.2 | 130.1 | 104.0 | 113.2 | 91.8 | 101.5 | 68.4 | 81.6 | 85.8 | 78.7 | 71.8 | 88.2 |
| ID620 | 127.8 | 126.9 | 151.2 | 111.7 | 144.6 | 136.7 | 69.9 | 141.5 | 118.6 | 79.6 | 64.3 | 73.2 |
| ID621 | 101.9 | 65.2 | 68.8 | 94.7 | 92.9 | 70.2 | 81.6 | 86.0 | 88.2 | 131.6 | 138.3 | 115.5 |
| ID622 | 68.7 | 58.2 | 64.1 | 70.0 | 132.2 | 84.8 | 87.3 | 134.3 | 83.6 | 118.2 | 104.2 | 137.9 |
| ID623 | 83.8 | 139.7 | 130.8 | 307.6 | 129.7 | 155.1 | 40.9 | 119.0 | 174.5 | 56.9 | 50.7 | 65.3 |
| ID624 | 94.1 | 84.9 | 94.3 | 94.9 | 75.3 | 84.9 | 60.5 | 88.7 | 78.9 | 95.9 | 97.7 | 97.3 |
| ID625 | 118.4 | 145.7 | 150.7 | 134.1 | 103.7 | 147.9 | 29.2 | 95.6 | 133.5 | 106.2 | 18.7 | 38.8 |
| ID626 | 181.0 | 211.2 | 253.1 | 207.8 | 260.9 | 217.8 | 76.2 | 261.6 | 227.8 | 97.9 | 91.3 | 93.6 |
| ID627 | 88.2 | 80.2 | 91.8 | 65.2 | 122.8 | 74.7 | 134.8 | 115.4 | 62.5 | 146.0 | 118.3 | 122.6 |
| ID628 | 75.1 | 53.5 | 55.5 | 59.9 | 85.0 | 67.6 | 152.5 | 94.0 | 58.9 | 203.0 | 151.9 | 151.3 |
| ID629 | 86.6 | 76.0 | 77.9 | 59.9 | 70.4 | 62.8 | 171.3 | 68.9 | 46.0 | 202.6 | 141.1 | 114.8 |
| ID630 | 44.7 | 41.1 | 42.2 | 31.8 | 75.3 | 32.9 | 281.4 | 72.6 | 30.4 | 266.7 | 200.0 | 148.6 |
| ID631 | 120.0 | 100.1 | 123.7 | 120.2 | 111.3 | 152.0 | 138.4 | 120.2 | 84.7 | 111.9 | 77.8 | 115.3 |
| ID632 | 136.2 | 131.2 | 154.5 | 113.3 | 118.7 | 127.1 | 62.4 | 107.2 | 112.6 | 57.1 | 51.3 | 56.7 |
| ID633 | 75.1 | 67.3 | 79.3 | 64.1 | 78.0 | 66.6 | 231.9 | 69.6 | 63.1 | 186.6 | 153.7 | 93.7 |
| ID634 | 161.6 | 253.7 | 250.9 | 143.2 | 82.6 | 98.7 | 103.5 | 100.6 | 141.7 | 125.0 | 57.6 | 83.5 |
| ID635 | 78.7 | 71.7 | 85.5 | 58.8 | 117.6 | 61.7 | 177.7 | 107.9 | 54.0 | 176.0 | 134.5 | 126.5 |
| ID636 | 80.6 | 102.2 | 70.0 | 110.8 | 62.4 | 63.1 | 34.5 | 76.9 | 99.2 | 26.5 | 33.8 | 30.9 |
| ID637 | 142.4 | 135.4 | 77.5 | 220.9 | 87.7 | 69.7 | 53.8 | 68.8 | 167.8 | 68.9 | 349.6 | 178.3 |
| ID638 | 604.5 | 190.6 | 155.5 | 194.7 | 97.8 | 50.1 | 36.8 | 15.0 | 135.7 | 107.8 | 725.3 | 270.0 |
| ID639 | 79.8 | 50.1 | 44.6 | 26.4 | 61.0 | 23.5 | 298.1 | 51.8 | 30.4 | 196.0 | 172.0 | 147.8 |
| ID64 | 120.8 | 143.4 | 102.8 | 188.3 | 53.6 | 93.2 | 71.9 | 102.5 | 150.8 | 66.0 | 149.7 | 137.9 |
| ID640 | 183.9 | 193.9 | 136.6 | 203.2 | 104.7 | 184.9 | 59.6 | 50.5 | 151.6 | 46.6 | 99.7 | 121.2 |
| ID641 | 50.2 | 41.6 | 38.3 | 47.3 | 83.8 | 45.8 | 134.9 | 104.0 | 54.0 | 132.7 | 134.4 | 148.7 |
| ID642 | 117.2 | 110.4 | 106.3 | 125.0 | 96.0 | 121.1 | 27.7 | 105.6 | 120.9 | 32.2 | 34.6 | 54.4 |
| ID643 | 1072.0 | 865.6 | 3622.0 | 1131.6 | 1216.6 | 278.7 | 1523.3 | 1065.6 | 1629.8 | 2011.7 | 4242.3 | 251.7 |
| ID644 | 28.1 | 37.4 | 37.2 | 19.2 | 37.3 | 18.2 | 306.8 | 64.6 | 23.6 | 248.2 | 231.8 | 163.3 |
| ID645 | 228.8 | 192.7 | 231.6 | 74.6 | 63.7 | 95.3 | 19.0 | 59.1 | 76.1 | 20.7 | 7.5 | 22.5 |
| ID646 | 116.7 | 111.1 | 100.9 | 110.3 | 90.0 | 102.4 | 70.6 | 77.7 | 97.3 | 58.9 | 63.1 | 74.4 |
| ID648 | 336.2 | 126.4 | 656.3 | 109.8 | 394.7 | 768.0 | 124.6 | 96.2 | 336.6 | 159.8 | 56.4 | 211.0 |
| ID649 | 139.1 | 131.0 | 133.1 | 52.1 | 61.7 | 60.8 | 346.6 | 43.4 | 49.0 | 131.5 | 100.3 | 77.4 |
| ID650 | 75.4 | 64.5 | 51.2 | 46.4 | 64.6 | 42.1 | 148.6 | 62.0 | 52.6 | 121.0 | 131.9 | 125.3 |
| ID651 | 89.2 | 47.2 | 26.4 | 22.9 | 28.5 | 21.1 | 225.0 | 48.6 | 83.5 | 63.6 | 62.9 | 71.2 |
| ID652 | 219.1 | 169.8 | 84.8 | 236.8 | 104.9 | 31.4 | 41.3 | 63.7 | 207.8 | 77.3 | 437.2 | 211.4 |
| ID653 | 241.3 | 119.9 | 219.7 | 114.4 | 51.3 | 103.3 | 17.3 | 52.4 | 66.6 | 16.1 | 31.8 | 47.6 |
| ID654 | 868.6 | 166.6 | 106.7 | 280.8 | 139.4 | 46.5 | 34.0 | 9.4 | 190.9 | 102.3 | 609.2 | 264.2 |
| ID655 | 121.8 | 108.5 | 105.9 | 135.7 | 104.7 | 120.0 | 14.7 | 78.8 | 123.8 | 22.1 | 27.4 | 46.2 |
| ID656 | 84.3 | 67.8 | 57.7 | 42.9 | 104.5 | 51.4 | 91.3 | 88.5 | 53.6 | 93.9 | 104.5 | 127.4 |
| ID657 | 115.7 | 110.6 | 86.4 | 85.9 | 66.9 | 81.6 | 33.0 | 68.2 | 63.1 | 31.3 | 42.7 | 71.0 |
| ID658 | 24.8 | 21.3 | 22.5 | 205.1 | 49.8 | 28.0 | 102.5 | 65.5 | 27.0 | 184.9 | 193.9 | 163.3 |
| ID659 | 175.5 | 127.4 | 193.6 | 11.4 | 23.9 | 23.0 | 51.3 | 16.6 | 22.3 | 76.3 | 103.2 | 107.3 |
| ID66 | 54.0 | 117.4 | 81.6 | 146.5 | 47.2 | 82.2 | 70.2 | 78.4 | 102.8 | 71.7 | 116.1 | 113.6 |
| ID660 | 268.8 | 212.3 | 250.5 | 113.6 | 102.8 | 130.5 | 0.5 | 78.5 | 90.9 | 15.6 | 8.0 | 38.5 |
| ID661 | 123.1 | 115.6 | 146.5 | 134.9 | 168.8 | 152.9 | 88.4 | 140.9 | 130.8 | 93.7 | 75.9 | 82.3 |
| ID662 | 117.4 | 56.5 | 8.9 | 113.9 | 10.7 | 50.8 | 18.1 | 4.1 | 113.8 | 10.2 | 38.1 | 56.5 |
| ID663 | 118.6 | 131.1 | 94.3 | 103.9 | 95.2 | 97.2 | 18.7 | 76.6 | 97.8 | 19.9 | 50.1 | 57.6 |
| ID664 | 65.0 | 48.9 | 68.4 | 60.8 | 133.4 | 57.0 | 234.8 | 112.5 | 42.3 | 269.0 | 182.5 | 143.0 |
| ID665 | 702.3 | 193.9 | 101.7 | 120.1 | 619.3 | 64.3 | 22.5 | 214.6 | 106.8 | 52.8 | 68.3 | 87.8 |
| ID666 | 166.1 | 103.2 | 129.5 | 188.4 | 161.6 | 115.7 | 141.5 | 130.7 | 138.5 | 147.6 | 139.5 | 122.8 |
| ID667 | 62.9 | 37.0 | 58.4 | 69.0 | 118.6 | 43.9 | 289.4 | 80.8 | 26.9 | 423.2 | 207.8 | 129.5 |
| ID668 | 81.5 | 141.4 | 135.8 | 116.1 | 123.9 | 106.7 | 68.7 | 186.1 | 84.8 | 44.5 | 59.1 | 83.0 |
| ID669 | 13.8 | 26.1 | 240.3 | 216.2 | 1.0 | 0.0 | 656.0 | 10.5 | 121.4 | 101.8 | 186.2 | 80.0 |
| ID67 | 48.4 | 102.6 | 102.2 | 211.8 | 59.6 | 104.9 | 81.3 | 99.1 | 471.0 | 98.6 | 139.8 | 92.6 |
| ID670 | 115.5 | 137.5 | 102.1 | 91.1 | 81.1 | 107.9 | 7.9 | 61.1 | 125.6 | 27.4 | 39.5 | 41.4 |
| ID671 | 126.9 | 74.0 | 105.9 | 0.0 | 89.9 | 14.1 | 358.2 | 90.8 | 10.9 | 268.3 | 272.6 | 191.4 |
| ID672 | 196.3 | 191.3 | 200.5 | 533.3 | 261.5 | 297.1 | 0.7 | 162.3 | 715.2 | 0.0 | 17.6 | 30.5 |
| ID673 | 28.8 | 42.0 | 28.1 | 29.8 | 56.4 | 58.1 | 309.0 | 59.2 | 28.3 | 204.6 | 169.1 | 108.6 |
| ID674 | 140.5 | 85.7 | 104.3 | 1.5 | 53.7 | 19.3 | 97.3 | 96.4 | 20.9 | 134.9 | 223.9 | 281.1 |
| ID676 | 115.7 | 115.4 | 111.8 | 128.8 | 114.3 | 122.2 | 51.6 | 105.9 | 130.5 | 50.6 | 55.2 | 70.8 |
| ID677 | 123.5 | 107.7 | 200.4 | 177.0 | 153.2 | 83.3 | 47.3 | 90.6 | 229.0 | 192.3 | 45.4 | 57.3 |
| ID678 | 166.2 | 128.5 | 212.4 | 186.9 | 366.4 | 252.2 | 39.4 | 256.7 | 391.9 | 344.2 | 40.8 | 69.4 |
| ID679 | 72.7 | 202.7 | 89.6 | 98.1 | 131.8 | 103.1 | 170.9 | 232.6 | 85.2 | 163.6 | 156.0 | 156.5 |
| ID68 | 8.8 | 32.1 | 3.5 | 0.0 | 15.3 | 8.0 | 813.8 | 24.3 | 0.3 | 363.8 | 461.1 | 108.8 |
| ID681 | 12.7 | 107.8 | 22.3 | 351.3 | 37.3 | 260.2 | 139.9 | 146.3 | 179.0 | 43.3 | 6.5 | 186.4 |
| ID682 | 75.5 | 106.8 | 138.4 | 115.5 | 59.8 | 109.1 | 58.7 | 60.2 | 0.3 | 1.2 | 70.1 | 94.5 |
| ID683 | 481.3 | 284.1 | 304.8 | 470.3 | 512.2 | 144.5 | 484.1 | 334.3 | 189.4 | 328.0 | 586.9 | 159.9 |
| ID684 | 81.8 | 105.7 | 94.2 | 91.4 | 88.9 | 36.8 | 137.9 | 88.1 | 17.6 | 15.9 | 130.9 | 108.7 |
| ID685 | 108.9 | 90.3 | 104.8 | 117.4 | 111.8 | 118.4 | 73.0 | 86.7 | 91.1 | 53.8 | 66.6 | 66.9 |
| ID687 | 76.7 | 147.8 | 53.6 | 242.7 | 95.9 | 124.6 | 43.2 | 201.0 | 131.3 | 52.1 | 17.8 | 67.5 |
| ID688 | 60.5 | 15.3 | 85.4 | 41.6 | 108.9 | 93.0 | 389.7 | 99.0 | 20.1 | 328.1 | 314.5 | 216.4 |
| ID689 | 104.0 | 17.4 | 286.4 | 61.7 | 14.9 | 108.0 | 7.1 | 1.2 | 1811.3 | 0.0 | 31.1 | 11.8 |
| ID69 | 68.9 | 100.1 | 96.1 | 191.1 | 68.5 | 88.7 | 95.3 | 91.5 | 159.3 | 86.2 | 319.0 | 95.7 |
| ID690 | 96.5 | 92.5 | 189.9 | 136.8 | 109.6 | 193.1 | 62.8 | 202.2 | 101.5 | 74.1 | 81.2 | 263.2 |
| ID691 | 99.3 | 72.0 | 81.5 | 126.2 | 46.3 | 83.3 | 10.1 | 74.3 | 82.0 | 15.3 | 22.8 | 41.3 |
| ID692 | 237.6 | 215.1 | 292.6 | 151.7 | 171.8 | 138.2 | 70.8 | 110.9 | 92.9 | 101.3 | 64.6 | 59.4 |
| ID693 | 146.1 | 243.5 | 336.5 | 165.2 | 95.6 | 162.1 | 111.8 | 203.5 | 80.7 | 50.2 | 91.9 | 66.3 |
| ID694 | 269.4 | 258.1 | 313.7 | 181.7 | 250.8 | 217.8 | 117.3 | 188.5 | 84.3 | 60.5 | 83.9 | 65.7 |
| ID695 | 138.8 | 144.0 | 179.9 | 101.2 | 102.0 | 107.8 | 65.3 | 80.2 | 82.4 | 50.2 | 61.0 | 72.5 |
| ID696 | 94.6 | 112.0 | 73.3 | 73.1 | 84.0 | 87.3 | 38.6 | 89.6 | 108.0 | 45.2 | 56.4 | 83.2 |
| ID697 | 880.7 | 599.2 | 0.0 | 226.8 | 499.6 | 126.0 | 238.5 | 320.8 | 178.3 | 336.0 | 126.0 | 114.2 |
| ID698 | 101.7 | 102.1 | 125.7 | 57.9 | 94.2 | 70.1 | 148.5 | 92.5 | 49.1 | 167.2 | 143.4 | 169.7 |
| ID699 | 68.3 | 73.3 | 113.1 | 87.9 | 50.4 | 127.1 | 220.0 | 87.7 | 34.6 | 141.0 | 139.3 | 142.0 |
| ID7 | 89.7 | 59.0 | 72.0 | 95.3 | 74.5 | 69.1 | 97.7 | 83.8 | 54.2 | 105.9 | 114.9 | 106.0 |
| ID70 | 133.7 | 125.7 | 140.7 | 114.3 | 107.8 | 132.2 | 28.9 | 97.4 | 125.2 | 23.2 | 51.3 | 45.8 |
| ID700 | 32.0 | 77.6 | 75.5 | 68.4 | 72.2 | 62.9 | 154.3 | 94.6 | 49.1 | 122.3 | 141.8 | 181.3 |
| ID701 | 1179.4 | 792.8 |  | 450.7 | 726.0 | 156.7 | 227.7 | 421.3 | 162.4 | 171.2 |  | 142.4 |
| ID702 | 82.9 | 175.5 | 161.4 | 156.1 | 112.3 | 163.0 | 149.1 | 194.4 | 86.0 | 98.9 | 89.1 | 96.8 |
| ID704 | 90.7 | 115.8 | 145.9 | 126.0 | 109.3 | 172.6 | 126.6 | 131.8 | 91.3 | 104.6 | 105.5 | 138.2 |
| ID705 | 135.7 | 339.0 | 315.8 | 161.9 | 126.1 | 259.4 | 127.7 | 269.5 | 116.3 | 91.5 | 324.9 | 222.6 |
| ID707 | 164.2 | 120.7 | 177.9 | 108.6 | 109.6 | 115.8 | 38.4 | 90.1 | 83.6 | 46.8 | 38.2 | 41.6 |
| ID708 | 161.7 | 147.3 | 163.3 | 103.6 | 89.7 | 115.4 | 42.8 | 84.3 | 87.4 | 43.2 | 39.9 | 57.5 |
| ID709 | 89.7 | 50.1 | 79.5 | 27.6 | 90.0 | 35.8 | 221.1 | 113.7 | 13.8 | 169.8 | 159.3 | 181.3 |
| ID71 | 67.9 | 101.0 | 111.1 | 267.3 | 64.0 | 94.6 | 106.2 | 94.3 | 132.3 | 72.6 | 133.5 | 92.8 |
| ID710 | 191.1 | 157.0 | 181.4 | 98.2 | 79.2 | 92.9 | 50.0 | 60.0 | 82.0 | 35.8 | 33.2 | 44.8 |
| ID712 | 52.4 | 31.8 | 51.6 | 77.6 | 187.5 | 33.9 | 270.5 | 115.4 | 71.9 | 252.1 | 516.5 | 442.1 |
| ID713 | 242.2 | 382.5 | 346.4 | 378.5 | 187.9 | 385.6 | 330.4 | 363.5 | 355.4 | 341.8 | 395.1 | 380.5 |
| ID714 | 77.3 | 74.2 | 76.1 | 28.4 | 85.2 | 42.4 | 328.1 | 103.0 | 52.5 | 357.2 | 274.1 | 205.5 |
| ID715 | 1209.3 | 875.3 | 1630.7 | 945.6 | 3024.9 | 2483.5 | 965.2 | 1465.7 | 588.3 | 699.2 | 597.0 | 774.9 |
| ID716 | 211.0 | 228.7 | 261.9 | 145.7 | 123.1 | 145.9 | 70.3 | 115.5 | 123.7 | 24.4 | 30.1 | 53.2 |
| ID717 | 20.4 | 38.8 | 40.7 | 87.9 | 94.9 | 100.6 | 564.4 | 62.8 | 76.2 | 344.4 | 322.7 | 165.0 |
| ID719 | 104.3 | 90.2 | 101.5 | 182.4 | 140.6 | 157.5 | 42.2 | 138.8 | 149.3 | 71.1 | 59.3 | 66.4 |
| ID72 | 115.3 | 147.7 | 158.2 | 223.6 | 81.8 | 167.2 | 25.7 | 75.7 | 137.4 | 14.4 | 88.3 | 36.3 |
| ID720 | 122.7 | 75.8 | 322.4 | 66.6 | 121.4 | 220.4 | 57.0 | 10.5 | 419.7 | 0.0 | 55.6 | 133.1 |
| ID721 | 107.6 | 106.5 | 127.1 | 137.9 | 150.8 | 149.0 | 73.1 | 129.1 | 131.4 | 72.4 | 65.5 | 70.3 |
| ID722 | 82.7 | 84.8 | 80.7 | 114.2 | 95.0 | 115.6 | 65.7 | 86.2 | 128.5 | 53.0 | 56.3 | 73.8 |
| ID723 | 315.9 | 198.4 | 195.7 | 243.1 | 176.8 | 195.8 | 169.6 | 183.1 | 191.5 | 155.4 | 208.7 | 225.0 |
| ID724 | 163.6 | 140.2 | 134.9 | 110.8 | 104.5 | 110.3 | 55.5 | 94.1 | 106.8 | 61.5 | 66.3 | 87.6 |
| ID725 | 189.4 | 98.8 | 118.9 | 165.9 | 155.0 | 129.4 | 145.7 | 102.6 | 100.0 | 165.5 | 145.8 | 133.0 |
| ID726 | 96.6 | 99.7 | 106.9 | 109.1 | 180.7 | 111.5 | 192.6 | 132.9 | 99.8 | 157.8 | 135.5 | 115.0 |
| ID727 | 130.3 | 108.2 | 101.0 | 59.0 | 79.0 | 57.2 | 179.9 | 83.1 | 48.7 | 157.8 | 138.1 | 124.9 |
| ID728 | 118.0 | 73.5 | 77.6 | 83.7 | 124.4 | 74.9 | 118.8 | 114.0 | 90.3 | 151.2 | 159.5 | 173.1 |
| ID729 | 112.6 | 55.4 | 38.4 | 70.4 | 226.4 | 24.5 | 120.8 | 43.7 | 74.9 | 102.4 | 86.8 | 54.5 |
| ID73 | 117.6 | 184.9 | 1774.4 | 133.1 | 130.5 | 136.0 | 80.1 | 206.3 | 146.3 | 39.1 | 11.9 | 96.7 |
| ID730 | 909.4 | 757.5 | 251.3 | 638.1 | 774.4 | 228.1 | 349.2 | 625.0 | 99.2 | 199.1 | 257.8 | 188.3 |
| ID731 | 172.7 | 162.7 | 161.1 | 43.8 | 41.1 | 45.6 | 114.0 | 46.0 | 31.8 | 75.7 | 68.3 | 46.1 |
| ID732 | 88.4 | 87.7 | 110.1 | 113.3 | 110.4 | 105.8 | 80.9 | 135.1 | 161.4 | 95.2 | 106.3 | 103.2 |
| ID733 | 33.2 | 21.3 | 22.4 | 54.6 | 37.4 | 42.8 | 278.2 | 43.0 | 64.1 | 179.6 | 195.2 | 89.1 |
| ID734 | 114.2 | 91.9 | 107.8 | 131.1 | 136.0 | 96.7 | 105.9 | 112.6 | 108.0 | 115.6 | 96.1 | 92.6 |
| ID735 | 123.6 | 96.4 | 88.1 | 97.3 | 80.9 | 89.4 | 78.3 | 78.7 | 97.3 | 76.2 | 74.0 | 74.3 |
| ID736 | 74.1 | 65.9 | 147.9 | 104.0 | 92.2 | 118.0 | 68.8 | 109.6 | 97.2 | 49.8 | 107.7 | 122.1 |
| ID737 | 131.5 | 120.4 | 117.1 | 110.3 | 92.5 | 92.6 | 47.1 | 81.1 | 100.0 | 53.5 | 55.5 | 62.1 |
| ID738 | 123.9 | 115.3 | 112.1 | 114.4 | 110.3 | 108.6 | 51.8 | 92.8 | 110.8 | 58.8 | 56.6 | 71.3 |
| ID739 | 261.5 | 208.3 | 237.0 | 27.4 | 43.9 | 28.7 | 10.1 | 18.0 | 29.7 | 9.5 | 45.3 | 30.4 |
| ID74 | 86.6 | 71.9 | 241.5 | 142.9 | 80.1 | 95.2 | 90.5 | 103.3 | 99.3 | 76.7 | 290.4 | 99.9 |
| ID740 | 32.5 | 29.8 | 27.6 | 30.5 | 40.7 | 24.0 | 295.2 | 42.6 | 34.1 | 244.4 | 212.1 | 129.8 |
| ID741 | 118.9 | 114.2 | 115.1 | 124.7 | 101.6 | 118.7 | 48.6 | 94.5 | 111.3 | 55.4 | 50.3 | 71.8 |
| ID742 | 138.1 | 114.0 | 118.0 | 88.7 | 82.6 | 101.0 | 32.9 | 93.8 | 93.8 | 57.1 | 63.7 | 126.9 |
| ID743 | 65.1 | 49.1 | 48.3 | 46.4 | 86.4 | 36.3 | 170.1 | 77.7 | 40.8 | 175.3 | 169.6 | 150.4 |
| ID744 | 437.9 | 319.7 | 148.8 | 303.8 | 412.8 | 152.2 | 246.0 | 317.2 | 88.1 | 220.4 | 176.6 | 142.7 |
| ID745 | 120.7 | 98.9 | 108.3 | 95.2 | 93.5 | 82.3 | 81.0 | 81.7 | 90.5 | 91.8 | 86.8 | 91.9 |
| ID746 | 69.6 | 39.3 | 56.0 | 14.0 | 44.4 | 17.5 | 145.3 | 54.9 | 14.5 | 206.0 | 206.1 | 266.1 |
| ID747 | 118.2 | 95.4 | 94.0 | 94.2 | 84.7 | 84.4 | 98.5 | 75.5 | 92.8 | 94.1 | 95.8 | 88.8 |
| ID748 | 150.6 | 103.5 | 106.7 | 113.1 | 99.2 | 94.8 | 85.5 | 78.4 | 101.3 | 79.6 | 86.5 | 80.4 |
| ID749 | 30.5 | 35.2 | 40.5 | 43.6 | 62.9 | 23.5 | 328.0 | 45.3 | 38.1 | 280.2 | 278.4 | 156.5 |
| ID75 | 62.8 | 147.7 | 93.0 | 126.0 | 67.1 | 85.4 | 82.1 | 89.0 | 130.4 | 85.0 | 153.6 | 124.1 |
| ID750 | 261.2 | 138.6 | 131.1 | 392.2 | 128.9 | 80.3 | 176.0 | 93.6 | 131.3 | 129.1 | 94.3 | 89.1 |
| ID751 | 39.3 | 24.2 | 197.3 | 64.7 | 64.6 | 11.4 | 325.4 | 68.5 | 94.3 | 181.4 | 278.2 | 159.8 |
| ID752 | 180.8 | 180.5 | 150.0 | 41.7 | 43.1 | 38.1 | 38.9 | 23.3 | 23.5 | 56.8 | 55.7 | 47.1 |
| ID753 | 210.4 | 127.2 | 40.9 | 146.4 | 73.1 | 49.4 | 28.2 | 41.1 | 147.5 | 50.0 | 166.4 | 160.2 |
| ID756 | 534.6 | 370.5 | 235.7 | 266.6 | 419.7 | 156.9 | 204.3 | 306.1 | 123.5 | 161.0 | 285.8 | 120.1 |
| ID757 | 72.2 | 66.4 | 46.3 | 80.6 | 74.9 | 62.7 | 184.0 | 130.6 | 103.8 | 65.7 | 78.3 | 142.4 |
| ID758 | 134.4 | 145.6 | 173.1 | 152.9 | 119.4 | 155.2 | 29.4 | 115.0 | 130.8 | 44.0 | 34.2 | 63.3 |
| ID759 | 124.8 | 83.2 | 67.2 | 115.7 | 96.7 | 87.7 | 159.6 | 60.7 | 59.9 | 121.9 | 168.0 | 88.9 |
| ID76 | 79.3 | 77.1 | 99.9 | 74.2 | 117.3 | 79.3 | 50.8 | 184.8 | 99.5 | 74.6 | 42.9 | 131.9 |
| ID760 | 146.6 | 268.4 | 156.5 | 132.0 | 121.2 | 107.0 | 156.5 | 196.3 | 119.1 | 161.3 | 131.2 | 95.2 |
| ID761 | 35.7 | 28.7 | 30.3 | 34.9 | 47.5 | 20.6 | 272.9 | 36.2 | 19.0 | 232.5 | 228.1 | 124.4 |
| ID762 | 148.2 | 98.8 | 41.8 | 156.7 | 115.5 | 96.9 | 287.1 | 81.7 | 109.3 | 129.0 | 233.4 | 81.2 |
| ID763 | 292.0 | 228.4 | 233.3 | 98.9 | 67.2 | 105.7 | 24.1 | 65.3 | 74.0 | 18.4 | 12.2 | 49.4 |
| ID764 | 121.6 | 104.3 | 111.2 | 102.0 | 106.2 | 34.1 | 103.9 | 37.2 | 80.9 | 38.5 | 98.1 | 59.2 |
| ID765 | 94.5 | 115.1 | 122.4 | 104.5 | 104.8 | 133.7 | 30.2 | 102.6 | 104.8 | 37.1 | 30.1 | 53.5 |
| ID766 | 679.3 | 448.7 | 160.0 | 355.4 | 511.4 | 149.4 | 213.9 | 328.1 | 105.9 | 158.4 | 184.5 | 116.4 |
| ID767 | 113.5 | 70.4 | 90.6 | 73.0 | 134.1 | 82.7 | 124.4 | 121.0 | 69.8 | 178.6 | 163.3 | 190.0 |
| ID768 | 53.2 | 58.6 | 65.3 | 78.1 | 105.4 | 79.6 | 128.5 | 106.9 | 55.1 | 148.1 | 109.4 | 112.7 |
| ID769 | 89.1 | 85.1 | 72.8 | 119.1 | 114.2 | 129.8 | 21.5 | 108.2 | 142.7 | 50.3 | 61.5 | 93.5 |
| ID77 | 101.2 | 25.3 | 128.3 | 343.0 | 90.2 | 139.8 | 0.7 | 46.3 | 103.6 | 66.2 | 57.0 | 68.0 |
| ID770 | 13.9 | 202.4 | 299.3 | 220.0 | 24.1 | 330.1 | 214.7 | 261.2 | 142.5 | 207.2 | 163.6 | 172.0 |
| ID771 | 110.8 | 31.6 | 104.0 | 65.3 | 55.3 | 65.6 | 363.2 | 105.4 | 27.0 | 356.9 | 447.2 | 207.3 |
| ID772 | 229.9 | 163.7 | 205.7 | 80.2 | 75.2 | 86.5 | 13.3 | 69.2 | 71.3 | 25.4 | 33.4 | 68.2 |
| ID773 | 101.9 | 104.2 | 103.5 | 112.3 | 78.3 | 115.9 | 68.2 | 94.7 | 107.7 | 75.6 | 62.3 | 87.2 |
| ID774 | 198.3 | 233.7 | 234.1 | 105.1 | 106.5 | 91.8 | 86.4 | 95.4 | 76.2 | 61.8 | 45.0 | 51.8 |
| ID775 | 139.6 | 109.0 | 131.8 | 114.3 | 157.3 | 96.6 | 112.8 | 116.3 | 113.3 | 126.2 | 134.4 | 151.0 |
| ID776 | 218.7 | 233.0 | 281.1 | 146.7 | 103.8 | 93.0 | 14.5 | 58.4 | 42.3 | 21.2 | 26.8 | 29.1 |
| ID777 | 65.4 | 66.4 | 140.1 | 112.5 | 69.0 | 42.1 | 155.3 | 72.0 | 108.5 | 95.1 | 156.5 | 121.0 |
| ID778 | 83.6 | 46.4 | 61.8 | 51.7 | 48.3 | 47.7 | 48.8 | 43.5 | 35.1 | 42.6 | 39.9 | 49.3 |
| ID779 | 138.2 | 100.1 | 86.0 | 117.5 | 159.4 | 129.1 | 25.1 | 96.2 | 111.1 | 42.8 | 42.6 | 119.3 |
| ID78 | 38.9 | 70.8 | 74.3 | 46.9 | 84.0 | 27.9 | 221.2 | 80.9 | 0.9 | 386.2 | 155.8 | 153.6 |
| ID780 | 50.0 | 29.3 | 45.3 | 31.9 | 111.6 | 30.6 | 333.2 | 75.4 | 33.3 | 286.8 | 271.9 | 225.8 |
| ID782 | 47.2 | 21.7 | 6.8 | 25.5 | 136.8 | 7.5 | 12.9 | 6.1 | 31.6 | 29.1 | 12.0 | 14.6 |
| ID783 | 98.0 | 24.2 | 6.5 | 27.9 | 71.3 | 5.0 | 12.3 | 7.5 | 34.3 | 8.2 | 18.1 | 23.9 |
| ID784 | 105.9 | 66.4 | 10.4 | 36.6 | 94.1 | 17.2 | 17.6 | 19.0 | 40.4 | 35.2 | 33.6 | 45.1 |
| ID785 | 254.7 | 177.3 | 183.5 | 95.9 | 70.2 | 102.8 | 13.8 | 95.9 | 71.7 | 48.3 | 42.1 | 76.0 |
| ID786 | 119.3 | 124.3 | 126.0 | 158.6 | 140.9 | 145.8 | 78.8 | 131.5 | 150.2 | 81.0 | 73.8 | 81.6 |
| ID787 | 62.6 | 43.4 | 60.7 | 52.7 | 122.0 | 66.4 | 206.2 | 106.3 | 58.8 | 171.4 | 149.6 | 124.3 |
| ID788 | 222.3 | 266.6 | 292.4 | 286.5 | 248.4 | 381.1 | 127.3 | 282.5 | 259.4 | 129.0 | 106.4 | 179.3 |
| ID789 | 143.9 | 140.2 | 173.2 | 153.5 | 132.7 | 110.8 | 77.7 | 99.9 | 122.8 | 86.3 | 74.8 | 74.2 |
| ID79 | 43.8 | 57.8 | 72.6 | 91.4 | 82.0 | 57.3 | 119.1 | 109.5 | 94.8 | 154.5 | 165.6 | 217.8 |
| ID790 | 87.7 | 56.7 | 55.8 | 60.3 | 120.5 | 51.3 | 306.9 | 115.3 | 72.6 | 208.4 | 172.3 | 154.8 |
| ID791 | 52.9 | 56.1 | 51.0 | 38.0 | 99.8 | 49.5 | 225.8 | 115.2 | 52.0 | 193.2 | 163.1 | 137.0 |
| ID792 | 62.9 | 66.9 | 63.3 | 72.3 | 82.6 | 55.4 | 189.9 | 76.1 | 66.9 | 175.8 | 146.7 | 100.1 |
| ID793 | 968.1 | 632.1 | 168.4 | 513.3 | 1028.7 | 205.9 | 359.4 | 694.6 | 191.2 | 141.0 | 120.4 | 169.7 |
| ID794 | 30.4 | 34.6 | 35.9 | 33.0 | 66.7 | 28.5 | 235.3 | 88.2 | 30.4 | 217.3 | 169.4 | 116.6 |
| ID795 | 109.9 | 99.8 | 118.8 | 61.2 | 82.9 | 65.8 | 184.6 | 68.3 | 57.7 | 148.5 | 127.4 | 99.6 |
| ID796 | 105.4 | 105.1 | 119.3 | 125.9 | 131.2 | 128.3 | 75.0 | 108.4 | 125.8 | 74.6 | 69.1 | 72.0 |
| ID797 | 74.5 | 57.3 | 57.2 | 70.9 | 97.0 | 67.4 | 240.5 | 116.2 | 68.7 | 178.8 | 174.0 | 128.2 |
| ID798 | 60.0 | 37.8 | 47.5 | 32.0 | 81.1 | 44.0 | 233.7 | 76.1 | 46.8 | 194.7 | 139.0 | 110.1 |
| ID799 | 68.8 | 48.9 | 69.0 | 51.9 | 130.3 | 66.8 | 219.0 | 119.5 | 65.0 | 197.3 | 149.1 | 129.2 |
| ID8 | 101.5 | 83.0 | 69.0 | 90.8 | 84.5 | 74.0 | 98.0 | 95.7 | 65.1 | 88.8 | 100.5 | 90.1 |
| ID80 | 126.2 | 94.8 | 94.3 | 154.3 | 98.0 | 90.7 | 86.3 | 88.5 | 109.5 | 80.8 | 116.5 | 92.6 |
| ID800 | 190.5 | 233.4 | 282.6 | 74.4 | 66.4 | 89.3 | 86.7 | 77.8 | 75.7 | 80.9 | 67.8 | 68.6 |
| ID801 | 174.4 | 2203.4 | 385.9 | 5916.4 | 210.3 | 124.6 | 97.5 | 923.2 | 1714.2 | 4.4 | 123.4 | 39.0 |
| ID802 | 135.0 | 128.9 | 162.7 | 110.3 | 85.9 | 86.0 | 118.9 | 97.0 | 90.3 | 124.7 | 95.9 | 98.6 |
| ID803 | 64.7 | 79.4 | 136.0 | 111.1 | 67.8 | 135.5 | 67.1 | 109.0 | 106.9 | 50.4 | 51.1 | 59.3 |
| ID804 | 54.6 | 53.3 | 85.4 | 68.8 | 87.7 | 77.0 | 164.9 | 137.8 | 67.2 | 133.2 | 116.2 | 94.2 |
| ID805 | 72.9 | 115.4 | 208.3 | 107.7 | 28.6 | 86.9 | 87.2 | 119.2 | 76.9 | 58.4 | 44.1 | 58.0 |
| ID806 | 221.9 | 287.1 | 355.4 | 29.8 | 96.8 | 28.8 | 24.7 | 29.8 | 31.7 | 32.0 | 27.5 | 25.5 |
| ID807 | 105.8 | 128.2 | 201.5 | 82.6 | 55.6 | 122.9 | 113.0 | 128.6 | 73.0 | 110.9 | 81.5 | 114.5 |
| ID808 | 164.4 | 138.5 | 198.6 | 74.4 | 105.8 | 95.8 | 124.7 | 106.9 | 80.0 | 124.7 | 100.2 | 97.9 |
| ID809 | 119.6 | 97.8 | 119.6 | 118.1 | 144.1 | 125.3 | 64.3 | 121.1 | 118.2 | 60.0 | 50.7 | 56.1 |
| ID81 | 104.7 | 33.5 | 108.3 | 191.8 | 80.6 | 109.7 | 5.3 | 53.3 | 98.4 | 59.1 | 90.8 | 88.9 |
| ID810 | 112.2 | 49.6 | 146.3 | 107.1 | 131.1 | 125.8 | 89.6 | 94.6 | 102.2 | 84.9 | 82.8 | 86.3 |
| ID811 | 855.4 | 2711.6 | 15.6 | 34.0 | 27.4 | 9.8 | 38.7 | 1522.6 | 43.1 | 36.4 | 28.1 | 50.3 |
| ID813 | 198.5 | 87.2 | 88.3 | 32.5 | 194.1 | 38.1 | 28.4 | 100.7 | 84.1 | 81.4 | 70.8 | 39.9 |
| ID814 | 141.0 | 42.3 | 131.0 | 83.3 | 100.0 | 116.7 | 100.4 | 140.3 | 87.3 | 157.5 | 95.8 | 103.9 |
| ID815 | 157.0 | 69.5 | 64.0 | 117.1 | 60.8 | 89.2 | 129.9 | 106.7 | 123.5 | 158.0 | 99.9 | 104.2 |
| ID816 | 140.3 | 174.4 | 59.1 | 91.6 | 31.4 | 256.6 | 107.6 | 146.5 |  |  | 358.1 | 76.0 |
| ID819 | 149.5 | 693.1 | 7.1 | 19.6 | 85.3 | 11.4 | 24.8 | 1907.3 | 11.5 | 51.4 | 29.0 | 38.4 |
| ID82 | 53.3 | 74.1 | 98.8 | 107.0 | 103.5 | 31.4 | 89.3 | 124.7 | 50.4 | 268.9 | 152.6 | 149.9 |
| ID820 | 351.8 | 1271.5 | 10.7 | 57.9 | 60.4 | 8.5 | 27.8 | 50.0 | 35.4 | 32.3 | 23.8 | 45.2 |
| ID821 | 185.7 | 38.4 | 74.8 | 77.7 | 21.2 | 9.4 | 31.2 | 7.9 | 26.2 | 32.6 | 36.6 | 40.3 |
| ID822 | 189.1 | 179.1 | 208.2 | 412.4 | 32.9 | 69.6 | 902.3 | 235.3 | 338.8 | 828.6 | 74.2 | 57.0 |
| ID823 | 215.0 | 143.2 | 142.6 | 168.0 | 62.3 | 149.1 | 124.6 | 199.6 | 181.5 | 183.4 | 92.3 | 80.4 |
| ID824 | 149.3 | 106.7 | 130.9 | 134.6 | 59.8 | 59.1 | 397.1 | 45.8 | 78.2 | 207.7 | 150.5 | 105.1 |
| ID825 | 201.0 | 130.6 | 199.0 | 56.7 | 142.1 | 71.0 | 55.4 | 69.7 | 58.6 | 70.9 | 52.0 | 63.0 |
| ID826 | 135.9 | 152.0 | 205.2 | 60.1 | 69.6 | 107.7 | 120.9 | 133.4 | 92.0 | 83.0 | 97.7 | 68.4 |
| ID827 | 143.4 | 122.5 | 188.0 | 184.6 | 77.5 | 72.1 | 29.3 | 149.3 | 112.2 | 266.6 | 264.7 | 111.9 |
| ID828 | 217.1 | 226.0 | 5.6 | 14.9 | 29.7 | 18.9 | 61.9 | 876.4 | 3.7 | 86.3 | 12.2 | 49.0 |
| ID83 | 120.2 | 122.6 | 89.1 | 172.1 | 216.8 | 81.9 | 85.4 | 91.4 | 114.7 | 76.2 | 111.1 | 108.9 |
| ID830 | 284.4 | 811.9 | 13.9 | 34.1 | 40.3 | 9.4 | 27.4 | 965.4 | 30.5 | 32.8 | 27.3 | 44.7 |
| ID831 | 297.8 | 3913.7 | 23.2 | 46.2 | 44.0 | 10.2 | 6.9 | 3635.1 | 64.6 | 44.0 | 35.3 | 43.9 |
| ID832 | 358.3 | 1528.9 | 31.8 | 56.5 | 70.2 | 7.8 | 14.8 | 69.7 | 27.5 | 27.8 | 12.8 | 40.5 |
| ID833 | 228.5 | 37.0 | 75.7 | 115.4 | 36.6 | 12.0 | 118.0 | 0.4 | 28.6 | 62.2 | 41.5 | 44.2 |
| ID834 | 104.4 | 61.5 | 137.8 | 129.6 | 69.4 | 62.1 | 150.3 | 82.7 | 108.6 | 128.7 | 276.1 | 57.4 |
| ID835 | 235.1 | 121.1 | 158.8 | 81.1 | 244.7 | 87.0 | 71.6 | 73.2 | 62.8 | 58.4 | 53.6 | 59.7 |
| ID836 | 172.1 | 77.1 | 86.5 | 85.5 | 121.3 | 84.4 | 67.0 | 68.9 | 77.0 | 64.9 | 61.5 | 67.5 |
| ID837 | 166.5 | 71.8 | 196.0 | 242.6 | 22.0 | 24.6 | 58.8 | 690.9 | 106.6 | 137.9 | 1237.3 | 54.0 |
| ID838 | 144.1 | 77.6 | 65.6 | 312.6 | 16.9 | 23.8 | 29.7 | 15.8 | 200.2 | 75.3 | 487.4 | 148.6 |
| ID839 | 43.6 | 86.6 | 6.2 | 114.2 | 5.9 | 24.1 | 65.2 | 130.3 | 56.9 | 52.3 | 7.7 | 81.9 |
| ID84 | 59.7 | 117.1 | 131.7 | 128.8 | 92.7 | 95.0 | 86.6 | 91.8 | 121.7 | 99.5 | 112.0 | 108.8 |
| ID840 | 156.2 | 92.3 | 54.7 | 62.4 | 25.6 | 78.5 | 84.7 | 91.5 | 68.9 | 76.4 | 80.2 | 79.0 |
| ID85 | 160.8 | 287.4 | 187.1 | 107.2 | 138.8 | 202.4 | 86.8 | 229.4 | 246.3 | 306.7 | 370.4 | 127.5 |
| ID86 | 97.4 | 96.5 | 103.8 | 111.6 | 78.5 | 88.8 | 53.0 | 72.5 | 89.8 | 67.9 | 62.8 | 96.9 |
| ID87 | 98.4 | 51.4 | 109.4 | 211.9 | 81.1 | 102.4 | 13.9 | 67.0 | 85.6 | 72.4 | 77.0 | 91.7 |
| ID88 | 105.4 | 107.0 | 109.5 | 115.5 | 81.1 | 86.7 | 53.5 | 67.8 | 115.2 | 70.2 | 76.6 | 96.3 |
| ID89 | 58.3 | 156.4 | 165.5 | 160.2 | 114.3 | 77.1 | 14.9 | 183.8 | 63.4 | 182.7 | 81.6 | 155.7 |
| ID9 | 86.7 | 42.4 | 50.5 | 67.5 | 73.2 | 52.6 | 111.4 | 79.2 | 51.7 | 96.3 | 112.9 | 134.5 |
| ID90 | 170.0 | 96.1 | 75.7 | 127.2 | 232.0 | 96.1 | 81.4 | 136.0 | 108.4 | 34.6 | 108.2 | 99.2 |
| ID91 | 28.0 | 72.6 | 53.6 | 38.3 | 38.5 | 35.1 | 362.3 | 33.9 | 27.6 | 262.2 | 402.6 | 132.7 |
| ID93 | 20.9 | 50.9 | 52.2 | 76.9 | 46.8 | 37.4 | 101.7 | 41.4 | 49.9 | 274.3 | 251.6 | 356.7 |
| ID94 | 78.5 | 107.7 | 97.3 | 112.2 | 79.4 | 78.5 | 47.6 | 76.0 | 90.9 | 78.3 | 93.9 | 117.8 |
| ID95 | 41.6 | 13.6 | 11.2 | 134.8 | 45.7 | 35.4 | 63.5 | 33.9 | 84.7 | 294.6 | 265.8 | 218.7 |
| ID96 | 57.2 | 71.1 | 12.6 | 54.9 | 81.4 | 4.2 | 145.7 | 68.4 | 44.7 | 249.9 | 349.4 | 264.2 |
| ID97 | 11.7 | 43.9 | 62.9 | 86.1 | 87.2 | 57.8 | 83.2 | 54.1 | 69.6 | 153.9 | 179.1 | 215.7 |
| ID98 | 15.1 | 32.6 | 15.5 | 25.0 | 33.5 | 45.4 | 559.7 | 56.4 | 50.8 | 213.1 | 262.3 | 101.5 |
| ID99 | 29.2 | 29.7 | 32.0 | 23.0 | 29.6 | 30.6 | 331.7 | 35.7 | 40.7 | 200.5 | 244.7 | 113.4 |

^a^ Due to limited space, only age-associated spot ID’s (spots highlighted in yellow) are shown on the 2D-DIGE image presented in Figure 1 . Should you require information on the location on 2-D-DIGE Image of other spot ID’s in this table, please contact the corresponding author

**Reference**

[1]	Sikulu MT, Monkman J, Dave KA, Hastie ML, Dale PE, Kitching RL, Killeen GF, Kay BH, Gorman JJ, Hugo L**E**. Proteomic changes occurring in the malaria mosquitoes Anopheles gambiae and Anopheles stephensi during aging. Journal of Proteomi*c*s 2015; 126:234-44.
