# Supplementary material for: Mass spectrometry identification of age-associated proteins from the malaria mosquitoes Anopheles gambiae s.s. and Anopheles stephensi
Source: Data Brief. 2015 Jul 16;4:461–7. doi: 10.1016/j.dib.2015.07.007 (PMC4534588; doi:10.1016/j.dib.2015.07.007)
Supplement: Supplementary file 1 — Supplementary material: Supplementary Table 1: Normalised spot volume data for all spots detected on 2D-DIGE images of cohort 1 of A. gambiae. Spots highlighted in yellow were found to be age-associated. Supplementary Table 2: Normalised spot volume data for all spots detected on 2D-DIGE images of cohort 2 of A. gambiae. Spots highlighted in yellow were found to be age-associated. Supplementary Table 3: Normalised spot volume data for all spots detected on 2D-DIGE images of cohort 1 of A. stephensi. Spots highlighted in yellow were found to be age-associated. Supplementary Table 4: Normalised spot volume data for all spots detected on 2D-DIGE images of cohort 2 of A. stephensi. Spots highlighted in yellow were found to be age-associated. External repository data: The mass spectrometry proteomics data have been deposited to the ProteomeXchange Consortium [8] via the PRIDE partner repository with the dataset identifier PXD002153 and 10.6019/PXD002153. [file mmc1.zip › Supplimentary file/Supplementary Table 4.doc]

Table 4

| **Spot ID^a^** | **Normalized Vol. D41b** | **Normalized Vol. D1a** | **Normalized Vol. D1c** | **Normalized Vol. D9a** | **Normalized Vol. D9c** | **Normalized Vol. D9b** | **Normalized Vol. D17b** | **Normalized Vol. D17c** | **Normalized Vol. D17c** | **Normalized Vol. D34c** | **Normalized Vol. D34b** | **Normalized Vol. D34a** |
| --- | --- | --- | --- | --- | --- | --- | --- | --- | --- | --- | --- | --- |
| ID1000 | 132.3 | 134.1 | 148.9 | 97.3 | 93.1 | 112.8 | 109.6 | 102.2 | 103.3 | 92.7 | 81.9 | 72.0 |
| ID1001 | 404.3 | 180.3 | 308.1 | 24.6 | 48.7 | 93.4 | 93.6 | 27.0 | 10.5 | 69.8 | 43.3 | 40.2 |
| ID100128 | 111.1 | 88.6 | 104.7 | 118.5 | 121.3 | 125.3 | 106.8 | 88.6 | 96.9 | 122.5 | 121.0 | 126.2 |
| ID100415 | 80.1 | 69.8 | 86.4 | 93.4 | 121.8 | 108.4 | 122.3 | 107.5 | 105.9 | 141.8 | 131.7 | 121.5 |
| ID1005 | 141.6 | 121.6 | 170.3 | 67.8 | 82.3 | 3.6 | 6.5 | 62.1 | 88.7 | 61.7 | 56.2 | 72.1 |
| ID1007 | 121.9 | 80.6 | 378.5 | 27.5 | 39.6 | 9.0 | 20.6 | 50.9 | 87.9 | 66.3 | 54.5 | 40.4 |
| ID1008 | 31.7 | 71.6 | 3839.8 | 8.7 | 21.2 | 153.1 | 305.9 | 54.4 | 313.0 | 70.8 | 39.7 | 9.5 |
| ID101521 | 63.1 | 48.6 | 52.5 | 83.7 | 102.6 | 83.1 | 110.9 | 110.3 | 121.0 | 168.8 | 124.8 | 108.5 |
| ID101789 | 97.5 | 83.9 | 110.1 | 74.9 | 85.5 | 93.7 | 91.8 | 90.8 | 91.7 | 98.9 | 113.7 | 114.4 |
| ID104078 | 103.6 | 99.8 | 93.8 | 96.1 | 82.3 | 104.0 | 93.6 | 82.1 | 86.2 | 78.4 | 85.3 | 94.9 |
| ID106643 | 169.5 | 265.6 | 161.7 | 80.0 | 87.8 | 156.6 | 128.0 | 66.8 | 95.3 | 84.1 | 94.0 | 47.7 |
| ID107354 | 122.1 | 101.4 | 51.0 | 142.8 | 157.2 | 145.4 | 120.4 | 139.0 | 147.6 | 187.9 | 139.8 | 135.2 |
| ID108643 | 109.4 | 110.1 | 165.6 | 98.6 | 131.9 | 105.0 | 98.4 | 109.8 | 115.1 | 133.8 | 99.7 | 115.1 |
| ID109371 | 108.3 | 39.6 | 73.4 | 69.5 | 85.5 | 75.7 | 66.7 | 93.9 | 93.0 | 112.7 | 80.9 | 85.8 |
| ID109667 | 117.7 | 136.8 | 133.1 | 108.4 | 107.5 | 118.2 | 112.0 | 98.6 | 96.2 | 106.2 | 100.1 | 95.0 |
| ID110393 | 124.9 | 144.5 | 125.5 | 100.1 | 108.4 | 119.1 | 114.0 | 105.9 | 101.2 | 99.9 | 107.9 | 97.2 |
| ID111106 | 109.0 | 369.9 | 214.3 | 110.8 | 104.7 | 113.9 | 112.5 | 98.7 | 94.6 | 76.2 | 46.9 | 77.0 |
| ID111847 | 62.2 | 98.5 | 92.9 | 147.6 | 122.3 | 128.2 | 160.6 | 126.0 | 144.4 | 158.1 | 130.9 | 121.2 |
| ID112519 | 131.1 | 148.8 | 125.0 | 105.3 | 76.4 | 102.1 | 92.5 | 98.5 | 103.2 | 84.9 | 75.2 | 99.7 |
| ID113102 | 117.3 | 115.0 | 115.4 | 104.0 | 96.0 | 106.7 | 96.0 | 105.7 | 102.1 | 97.6 | 102.5 | 88.9 |
| ID1158 | 104.0 | 122.3 | 114.8 | 101.7 | 108.7 | 114.6 | 157.5 | 117.5 | 121.9 | 99.3 | 135.6 | 132.7 |
| ID116748 | 49.2 | 51.8 | 58.4 | 71.0 | 69.2 | 114.9 | 109.0 | 97.6 | 98.3 | 155.4 | 158.5 | 116.8 |
| ID116780 | 127.2 | 137.1 | 135.5 | 106.3 | 62.8 | 94.5 | 95.5 | 118.9 | 113.6 | 296.4 | 136.3 | 112.7 |
| ID117517 | 283.3 | 63.0 | 74.5 | 169.9 | 139.7 | 116.6 | 59.2 | 128.1 | 175.1 | 86.2 | 394.6 | 157.2 |
| ID118270 | 311.6 | 78.9 | 72.3 | 89.2 | 121.4 | 224.7 | 139.8 | 177.1 | 131.4 | 89.2 | 361.6 | 225.1 |
| ID1186 | 81.9 | 77.9 | 71.5 | 73.5 | 72.2 | 72.0 | 76.0 | 73.1 | 69.9 | 71.9 | 85.2 | 77.2 |
| ID11874 | 105.9 | 124.1 | 126.8 | 97.0 | 84.0 | 104.4 | 84.4 | 100.2 | 100.4 | 86.9 | 81.7 | 75.3 |
| ID119049 | 85.6 | 72.0 | 62.5 | 92.5 | 81.0 | 93.2 | 78.6 | 88.9 | 100.9 | 108.7 | 98.8 | 83.9 |
| ID129544 | 211.5 | 136.8 | 158.7 | 205.4 | 210.1 | 152.5 | 227.1 | 154.7 | 268.6 | 175.3 | 193.3 | 239.7 |
| ID13072 | 149.9 | 140.8 | 195.7 | 48.2 | 69.6 | 74.8 | 138.4 | 51.3 | 53.3 | 74.3 | 118.3 | 179.0 |
| ID13127 | 136.6 | 101.5 | 151.8 | 95.2 | 113.1 | 111.7 | 119.7 | 89.1 | 108.2 | 103.0 | 100.0 | 91.0 |
| ID13298 | 132.3 | 127.1 | 117.6 | 146.2 | 146.0 | 113.7 | 127.4 | 114.1 | 132.1 | 118.2 | 101.5 | 138.2 |
| ID134685 | 92.4 | 95.5 | 101.6 | 115.1 | 108.3 | 116.9 | 114.4 | 105.9 | 113.5 | 92.5 | 92.7 | 104.1 |
| ID13681 | 77.4 | 102.0 | 83.3 | 93.7 | 87.5 | 87.4 | 74.0 | 73.9 | 69.2 | 92.2 | 81.3 | 76.8 |
| ID140272 | 90.7 | 78.7 | 87.4 | 84.8 | 87.0 | 100.0 | 103.6 | 94.5 | 98.2 | 91.3 | 103.9 | 110.2 |
| ID140941 | 118.3 | 88.5 | 85.3 | 62.2 | 65.3 | 59.3 | 58.9 | 51.7 | 35.4 | 64.1 | 52.4 | 68.1 |
| ID142504 | 103.2 | 101.2 | 102.7 | 113.8 | 107.4 | 114.0 | 97.6 | 106.2 | 101.0 | 104.7 | 94.9 | 89.8 |
| ID143955 | 105.3 | 73.9 | 80.1 | 84.6 | 85.8 | 67.3 | 66.3 | 83.5 | 88.0 | 83.6 | 67.3 | 86.1 |
| ID145524 | 85.7 | 65.8 | 64.7 | 110.0 | 98.0 | 91.8 | 92.9 | 119.5 | 109.3 | 114.7 | 92.9 | 105.9 |
| ID146831 | 79.1 | 59.2 | 36.4 | 99.6 | 91.8 | 87.7 | 83.6 | 97.7 | 87.7 | 107.3 | 84.0 | 98.5 |
| ID147908 | 127.6 | 138.3 | 127.6 | 107.2 | 99.7 | 106.9 | 106.0 | 102.3 | 98.1 | 89.1 | 92.2 | 91.9 |
| ID148523 | 110.4 | 107.6 | 93.7 | 117.6 | 113.7 | 159.1 | 98.5 | 110.9 | 96.3 | 117.4 | 122.1 | 71.9 |
| ID148538 | 75.4 | 45.4 | 56.9 | 93.6 | 108.5 | 123.3 | 132.2 | 102.8 | 101.2 | 118.7 | 103.5 | 104.4 |
| ID14873 | 115.2 | 104.7 | 109.2 | 105.9 | 106.6 | 112.9 | 111.0 | 89.1 | 98.6 | 89.6 | 102.6 | 97.2 |
| ID149558 | 89.2 | 81.8 | 85.5 | 80.0 | 88.6 | 102.3 | 112.3 | 93.2 | 92.2 | 123.1 | 119.8 | 120.9 |
| ID150002 | 119.8 | 105.2 | 92.4 | 84.8 | 120.8 | 117.8 | 69.4 | 65.9 | 63.0 | 79.7 | 89.9 | 98.3 |
| ID150099 | 90.6 | 93.2 | 92.4 | 93.8 | 88.7 | 85.6 | 83.4 | 98.6 | 91.9 | 102.2 | 89.5 | 76.3 |
| ID150134 | 84.5 | 74.8 | 68.4 | 94.4 | 104.6 | 98.3 | 108.8 | 126.6 | 122.7 | 162.7 | 116.6 | 112.3 |
| ID15082 | 91.1 | 90.8 | 112.4 | 123.5 | 117.1 | 124.2 | 123.6 | 124.2 | 115.7 | 102.8 | 97.0 | 97.5 |
| ID151606 | 120.8 | 75.7 | 115.2 | 97.0 | 91.0 | 45.1 | 53.5 | 158.3 | 99.8 | 118.0 | 181.5 | 114.7 |
| ID15293 | 93.4 | 56.9 | 79.5 | 79.6 | 87.5 | 130.5 | 105.9 | 95.5 | 106.3 | 114.0 | 163.0 | 147.7 |
| ID154944 | 325.3 | 252.4 | 303.7 | 143.1 | 103.5 | 154.5 | 137.9 | 95.7 | 126.7 | 96.1 | 68.0 | 111.4 |
| ID1554 | 251.3 | 100.8 | 279.8 | 95.0 | 151.6 | 55.8 | 72.4 | 221.4 | 253.9 | 125.7 | 141.5 | 285.9 |
| ID1566 | 188.8 | 68.7 | 249.0 | 219.7 | 183.6 | 61.9 | 68.5 | 153.7 | 327.3 | 139.9 | 102.5 | 243.2 |
| ID1579 | 72.4 | 63.5 | 60.8 | 81.9 | 82.2 | 75.7 | 102.0 | 65.6 | 87.3 | 71.9 | 88.0 | 101.7 |
| ID161825 | 47.7 | 35.1 | 39.6 | 50.4 | 64.5 | 71.9 | 94.7 | 85.4 | 79.2 | 104.8 | 115.1 | 123.5 |
| ID164057 | 82.8 |  | 111.8 |  | 129.6 | 108.8 | 157.8 |  | 150.1 | 146.1 | 102.5 | 457.2 |
| ID165744 | 36.0 | 74.4 | 80.4 | 68.7 | 65.8 | 79.4 | 64.2 | 79.7 | 73.8 | 79.8 | 72.1 | 63.6 |
| ID1670 | 88.6 | 90.3 | 93.7 | 121.9 | 118.4 | 121.1 | 129.3 | 104.2 | 115.8 | 102.5 | 103.4 | 101.6 |
| ID17595 | 116.0 | 110.8 | 107.9 | 73.1 | 69.9 | 77.6 | 98.7 | 78.6 | 81.0 | 74.0 | 86.9 | 90.5 |
| ID17841 | 57.5 | 54.0 | 50.5 | 74.8 | 74.4 | 76.2 | 95.3 | 84.3 | 90.2 | 106.7 | 109.2 | 106.7 |
| ID1799 | 112.5 | 102.9 | 94.7 | 106.3 | 112.5 | 124.4 | 117.2 | 110.6 | 99.3 | 93.7 | 97.7 | 95.5 |
| ID1808 | 81.4 | 77.3 | 75.5 | 100.5 | 97.3 | 110.3 | 102.4 | 99.2 | 102.0 | 97.0 | 99.5 | 101.6 |
| ID1823 | 110.9 | 103.9 | 103.1 | 103.2 | 95.6 | 111.4 | 99.5 | 77.2 | 82.3 | 48.6 | 47.7 | 48.4 |
| ID18244 | 64.5 | 66.9 | 64.6 | 96.0 | 84.8 | 98.0 | 113.2 | 98.6 | 105.4 | 118.0 | 129.0 | 117.1 |
| ID18296 | 77.3 | 91.5 | 99.8 | 108.2 | 129.1 | 157.4 | 182.9 | 157.3 | 118.6 | 134.4 | 183.9 | 128.7 |
| ID18350 | 316.7 | 388.7 | 307.6 | 87.2 | 74.5 | 62.5 | 102.6 | 67.2 | 64.5 | 38.3 | 52.9 | 50.5 |
| ID18956 | 97.1 | 128.0 | 99.1 | 84.1 | 86.5 | 101.2 | 77.4 | 92.3 | 80.4 | 136.2 | 89.2 | 87.1 |
| ID1908 | 102.2 | 82.8 | 105.6 | 65.5 | 54.6 | 102.5 | 103.6 | 95.2 | 89.2 | 113.6 | 157.3 | 102.7 |
| ID19246 | 101.1 | 94.0 | 80.2 | 111.2 | 45.2 | 196.2 | 156.1 | 69.5 | 96.3 | 66.4 | 94.9 | 153.4 |
| ID20094 | 95.4 | 82.6 | 79.0 | 102.7 | 86.4 | 120.9 | 147.3 | 134.2 | 120.3 | 112.9 | 103.8 | 86.7 |
| ID20380 | 106.9 | 118.8 | 139.3 | 210.6 | 155.6 | 153.9 | 149.0 | 241.5 | 156.7 | 302.6 | 121.9 | 103.3 |
| ID20652 | 108.5 | 106.6 | 129.3 | 106.3 | 79.6 | 83.9 | 115.9 | 121.3 | 112.4 | 108.5 | 138.3 | 147.5 |
| ID20671 | 352.9 | 253.3 | 252.7 | 120.1 | 87.8 | 60.0 | 60.5 | 32.2 | 98.0 | 87.5 | 70.4 | 90.5 |
| ID21258 | 114.6 | 138.4 | 110.2 | 154.8 | 126.1 | 133.1 | 120.8 | 106.1 | 110.4 | 102.5 | 110.4 | 123.8 |
| ID21275 | 102.2 | 78.9 | 87.1 | 74.1 | 73.6 | 77.0 | 84.1 | 64.4 | 61.6 | 64.2 | 87.1 | 66.1 |
| ID23136 | 123.1 | 121.2 | 108.8 | 116.9 | 94.4 | 121.8 | 89.3 | 91.1 | 98.7 | 96.3 | 97.6 | 96.1 |
| ID23464 | 106.0 | 108.6 | 108.2 | 106.7 | 105.4 | 112.7 | 107.4 | 111.2 | 104.4 | 107.8 | 110.5 | 105.6 |
| ID23484 | 140.0 | 131.8 | 135.9 | 71.7 | 72.8 | 75.7 | 67.7 | 81.1 | 64.4 | 80.9 | 64.1 | 71.8 |
| ID24494 | 73.3 | 84.5 | 87.1 | 78.9 | 81.9 | 91.8 | 79.4 | 88.0 | 79.2 | 86.8 | 81.4 | 83.6 |
| ID254 | 120.5 | 94.2 | 205.3 | 78.2 | 86.2 | 122.6 | 112.6 | 113.1 | 58.1 | 83.2 | 116.0 | 117.7 |
| ID2605 | 218.4 | 226.9 | 204.0 | 92.4 | 119.7 | 131.4 | 123.2 | 61.7 | 83.4 | 64.2 | 74.5 | 63.1 |
| ID262 | 128.3 | 102.7 | 116.9 | 99.2 | 96.2 | 70.6 | 86.7 | 142.5 | 121.2 | 122.1 | 109.1 | 64.6 |
| ID26664 | 110.6 | 102.1 | 105.4 | 121.0 | 110.2 | 114.3 | 99.1 | 88.9 | 110.1 | 94.5 | 104.1 | 112.9 |
| ID268 | 116.3 | 204.4 | 1740.6 | 86.9 | 99.7 | 153.2 | 75.5 |  | 57.9 | 93.7 | 44.5 |  |
| ID271 | 42.1 | 75.0 | 86.4 | 65.7 | 175.9 | 113.2 | 94.0 | 88.2 | 118.6 | 509.6 | 97.8 | 130.6 |
| ID272 | 86.4 | 29.2 | 124.6 | 41.9 | 161.5 | 150.3 | 106.1 | 120.3 | 148.4 | 162.9 | 71.0 | 90.6 |
| ID27220 | 100.2 | 96.9 | 106.2 | 124.0 | 89.1 | 104.1 | 84.7 | 98.4 | 109.6 | 73.5 | 80.6 | 99.1 |
| ID275 | 57.7 | 67.7 | 58.9 | 101.6 | 103.2 | 81.6 | 132.6 | 133.5 | 117.5 | 136.6 | 104.3 | 86.7 |
| ID276 | 68.4 | 84.8 | 73.8 | 128.1 | 120.1 | 108.0 | 132.2 | 164.2 | 137.4 | 152.4 | 108.6 | 90.8 |
| ID280 | 53.6 | 69.3 | 60.9 | 64.7 | 86.6 | 47.9 | 113.9 | 82.8 | 78.3 | 114.6 | 103.5 | 50.0 |
| ID281 | 73.7 | 87.0 | 79.9 | 79.4 | 104.6 | 71.8 | 111.2 | 111.0 | 92.9 | 136.5 | 106.6 | 83.4 |
| ID282 | 88.3 | 102.5 | 98.0 | 93.6 | 116.8 | 84.3 | 133.7 | 121.7 | 98.5 | 157.4 | 119.0 | 94.9 |
| ID284 | 72.6 | 78.2 | 63.0 | 79.3 | 110.8 | 89.7 | 149.3 | 190.3 | 100.3 | 153.5 | 114.8 | 126.3 |
| ID288 | 44.5 | 54.0 | 36.9 | 80.3 | 90.0 | 72.6 | 137.0 | 130.8 | 112.3 | 130.8 | 96.6 | 87.5 |
| ID289 | 110.1 | 78.6 | 85.8 | 83.2 | 78.4 | 120.1 | 118.6 | 80.8 | 79.8 | 88.5 | 139.7 | 87.0 |
| ID292 | 43.5 | 81.1 | 66.5 | 94.5 | 130.6 | 100.5 | 81.7 | 123.1 | 113.4 | 171.0 | 102.9 | 108.4 |
| ID300 | 109.6 | 116.0 | 104.6 | 110.5 | 100.0 | 152.1 | 103.6 | 96.2 | 106.4 | 112.5 | 110.7 | 78.2 |
| ID303 | 123.4 | 98.5 | 86.6 | 98.9 | 74.5 | 177.2 | 192.3 | 79.6 | 98.5 | 84.2 | 128.6 | 78.6 |
| ID304 | 114.1 | 168.8 | 89.7 | 85.0 | 149.1 | 111.1 | 80.6 | 136.0 | 135.8 | 88.2 | 122.3 | 167.6 |
| ID307 | 90.5 | 79.4 | 50.3 | 73.5 | 63.7 | 56.4 | 81.7 | 54.9 | 416.9 | 24.6 | 95.7 | 61.3 |
| ID308 | 81.6 | 118.9 | 117.0 | 91.2 | 73.2 | 131.6 | 117.9 | 115.5 | 91.9 | 104.3 | 220.4 | 107.4 |
| ID312 | 117.9 | 140.7 | 147.6 | 114.4 | 96.4 | 117.4 | 100.8 | 106.9 | 111.2 | 101.9 | 90.6 | 57.2 |
| ID315 | 75.0 | 105.1 | 89.4 | 138.6 | 140.8 | 84.0 | 87.1 | 97.4 | 98.0 | 104.5 | 87.6 | 81.7 |
| ID317 | 61.2 | 72.9 | 76.2 | 98.9 | 97.1 | 116.3 | 89.1 | 82.3 | 87.6 | 94.4 | 94.3 | 80.2 |
| ID318 | 106.2 | 116.4 | 122.1 | 92.7 | 77.6 | 102.2 | 79.3 | 110.2 | 100.2 | 78.9 | 84.3 | 67.6 |
| ID319 | 102.5 | 116.9 | 131.3 | 107.9 | 99.8 | 151.7 | 81.4 | 198.2 | 107.6 | 102.3 | 95.4 | 55.2 |
| ID320 | 89.1 | 94.3 | 95.3 | 103.4 | 101.2 | 99.8 | 104.0 | 159.8 | 97.0 | 104.1 | 97.1 | 62.6 |
| ID323 | 84.8 | 97.6 | 98.0 | 87.5 | 94.5 | 97.1 | 90.2 | 94.2 | 93.5 | 94.2 | 76.5 | 83.1 |
| ID325 | 134.2 | 154.3 | 223.7 | 151.4 | 89.7 | 107.4 | 94.6 | 120.1 | 108.0 | 131.0 | 135.1 | 112.7 |
| ID326 | 112.2 | 118.9 | 102.9 | 101.3 | 94.5 | 98.8 | 86.8 | 98.1 | 92.1 | 88.5 | 99.2 | 69.0 |
| ID327 | 92.6 | 92.0 | 90.5 | 111.5 | 105.4 | 119.9 | 114.7 | 118.7 | 102.7 | 104.8 | 100.0 | 88.1 |
| ID331 | 133.2 | 118.5 | 93.8 | 104.2 | 87.0 | 134.4 | 178.3 | 108.4 | 100.2 | 85.3 | 110.1 | 110.8 |
| ID333 | 102.7 | 108.5 | 107.0 | 103.7 | 98.1 | 104.4 | 100.9 | 109.6 | 96.8 | 100.6 | 95.9 | 79.9 |
| ID334 | 76.3 | 86.1 | 85.6 | 118.5 | 111.0 | 115.8 | 92.5 | 129.5 | 101.0 | 108.0 | 93.1 | 73.1 |
| ID336 | 124.6 | 127.1 | 115.3 | 107.0 | 97.5 | 101.3 | 84.8 | 102.0 | 90.1 | 88.8 | 85.0 | 72.7 |
| ID339 | 88.5 | 39.9 | 51.0 | 47.4 | 74.3 | 113.6 | 93.1 | 94.6 | 102.9 | 94.8 | 158.5 | 100.3 |
| ID341 | 79.1 | 156.2 | 89.9 | 248.1 | 132.1 | 2106.4 | 318.1 | 147.3 | 126.5 | 131.4 | 84.3 | 49.9 |
| ID344 | 101.5 | 256.7 | 155.2 | 200.4 | 102.9 | 88.0 | 79.3 | 164.6 | 110.0 | 99.9 | 93.6 | 14.5 |
| ID346 | 106.4 | 128.5 | 114.7 | 98.7 | 69.6 | 100.1 | 78.8 | 94.6 | 90.7 | 79.2 | 75.7 | 73.2 |
| ID347 | 219.9 | 109.9 | 121.2 | 95.3 | 58.8 | 86.3 | 61.3 | 118.7 | 95.6 | 92.0 | 151.6 | 82.5 |
| ID349 | 85.0 | 109.1 | 92.8 | 101.1 | 67.8 | 134.0 | 189.2 | 96.5 | 93.8 | 87.6 | 95.9 | 96.7 |
| ID351 | 271.6 | 134.9 | 142.5 | 73.1 | 84.8 | 95.9 | 77.5 | 55.3 | 78.7 | 63.6 | 102.2 | 97.1 |
| ID352 | 96.6 | 100.5 | 102.5 | 72.8 | 84.2 | 66.6 | 75.1 | 95.5 | 89.3 | 83.5 | 79.3 | 60.8 |
| ID354 | 123.7 | 104.6 | 107.0 | 103.5 | 118.3 | 81.1 | 75.5 | 172.9 | 92.4 | 187.2 | 145.6 | 149.0 |
| ID355 | 86.2 | 77.9 | 64.4 | 63.2 | 74.4 | 80.7 | 60.2 | 88.1 | 71.1 | 70.7 | 68.9 | 36.5 |
| ID356 | 104.9 | 102.2 | 109.7 | 85.3 | 94.2 | 80.7 | 91.1 | 121.7 | 100.8 | 96.0 | 81.9 | 71.7 |
| ID357 | 99.2 | 102.8 | 104.7 | 103.7 | 93.8 | 103.3 | 94.2 | 108.4 | 87.7 | 92.9 | 86.6 | 59.0 |
| ID358 | 96.5 | 111.1 | 89.2 | 132.5 | 118.0 | 128.4 | 94.5 | 182.5 | 78.6 | 114.8 | 89.5 | 27.9 |
| ID359 | 111.3 | 110.7 | 105.4 | 100.4 | 100.9 | 103.4 | 94.5 | 103.8 | 90.1 | 106.5 | 88.3 | 83.8 |
| ID360 | 99.2 | 109.6 | 93.6 | 115.8 | 110.1 | 123.3 | 95.4 | 111.4 | 81.6 | 109.1 | 96.0 | 74.1 |
| ID361 | 91.7 | 117.1 | 95.9 | 87.5 | 103.1 | 97.5 | 118.2 | 103.5 | 104.4 | 87.2 | 118.3 | 112.4 |
| ID362 | 97.7 | 107.9 | 106.8 | 91.2 | 103.5 | 97.9 | 90.0 | 102.7 | 88.5 | 203.5 | 97.6 | 89.3 |
| ID363 | 95.5 | 111.6 | 105.5 | 108.7 | 112.2 | 102.9 | 87.8 | 111.6 | 102.9 | 103.3 | 83.7 | 78.7 |
| ID364 | 96.9 | 107.8 | 99.4 | 113.0 | 110.5 | 105.9 | 88.1 | 104.0 | 94.4 | 103.2 | 87.2 | 74.4 |
| ID366 | 246.8 | 113.4 | 51.7 | 116.3 | 100.0 | 141.4 | 104.0 | 115.4 | 108.7 | 105.5 | 388.1 | 88.1 |
| ID367 | 93.9 | 112.3 | 114.9 | 97.8 | 107.5 | 98.1 | 93.0 | 103.2 | 92.5 | 169.8 | 99.6 | 80.9 |
| ID368 | 106.6 | 114.1 | 107.8 | 98.5 | 99.6 | 105.2 | 101.9 | 107.4 | 92.9 | 125.3 | 97.5 | 81.6 |
| ID369 | 112.8 | 112.1 | 108.5 | 100.2 | 103.5 | 104.2 | 96.8 | 109.4 | 92.4 | 130.8 | 90.4 | 84.9 |
| ID370 | 110.6 | 98.7 | 85.2 | 117.3 | 100.1 | 134.9 | 93.5 | 141.9 | 104.0 | 95.3 | 124.6 | 103.0 |
| ID371 | 89.1 | 99.3 | 80.0 | 119.9 | 114.9 | 113.2 | 118.0 | 92.5 | 116.1 | 106.9 | 98.8 | 78.2 |
| ID372 | 152.9 | 50.2 | 56.1 | 38.2 | 96.2 | 142.9 | 98.9 | 91.6 | 138.1 | 95.5 | 251.0 | 117.8 |
| ID373 | 105.5 | 111.6 | 109.4 | 102.1 | 106.5 | 103.4 | 93.3 | 105.9 | 96.2 | 126.2 | 92.1 | 86.4 |
| ID374 | 86.5 | 112.2 | 93.1 | 130.3 | 120.0 | 106.7 | 104.7 | 124.2 | 93.7 | 125.0 | 102.7 | 98.9 |
| ID375 | 87.9 | 98.3 | 88.8 | 133.8 | 108.7 | 122.3 | 56.4 | 92.7 | 96.2 | 107.5 | 87.1 | 81.6 |
| ID376 | 212.6 | 54.6 | 49.4 | 32.7 | 91.0 | 138.0 | 106.8 | 41.3 | 126.0 | 81.0 | 253.0 | 171.8 |
| ID378 | 182.5 | 135.7 | 184.5 | 94.2 | 66.9 | 64.9 | 69.6 | 61.5 | 71.9 | 75.7 | 63.5 | 53.7 |
| ID383 | 72.3 | 82.7 | 65.4 | 94.9 | 83.3 | 94.6 | 103.6 | 76.3 | 93.7 | 79.1 | 80.7 | 73.9 |
| ID384 | 83.1 | 115.8 | 79.8 | 123.2 | 97.0 | 121.3 | 121.5 | 105.5 | 110.0 | 107.6 | 96.7 | 96.7 |
| ID38550 | 166.8 | 132.5 | 111.2 | 77.6 | 65.9 | 80.2 | 70.9 | 68.8 | 69.6 | 54.9 | 34.8 | 73.4 |
| ID386 | 103.8 | 104.9 | 97.1 | 94.5 | 96.6 | 99.2 | 106.4 | 92.0 | 96.2 | 95.6 | 103.3 | 100.2 |
| ID387 | 210.3 | 208.8 | 215.1 | 76.3 | 74.2 | 77.3 | 84.3 | 72.2 | 74.9 | 76.4 | 79.4 | 52.3 |
| ID388 | 268.9 | 146.3 | 152.5 | 44.3 | 64.9 | 82.7 | 73.0 | 47.4 | 74.1 | 61.9 | 95.9 | 58.2 |
| ID389 | 93.2 | 133.8 | 120.8 | 90.8 | 91.2 | 87.5 | 98.7 | 99.9 | 88.7 | 129.8 | 106.0 | 84.5 |
| ID391 | 83.7 | 88.1 | 82.5 | 96.2 | 96.1 | 104.5 | 111.5 | 96.9 | 104.7 | 127.7 | 97.2 | 107.1 |
| ID39171 | 0.9 | 5.2 | 3.2 | 68.7 | 94.7 | 92.0 | 178.3 | 120.7 | 116.1 | 113.5 | 124.8 | 246.2 |
| ID39241 | 133.7 | 96.8 | 86.6 | 93.9 | 96.3 | 106.4 | 108.1 | 80.0 | 104.1 | 113.6 | 110.5 | 115.0 |
| ID393 | 43.8 | 92.6 | 65.4 | 73.8 | 83.0 | 77.6 | 121.8 | 112.7 | 106.6 | 209.1 | 131.2 | 122.1 |
| ID394 | 35.6 | 45.3 | 40.2 | 74.6 | 80.7 | 75.4 | 136.6 | 118.9 | 127.8 | 186.6 | 129.6 | 119.4 |
| ID395 | 53.5 | 50.3 | 49.9 | 85.5 | 95.7 | 87.7 | 146.6 | 122.2 | 126.4 | 187.8 | 124.6 | 118.3 |
| ID396 | 67.3 | 59.3 | 60.7 | 103.2 | 110.5 | 106.0 | 149.0 | 134.0 | 133.0 | 184.9 | 123.5 | 119.0 |
| ID397 | 102.6 | 83.2 | 80.9 | 95.8 | 92.3 | 113.0 | 108.8 | 101.5 | 102.9 | 99.1 | 112.2 | 97.9 |
| ID398 | 88.4 | 85.5 | 73.8 | 101.6 | 108.2 | 102.3 | 96.8 | 101.3 | 97.3 | 120.8 | 93.7 | 89.3 |
| ID39866 | 85.7 | 67.5 | 69.7 | 111.3 | 156.6 | 137.4 | 139.2 | 190.6 | 108.0 | 35.4 | 58.4 | 157.3 |
| ID39937 | 92.9 | 78.4 | 70.1 | 95.1 | 100.6 | 101.1 | 98.8 | 103.1 | 94.6 | 107.7 | 96.8 | 108.2 |
| ID400 | 84.5 | 80.7 | 73.9 | 102.0 | 118.9 | 110.7 | 106.8 | 100.9 | 99.1 | 120.3 | 104.5 | 93.3 |
| ID402 | 59.9 | 70.6 | 50.6 | 73.8 | 72.6 | 77.5 | 90.6 | 57.9 | 81.7 | 70.2 | 71.4 | 65.1 |
| ID40290 | 220.2 | 160.8 | 167.1 | 70.9 | 93.7 | 85.6 | 60.6 | 73.8 | 61.4 | 35.9 | 97.8 | 37.7 |
| ID403 | 78.8 | 55.4 | 64.4 | 84.1 | 106.6 | 114.3 | 105.6 | 104.0 | 92.5 | 118.0 | 99.0 | 93.0 |
| ID404 | 86.2 | 60.1 | 60.0 | 71.9 | 83.6 | 101.0 | 102.4 | 84.8 | 87.8 | 89.7 | 104.7 | 90.6 |
| ID405 | 93.4 | 68.7 | 69.7 | 81.7 | 88.3 | 99.6 | 96.9 | 90.7 | 88.5 | 85.7 | 93.1 | 85.4 |
| ID406 | 88.3 | 70.0 | 79.0 | 81.3 | 83.9 | 100.3 | 98.5 | 96.3 | 92.4 | 89.2 | 98.8 | 86.5 |
| ID407 | 92.8 | 84.3 | 84.5 | 103.0 | 95.2 | 103.0 | 95.2 | 99.3 | 92.5 | 102.3 | 98.6 | 95.6 |
| ID408 | 111.9 | 74.1 | 67.0 | 76.8 | 86.0 | 105.9 | 97.5 | 88.6 | 88.8 | 96.9 | 136.3 | 91.9 |
| ID409 | 72.0 | 68.0 | 63.6 | 73.8 | 80.8 | 84.8 | 90.8 | 59.0 | 91.9 | 93.9 | 87.7 | 122.5 |
| ID40928 | 112.2 | 97.5 | 99.3 | 89.0 | 112.1 | 109.2 | 80.8 | 118.2 | 71.0 | 90.8 | 91.8 | 103.3 |
| ID410 | 75.2 | 65.0 | 71.0 | 74.0 | 78.4 | 87.7 | 92.5 | 79.5 | 69.0 | 88.1 | 80.3 | 74.8 |
| ID411 | 77.6 | 65.2 | 63.0 | 77.5 | 87.5 | 67.5 | 78.1 | 85.6 | 83.8 | 92.7 | 90.4 | 81.3 |
| ID412 | 71.7 | 61.3 | 50.3 | 65.4 | 74.3 | 68.4 | 81.6 | 63.7 | 78.9 | 75.4 | 91.7 | 79.1 |
| ID41364 | 242.0 | 148.0 | 170.6 | 104.7 | 128.5 | 144.1 | 52.6 | 73.2 | 35.5 | 65.6 | 48.6 | 109.5 |
| ID414 | 105.3 | 113.1 | 106.9 | 121.4 | 119.7 | 116.8 | 114.3 | 126.9 | 114.9 | 111.8 | 106.2 | 101.4 |
| ID415 | 80.1 | 72.3 | 74.1 | 73.0 | 75.0 | 77.2 | 85.1 | 80.8 | 76.7 | 73.5 | 89.6 | 86.5 |
| ID416 | 92.0 | 92.3 | 86.8 | 95.8 | 98.2 | 95.1 | 101.8 | 101.1 | 94.5 | 93.7 | 98.0 | 99.3 |
| ID417 | 94.3 | 116.7 | 282.8 | 97.4 | 214.6 | 97.4 | 67.4 | 26.4 | 78.9 | 276.1 | 103.6 | 88.6 |
| ID418 | 139.8 | 135.3 | 129.6 | 78.1 | 95.3 | 105.0 | 95.8 | 77.9 | 98.5 | 97.6 | 138.3 | 123.1 |
| ID419 | 104.1 | 110.2 | 103.4 | 104.4 | 123.7 | 114.0 | 103.0 | 124.8 | 105.0 | 117.1 | 102.4 | 110.6 |
| ID420 | 140.0 | 72.6 | 72.4 | 54.7 | 63.3 | 58.4 | 88.9 | 74.7 | 78.1 | 94.7 | 84.7 | 78.3 |
| ID42004 | 177.2 | 129.9 | 143.4 | 85.9 | 130.6 | 72.7 | 40.6 | 51.7 | 39.6 | 98.2 | 54.5 | 87.0 |
| ID42094 | 130.1 | 70.4 | 93.7 | 71.3 | 76.1 | 73.9 | 63.7 | 72.4 | 150.3 | 77.4 | 79.4 | 82.6 |
| ID421 | 214.5 | 146.8 | 136.0 | 77.3 | 88.4 | 94.7 | 85.8 | 74.9 | 88.0 | 76.9 | 120.7 | 92.1 |
| ID422 | 164.0 | 152.9 | 145.2 | 85.5 | 93.1 | 104.3 | 98.4 | 80.9 | 96.4 | 89.6 | 133.8 | 112.4 |
| ID423 | 144.3 | 152.9 | 141.3 | 83.0 | 90.7 | 86.5 | 90.1 | 82.9 | 84.0 | 90.4 | 105.3 | 90.0 |
| ID424 | 111.3 | 125.0 | 126.3 | 80.3 | 83.8 | 81.6 | 89.8 | 77.7 | 82.2 | 85.7 | 116.0 | 109.9 |
| ID42467 | 155.5 | 132.7 | 137.7 | 58.9 | 69.1 | 55.8 | 52.6 | 46.2 | 44.0 | 65.9 | 41.2 | 51.6 |
| ID425 | 196.6 | 216.7 | 197.8 | 90.5 | 96.5 | 85.9 | 78.9 | 84.0 | 77.8 | 85.3 | 83.5 | 72.5 |
| ID426 | 180.0 | 197.9 | 200.7 | 86.6 | 88.6 | 84.7 | 82.4 | 81.3 | 76.9 | 92.6 | 78.7 | 77.2 |
| ID428 | 131.7 | 115.9 | 116.7 | 122.0 | 89.9 | 100.1 | 98.8 | 68.5 | 85.0 | 102.3 | 75.1 | 91.3 |
| ID42828 | 66.8 | 76.1 | 83.1 | 95.8 | 72.5 | 101.1 | 110.5 | 134.3 | 94.2 | 94.5 | 98.1 | 131.5 |
| ID429 | 150.8 | 119.8 | 111.7 | 66.1 | 74.2 | 80.8 | 84.9 | 62.8 | 79.4 | 70.3 | 98.5 | 87.4 |
| ID430 | 89.1 | 80.7 | 76.7 | 74.6 | 72.7 | 85.0 | 80.2 | 75.1 | 75.5 | 73.9 | 98.8 | 106.2 |
| ID431 | 223.2 | 203.1 | 171.9 | 54.1 | 67.0 | 82.9 | 79.8 | 109.4 | 56.6 | 72.3 | 107.9 | 90.8 |
| ID432 | 89.4 | 78.5 | 84.6 | 81.6 | 80.4 | 69.1 | 70.4 | 76.4 | 81.0 | 106.7 | 123.1 | 144.0 |
| ID43224 | 102.0 | 97.5 | 82.9 | 93.7 | 85.7 | 85.9 | 107.1 | 86.9 | 87.8 | 99.0 | 84.2 | 88.9 |
| ID433 | 121.3 | 93.7 | 98.7 | 68.1 | 49.9 | 71.3 | 64.3 | 53.3 | 81.1 | 49.3 | 199.9 | 351.7 |
| ID434 | 84.9 | 79.4 | 73.0 | 76.6 | 69.7 | 79.4 | 82.0 | 80.9 | 74.1 | 105.9 | 124.1 | 120.6 |
| ID435 | 91.3 | 77.0 | 77.1 | 92.2 | 90.1 | 102.0 | 94.9 | 87.0 | 87.0 | 84.1 | 91.3 | 82.3 |
| ID43567 | 102.5 | 99.2 | 96.6 | 100.4 | 101.3 | 114.5 | 101.9 | 97.0 | 103.0 | 117.2 | 118.8 | 113.7 |
| ID436 | 162.5 | 135.3 | 150.1 | 84.7 | 75.1 | 82.5 | 83.4 | 81.9 | 82.6 | 116.6 | 85.0 | 87.9 |
| ID437 | 114.1 | 90.0 | 92.9 | 72.0 | 78.3 | 89.6 | 80.9 | 74.3 | 89.3 | 72.4 | 115.9 | 114.0 |
| ID439 | 187.3 | 692.0 | 275.3 | 66.0 | 56.2 | 79.8 | 75.5 | 46.5 | 67.1 | 54.1 | 76.3 | 53.5 |
| ID43951 | 82.4 | 86.6 | 80.4 | 94.7 | 93.9 | 116.7 | 104.0 | 99.4 | 105.3 | 104.5 | 121.9 | 121.7 |
| ID440 | 121.7 | 94.3 | 101.4 | 82.9 | 93.0 | 105.6 | 89.6 | 83.4 | 87.1 | 81.1 | 108.2 | 103.0 |
| ID441 | 147.8 | 174.8 | 161.1 | 75.8 | 79.5 | 80.0 | 75.9 | 72.7 | 73.4 | 78.0 | 16.1 | 77.9 |
| ID442 | 82.3 | 74.5 | 104.6 | 89.0 | 96.5 | 98.1 | 108.5 | 112.4 | 107.7 | 110.8 | 111.6 | 99.9 |
| ID443 | 107.4 | 97.7 | 102.6 | 114.8 | 110.5 | 122.2 | 105.1 | 110.9 | 103.1 | 102.4 | 98.5 | 88.1 |
| ID44325 | 103.6 | 108.3 | 94.1 | 104.5 | 89.6 | 77.8 | 84.0 | 79.8 | 94.0 | 101.0 | 93.8 | 87.6 |
| ID444 | 67.6 | 64.5 | 75.6 | 71.5 | 78.2 | 80.5 | 96.8 | 98.3 | 106.9 | 116.5 | 115.9 | 108.7 |
| ID445 | 114.3 | 83.7 | 99.9 | 89.0 | 100.5 | 108.6 | 94.2 | 89.1 | 92.6 | 86.2 | 96.0 | 100.7 |
| ID446 | 120.2 | 104.9 | 119.3 | 126.7 | 113.0 | 126.1 | 104.5 | 110.0 | 96.5 | 104.0 | 91.9 | 87.6 |
| ID447 | 110.7 | 109.0 | 151.5 | 114.4 | 109.7 | 126.0 | 112.4 | 126.6 | 90.5 | 98.2 | 100.5 | 90.6 |
| ID448 | 154.7 | 162.3 | 188.5 | 95.4 | 97.3 | 108.4 | 90.0 | 111.6 | 79.7 | 91.3 | 93.5 | 79.5 |
| ID449 | 169.9 | 200.4 | 150.4 | 88.4 | 88.7 | 97.9 | 80.7 | 91.6 | 83.3 | 84.5 | 79.2 | 67.7 |
| ID450 | 101.2 | 109.3 | 104.7 | 94.5 | 116.2 | 96.8 | 106.2 | 103.3 | 85.0 | 135.3 | 104.1 | 92.5 |
| ID45022 | 111.3 | 100.1 | 116.9 | 99.4 | 108.1 | 94.5 | 91.5 |  | 108.6 | 121.5 | 134.9 |  |
| ID45086 | 80.0 | 84.3 | 92.7 | 105.7 | 104.7 | 101.6 | 99.0 | 102.7 | 104.5 | 104.5 | 102.8 | 107.7 |
| ID451 | 164.9 | 148.0 | 139.6 | 129.7 | 121.4 | 120.6 | 98.2 | 108.7 | 105.7 | 116.0 | 88.1 | 91.0 |
| ID452 | 153.9 | 89.7 | 67.0 | 74.1 | 101.6 | 166.7 | 125.0 | 58.7 | 137.1 | 107.4 | 330.3 | 195.9 |
| ID453 | 93.2 | 108.8 | 64.4 | 85.0 | 98.0 | 81.5 | 89.4 | 97.6 | 94.5 | 127.7 | 90.3 | 86.5 |
| ID454 | 98.4 | 103.3 | 102.8 | 111.5 | 114.8 | 123.8 | 109.9 | 122.7 | 111.6 | 98.0 | 93.9 | 84.8 |
| ID45467 | 121.8 | 122.4 | 169.6 | 105.0 | 113.0 | 108.0 | 91.6 | 110.3 | 109.0 | 112.4 | 137.2 | 113.9 |
| ID455 | 105.6 | 72.9 | 85.6 | 86.7 | 105.5 | 93.2 | 99.8 | 86.6 | 109.1 | 128.7 | 100.4 | 105.5 |
| ID458 | 245.6 | 112.8 | 97.9 | 86.8 | 74.6 | 107.0 | 93.4 | 49.7 | 79.9 | 86.0 | 66.5 | 96.4 |
| ID45861 | 91.2 | 87.8 | 111.4 | 116.2 | 118.2 | 118.9 | 115.4 | 124.5 | 114.4 | 117.0 | 113.1 | 120.4 |
| ID459 | 141.1 | 135.4 | 144.9 | 100.0 | 111.0 | 95.6 | 100.9 | 114.3 | 97.8 | 124.8 | 93.4 | 84.3 |
| ID460 | 105.5 | 95.2 | 96.8 | 100.5 | 120.2 | 103.8 | 101.9 | 107.7 | 95.1 | 125.6 | 103.4 | 95.6 |
| ID461 | 213.6 | 60.3 | 64.1 | 31.8 | 99.2 | 156.9 | 119.3 | 35.2 | 120.0 | 93.1 | 278.3 | 174.5 |
| ID462 | 123.5 | 106.9 | 117.8 | 114.6 | 110.7 | 104.1 | 107.4 | 106.6 | 100.0 | 137.6 | 110.6 | 102.0 |
| ID46261 | 89.8 | 77.3 | 94.5 | 103.8 | 96.8 | 128.4 | 127.7 | 110.9 | 113.3 | 99.5 | 123.6 | 134.9 |
| ID464 | 99.8 | 83.8 | 84.1 | 110.8 | 127.7 | 121.1 | 108.4 | 134.7 | 113.5 | 161.0 | 146.1 | 141.1 |
| ID465 | 82.4 | 82.8 | 85.3 | 102.2 | 121.2 | 110.1 | 110.5 | 128.0 | 101.3 | 155.0 | 109.5 | 99.6 |
| ID466 | 170.7 | 149.3 | 142.9 | 122.7 | 98.8 | 81.6 | 103.5 | 80.2 | 100.3 | 83.8 | 76.0 | 73.3 |
| ID46682 | 96.4 | 71.4 | 72.9 | 77.4 | 86.5 | 102.7 | 137.3 | 94.6 | 99.1 | 80.9 | 116.1 | 121.1 |
| ID467 | 78.8 | 64.1 | 87.1 | 80.8 | 89.9 | 88.2 | 91.6 | 96.5 | 114.1 | 118.7 | 88.2 | 93.2 |
| ID468 | 154.1 | 175.3 | 153.2 | 123.5 | 113.2 | 96.5 | 106.8 | 93.1 | 104.0 | 88.6 | 75.7 | 75.3 |
| ID47101 | 100.3 | 121.5 | 116.2 | 121.5 | 231.1 | 114.7 | 110.7 | 192.7 | 82.2 | 247.3 | 93.5 | 140.8 |
| ID47424 | 96.5 | 112.1 | 106.6 | 83.9 | 106.9 | 95.5 | 106.7 | 98.6 | 106.8 | 97.3 | 115.1 | 112.0 |
| ID47447 | 68.9 | 83.2 | 62.6 | 83.5 | 136.7 | 104.9 | 99.5 | 126.0 | 109.4 | 203.8 | 133.1 | 97.1 |
| ID47459 | 97.7 | 97.8 | 86.5 | 68.6 | 97.1 | 78.3 | 66.1 | 59.6 | 76.8 | 56.0 | 270.0 | 237.8 |
| ID47497 | 79.7 | 63.9 | 69.3 | 101.7 | 95.3 | 93.7 | 106.7 | 108.9 | 106.3 | 131.4 | 141.4 | 125.3 |
| ID475 | 429.8 | 110.8 | 702.6 | 125.3 | 176.8 | 93.6 | 141.3 | 38.3 | 56.7 | 115.9 | 51.7 | 54.6 |
| ID47548 | 109.6 | 103.3 | 79.1 | 89.9 | 85.8 | 119.7 | 90.4 | 119.3 | 96.9 | 100.7 | 112.9 | 111.5 |
| ID476 | 122.2 | 97.4 | 92.2 | 83.7 | 86.7 | 83.3 | 93.9 | 83.7 | 96.0 | 81.4 | 92.1 | 89.4 |
| ID477 | 151.8 | 128.8 | 128.1 | 100.8 | 108.7 | 114.2 | 109.2 | 97.3 | 110.2 | 93.5 | 105.2 | 100.0 |
| ID478 | 133.9 | 114.6 | 138.0 | 92.3 | 86.6 | 83.9 | 101.1 | 90.1 | 91.2 | 115.3 | 107.5 | 99.2 |
| ID479 | 122.5 | 134.9 | 129.6 | 85.1 | 87.5 | 96.3 | 100.6 | 104.7 | 93.6 | 101.8 | 106.3 | 98.5 |
| ID47993 | 99.5 | 124.8 | 134.2 | 49.3 | 65.9 | 58.4 | 69.4 | 70.7 | 73.5 | 79.5 | 96.9 | 84.3 |
| ID480 | 86.0 | 84.0 | 91.3 | 81.4 | 81.3 | 87.3 | 102.4 | 103.0 | 93.0 | 115.8 | 112.0 | 101.8 |
| ID481 | 121.0 | 45.7 | 134.4 | 48.8 | 113.5 | 204.2 | 156.8 | 27.7 | 132.3 | 93.0 | 380.7 | 290.3 |
| ID482 | 95.9 | 99.2 | 102.8 | 96.1 | 93.2 | 87.0 | 113.9 | 113.9 | 103.1 | 122.9 | 108.9 | 109.1 |
| ID483 | 129.0 | 115.4 | 112.5 | 97.1 | 97.9 | 154.1 | 138.5 | 85.2 | 106.9 | 95.4 | 148.7 | 135.6 |
| ID484 | 89.2 | 100.0 | 104.9 | 108.9 | 104.0 | 116.8 | 113.4 | 126.7 | 109.7 | 111.8 | 102.8 | 90.6 |
| ID48461 | 115.5 | 121.0 | 130.0 | 70.8 | 84.0 | 80.2 | 61.4 | 75.4 | 76.7 | 84.7 | 80.4 | 66.3 |
| ID485 | 82.2 | 84.2 | 79.5 | 94.2 | 97.0 | 87.1 | 92.4 | 103.2 | 93.0 | 101.0 | 93.0 | 101.7 |
| ID486 | 88.5 | 92.0 | 119.3 | 91.3 | 83.5 | 74.8 | 97.0 | 93.7 | 93.4 | 124.2 | 107.6 | 104.4 |
| ID487 | 113.3 | 111.8 | 115.9 | 112.1 | 108.7 | 115.1 | 109.4 | 111.3 | 108.4 | 104.1 | 101.3 | 99.9 |
| ID48834 | 59.9 | 63.3 | 54.0 | 100.0 | 85.9 | 78.9 | 104.0 | 87.5 | 92.9 | 102.7 | 104.9 | 91.7 |
| ID48841 | 2482.4 | 138.1 | 418.8 | 111.8 | 181.4 | 114.3 | 102.1 | 183.0 | 43.8 | 555.2 | 75.4 | 170.5 |
| ID48862 | 87.2 | 92.1 | 79.8 | 102.0 | 65.1 | 97.6 | 79.3 | 67.7 | 85.9 | 99.2 | 71.0 | 120.4 |
| ID489 | 97.7 | 103.7 | 113.2 | 108.4 | 106.0 | 115.3 | 113.2 | 141.8 | 115.4 | 114.2 | 113.8 | 93.8 |
| ID490 | 93.3 | 95.3 | 99.1 | 111.1 | 115.2 | 115.2 | 115.3 | 116.3 | 114.1 | 108.0 | 99.9 | 103.9 |
| ID491 | 90.6 | 86.4 | 87.6 | 79.7 | 87.3 | 83.0 | 96.0 | 84.5 | 91.9 | 84.7 | 85.7 | 89.6 |
| ID49134 | 88.3 | 93.3 | 89.3 | 105.9 | 105.7 | 112.4 | 111.7 | 106.0 | 106.2 | 106.4 | 106.3 | 108.8 |
| ID492 | 103.8 | 98.9 | 101.0 | 108.3 | 106.5 | 110.2 | 122.3 | 121.8 | 121.4 | 119.2 | 110.6 | 113.4 |
| ID493 | 94.6 | 94.5 | 91.3 | 98.2 | 98.1 | 101.2 | 115.9 | 105.3 | 106.4 | 106.1 | 101.9 | 106.5 |
| ID494 | 86.3 | 63.6 | 78.4 | 74.2 | 89.6 | 82.7 | 96.8 | 86.7 | 88.5 | 95.4 | 93.1 | 93.5 |
| ID495 | 130.9 | 115.5 | 128.3 | 97.2 | 96.1 | 102.1 | 109.1 | 97.4 | 99.8 | 100.0 | 91.6 | 95.6 |
| ID49592 | 85.2 | 97.2 | 97.3 | 92.9 | 80.2 | 104.0 | 86.0 | 88.9 | 80.1 | 90.9 | 85.4 | 81.9 |
| ID496 | 108.7 | 111.1 | 110.3 | 107.9 | 111.2 | 113.9 | 113.9 | 112.8 | 106.9 | 110.9 | 111.6 | 106.8 |
| ID497 | 106.4 | 105.8 | 103.7 | 100.5 | 90.2 | 97.9 | 97.3 | 94.0 | 97.2 | 93.1 | 101.7 | 91.5 |
| ID498 | 73.0 | 97.8 | 91.8 | 109.5 | 87.9 | 90.6 | 89.1 | 101.3 | 93.6 | 94.2 | 79.0 | 93.3 |
| ID499 | 53.5 | 55.2 | 67.9 | 74.2 | 74.2 | 73.7 | 92.8 | 87.8 | 88.8 | 103.1 | 91.2 | 99.0 |
| ID501 | 36.6 | 60.3 | 33.1 | 95.3 | 68.3 | 83.4 | 91.4 | 104.3 | 53.8 | 85.0 | 68.6 | 107.2 |
| ID502 | 127.1 | 138.3 | 132.5 | 86.4 | 90.9 | 94.2 | 80.9 | 76.3 | 72.2 | 85.6 | 92.5 | 90.6 |
| ID503 | 169.7 | 121.7 | 163.6 | 70.3 | 99.8 | 115.7 | 94.7 | 76.2 | 139.2 | 92.0 | 135.7 | 118.9 |
| ID504 | 52.9 | 46.7 | 50.1 | 63.0 | 86.0 | 58.2 | 80.6 | 84.1 | 86.1 | 105.3 | 93.1 | 109.2 |
| ID505 | 50.4 | 37.6 | 55.2 | 52.8 | 66.9 | 51.4 | 70.2 | 65.7 | 76.2 | 84.8 | 75.4 | 81.9 |
| ID506 | 108.7 | 121.2 | 151.3 | 83.2 | 102.2 | 67.1 | 71.4 | 111.9 | 96.3 | 108.5 | 122.6 | 125.1 |
| ID508 | 99.1 | 104.2 | 100.3 | 107.4 | 105.9 | 115.1 | 110.7 | 112.8 | 109.6 | 107.4 | 101.9 | 99.7 |
| ID509 | 118.5 | 135.4 | 128.2 | 98.7 | 101.6 | 124.2 | 160.9 | 114.5 | 112.9 | 111.1 | 116.6 | 121.0 |
| ID510 | 146.1 | 171.5 | 150.5 | 93.4 | 92.2 | 109.3 | 106.5 | 107.6 | 97.9 | 101.2 | 86.9 | 92.2 |
| ID511 | 87.1 | 97.5 | 93.4 | 99.0 | 100.2 | 103.5 | 120.7 | 109.6 | 109.7 | 110.6 | 103.7 | 108.7 |
| ID512 | 109.3 | 114.6 | 112.5 | 106.1 | 106.0 | 112.0 | 109.5 | 109.4 | 105.3 | 106.4 | 97.5 | 99.0 |
| ID513 | 85.5 | 94.8 | 102.8 | 81.0 | 79.0 | 73.7 | 82.4 | 87.0 | 85.4 | 94.3 | 78.4 | 81.7 |
| ID514 | 102.0 | 108.6 | 111.1 | 93.9 | 92.0 | 90.3 | 95.0 | 103.0 | 94.9 | 106.8 | 88.0 | 86.5 |
| ID515 | 78.7 | 78.0 | 85.9 | 74.4 | 75.9 | 78.3 | 88.4 | 91.5 | 80.9 | 99.1 | 85.6 | 86.0 |
| ID516 | 97.9 | 101.6 | 103.1 | 94.1 | 100.7 | 105.4 | 106.3 | 105.7 | 100.7 | 94.7 | 89.2 | 98.9 |
| ID51628 | 90.5 | 72.5 | 56.1 | 109.6 | 103.3 | 115.3 | 121.6 | 92.9 | 132.5 | 110.7 | 115.7 | 121.6 |
| ID517 | 107.4 | 97.6 | 109.6 | 88.5 | 99.6 | 103.5 | 94.9 | 94.3 | 99.0 | 80.1 | 92.5 | 90.1 |
| ID518 | 81.2 | 69.8 | 78.7 | 86.0 | 94.2 | 92.1 | 106.5 | 112.0 | 104.8 | 125.4 | 119.4 | 127.8 |
| ID520 | 74.3 | 59.5 | 69.2 | 69.6 | 74.0 | 75.8 | 101.0 | 83.3 | 86.5 | 86.4 | 97.4 | 103.2 |
| ID521 | 66.6 | 58.9 | 60.4 | 78.2 | 80.1 | 81.0 | 114.3 | 120.5 | 115.1 | 143.9 | 132.0 | 124.3 |
| ID522 | 90.3 | 87.0 | 94.4 | 87.8 | 105.9 | 92.1 | 101.8 | 100.7 | 90.8 | 96.8 | 111.0 | 88.4 |
| ID523 | 100.5 | 113.3 | 104.8 | 77.6 | 75.4 | 78.7 | 92.8 | 87.0 | 79.3 | 87.1 | 91.5 | 95.4 |
| ID524 | 77.9 | 63.6 | 68.8 | 84.3 | 79.1 | 81.8 | 114.1 | 98.0 | 101.4 | 118.4 | 116.7 | 97.4 |
| ID525 | 78.4 | 72.0 | 80.3 | 85.2 | 88.7 | 91.5 | 103.4 | 106.1 | 96.2 | 105.0 | 101.3 | 105.7 |
| ID526 | 80.7 | 71.9 | 75.3 | 89.5 | 84.9 | 84.7 | 107.6 | 94.2 | 97.5 | 107.3 | 112.9 | 104.8 |
| ID52622 | 297.0 | 345.9 | 269.3 | 50.7 | 39.3 | 75.2 | 38.4 | 44.9 | 26.3 | 34.5 | 42.8 | 38.2 |
| ID527 | 84.0 | 97.6 | 101.0 | 112.8 | 101.7 | 113.7 | 114.1 | 119.9 | 119.2 | 101.9 | 96.7 | 112.8 |
| ID528 | 76.1 | 73.7 | 74.8 | 86.7 | 87.5 | 86.6 | 96.3 | 92.3 | 100.3 | 89.1 | 91.3 | 101.0 |
| ID529 | 152.2 | 124.4 | 185.2 | 111.3 | 88.9 | 102.2 | 93.0 | 85.3 | 86.9 | 77.3 | 74.7 | 72.1 |
| ID531 | 99.4 | 94.9 | 98.0 | 100.8 | 99.7 | 109.0 | 104.7 | 104.8 | 103.8 | 101.8 | 108.2 | 105.6 |
| ID532 | 80.3 | 73.1 | 76.4 | 69.0 | 84.4 | 73.9 | 86.7 | 82.4 | 83.9 | 96.4 | 80.9 | 88.7 |
| ID533 | 107.9 | 110.4 | 134.4 | 124.9 | 122.7 | 122.2 | 121.7 | 132.1 | 129.8 | 117.2 | 111.3 | 116.8 |
| ID534 | 94.9 | 95.1 | 100.7 | 108.7 | 112.7 | 115.2 | 110.9 | 107.8 | 109.3 | 100.1 | 98.3 | 101.6 |
| ID535 | 98.2 | 94.6 | 96.9 | 102.9 | 104.5 | 110.0 | 114.7 | 110.2 | 109.8 | 107.1 | 112.7 | 108.1 |
| ID536 | 101.3 | 105.3 | 108.4 | 106.0 | 102.9 | 117.4 | 114.5 | 110.1 | 105.0 | 104.9 | 109.7 | 105.6 |
| ID537 | 72.6 | 70.6 | 63.2 | 73.9 | 78.9 | 82.2 | 91.5 | 88.4 | 72.8 | 84.1 | 91.1 | 100.0 |
| ID538 | 96.2 | 105.0 | 112.8 | 125.2 | 112.3 | 116.7 | 111.2 | 111.7 | 124.9 | 112.9 | 102.0 | 101.8 |
| ID539 | 59.3 | 54.2 | 62.0 | 76.4 | 81.7 | 78.8 | 100.0 | 98.3 | 103.6 | 122.1 | 126.4 | 125.5 |
| ID540 | 83.1 | 82.9 | 87.2 | 110.1 | 104.0 | 107.7 | 96.8 | 104.8 | 101.6 | 102.9 | 92.0 | 98.5 |
| ID54032 | 81.6 | 100.7 | 88.7 | 104.0 | 82.7 | 113.8 | 106.0 | 95.7 | 95.4 | 79.9 | 86.3 | 90.9 |
| ID542 | 164.4 | 167.8 | 163.0 | 91.8 | 110.5 | 106.7 | 101.7 | 111.3 | 101.5 | 109.5 | 110.6 | 117.0 |
| ID543 | 66.4 | 68.3 | 59.7 | 82.6 | 56.9 | 83.1 | 94.4 | 73.5 | 74.2 | 80.0 | 85.8 | 76.0 |
| ID545 | 91.3 | 98.8 | 100.7 | 105.6 | 113.0 | 116.5 | 113.0 | 109.9 | 110.6 | 108.1 | 101.7 | 105.4 |
| ID54565 | 62.3 | 81.5 | 84.3 | 77.2 | 57.1 | 90.4 | 90.7 | 83.5 | 92.1 | 57.5 | 73.7 | 84.9 |
| ID546 | 80.7 | 73.1 | 77.6 | 91.5 | 88.8 | 98.7 | 94.3 | 101.2 | 87.5 | 98.3 | 98.7 | 102.7 |
| ID547 | 94.5 | 96.4 | 105.1 | 122.1 | 101.1 | 134.1 | 114.6 | 127.3 | 112.2 | 110.3 | 99.2 | 116.0 |
| ID548 |  | 76.7 | 89.7 | 90.2 | 91.8 | 95.4 | 86.7 | 95.6 | 100.4 | 99.7 |  | 98.8 |
| ID549 | 73.7 | 72.7 | 70.2 | 74.9 | 93.6 | 77.7 | 83.3 | 80.4 | 82.1 | 106.4 | 95.8 | 90.3 |
| ID550 | 88.7 | 78.9 | 82.7 | 85.4 | 101.5 | 95.2 | 111.7 | 94.1 | 103.0 | 101.4 | 98.8 | 104.4 |
| ID55007 | 110.1 | 95.6 | 114.2 | 100.0 | 118.1 | 108.9 | 98.9 | 97.5 | 103.3 | 123.8 | 97.4 | 78.9 |
| ID551 | 82.1 | 79.1 | 73.8 | 110.0 | 98.7 | 112.1 | 106.7 | 111.3 | 93.5 | 106.0 | 103.0 | 107.8 |
| ID55186 | 239.5 | 105.4 | 151.3 | 119.0 | 226.2 | 165.5 | 107.3 | 151.5 | 138.4 | 227.7 | 134.6 | 101.6 |
| ID552 | 80.3 | 205.2 | 94.4 | 116.0 | 114.4 | 109.4 | 119.0 | 124.0 | 129.1 | 134.4 | 104.6 | 132.0 |
| ID553 | 130.8 | 133.9 | 134.9 | 114.5 | 114.5 | 121.9 | 123.2 | 112.9 | 108.8 | 100.5 | 93.6 | 95.0 |
| ID555 | 110.5 | 108.9 | 107.2 | 89.1 | 88.8 | 90.6 | 97.0 | 87.3 | 90.7 | 87.7 | 90.5 | 93.1 |
| ID556 | 100.1 | 120.0 | 111.7 | 101.6 | 99.6 | 108.0 | 110.3 | 105.4 | 98.7 | 114.0 | 106.0 | 96.9 |
| ID557 | 103.0 | 89.3 | 80.8 | 100.4 | 95.2 | 111.2 | 93.0 | 113.4 | 110.5 | 93.3 | 96.6 | 123.0 |
| ID558 | 120.4 | 129.7 | 131.6 | 67.0 | 73.1 | 76.8 | 83.4 | 76.2 | 76.6 | 75.5 | 89.6 | 87.8 |
| ID560 | 126.1 | 131.1 | 135.6 | 108.4 | 108.0 | 117.0 | 116.8 | 113.0 | 103.6 | 93.6 | 91.0 | 100.0 |
| ID561 | 102.9 | 99.8 | 103.6 | 83.3 | 82.2 | 82.6 | 90.9 | 83.4 | 83.8 | 89.4 | 85.9 | 82.4 |
| ID562 | 102.9 | 107.4 | 110.1 | 109.0 | 105.3 | 114.3 | 105.0 | 111.3 | 101.6 | 110.0 | 104.0 | 106.2 |
| ID563 | 123.5 | 129.6 | 122.6 | 95.9 | 90.9 | 97.3 | 95.2 | 88.6 | 88.0 | 80.1 | 84.2 | 85.7 |
| ID564 | 77.6 | 85.3 | 103.8 | 109.4 | 102.3 | 82.8 | 63.0 | 81.4 | 80.5 | 92.6 | 74.5 | 71.2 |
| ID565 | 95.0 | 125.4 | 109.3 | 94.7 | 103.6 | 103.8 | 99.7 | 100.0 | 91.4 | 102.1 | 84.0 | 94.1 |
| ID566 | 128.0 | 149.0 | 135.6 | 120.0 | 103.7 | 124.6 | 110.0 | 106.6 | 98.8 | 92.8 | 85.3 | 82.7 |
| ID567 | 67.4 | 57.0 | 60.1 | 83.2 | 92.7 | 81.1 | 101.5 | 60.5 | 74.1 | 97.0 | 80.3 | 63.3 |
| ID568 | 132.0 | 127.9 | 134.1 | 81.0 | 81.0 | 82.9 | 73.4 | 86.5 | 71.1 | 73.1 | 72.3 | 77.9 |
| ID56884 | 135.9 | 138.7 | 148.7 | 75.3 | 60.6 | 73.2 | 77.6 | 70.4 | 74.2 | 67.1 | 72.1 | 65.1 |
| ID570 | 115.3 | 127.1 | 115.9 | 106.6 | 162.2 | 113.3 | 109.6 | 147.4 | 97.9 | 258.0 | 86.7 | 36.5 |
| ID5709 | 68.4 | 85.6 | 78.9 | 63.8 | 75.5 | 85.6 | 77.7 | 90.5 | 67.8 | 108.4 | 88.3 | 75.3 |
| ID571 | 96.4 | 82.4 | 99.8 | 109.0 | 101.2 | 113.0 | 100.7 | 110.1 | 108.8 | 108.1 | 97.3 | 104.0 |
| ID574 | 50.9 | 45.5 | 63.0 | 77.9 | 73.4 | 65.8 | 65.5 | 83.2 | 87.7 | 104.4 | 77.3 | 98.8 |
| ID57409 | 85.5 | 93.4 | 84.2 | 74.6 | 73.7 | 87.5 | 90.6 | 78.0 | 76.5 | 85.4 | 87.9 | 86.4 |
| ID575 | 96.0 | 86.3 | 99.3 | 113.5 | 101.8 | 89.2 | 77.1 | 75.1 | 84.6 | 98.1 | 82.9 | 72.0 |
| ID577 | 76.4 | 75.3 | 85.6 | 97.4 | 86.4 | 91.2 | 73.6 | 99.9 | 96.5 | 90.4 | 83.3 | 80.5 |
| ID57956 | 130.8 | 186.5 | 204.8 | 44.2 | 60.2 | 94.3 | 44.5 | 52.9 | 51.2 | 52.9 | 61.0 | 48.7 |
| ID580 | 64.9 | 66.6 | 67.4 | 90.2 | 101.3 | 108.2 | 90.7 | 76.2 | 80.4 | 107.6 | 95.7 | 59.1 |
| ID581 | 92.7 | 96.9 | 89.1 | 94.7 | 90.3 | 101.8 | 103.9 | 85.3 | 86.9 | 84.1 | 88.5 | 88.8 |
| ID5810 | 125.0 | 98.1 | 105.0 | 186.5 | 172.3 | 156.3 | 185.1 | 157.7 | 159.9 | 153.5 | 154.5 | 205.2 |
| ID583 | 109.3 | 114.1 | 110.7 | 101.3 | 100.0 | 106.9 | 104.9 | 101.0 | 101.7 | 99.5 | 102.4 | 99.7 |
| ID584 | 106.2 | 110.9 | 110.0 | 108.6 | 107.4 | 114.4 | 108.9 | 109.9 | 108.7 | 103.8 | 97.3 | 98.3 |
| ID585 | 78.2 | 89.9 | 77.0 | 95.5 | 91.9 | 99.1 | 101.0 | 91.6 | 90.5 | 75.4 | 79.3 | 82.1 |
| ID58512 | 121.7 | 132.4 | 138.9 | 79.6 | 81.4 | 93.4 | 116.2 | 92.8 | 92.2 | 85.0 | 99.2 | 93.0 |
| ID587 | 106.1 | 106.1 | 104.4 | 95.1 | 93.7 | 102.4 | 101.6 | 97.6 | 97.6 | 92.5 | 99.8 | 96.3 |
| ID588 | 95.3 | 107.7 | 100.6 | 111.0 | 103.4 | 116.1 | 111.2 | 106.9 | 105.1 | 100.9 | 99.2 | 99.3 |
| ID589 | 101.9 | 101.1 | 98.2 | 102.4 | 95.0 | 105.8 | 106.6 | 98.5 | 102.6 | 94.7 | 103.6 | 96.7 |
| ID590 | 91.3 | 91.4 | 95.5 | 125.0 | 116.5 | 111.7 | 98.7 | 107.2 | 103.5 | 109.3 | 97.1 | 102.9 |
| ID59072 | 96.5 | 98.0 | 108.4 | 91.7 | 86.3 | 104.8 | 104.1 | 95.2 | 97.5 | 99.3 | 111.7 | 112.5 |
| ID591 | 94.0 | 86.8 | 84.8 | 90.2 | 93.0 | 96.6 | 97.5 | 89.7 | 98.7 | 94.4 | 99.6 | 102.8 |
| ID592 | 102.1 | 102.8 | 100.1 | 102.0 | 88.1 | 102.7 | 106.5 | 94.8 | 94.7 | 83.6 | 94.4 | 93.3 |
| ID593 | 86.2 | 100.7 | 93.6 | 107.3 | 90.9 | 96.3 | 104.1 | 103.7 | 95.4 | 99.1 | 103.6 | 100.7 |
| ID594 | 104.2 | 100.6 | 100.3 | 105.9 | 102.5 | 103.5 | 97.0 | 103.5 | 96.6 | 104.5 | 94.9 | 98.4 |
| ID595 | 59.1 | 51.6 | 61.3 | 102.0 | 100.2 | 111.6 | 109.6 | 117.0 | 106.9 | 145.5 | 138.3 | 121.3 |
| ID59560 | 95.2 | 104.1 | 102.8 | 114.1 | 113.5 | 126.9 | 151.0 | 115.0 | 127.8 | 119.8 | 119.0 | 130.0 |
| ID597 | 130.5 | 141.1 | 132.5 | 89.9 | 113.8 | 101.7 | 86.2 | 112.9 | 91.5 | 110.6 | 83.8 | 68.6 |
| ID598 | 60.6 | 53.1 | 50.1 | 104.3 | 100.5 | 116.0 | 104.9 | 113.9 | 106.3 | 129.3 | 131.1 | 121.5 |
| ID599 | 74.6 | 80.8 | 91.9 | 116.9 | 111.0 | 106.1 | 100.6 | 108.5 | 105.8 | 105.7 | 87.0 | 99.0 |
| ID600 | 91.4 | 76.7 | 74.1 | 72.2 | 73.6 | 70.2 | 75.2 | 76.9 | 85.8 | 81.4 | 92.8 | 96.1 |
| ID601 | 51.7 | 66.4 | 54.6 | 107.0 | 106.4 | 103.9 | 93.2 | 109.8 | 107.1 | 116.5 | 116.3 | 110.7 |
| ID602 | 94.1 | 119.5 | 111.8 | 96.2 | 98.9 | 110.2 | 106.9 | 97.9 | 96.0 | 100.5 | 110.7 | 103.9 |
| ID60367 | 90.0 | 109.7 | 109.5 | 88.9 | 90.7 | 94.8 | 129.3 | 100.5 | 102.5 | 107.1 | 101.3 | 119.9 |
| ID605 | 70.6 | 69.0 | 57.0 | 109.4 | 112.1 | 116.2 | 112.6 | 110.2 | 111.1 | 113.6 | 106.7 | 110.6 |
| ID606 | 65.8 | 91.3 | 50.5 | 103.3 | 77.4 | 98.3 | 96.2 | 73.8 | 70.0 | 75.2 | 88.9 | 84.0 |
| ID607 | 57.8 | 48.8 | 51.2 | 67.1 | 93.2 | 74.7 | 86.0 | 89.9 | 87.4 | 100.8 | 94.7 | 100.6 |
| ID608 | 59.0 | 61.1 | 76.6 | 91.1 | 100.7 | 90.5 | 99.7 | 94.3 | 99.3 | 98.9 | 69.8 | 98.3 |
| ID609 | 22.8 | 31.1 | 22.1 | 117.1 | 112.1 | 122.2 | 103.8 | 116.3 | 112.1 | 128.8 | 119.5 | 121.3 |
| ID610 | 54.9 | 47.7 | 51.3 | 108.1 | 104.1 | 121.5 | 104.7 | 108.4 | 103.8 | 120.0 | 115.4 | 110.0 |
| ID612 | 95.8 | 92.5 | 90.9 | 98.3 | 91.5 | 101.3 | 108.3 | 80.3 | 100.1 | 92.5 | 101.0 | 87.2 |
| ID61221 | 90.1 | 92.2 | 94.0 | 79.9 | 81.7 | 86.9 | 100.7 | 78.4 | 88.5 | 90.4 | 93.2 | 97.1 |
| ID613 | 120.0 | 125.5 | 115.0 | 106.5 | 100.7 | 114.6 | 103.9 | 109.8 | 103.7 | 102.1 | 104.5 | 102.5 |
| ID614 | 45.3 | 40.4 | 48.0 | 94.2 | 87.3 | 99.8 | 116.2 | 120.8 | 114.7 | 171.4 | 131.1 | 112.3 |
| ID615 | 28.5 | 35.6 | 32.5 | 105.5 | 116.1 | 114.4 | 111.8 | 131.6 | 128.3 | 157.9 | 142.9 | 129.5 |
| ID616 | 39.2 | 26.5 | 34.5 | 79.4 | 80.6 | 93.9 | 100.0 | 100.1 | 96.6 | 147.4 | 113.5 | 100.0 |
| ID617 | 9.4 | 47.9 | 47.1 | 112.2 | 102.5 | 117.9 | 116.1 | 94.6 | 95.1 | 115.6 | 104.1 | 90.7 |
| ID618 | 121.8 | 132.3 | 126.5 | 89.3 | 92.4 | 99.7 | 93.5 | 96.4 | 90.4 | 102.9 | 101.3 | 105.4 |
| ID619 | 105.1 | 84.0 | 68.4 | 123.1 | 107.7 | 119.2 | 135.1 | 92.1 | 94.0 | 82.4 | 99.5 | 102.6 |
| ID620 | 117.8 | 118.0 | 123.2 | 89.5 | 87.1 | 97.7 | 88.2 | 93.0 | 86.7 | 97.0 | 95.0 | 102.4 |
| ID62005 | 100.5 | 102.4 | 100.7 | 82.0 | 87.5 | 127.5 | 138.3 | 98.6 | 96.1 | 96.5 | 123.3 | 131.1 |
| ID621 | 71.2 | 72.8 | 74.2 | 99.8 | 103.5 | 105.8 | 108.3 | 113.5 | 108.2 | 116.8 | 106.0 | 112.4 |
| ID623 | 80.7 | 66.8 | 66.5 | 74.8 | 82.9 | 90.2 | 98.1 | 84.7 | 82.1 | 79.4 | 89.3 | 98.2 |
| ID624 | 86.4 | 71.3 | 70.9 | 99.6 | 110.2 | 122.8 | 103.6 | 111.0 | 99.6 | 127.2 | 109.0 | 101.1 |
| ID626 | 76.3 | 62.0 | 58.4 | 75.6 | 81.6 | 92.1 | 99.8 | 88.1 | 83.4 | 90.7 | 105.7 | 113.3 |
| ID62635 | 593.6 | 116.1 | 133.1 | 92.2 | 86.7 | 95.5 | 99.6 | 77.8 | 103.9 | 99.9 | 173.9 | 117.9 |
| ID627 | 83.0 | 63.0 | 61.7 | 81.6 | 87.3 | 103.2 | 100.8 | 89.8 | 89.7 | 103.2 | 123.7 | 115.9 |
| ID628 | 88.0 | 88.1 | 81.1 | 92.7 | 90.9 | 107.2 | 106.0 | 93.4 | 100.8 | 105.0 | 116.6 | 112.2 |
| ID629 | 79.6 | 79.1 | 80.4 | 91.9 | 98.7 | 107.7 | 104.6 | 101.3 | 103.6 | 110.8 | 110.8 | 108.9 |
| ID630 | 97.3 | 75.0 | 77.8 | 88.0 | 94.1 | 109.0 | 102.5 | 81.0 | 93.8 | 96.7 | 121.9 | 113.6 |
| ID631 | 68.0 | 75.2 | 75.7 | 104.4 | 104.6 | 103.6 | 104.2 | 92.0 | 101.3 | 105.9 | 101.6 | 96.6 |
| ID63170 | 154.7 | 113.5 | 97.2 | 89.1 | 63.4 | 84.5 | 85.5 | 49.0 | 92.4 | 64.1 | 60.4 | 88.7 |
| ID632 | 111.1 | 115.7 | 117.5 | 105.8 | 116.7 | 113.2 | 103.7 | 98.5 | 101.6 | 116.6 | 90.9 | 78.0 |
| ID634 | 130.6 | 89.8 | 106.3 | 111.6 | 148.4 | 111.3 | 115.6 | 111.2 | 117.8 | 125.1 | 134.6 | 105.6 |
| ID391 | 65.0 | 59.3 | 60.8 | 69.9 | 66.9 | 67.3 | 101.9 | 92.1 | 96.7 | 138.0 | 140.2 | 142.6 |
| ID637 | 74.3 | 67.6 | 60.0 | 98.2 | 113.3 | 98.7 | 111.4 | 98.8 | 107.9 | 121.3 | 103.0 | 104.7 |
| ID63722 | 158.1 | 139.2 | 131.2 | 80.0 | 86.4 | 87.9 | 88.6 | 77.8 | 90.6 | 80.4 | 105.7 | 83.7 |
| ID639 | 106.1 | 85.3 | 86.5 | 112.9 | 112.7 | 112.6 | 114.6 | 106.7 | 108.3 | 113.5 | 140.0 | 101.1 |
| ID640 | 87.8 | 87.4 | 85.8 | 121.8 | 109.2 | 119.4 | 123.3 | 106.8 | 108.9 | 111.3 | 107.0 | 104.1 |
| ID641 | 44.1 | 17.8 | 33.9 | 50.0 | 65.8 | 58.3 | 95.6 | 82.8 | 91.9 | 161.2 | 165.9 | 182.6 |
| ID642 | 99.3 | 79.3 | 80.7 | 108.8 | 107.4 | 101.1 | 98.8 | 102.4 | 115.8 | 109.1 | 110.4 | 121.7 |
| ID643 | 104.3 | 85.2 | 102.5 | 91.6 | 106.2 | 94.4 | 91.7 | 98.4 | 98.1 | 116.9 | 95.3 | 102.7 |
| ID64389 | 125.9 | 113.1 | 87.7 | 98.4 | 84.4 | 97.4 | 90.6 | 91.6 | 86.8 | 88.8 | 99.5 | 98.8 |
| ID644 | 78.6 | 64.7 | 66.4 | 80.9 | 88.2 | 83.6 | 111.3 | 100.8 | 105.7 | 119.3 | 126.2 | 126.4 |
| ID646 | 92.5 | 109.3 | 86.1 | 105.5 | 96.8 | 112.2 | 112.9 | 90.1 | 93.8 | 89.7 | 105.6 | 119.5 |
| ID647 | 92.5 | 95.1 | 78.6 | 101.6 | 112.6 | 95.4 | 127.8 | 94.8 | 109.2 | 116.8 | 124.8 | 131.5 |
| ID648 | 123.5 | 126.7 | 125.3 | 113.2 | 110.0 | 111.9 | 103.8 | 101.7 | 102.2 | 91.1 | 90.0 | 93.8 |
| ID649 | 102.4 | 111.5 | 95.1 | 116.9 | 118.3 | 117.3 | 114.0 | 108.1 | 105.7 | 99.1 | 83.7 | 90.6 |
| ID653 | 135.9 | 133.7 | 159.1 | 101.2 | 134.4 | 135.2 | 104.2 | 88.8 | 98.5 | 97.8 | 84.5 | 90.8 |
| ID418 | 65.5 | 67.5 | 57.4 | 83.8 | 95.2 | 84.5 | 109.5 | 112.3 | 147.9 | 136.7 | 147.5 | 160.6 |
| ID657 | 127.9 | 124.2 | 128.9 | 113.4 | 112.2 | 115.1 | 103.0 | 103.4 | 102.2 | 92.9 | 90.0 | 91.2 |
| ID658 | 102.7 | 93.5 | 106.0 | 93.2 | 108.1 | 100.6 | 118.3 | 94.7 | 106.5 | 87.5 | 99.0 | 112.6 |
| ID659 | 108.7 | 107.2 | 99.7 | 119.5 | 116.3 | 115.9 | 105.7 | 96.3 | 90.3 | 88.6 | 84.4 | 78.3 |
| ID660 | 107.0 | 90.9 | 103.7 | 88.7 | 116.1 | 93.8 | 95.4 | 135.6 | 789.6 | 114.4 | 84.6 | 119.1 |
| ID662 | 102.4 | 87.0 | 85.2 | 78.6 | 81.8 | 77.5 | 86.1 | 68.7 | 78.4 | 64.6 | 73.1 | 79.7 |
| ID663 | 99.0 | 118.5 | 110.5 | 112.2 | 114.7 | 125.7 | 122.3 | 115.8 | 106.4 | 92.1 | 93.7 | 107.5 |
| ID664 | 110.9 | 89.9 | 106.3 | 91.3 | 121.8 | 95.7 | 95.1 | 106.1 | 98.3 | 107.4 | 104.3 | 107.9 |
| ID667 | 117.7 | 149.6 | 132.5 | 95.1 | 101.0 | 103.8 | 100.8 | 100.9 | 102.1 | 86.5 | 97.4 | 109.2 |
| ID669 | 93.8 | 85.1 | 82.1 | 65.0 | 68.9 | 72.3 | 74.1 | 66.5 | 62.0 | 67.5 | 71.7 | 75.5 |
| ID670 | 135.6 | 137.2 | 137.6 | 73.7 | 82.9 | 78.5 | 74.3 | 72.2 | 61.5 | 79.2 | 64.2 | 73.5 |
| ID671 | 74.8 | 66.4 | 68.4 | 86.1 | 91.2 | 86.1 | 104.5 | 85.3 | 95.4 | 89.7 | 97.4 | 102.4 |
| ID672 | 116.5 | 69.7 | 104.8 | 95.0 | 106.8 | 94.5 | 102.9 | 104.0 | 140.5 | 108.0 | 80.8 | 100.5 |
| ID673 | 80.8 | 67.3 | 72.0 | 72.6 | 79.4 | 81.2 | 97.5 | 80.6 | 86.5 | 76.5 | 92.0 | 89.6 |
| ID674 | 91.3 | 95.2 | 94.0 | 112.2 | 110.6 | 114.1 | 117.1 | 113.1 | 114.4 | 105.3 | 106.4 | 107.6 |
| ID676 | 129.8 | 130.3 | 116.5 | 113.3 | 133.6 | 118.6 | 132.4 | 110.7 | 104.5 | 109.7 | 110.8 | 120.7 |
| ID678 | 75.6 | 76.9 | 70.8 | 76.8 | 85.6 | 91.7 | 94.0 | 40.1 | 72.0 | 79.3 | 68.0 | 44.3 |
| ID679 | 92.9 | 88.5 | 81.3 | 80.4 | 104.6 | 92.0 | 103.4 | 81.5 | 78.5 | 95.2 | 93.3 | 88.1 |
| ID680 | 136.4 | 123.6 | 130.6 | 101.8 | 100.6 | 107.2 | 119.8 | 107.3 | 121.1 | 92.0 | 95.8 | 113.2 |
| ID681 | 107.0 | 99.0 | 101.6 | 105.2 | 101.4 | 107.2 | 96.0 | 104.1 | 97.2 | 94.3 | 96.5 | 100.3 |
| ID682 | 105.1 | 94.0 | 96.6 | 105.1 | 103.7 | 98.6 | 94.2 | 96.6 | 92.5 | 94.2 | 91.3 | 92.4 |
| ID683 | 105.7 | 92.6 | 91.4 | 92.8 | 112.5 | 93.9 | 93.4 | 94.7 | 93.3 | 99.8 | 102.4 | 104.1 |
| ID684 | 226.1 | 194.6 | 226.7 | 92.4 | 86.3 | 101.8 | 98.8 | 74.0 | 76.5 | 76.6 | 71.5 | 70.2 |
| ID685 | 100.9 | 89.4 | 87.6 | 93.0 | 87.1 | 90.1 | 82.9 | 83.4 | 89.9 | 89.3 | 85.4 | 81.2 |
| ID686 | 84.2 | 87.9 | 83.2 | 91.3 | 95.1 | 96.0 | 96.1 | 92.3 | 77.0 | 83.7 | 62.4 | 96.1 |
| ID687 | 112.9 | 113.3 | 96.5 | 115.2 | 101.4 | 122.1 | 112.2 | 98.0 | 95.4 | 90.0 | 92.1 | 98.2 |
| ID688 | 254.4 | 253.7 | 223.2 | 58.4 | 86.3 | 100.7 | 85.9 | 63.3 | 72.4 | 46.8 | 51.3 | 43.6 |
| ID689 | 111.0 | 104.8 | 105.8 | 136.9 | 95.3 | 124.9 | 91.1 | 107.8 | 108.1 | 99.0 | 84.3 | 76.6 |
| ID691 | 74.3 | 64.0 | 81.0 | 83.8 | 61.9 | 73.5 | 63.7 | 91.9 | 85.1 | 52.4 | 60.2 | 87.1 |
| ID692 | 106.2 | 103.3 | 99.6 | 127.0 | 103.0 | 109.8 | 84.0 | 97.2 | 92.6 | 73.6 | 52.5 | 57.0 |
| ID693 | 95.5 | 103.3 | 105.7 | 103.1 | 98.1 | 100.1 | 133.0 | 119.7 | 116.0 | 108.3 | 109.6 | 129.8 |
| ID694 | 95.6 | 94.9 | 136.8 | 102.3 | 92.3 | 108.7 | 107.4 | 170.0 | 145.5 | 85.1 | 95.1 | 195.3 |
| ID695 | 99.3 | 107.1 | 95.7 | 153.6 | 156.3 | 176.8 | 144.6 | 146.8 | 166.1 | 86.2 | 78.5 | 91.9 |
| ID696 | 122.4 | 115.3 | 95.0 | 132.7 | 103.6 | 120.1 | 189.5 | 115.8 | 128.9 | 107.7 | 116.7 | 115.1 |
| ID697 | 79.9 | 66.4 | 60.2 | 120.3 | 120.7 | 136.9 | 164.2 | 159.7 | 162.5 | 139.2 | 167.7 | 207.5 |
| ID698 | 110.4 | 89.6 | 69.4 | 112.3 | 115.9 | 118.5 | 140.8 | 133.8 | 129.9 | 131.3 | 145.5 | 212.6 |
| ID699 | 168.7 | 178.3 | 156.3 | 161.3 | 136.9 | 146.0 | 129.1 | 142.4 | 138.5 | 110.2 | 64.8 | 59.5 |
| ID700 | 104.3 | 92.8 | 113.0 | 77.8 | 104.1 | 104.1 | 83.8 | 75.0 | 95.2 | 104.6 | 78.0 | 73.1 |
| ID701 | 147.9 | 127.2 | 128.5 | 116.2 | 115.3 | 110.8 | 111.0 | 101.0 | 102.9 | 102.0 | 76.2 | 89.5 |
| ID703 | 184.6 | 132.3 | 148.2 | 104.3 | 101.1 | 96.2 | 131.3 | 106.0 | 144.9 | 126.6 | 92.5 | 82.6 |
| ID705 | 98.3 | 82.6 | 99.2 | 97.0 | 90.6 | 112.6 | 99.0 | 129.0 | 104.8 | 131.3 | 90.6 | 77.5 |
| ID707 | 125.5 | 107.4 | 102.7 | 103.1 | 168.5 | 98.4 | 96.0 | 104.1 | 102.2 | 196.6 | 111.0 | 114.9 |
| ID709 | 51.7 | 71.4 | 59.7 | 22.5 | 0.8 | 34.0 | 1.3 | 36.0 | 11.1 | 15.6 | 15.1 | 1.9 |
| ID711 | 102.6 | 107.0 | 101.1 | 104.4 | 98.8 | 106.4 | 108.6 | 102.5 | 101.2 | 92.9 | 100.1 | 99.8 |
| ID71147 | 102.9 | 107.9 | 101.2 | 80.4 | 84.1 | 85.4 | 84.0 | 83.6 | 84.8 | 75.6 | 86.9 | 85.4 |
| ID71199 | 134.0 | 150.2 | 156.9 | 107.0 | 142.6 | 137.3 | 181.8 | 153.2 | 140.8 | 119.5 | 146.0 | 163.7 |
| ID712 | 112.8 | 123.3 | 126.7 | 90.8 | 99.7 | 98.1 | 130.0 | 135.4 | 90.4 | 116.1 | 98.4 | 99.0 |
| ID713 | 193.1 | 182.4 | 194.7 | 94.7 | 88.6 | 95.8 | 131.6 | 113.1 | 117.8 | 122.1 | 86.5 | 90.7 |
| ID714 | 260.2 | 271.4 | 265.6 | 71.7 | 66.6 | 71.1 | 66.5 | 64.0 | 47.8 | 39.7 | 64.8 | 61.6 |
| ID716 | 112.7 | 129.7 | 128.7 | 80.2 | 84.8 | 86.8 | 113.2 | 109.2 | 80.2 | 96.6 | 115.4 | 106.2 |
| ID717 | 114.5 | 150.8 | 109.0 | 167.8 | 114.8 | 146.8 | 153.2 | 92.4 | 191.9 | 91.4 | 112.7 | 109.0 |
| ID719 | 156.5 | 99.0 | 134.6 | 87.0 | 120.4 | 104.0 | 114.3 | 161.8 | 74.4 | 212.5 | 116.0 | 176.1 |
| ID721 | 87.2 | 99.4 | 98.7 | 95.1 | 92.6 | 115.8 | 98.6 | 107.5 | 94.6 | 107.5 | 104.6 | 90.2 |
| ID722 | 119.1 | 164.2 | 215.8 | 80.8 | 57.6 | 95.0 | 102.4 | 90.3 | 64.5 | 54.9 | 94.3 | 75.8 |
| ID723 | 97.5 | 86.4 | 90.7 | 108.8 | 107.9 | 119.0 | 108.8 | 100.4 | 108.1 | 109.4 | 111.0 | 114.9 |
| ID724 | 122.3 | 127.5 | 125.6 | 94.2 | 101.6 | 104.0 | 99.9 | 91.0 | 76.1 | 84.0 | 85.0 | 95.4 |
| ID725 | 102.2 | 96.9 | 102.3 | 97.6 | 104.7 | 115.4 | 102.0 | 98.6 | 94.3 | 105.8 | 105.3 | 115.9 |
| ID727 | 130.9 | 133.8 | 141.6 | 108.5 | 128.8 | 125.4 | 91.4 | 85.5 | 80.5 | 93.7 | 75.0 | 67.4 |
| ID728 | 103.1 | 100.3 | 101.0 | 96.5 | 99.7 | 110.1 | 99.4 | 95.4 | 88.2 | 98.9 | 107.2 | 117.2 |
| ID729 | 105.5 | 107.7 | 108.1 | 101.9 | 101.8 | 112.2 | 97.6 | 90.1 | 92.5 | 94.2 | 99.2 | 105.4 |
| ID730 | 82.9 | 83.7 | 72.5 | 110.1 | 93.5 | 115.4 | 159.2 | 139.5 | 103.1 | 147.6 | 177.0 | 156.4 |
| ID731 | 74.8 | 70.9 | 69.0 | 75.6 | 74.9 | 90.7 | 87.6 | 81.4 | 84.5 | 91.5 | 97.7 | 86.3 |
| ID732 | 50.7 | 48.8 | 51.3 | 84.9 | 83.7 | 89.2 | 140.5 | 139.6 | 132.3 | 155.8 | 130.9 | 129.5 |
| ID733 | 83.9 | 90.0 | 90.1 | 91.1 | 89.0 | 109.3 | 93.1 | 95.3 | 90.2 | 104.7 | 102.1 | 83.6 |
| ID736 | 177.2 | 208.5 | 198.7 | 91.1 | 41.3 | 89.8 | 75.6 | 83.9 | 70.4 | 61.9 | 70.6 | 86.7 |
| ID737 | 95.5 | 89.8 | 93.3 | 84.4 | 77.3 | 96.0 | 110.8 | 92.4 | 99.7 | 125.5 | 92.5 | 95.4 |
| ID501 | 206.9 | 334.0 | 290.6 | 79.8 | 86.0 | 104.1 | 23.7 | 49.1 | 31.4 | 40.0 | 42.0 | 27.2 |
| ID739 | 165.3 | 172.4 | 186.1 | 67.3 | 74.1 | 75.8 | 78.5 | 56.5 | 60.9 | 100.4 | 69.4 | 66.0 |
| ID515 | 165.0 | 213.4 | 156.9 | 64.9 | 45.4 | 63.2 | 42.3 | 62.5 | 38.7 | 6.6 | 26.3 | 33.5 |
| ID742 | 101.1 | 97.2 | 96.8 | 86.5 | 76.0 | 100.5 | 85.2 | 80.8 | 89.3 | 95.1 | 94.3 | 89.4 |
| ID745 | 140.3 | 123.7 | 105.3 | 103.9 | 95.4 | 144.9 | 119.2 | 94.3 | 101.1 | 95.3 | 136.5 | 107.9 |
| ID746 | 94.6 | 94.3 | 93.4 | 103.7 | 103.1 | 107.8 | 100.5 | 98.6 | 99.9 | 95.6 | 100.4 | 90.9 |
| ID747 | 77.5 | 143.7 | 152.8 | 112.5 | 108.7 | 108.2 | 111.2 | 103.7 | 119.2 | 111.5 | 66.4 | 90.5 |
| ID748 | 81.6 | 94.4 | 82.4 | 92.0 | 85.2 | 116.1 | 97.4 | 94.5 | 91.0 | 114.3 | 103.1 | 94.2 |
| ID750 | 80.2 | 88.4 | 88.3 | 89.0 | 85.2 | 101.4 | 84.5 | 87.1 | 81.8 | 91.7 | 83.1 | 81.5 |
| ID751 | 192.4 | 233.1 | 193.1 | 103.2 | 106.7 | 109.6 | 115.0 | 101.0 | 96.9 | 102.2 | 86.8 | 82.2 |
| ID753 | 59.7 | 64.2 | 80.0 | 79.5 | 82.4 | 78.9 | 88.3 | 85.6 | 90.5 | 85.7 | 66.1 | 98.6 |
| ID755 | 82.3 | 79.8 | 71.6 | 92.0 | 92.4 | 86.6 | 84.7 | 78.8 | 82.1 | 94.7 | 95.7 | 77.3 |
| ID532 | 62.3 | 53.9 | 63.8 | 62.4 | 84.2 | 71.0 | 81.2 | 90.5 | 81.7 | 98.8 | 100.0 | 103.5 |
| ID757 | 76.5 | 57.1 | 58.9 | 86.6 | 93.0 | 95.1 | 107.3 | 86.9 | 87.0 | 120.8 | 91.8 | 97.6 |
| ID758 | 63.3 | 60.2 | 61.1 | 84.4 | 84.9 | 89.9 | 97.6 | 74.2 | 81.1 | 98.1 | 100.8 | 72.3 |
| ID759 | 119.7 | 115.1 | 120.1 | 112.7 | 111.7 | 121.6 | 121.0 | 110.4 | 113.1 | 102.1 | 113.3 | 113.7 |
| ID760 | 127.1 | 136.4 | 132.6 | 92.1 | 90.5 | 98.7 | 99.5 | 94.5 | 88.3 | 86.5 | 80.9 | 80.1 |
| ID761 | 88.9 | 90.9 | 91.2 | 87.1 | 99.7 | 102.8 | 105.3 | 95.5 | 89.9 | 104.2 | 96.4 | 101.7 |
| ID762 | 100.2 | 102.0 | 100.2 | 91.9 | 93.3 | 102.6 | 105.2 | 100.1 | 95.4 | 96.0 | 98.0 | 100.1 |
| ID763 | 97.6 | 93.2 | 99.4 | 94.2 | 96.4 | 109.8 | 100.5 | 101.6 | 96.6 | 95.9 | 99.7 | 103.7 |
| ID765 | 98.0 | 96.1 | 100.6 | 116.5 | 108.9 | 118.4 | 120.2 | 110.7 | 111.3 | 102.1 | 100.7 | 104.9 |
| ID766 | 183.6 | 178.9 | 172.9 | 62.5 | 72.6 | 73.8 | 65.1 | 52.7 | 68.5 | 75.4 | 91.3 | 85.4 |
| ID767 | 178.3 | 192.2 | 193.7 | 69.0 | 65.9 | 74.2 | 63.8 | 59.4 | 65.1 | 86.2 | 69.8 | 55.2 |
| ID768 | 84.2 | 77.8 | 79.7 | 69.7 | 88.8 | 86.8 | 108.8 | 76.1 | 85.8 | 94.4 | 91.9 | 101.5 |
| ID771 | 81.1 | 78.4 | 60.5 | 100.4 | 92.7 | 97.4 | 113.1 | 107.5 | 71.0 | 97.1 | 85.3 | 102.5 |
| ID772 | 106.7 | 101.6 | 104.6 | 82.3 | 79.9 | 80.4 | 85.4 | 74.7 | 83.8 | 82.4 | 83.3 | 87.8 |
| ID773 | 86.7 | 20.3 | 85.7 | 75.6 | 92.3 | 110.8 | 103.0 | 73.8 | 94.9 | 103.1 | 104.9 | 84.0 |
| ID774 | 84.6 | 95.5 | 88.2 | 83.3 | 92.9 | 96.4 | 108.4 | 94.9 | 92.7 | 95.8 | 92.9 | 97.2 |
| ID775 | 92.3 | 93.8 | 89.3 | 80.4 | 82.9 | 93.8 | 96.1 | 72.0 | 85.9 | 88.8 | 87.1 | 94.4 |
| ID776 | 76.2 | 83.8 | 81.5 | 71.6 | 73.0 | 70.3 | 91.1 | 72.2 | 78.9 | 83.6 | 82.4 | 86.0 |
| ID778 | 95.8 | 109.6 | 95.1 | 89.0 | 88.2 | 96.4 | 91.9 | 76.6 | 79.0 | 92.9 | 77.9 | 86.0 |
| ID779 | 76.5 | 80.1 | 84.4 | 98.9 | 73.1 | 99.7 | 87.8 | 82.2 | 89.0 | 95.8 | 100.6 | 108.6 |
| ID780 | 100.3 | 99.2 | 101.3 | 105.5 | 106.4 | 115.9 | 111.7 | 107.3 | 113.3 | 102.3 | 107.1 | 110.3 |
| ID781 | 77.2 | 90.5 | 77.9 | 93.1 | 66.8 | 107.7 | 102.0 | 79.8 | 83.7 | 108.5 | 112.6 | 82.8 |
| ID565 | 130.0 | 129.7 | 132.3 | 91.9 | 89.8 | 104.1 | 95.5 | 92.5 | 87.4 | 80.0 | 81.2 | 78.1 |
| ID784 | 70.0 | 119.4 | 113.5 | 112.5 | 117.5 | 111.4 | 110.8 | 99.7 | 92.5 | 100.6 | 83.0 | 101.1 |
| ID785 | 105.1 | 99.1 | 103.4 | 101.1 | 98.7 | 115.7 | 98.1 | 87.3 | 86.3 | 69.3 | 67.2 | 68.2 |
| ID786 | 85.8 | 138.4 | 95.1 | 76.9 | 106.5 | 90.0 | 79.6 | 99.5 | 89.3 | 133.6 | 112.1 | 100.5 |
| ID787 | 70.5 | 71.8 | 70.7 | 93.9 | 87.9 | 103.1 | 106.7 | 96.4 | 100.8 | 119.0 | 104.0 | 100.5 |
| ID788 | 100.8 | 94.6 | 96.5 | 104.4 | 101.8 | 122.2 | 105.0 | 96.4 | 94.9 | 82.4 | 83.1 | 80.7 |
| ID789 | 103.4 | 178.1 | 101.1 | 98.8 | 85.6 | 92.3 | 66.3 | 85.9 | 83.3 | 110.0 | 94.8 | 86.7 |
| ID790 | 79.4 | 73.2 | 76.9 | 81.2 | 84.5 | 96.7 | 106.5 | 89.1 | 96.6 | 100.0 | 111.2 | 109.7 |
| ID791 | 100.7 | 108.2 | 112.0 | 79.6 | 77.9 | 82.2 | 90.9 | 87.6 | 83.5 | 96.5 | 76.3 | 81.4 |
| ID792 | 263.7 | 121.8 | 65.8 | 87.2 | 95.3 | 108.8 | 69.2 | 70.4 | 86.8 | 67.6 | 39.4 | 100.0 |
| ID795 | 51.5 | 139.1 | 102.5 | 131.8 | 141.9 | 179.3 | 137.6 | 348.5 | 211.8 | 465.3 | 371.2 | 210.9 |
| ID796 | 82.8 | 91.8 | 50.1 | 119.9 | 122.4 | 212.7 | 160.5 | 272.3 | 112.6 | 392.0 | 325.2 | 303.9 |
| ID797 | 116.7 | 119.0 | 118.5 | 105.7 | 115.5 | 118.0 | 120.5 | 109.7 | 108.4 | 110.3 | 130.6 | 138.7 |
| ID798 | 188.1 | 160.3 | 193.2 | 94.7 | 101.4 | 233.8 | 153.3 | 81.5 | 98.5 | 80.8 | 121.1 | 82.2 |
| ID799 | 164.5 | 34.1 | 32.7 | 39.6 | 105.0 | 181.8 | 125.7 | 44.1 | 126.5 | 55.6 | 293.5 | 169.1 |
| ID800 | 101.7 | 99.1 | 110.7 | 108.5 | 99.8 | 106.0 | 102.4 | 102.3 | 99.6 | 107.8 | 104.1 | 99.8 |
| ID802 | 94.8 | 84.8 | 84.2 | 112.8 | 97.3 | 94.4 | 96.9 | 89.4 | 101.4 | 88.8 | 80.5 | 89.0 |
| ID804 | 70.6 | 67.0 | 72.3 | 100.4 | 92.4 | 107.7 | 110.0 | 102.0 | 107.0 | 111.9 | 116.7 | 117.3 |
| ID805 | 101.7 | 95.8 | 92.6 | 98.4 | 90.9 | 96.7 | 91.6 | 83.1 | 89.0 | 85.7 | 77.6 | 86.0 |
| ID806 |  | 222.6 | 234.0 | 135.3 | 220.6 | 271.0 | 94.6 | 157.1 | 109.4 | 51.8 |  | 164.9 |
| ID807 | 117.2 | 146.7 | 131.7 | 105.3 | 92.7 | 102.1 | 81.0 | 88.6 | 72.2 | 92.2 | 70.9 | 66.1 |
| ID808 | 143.1 | 133.0 | 134.2 | 140.3 | 119.6 | 134.0 | 135.8 | 121.8 | 118.7 | 96.9 | 113.5 | 108.0 |
| ID809 | 80.0 | 69.8 | 75.3 | 97.2 | 89.6 | 104.7 | 108.7 | 100.2 | 106.6 | 116.1 | 126.8 | 122.6 |
| ID811 | 80.9 | 71.9 | 70.3 | 79.4 | 76.9 | 81.6 | 80.7 | 70.0 | 79.1 | 73.4 | 81.5 | 82.1 |
| ID812 | 98.7 | 103.7 | 105.0 | 95.0 | 92.9 | 103.4 | 102.9 | 98.1 | 96.2 | 100.3 | 95.1 | 90.4 |
| ID813 | 92.1 | 93.1 | 94.4 | 84.3 | 82.5 | 87.1 | 94.2 | 83.3 | 88.2 | 84.4 | 86.1 | 88.6 |
| ID814 | 145.9 | 122.7 | 125.2 | 115.1 | 123.2 | 96.1 | 110.8 | 99.5 | 107.4 | 75.8 | 66.3 | 90.2 |
| ID815 | 154.3 | 153.8 | 138.8 | 124.1 | 106.7 | 106.1 | 131.0 | 123.6 | 119.8 | 134.1 | 104.6 | 123.5 |
| ID816 | 172.0 | 125.9 | 135.6 | 77.6 | 108.7 | 114.2 | 98.9 | 65.6 | 85.0 | 76.8 | 135.9 | 119.6 |
| ID817 | 511.7 | 108.3 | 111.7 | 121.9 | 122.7 | 128.4 | 113.3 | 94.7 | 96.3 | 65.6 | 6.1 | 69.3 |
| ID818 | 158.7 | 122.2 | 156.3 | 100.6 | 103.4 | 93.4 | 91.2 | 104.2 | 104.2 | 84.6 | 111.6 | 99.7 |
| ID819 | 100.1 | 102.6 | 102.1 | 98.4 | 95.5 | 114.6 | 95.2 | 91.6 | 85.6 | 95.3 | 98.3 | 87.9 |
| ID820 | 168.8 | 90.4 | 113.0 | 72.6 | 104.8 | 121.8 | 103.6 | 84.3 | 104.7 | 87.7 | 179.5 | 124.5 |
| ID822 | 82.6 | 77.8 | 84.6 | 87.9 | 82.8 | 94.3 | 94.1 | 88.5 | 94.5 | 106.3 | 115.5 | 110.7 |
| ID823 | 106.3 | 118.5 | 116.7 | 93.5 | 98.1 | 113.1 | 106.0 | 110.0 | 93.1 | 108.6 | 123.1 | 116.1 |
| ID824 | 147.1 | 170.7 | 162.4 | 64.2 | 60.7 | 76.4 | 41.6 | 42.4 | 34.1 | 23.5 | 24.3 | 19.1 |
| ID607 | 121.2 | 138.4 | 132.3 | 93.6 | 83.9 | 97.0 | 77.1 | 74.2 | 73.4 | 47.5 | 44.9 | 54.6 |
| ID826 | 107.7 | 101.0 | 111.6 | 73.0 | 72.7 | 95.5 | 90.8 | 77.8 | 84.9 | 77.5 | 91.9 | 90.2 |
| ID827 | 94.6 | 90.4 | 96.8 | 81.0 | 80.9 | 87.7 | 83.5 | 80.0 | 84.0 | 90.2 | 100.3 | 97.5 |
| ID828 | 94.2 | 93.7 | 94.7 | 93.8 | 93.3 | 105.7 | 88.4 | 87.4 | 88.9 | 96.1 | 97.8 | 99.8 |
| ID829 | 82.8 | 85.6 | 85.7 | 101.1 | 97.1 | 103.1 | 126.5 | 108.8 | 109.4 | 113.2 | 106.8 | 105.1 |
| ID830 | 81.0 | 72.1 | 82.1 | 80.8 | 78.0 | 104.7 | 109.1 | 96.1 | 100.7 | 126.8 | 151.6 | 132.8 |
| ID833 | 107.9 | 113.7 | 112.5 | 99.2 | 126.1 | 108.0 | 149.9 | 108.4 | 127.6 | 96.3 | 114.1 | 137.4 |
| ID834 | 89.6 | 86.9 | 97.5 | 107.4 | 111.2 | 120.5 | 112.6 | 104.8 | 100.3 | 95.4 | 96.1 | 95.4 |
| ID8346 | 356.9 | 461.8 | 448.6 | 54.4 | 57.1 | 54.8 | 21.1 | 21.9 | 19.0 | 11.5 | 24.6 | 28.0 |
| ID835 | 108.6 | 107.2 | 103.9 | 104.9 | 102.8 | 100.5 | 106.4 | 89.8 | 101.1 | 92.4 | 95.9 | 108.0 |
| ID836 | 57.0 | 51.5 | 53.3 | 80.9 | 76.4 | 78.1 | 111.6 | 89.6 | 102.3 | 115.4 | 109.4 | 113.3 |
| ID837 | 118.3 | 61.5 | 66.5 | 104.3 | 105.4 | 128.8 | 145.9 | 98.0 | 132.7 | 113.9 | 156.2 | 130.5 |
| ID838 | 105.2 | 108.0 | 99.6 | 107.6 | 105.1 | 102.4 | 109.4 | 87.7 | 99.0 | 93.7 | 92.8 | 108.4 |
| ID840 | 99.1 | 111.3 | 106.8 | 96.8 | 91.2 | 107.7 | 95.0 | 95.0 | 89.6 | 88.5 | 91.7 | 84.0 |
| ID841 | 101.9 | 115.8 | 114.2 | 92.0 | 98.2 | 100.9 | 105.7 | 97.4 | 98.6 | 101.7 | 102.3 | 103.8 |
| ID843 | 125.0 | 145.9 | 131.6 | 107.3 | 101.2 | 110.2 | 104.7 | 98.8 | 95.8 | 93.0 | 93.1 | 92.2 |
| ID84360 | 108.7 | 99.5 | 101.8 | 115.7 | 112.0 | 124.0 | 102.0 | 106.0 | 100.6 | 99.4 | 91.3 | 86.1 |
| ID844 | 102.5 | 95.6 | 93.1 | 91.3 | 88.4 | 83.7 | 94.3 | 73.1 | 90.5 | 72.7 | 81.9 | 95.6 |
| ID84422 | 59.1 | 94.9 | 68.0 | 103.7 | 87.6 | 97.2 | 108.1 | 84.5 | 74.4 | 121.0 | 79.4 | 102.4 |
| ID845 | 138.8 | 126.2 | 119.1 | 84.2 | 92.1 | 90.8 | 96.7 | 85.2 | 84.3 | 99.3 | 80.0 | 94.6 |
| ID847 | 122.9 | 116.6 | 117.9 | 96.9 | 98.2 | 101.3 | 103.0 | 89.1 | 96.3 | 87.7 | 96.1 | 107.2 |
| ID848 | 110.2 | 92.3 | 97.2 | 81.3 | 84.8 | 80.2 | 88.0 | 69.6 | 84.2 | 69.2 | 80.9 | 92.9 |
| ID849 | 89.1 | 79.5 | 80.9 | 74.6 | 73.1 | 71.5 | 79.2 | 64.0 | 75.4 | 64.2 | 77.7 | 83.1 |
| ID84991 | 112.5 | 99.5 | 110.4 | 122.6 | 115.4 | 124.2 | 99.6 | 112.5 | 100.3 | 103.6 | 91.8 | 92.8 |
| ID851 | 122.1 | 116.4 | 117.3 | 100.5 | 97.0 | 99.9 | 98.7 | 85.9 | 94.7 | 85.8 | 90.3 | 103.2 |
| ID852 | 91.8 | 91.1 | 92.2 | 109.3 | 98.3 | 112.1 | 97.7 | 98.1 | 99.5 | 101.7 | 92.3 | 92.8 |
| ID853 | 104.7 | 106.6 | 112.1 | 134.4 | 109.0 | 131.9 | 100.2 | 105.3 | 101.2 | 112.7 | 83.5 | 91.2 |
| ID641 | 62.8 | 48.1 | 51.2 | 81.2 | 74.2 | 72.2 | 94.6 | 81.4 | 95.4 | 118.3 | 122.3 | 131.5 |
| ID645 | 186.7 | 198.7 | 198.9 | 61.1 | 64.0 | 67.2 | 69.3 | 56.4 | 58.9 | 60.9 | 56.3 | 49.2 |
| ID857 | 186.9 | 205.8 | 197.0 | 82.1 | 80.7 | 89.9 | 59.9 | 61.0 | 57.7 | 48.2 | 51.1 | 53.0 |
| ID858 | 111.1 | 109.9 | 111.3 | 99.9 | 90.6 | 104.3 | 88.8 | 91.0 | 88.2 | 83.5 | 77.4 | 78.9 |
| ID859 | 167.0 | 189.7 | 166.6 | 107.1 | 100.8 | 102.9 | 103.8 | 105.1 | 94.5 | 91.4 | 81.7 | 99.3 |
| ID860 | 158.4 | 165.6 | 162.0 | 97.5 | 86.1 | 92.1 | 78.2 | 76.1 | 79.7 | 69.6 | 62.6 | 75.0 |
| ID86104 | 131.3 | 103.4 | 102.3 | 119.2 | 106.9 | 121.4 | 99.4 | 92.2 | 94.4 | 86.3 | 87.0 | 81.1 |
| ID86191 | 106.5 | 107.4 | 117.0 | 123.5 | 116.7 | 118.8 | 95.7 | 112.8 | 108.8 | 109.3 | 104.1 | 93.8 |
| ID863 | 77.4 | 77.4 | 85.2 | 97.6 | 91.8 | 106.2 | 89.7 | 95.3 | 90.6 | 94.4 | 91.4 | 86.0 |
| ID659 | 352.1 | 309.4 | 279.0 | 56.8 | 55.8 | 35.1 | 23.3 | 38.6 | 33.1 | 34.0 | 23.8 | 20.0 |
| ID653 | 144.3 | 168.9 | 167.5 | 77.1 | 89.7 | 97.6 | 62.8 | 58.1 | 57.1 | 83.4 | 69.2 | 75.3 |
| ID871 | 111.9 | 102.6 | 102.4 | 124.3 | 124.6 | 117.1 | 136.4 | 112.6 | 129.7 | 110.6 | 110.6 | 128.0 |
| ID872 | 8.6 | 84.2 | 90.4 | 47.5 | 62.9 | 119.1 | 17.1 | 95.1 | 76.5 | 96.3 | 96.7 | 51.8 |
| ID873 | 151.4 | 128.3 | 134.4 | 73.3 | 115.9 | 79.3 | 109.5 | 66.8 | 82.4 | 171.1 | 123.1 | 146.3 |
| ID87303 | 170.6 | 201.3 | 108.4 | 170.2 | 95.7 | 100.2 | 81.5 | 82.3 | 91.0 | 88.1 | 45.4 | 68.4 |
| ID878 | 120.4 | 139.0 | 115.3 | 143.9 | 156.1 | 147.1 | 178.1 | 165.4 | 132.4 | 122.2 | 139.2 | 147.0 |
| ID879 | 94.8 | 50.3 | 51.9 | 79.2 | 85.8 | 59.4 | 104.4 | 72.0 | 98.0 | 73.4 | 92.3 | 111.5 |
| ID87904 | 115.5 | 156.5 | 98.5 | 94.1 | 80.4 | 112.4 | 100.4 | 77.5 | 87.1 | 83.3 | 82.6 | 77.4 |
| ID88029 | 131.4 | 171.1 | 120.0 | 108.3 | 81.2 | 98.1 | 104.7 | 92.4 | 76.9 | 75.7 | 66.8 | 115.3 |
| ID885 | 79.6 | 88.8 | 92.1 | 96.7 | 92.3 | 114.9 | 99.2 | 98.2 | 91.3 | 102.7 | 98.6 | 93.0 |
| ID886 | 120.6 | 83.3 | 90.4 | 84.5 | 129.0 | 145.2 | 136.7 | 126.3 | 113.5 | 132.8 | 135.1 | 117.7 |
| ID88622 | 88.6 | 80.3 | 107.0 | 73.1 | 75.5 | 81.3 | 77.4 | 65.1 | 78.0 | 94.1 | 74.8 | 83.4 |
| ID887 | 83.6 | 84.1 | 94.2 | 83.7 | 104.4 | 121.9 | 111.7 | 97.9 | 96.3 | 88.8 | 76.9 | 66.2 |
| ID888 | 175.8 | 167.3 | 217.1 | 102.4 | 124.1 | 127.4 | 134.6 | 131.1 | 114.2 | 119.3 | 143.6 | 119.8 |
| ID889 | 87.5 | 83.9 | 92.5 | 100.7 | 95.5 | 99.6 | 112.6 | 101.3 | 103.3 | 111.7 | 115.8 | 108.2 |
| ID891 | 69.4 | 77.4 | 82.7 | 78.2 | 72.0 | 93.6 | 66.8 | 81.3 | 69.8 | 89.1 | 67.1 | 60.0 |
| ID695 | 142.5 | 149.2 | 142.0 | 77.8 | 81.9 | 91.2 | 83.6 | 89.5 | 82.6 | 66.1 | 72.6 | 87.2 |
| ID89219 | 50.5 | 105.2 | 45.6 | 77.1 | 77.4 | 79.5 | 90.1 | 74.4 | 84.2 | 89.1 | 110.9 | 90.9 |
| ID894 | 638.4 | 382.2 | 575.0 | 665.6 | 790.9 | 591.9 | 811.3 | 519.3 | 940.8 | 553.8 | 491.9 | 819.8 |
| ID707 | 134.6 | 152.1 | 131.9 | 105.9 | 99.7 | 91.8 | 101.8 | 72.9 | 84.3 | 50.4 | 49.5 | 59.0 |
| ID896 | 148.8 | 125.2 | 148.3 | 81.6 | 70.5 | 84.6 | 67.0 | 63.3 | 74.8 | 72.4 | 77.3 | 62.3 |
| ID708 | 119.2 | 143.1 | 124.2 | 93.0 | 89.7 | 91.8 | 98.4 | 85.6 | 89.7 | 77.5 | 72.3 | 75.3 |
| ID89827 | 96.5 | 85.3 | 85.9 | 87.6 | 96.8 | 94.2 | 105.4 | 92.7 | 93.8 | 95.7 | 95.8 | 95.2 |
| ID899 | 114.7 | 98.3 | 108.9 | 74.0 | 89.1 | 129.8 | 94.1 | 56.3 | 72.7 | 35.6 | 99.5 | 69.1 |
| ID710 | 161.0 | 207.3 | 182.6 | 67.2 | 64.1 | 75.7 | 63.3 | 62.4 | 61.7 | 67.0 | 67.1 | 62.6 |
| ID901 | 4.4 | 38.5 | 32.1 | 7.8 | 6.5 | 33.3 | 2.9 | 43.5 | 6.4 | 36.3 | 37.8 | 11.1 |
| ID903 | 191.9 | 175.8 | 190.9 | 101.3 | 91.4 | 108.4 | 117.3 | 74.6 | 91.4 | 218.2 | 86.2 | 77.4 |
| ID904 | 37.1 | 64.7 | 58.4 | 67.6 | 59.2 | 88.0 | 67.8 | 90.2 | 62.5 | 104.8 | 90.9 | 73.9 |
| ID90440 | 123.3 | 116.9 | 118.6 | 116.0 | 102.8 | 120.1 | 106.2 | 102.7 | 104.1 | 103.9 | 94.7 | 98.6 |
| ID905 | 58.6 | 53.1 | 51.3 | 123.6 | 137.7 | 129.7 | 180.8 | 110.7 | 139.3 | 98.1 | 113.1 | 114.5 |
| ID906 | 94.0 | 89.3 | 92.2 | 120.9 | 119.5 | 118.1 | 127.7 | 107.4 | 114.5 | 98.4 | 101.6 | 110.1 |
| ID907 | 116.6 | 117.4 | 109.7 | 102.8 | 97.5 | 100.6 | 106.9 | 96.8 | 109.6 | 101.2 | 109.5 | 106.1 |
| ID910 | 90.4 | 108.0 | 102.2 | 97.4 | 89.3 | 107.9 | 96.3 | 99.3 | 91.2 | 96.1 | 99.2 | 86.7 |
| ID91078 | 94.8 | 68.8 | 82.9 | 72.8 | 83.9 | 69.9 | 103.0 | 110.3 | 112.4 | 111.7 | 106.8 | 116.6 |
| ID912 | 216.3 | 207.1 | 207.1 | 177.4 | 170.6 | 198.7 | 178.3 | 214.7 | 186.3 | 211.3 | 191.2 | 169.0 |
| ID914 | 79.7 | 61.5 | 79.7 | 76.0 | 74.3 | 89.9 | 100.0 | 86.2 | 109.0 | 128.2 | 194.2 | 212.9 |
| ID915 | 145.1 | 186.7 | 162.0 | 61.2 | 53.3 | 63.9 | 45.9 | 48.5 | 50.2 | 55.1 | 49.0 | 39.2 |
| ID916 | 80.3 | 93.3 | 92.7 | 99.2 | 92.7 | 102.2 | 128.2 | 110.2 | 109.4 | 121.4 | 103.2 | 120.2 |
| ID920 | 88.3 | 109.9 | 106.2 | 89.7 | 84.2 | 104.2 | 89.6 | 95.6 | 85.6 | 92.6 | 97.7 | 88.3 |
| ID92193 | 120.3 | 159.7 | 140.0 | 109.4 | 112.5 | 108.0 | 145.3 | 107.7 | 114.8 | 110.3 | 129.6 | 115.7 |
| ID922 | 83.0 | 87.4 | 93.8 | 83.6 | 120.5 | 93.0 | 92.6 | 87.3 | 88.9 | 135.3 | 96.7 | 99.5 |
| ID92323 | 124.7 | 149.2 | 139.1 | 105.6 | 96.0 | 104.4 | 134.8 | 93.4 | 103.3 | 95.2 | 101.9 | 101.6 |
| ID924 | 175.4 | 260.3 | 260.9 | 10.2 | 19.0 | 34.1 | 24.5 | 33.6 | 13.8 | 26.4 | 38.2 | 32.4 |
| ID92960 | 128.0 | 136.3 | 164.4 | 105.6 | 105.3 | 132.9 | 178.6 | 99.1 | 109.1 | 98.6 | 121.0 | 123.2 |
| ID931 | 126.5 | 168.4 | 173.7 | 36.9 | 33.5 | 50.4 | 36.9 | 36.5 | 38.1 | 29.5 | 49.6 | 38.5 |
| ID933 | 108.1 | 144.4 | 193.0 | 208.9 | 222.6 | 183.4 | 202.9 | 123.5 | 235.5 | 131.1 | 103.0 | 142.1 |
| ID763 | 153.8 | 206.5 | 196.3 | 70.3 | 71.9 | 80.2 | 65.3 | 56.6 | 60.6 | 55.7 | 57.6 | 47.6 |
| ID93595 | 119.0 | 112.8 | 107.7 | 118.6 | 195.7 | 199.5 | 169.1 | 70.3 | 110.4 | 766.1 | 142.8 | 399.8 |
| ID938 | 127.4 | 118.1 | 123.2 | 101.6 | 107.8 | 105.7 | 101.6 | 89.5 | 107.6 | 92.0 | 80.7 | 95.9 |
| ID939 | 156.9 | 142.0 | 168.8 | 230.6 | 191.2 | 190.2 | 244.5 | 124.5 | 226.1 | 128.3 | 103.6 | 167.7 |
| ID940 | 170.5 | 157.5 | 172.3 | 54.6 | 73.7 | 74.8 | 102.1 | 83.7 | 84.4 | 84.9 | 73.8 | 85.5 |
| ID94178 | 165.8 | 94.5 | 87.6 | 50.1 | 60.1 | 73.9 | 74.8 | 54.4 | 67.1 | 51.3 | 97.4 | 85.0 |
| ID942 | 77.3 | 88.2 | 83.7 | 78.0 | 70.4 | 89.2 | 77.6 | 81.7 | 74.4 | 80.1 | 86.6 | 75.4 |
| ID774 | 177.4 | 227.5 | 193.5 | 95.1 | 97.0 | 97.6 | 63.5 | 56.3 | 59.6 | 45.2 | 46.4 | 40.1 |
| ID944 | 153.5 | 151.4 | 165.1 | 77.9 | 97.4 | 93.2 | 65.8 | 61.2 | 66.1 | 91.2 | 54.5 | 69.4 |
| ID776 | 197.7 | 224.2 | 235.5 | 82.2 | 79.3 | 100.2 | 62.5 | 44.1 | 49.4 | 35.5 | 42.6 | 27.2 |
| ID947 | 76.0 | 138.0 | 94.8 | 70.3 | 41.3 | 47.1 | 50.3 | 58.5 | 41.7 | 31.9 | 25.1 | 48.3 |
| ID948 | 105.8 | 99.3 | 101.5 | 82.5 | 83.9 | 105.2 | 98.6 | 80.6 | 86.2 | 102.6 | 103.4 | 87.3 |
| ID94856 | 142.2 | 175.0 | 161.7 | 87.7 | 104.7 | 103.2 | 96.2 | 101.3 | 83.3 | 111.0 | 159.8 | 127.1 |
| ID949 | 27.7 | 26.2 | 24.6 | 16.7 | 14.2 | 26.8 | 8.6 | 21.2 | 12.3 | 26.3 | 38.5 | 10.0 |
| ID952 | 71.3 | 55.6 | 49.9 | 40.9 | 35.7 | 34.2 | 39.1 | 38.7 | 37.0 | 41.9 | 30.8 | 34.3 |
| ID953 | 80.7 | 86.5 | 89.3 | 112.4 | 107.7 | 124.0 | 108.9 | 108.3 | 101.5 | 104.7 | 92.3 | 95.4 |
| ID954 | 87.2 | 87.0 | 90.9 | 115.0 | 113.0 | 116.9 | 121.5 | 102.8 | 109.6 | 97.9 | 99.2 | 104.7 |
| ID95466 | 74.6 | 59.2 | 62.1 | 58.0 | 64.0 | 61.4 | 67.3 | 66.8 | 62.1 | 57.9 | 85.7 | 75.6 |
| ID955 | 123.9 | 137.4 | 138.5 | 94.2 | 95.6 | 92.4 | 101.5 | 84.5 | 95.8 | 87.5 | 96.7 | 101.8 |
| ID957 | 148.5 | 125.3 | 109.0 | 284.5 | 179.5 | 210.1 | 142.0 | 199.1 | 132.0 | 167.7 | 93.9 | 154.8 |
| ID960 | 69.6 | 55.0 | 56.6 | 80.3 | 82.0 | 80.0 | 97.6 | 66.6 | 87.9 | 78.8 | 92.7 | 107.2 |
| ID961 | 84.8 | 84.2 | 89.4 | 111.2 | 105.5 | 111.4 | 115.7 | 95.7 | 103.5 | 93.9 | 96.3 | 102.5 |
| ID963 | 65.8 | 46.1 | 48.2 | 76.1 | 87.4 | 65.6 | 85.0 | 64.8 | 92.6 | 73.1 | 78.1 | 87.0 |
| ID964 | 159.2 | 171.7 | 160.4 | 69.3 | 69.5 | 68.7 | 73.4 | 65.2 | 68.7 | 72.7 | 76.8 | 80.1 |
| ID966 | 135.2 | 131.1 | 130.5 | 84.5 | 81.3 | 92.2 | 88.6 | 85.8 | 82.3 | 83.3 | 74.0 | 76.2 |
| ID967 | 287.7 | 342.8 | 332.7 | 43.6 | 41.2 | 49.3 | 23.0 | 20.9 | 20.3 | 16.8 | 31.4 | 22.5 |
| ID96711 | 78.2 | 92.4 | 111.6 | 66.2 | 69.4 | 74.7 | 61.8 | 88.2 | 56.5 | 58.2 | 77.0 | 81.4 |
| ID968 | 104.7 | 92.4 | 91.9 | 104.8 | 110.1 | 109.9 | 103.0 | 83.1 | 96.7 | 81.8 | 74.0 | 82.9 |
| ID969 | 87.9 | 78.8 | 84.1 | 107.0 | 118.7 | 110.7 | 123.5 | 90.0 | 106.6 | 87.6 | 89.9 | 96.9 |
| ID972 | 205.5 | 113.6 | 147.8 | 208.7 | 209.4 | 233.2 | 105.5 | 120.6 | 156.0 | 87.7 | 76.0 | 69.3 |
| ID97373 | 83.3 | 109.3 | 91.1 | 79.0 | 75.0 | 76.0 | 98.1 | 83.2 | 76.8 | 106.2 | 100.9 | 78.8 |
| ID974 | 186.3 | 171.5 | 172.0 | 81.6 | 68.4 | 77.4 | 91.2 | 73.1 | 80.4 | 54.3 | 70.7 | 83.8 |
| ID97436 | 68.5 | 181.9 | 126.5 | 96.6 | 75.9 | 86.4 | 66.7 | 91.0 | 79.4 | 64.3 | 91.6 | 36.5 |
| ID976 | 96.7 | 105.1 | 100.3 | 101.8 | 105.4 | 113.4 | 106.1 | 87.3 | 86.6 | 79.9 | 74.6 | 79.6 |
| ID977 | 114.6 | 139.4 | 123.9 | 124.6 | 119.9 | 120.3 | 140.7 | 110.2 | 136.3 | 114.7 | 133.6 | 125.0 |
| ID980 | 72.3 | 88.5 | 113.0 | 127.2 | 110.4 | 118.6 | 117.2 | 107.4 | 107.0 | 101.9 | 134.6 | 119.8 |
| ID98107 | 57.3 | 61.7 | 40.3 | 48.6 | 84.5 | 50.4 | 77.6 | 121.9 | 64.6 | 104.5 | 100.4 | 56.8 |
| ID983 | 120.7 | 121.7 | 123.3 | 109.5 | 104.8 | 105.6 | 109.4 | 100.7 | 106.1 | 99.7 | 98.1 | 100.3 |
| ID986 | 97.1 | 82.9 | 84.4 | 105.6 | 96.3 | 98.6 | 94.0 | 86.1 | 98.3 | 85.1 | 76.4 | 90.6 |
| ID989 | 266.4 | 171.4 | 327.5 | 140.5 | 132.1 | 86.8 | 121.0 | 156.9 | 214.5 | 104.0 | 109.2 | 179.5 |
| ID991 | 112.5 | 92.6 | 98.5 | 132.8 | 135.7 | 133.5 | 144.3 | 96.7 | 133.6 | 104.3 | 85.3 | 125.0 |
| ID992 | 111.1 | 87.1 | 99.3 | 130.6 | 152.6 | 175.4 | 446.5 | 125.7 | 139.8 | 122.7 | 107.2 | 152.1 |
| ID993 | 163.5 | 136.5 | 176.7 | 85.4 | 115.6 |  |  | 68.0 | 100.8 | 86.5 | 74.8 | 109.5 |
| ID994 | 116.9 | 121.0 | 121.8 | 107.0 | 102.7 | 117.6 | 129.9 | 86.4 | 100.8 | 95.3 | 95.1 | 101.4 |
| ID996 | 58.3 | 67.1 | 72.7 | 126.5 | 110.0 | 125.4 | 135.9 | 124.8 | 119.1 | 123.4 | 146.2 | 146.5 |
| ID997 | 91.4 | 107.0 | 105.4 | 133.0 | 112.0 | 116.4 | 126.7 | 103.5 | 102.6 | 102.1 | 125.9 | 119.4 |
| ID999 | 154.3 | 132.0 | 162.4 | 101.7 | 136.2 | 94.4 | 104.3 | 80.4 | 96.5 | 116.8 | 93.3 | 111.6 |

^a^ Due to limited space, only age-associated spot ID’s (spots highlighted in yellow) are shown on the 2D-DIGE image presented in Figure 1 . Should you require information on the location on 2-D-DIGE Image of other spot ID’s in this table, please contact the corresponding author

**Reference**

[1]	Sikulu MT, Monkman J, Dave KA, Hastie ML, Dale PE, Kitching RL, Killeen GF, Kay BH, Gorman JJ, Hugo L**E**. Proteomic changes occurring in the malaria mosquitoes Anopheles gambiae and Anopheles stephensi during aging. Journal of Proteomi*c*s 2015; 126:234-44.
